# Leveraging pQTL-based Mendelian randomization to identify new treatment prospects for primary biliary cholangitis and primary sclerosing cholangitis

**DOI:** 10.18632/aging.205867

**Published:** 2024-05-27

**Authors:** Lei Dai, Yunyan Ye, Joseph Mugaany, Zetong Hu, Jing Huang, Changjiang Lu

**Affiliations:** 1 Department of Hepato-Pancreato-Biliary Surgery, Ningbo Medical Centre Lihuili Hospital, The Affiliated Hospital of Ningbo University, Ningbo, Zhejiang 315040, China; 2 Department of Ophthalmology, Ningbo Medical Centre Lihuili Hospital, The Affiliated Hospital of Ningbo University, Ningbo, Zhejiang 315040, China; 3 Health Science Center, Ningbo University, Ningbo 315211, China

**Keywords:** primary biliary cholangitis, primary sclerosing cholangitis, Mendelian randomization, drug, SNPs

## Abstract

Primary biliary cholangitis (PBC) and primary sclerosing cholangitis (PSC) are autoimmune disorders characterized by progressive and chronic damage to the bile ducts, presenting clinicians with significant challenges. The objective of this study is to identify potential druggable targets to offer new avenues for treatment. A Mendelian randomization analysis was performed to identify druggable targets for PBC and PSC. This involved obtaining Cis-protein quantitative trait loci (Cis-pQTL) data from the deCODE database to serve as exposure. Outcome data for PBC (557 cases and 281,127 controls) and PSC (1,715 cases and 330,903 controls) were obtained from the FINNGEN database. Colocalization analysis was conducted to determine whether these features share the same associated SNPs. Validation of the expression level of druggable targets was done using the GSE119600 dataset and immunohistochemistry for clinical samples. Lastly, the DRUGBANK database was used to predict potential drugs. The MR analysis identified eight druggable targets each for PBC and PSC. Subsequent summary-data-based MR and colocalization analyses showed that LEFTY2 had strong evidence as a therapeutic candidate for PBC, while HSPB1 had moderate evidence. For PSC, only FCGR3B showed strong evidence as a therapeutic candidate. Additionally, upregulated expression of these genes was validated in PBC and PSC groups by GEO dataset and clinical samples. This study identifies two novel druggable targets with strong evidence for therapeutic candidates for PBC (LEFTY2 and HSPB1) and one for PSC (FCGR3B). These targets offer new therapeutic opportunities to address the challenging nature of PBC and PSC treatment.

## INTRODUCTION

Chronic autoimmune liver diseases, specifically primary biliary cholangitis (PBC) and primary sclerosing cholangitis (PSC), demand urgent intervention from medical professionals [1–5]. PBC leads to progressive destruction of small bile ducts, impairing bile flow, [1] while PSC inflames and scars the bile ducts, resulting in narrowing and obstruction [6]. Both diseases pose significant health risks, are major causes of liver transplantation [3, 7]. Early detection and monitoring play a crucial role in managing PBC and PSC, while effective pharmacological treatments remain limited. While symptomatic relief can be partially achieved through immunosuppressants and ursodeoxycholic acid (UDCA), [8, 9] there is a pressing necessity to identify new therapeutic targets for both diseases. Identifying effective drugs to prevent the progression and complications of PBC and PSC is crucial in improving patient outcomes and reducing the burden on healthcare systems. Comprehensive research efforts are needed to explore new avenues for treatment and advance our understanding of the underlying mechanisms driving these diseases.

To accurately gauge the efficacy of drug treatment strategies, the adoption of a large-scale randomized clinical trial (RCT) or real-world experience proves highly advantageous. Nevertheless, meticulous planning, extensive investment in design and execution, along with resource allocation, are imperative prerequisites. Notably, over the past few years, this has emerged as the most economical approach for incorporating human genetics studies into pharmacological innovation efforts. Although UDCA and obeticholic acid (OCA) have demonstrated effectiveness in partial patients with PBC and PSC, no curable medication has come out yet [10, 11].

In the pursuit of investigating causal relationships, Mendelian randomization (MR) offers an approach that relies on common genetic variants as unbiased proxies [12]. When analyzing drug targets, researchers often turn to protein quantitative trait loci (pQTL) situated within the genomic region of the target gene [13]. These pQTLs serve as regulators, exerting influence over gene expression [14–18]. The utility of MR analyses has been demonstrated in various diseases, such as COVID-19 [19], Parkinson’s disease [20], and Aortic aneurysms (AAs) [21]. Intriguingly, in the case of PBC, studies employing genetic markers have identified three plasma proteins (CD40, ficolin-1, and protein FAM177A1) that exhibit potential protective influences on PBC [22]. Moreover, several other potential biomarkers, such as SPATA31A3, GARP [23], anti-Sp100, and anti-gp210 [24], have also been unveiled. However, the exploration of genomic evidence for a broader array of potential drug targets for PBC and PSC remains untapped.

In this study, our objective was to identify potential pharmacological targets for mitigating the progression of PBC and PSC. We employed MR analyses, integrating pQTL data from two distinct plasma proteome datasets with independent PBC and PSC datasets sourced from FINNGEN. Specifically, we explored the association between genetically mediated druggable genes and the risks associated with PBC and PSC, along with investigating the corresponding drugs and their mechanisms of action. The findings from our research provide crucial insights into the underlying pathophysiology of PBC and PSC. Building upon these discoveries, additional experiments can be conducted in both *in vitro* and *in vivo* models, aiming to validate the feasibility of these targets and assess the therapeutic efficacy of relevant pharmacological interventions.

## MATERIALS AND METHODS

### Ethical approval

We conducted a secondary analysis of publicly available and accessible data. In accordance with the initial GWAS protocols, the original authors obtained necessary ethical approval and all participants provided informed consent. The research ethics committee at Lihuili Hospital, affiliated with Ningbo University (Approval No. KY2023ML052), granted approval for the implementation of research procedures involving human subjects. Written informed consent was obtained from all patients, following the principles outlined in the Helsinki Declaration of 1964 and its subsequent revisions, as well as relevant ethical standards, prior to their enrollment.

### Patent gene selection

We obtained a total of 4,479 druggable genes from “The druggable genome and support for target identification and validation in drug development [25]” (Supplementary Table 1). According to this study, the druggable genes can be divided into three categories. The first category includes 1,427 genes that are therapeutic targets of approved small molecules and biologic drugs, as well as clinical candidates. The second category consists of 682 genes encoding targets with known bioactive small molecule binding partners and at least 50% similarity to approved drug targets. The third category comprises 2,370 genes encoding secreted or extracellular proteins that have lower similarity to approved drug targets, and those are key members of druggable gene families not covered by the initial two categories.

### pQTL dataset

pQTL refers to the study of the correlation between genetic variations and gene expression by treating protein expression as a phenotype. Here, we conducted the analysis using pQTL data from two sources. The first set of pQTL data was obtained from the deCODE database [26], which includes 4,907 proteins and is derived from Large-scale integration of the plasma proteome with genetics and disease. This dataset was primarily used for identifying potential drug targets related to PBC and PSC. The cis-pQTL screening criteria were as follows: (1) *p*-value < 5e-08, (2) removal of SNPs in the major histocompatibility complex (MHC) region, (3) SNPs within 1 Mb upstream and downstream of the gene, (4) removal of SNPs with linkage disequilibrium r2 < 0.1. The second set of pQTL data was derived from Phenome-wide Mendelian randomization mapping the influence of the plasma proteome on complex diseases [27] and used to replicate the findings from the deCODE dataset. It includes data from five GWAS summary datasets. The cis-pQTL screening criteria were as follows: (1) *p*-value < 5e-08, (2) removal of SNPs in the major histocompatibility complex (MHC) region, (3) SNPs within 500 kb upstream and downstream of the leading pQTL, (4) removal of SNPs with linkage disequilibrium r2 < 0.001. The selected datasets were all of European ancestry.

### The outcome dataset

We utilized data from the FINNGEN database [28] (https://www.finngen.fi/en) on PBC and PSC as outcome data. The data were adjusted for gender, age, genetic typing, and 10 principal components as covariates. Detailed data information was shown in Supplementary Table 2. The selected datasets included individuals of European ancestry.

### Two-sample Mendelian randomization

Two-sample Mendelian randomization analysis was conducted using the TwoSampleMR package [29]. Expression levels of druggable proteins from the deCODE database served as the exposure variables, while PBC and PSC were considered as the outcome variables. The Wald ratio method was employed to assess the causal effects of exposures with a single SNP, whereas the inverse variance weighted (IVW) method was used for exposures with two or more SNPs. Heterogeneity testing, multiplicity testing, leave-one-out analysis, directional tests using Steiger’s test, and directional filtering were performed to establish the directionality of causation.

### SMR analysis

Summary-data-based Mendelian Randomization (SMR) [30] employs GWAS summary data from both GWAS and pQTL studies to examine the pleiotropic associations between baseline protein expression levels and complex traits of interest. The SMR and HEIDI methods can be understood as an analysis aimed at evaluating whether the SNP’s impact on the phenotype is influenced by protein expression.

### Colocalization analysis

Colocalization analysis was performed using the coloc package [31]. Its purpose was to determine whether these features share common associated SNPs, shedding light on their biological relationships. The coloc package employed Bayesian methods to assess the support for five mutually exclusive hypotheses: (1) neither the SNP nor trait 1 or trait 2 are associated; (2) the SNP is associated with trait 1; (3) the SNP is associated with trait 2; (4) the SNP is independently associated with both trait 1 and trait 2; (5) the SNP is jointly associated with both trait 1 and trait 2. Posterior probabilities for H0, H1, H2, H3, and H4 were calculated for each test. We considered PP.H4 > 0.8 as indicative of colocalization.

### Drug target analysis

We classified the causal proteins associated with PBC and PSC into three classes of potential drug targets based on the findings from Mendelian randomization, HEIDI tests, and co-localization analysis. Subsequently, we examined the drugs and their respective mechanisms of action related to the top drug targets in the first two classes using the comprehensive DRUGBANK database [32], known for its curated information on drug properties, pharmacokinetics, and interactions.

### Differential expression analysis

We further employed GSE119600 dataset [33] from the Gene Expression Omnibus (GEO) repository to assess the differential expression of aforementioned druggable genes in individuals with PBC and PSC, as compared to healthy controls. The analysis included blood transcriptome data from 45 patients with PSC, 90 patients with PBC, and 47 individuals in a healthy control group.

### Immunohistochemistry (IHC)

A total of 310 subjects who underwent liver transplantation were recruited from Ningbo Medical Center Lihuili Hospital between January 2016 and August 2022. Among these subjects, 3 were diagnosed with PBC and 1 with PSC based on pathology. Liver tissue specimens were collected from the PBC (*n* = 3) and PSC (*n* = 1) patients, as well as normal liver tissue specimens obtained from a liver donor (*n* = 1). Immunohistochemical analysis was performed to investigate the expression of the aforementioned potential druggable genes in the liver tissues. The standardized procedures described in a previous publication [34] were followed. Specifically, frozen sections were fixed in methanol for a total of 20 minutes. After antigen retrieval and suppression of endogenous peroxidase activity, the sections were incubated at room temperature with 3% BSA (Sigma, CA, USA) for 30 minutes. Following the removal of the blocking solution, preconfigured primary antibodies were added to the sections and incubated overnight at 4 °C. Subsequently, the sections were incubated at room temperature for 50 minutes with the corresponding secondary antibodies. DAB chromogenic solution (Genetech, Shanghai, China) and hematoxylin were used for section staining, where the nucleus stained with hematoxylin appeared blue, and positive expression of DAB appeared brownish yellow. An imaging system (Leica DFC450C, Leica, Shanghai, China) was utilized for image acquisition and analysis.

### Statistical methods

All statistical analyses and data calculations were carried out using the open-source language and environment for statistical computing and graphics, R software (version 4.1.2; available at https://www.r-project.org/). The threshold for statistical significance was set at a *p*-value < 0.05.

### Availability of data and materials

The original contributions presented in the study are included in the article/supplementary material, further inquiries can be directed to the corresponding author/s. All data and original files in our work are freely available under a ‘Creative Commons BY 4.0’ license. All methods were carried out in accordance with relevant guidelines and regulations.

## RESULTS

**Figure 1:**
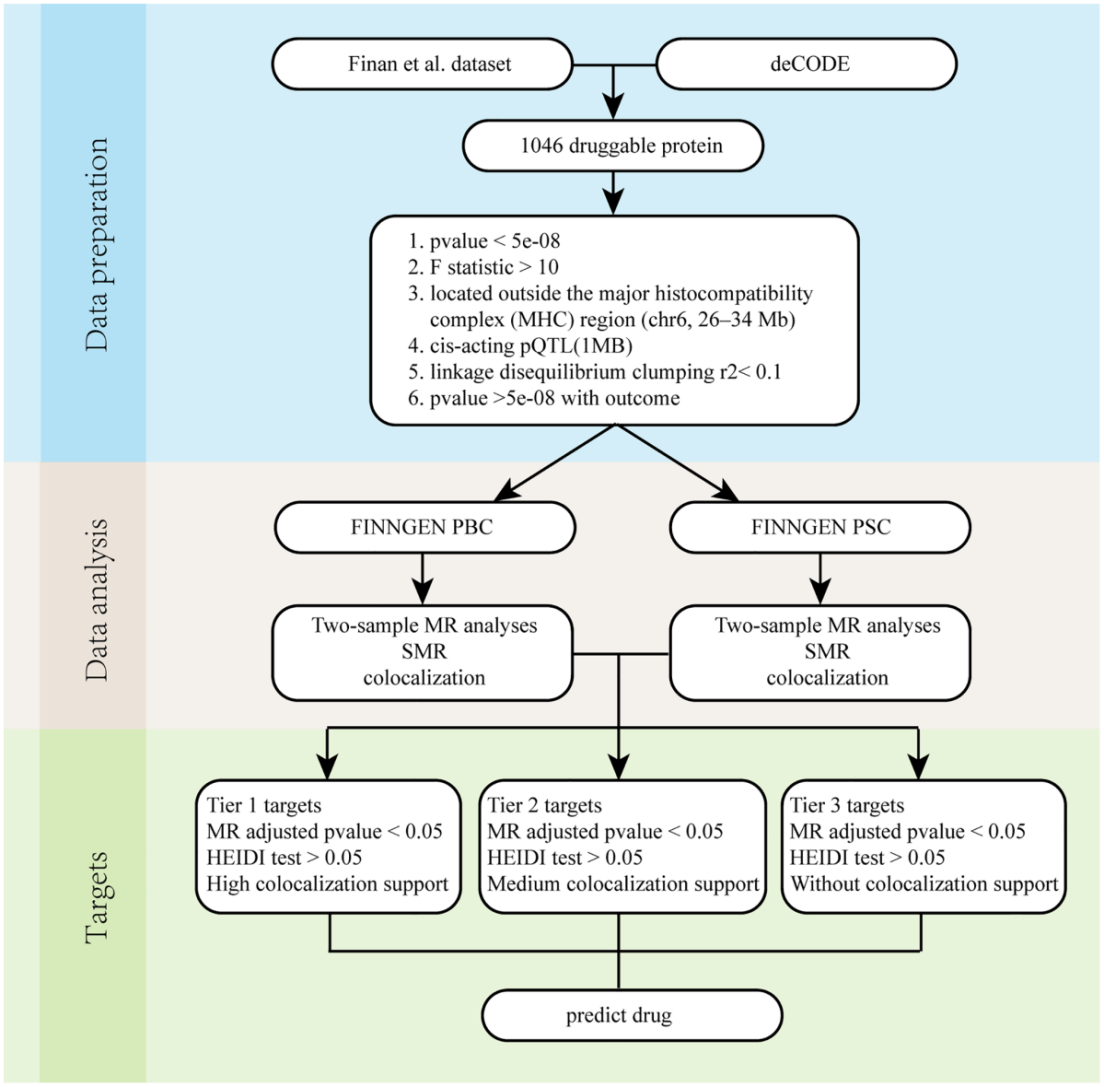
**Overall flow chart.** Abbreviations: PBC: Primary biliary cholangitis; PSC: Primary sclerosing cholangitis; SMR: Summary-data-based Mendelian randomization.

Figure 1 demonstrates the whole design and procedure of this study.

### MR analysis of druggable proteins

We selected 1046 proteins encoded by druggable genes from 4907 proteins in the deCODE database (Supplementary Table 3) and performed two-sample Mendelian randomization analysis with PBC and PSC. The *p*-values of the analysis results were adjusted using the stringent Bonferroni method (*p*-value < 0.05/1046). From the results in Table 1, it could be observed that there were 8 proteins with causal relationships with PBC. Among them, HSPB1, LEFTY2, FGF2, TNFAIP6, ITIH5, and PBC had a positive correlation, while EPHA1, ERAP1, and PBC had a negative correlation (all results were shown in Supplementary Table 4). From the results in Table 2, it could be observed that there are another 8 proteins with causal relationships with PSC. Among them, GSTO1, RNASET2, FCGR2A, FCGR2B, RNASE6, and CTSB had a negative correlation with PSC, while FCGR3A, FCGR3B, and PSC had a positive correlation (all results were shown in Supplementary Table 5).

**Table 1:** The result of Mendelian randomization between protein and primary biliary cholangitis.

**Exposure**	**Gene**	**UniProt**	**Outcome**	**Method**	**Nsnp**	**b**	**se**	***p*-val**
11103_24_HSPB1_HSP_27.txt.gz	HSPB1	P04792	PBC	IVW	55	0.485995906	0.090246638	7.24E-08
15503_15_LEFTY2_Lefty_A.txt.gz	LEFTY2	O00292	PBC	IVW	16	1.03573495	0.222256859	3.16E-06
15503_20_LEFTY2_Lefty_A.txt.gz	LEFTY2	O00292	PBC	IVW	115	0.279742942	0.045272049	6.44E-10
3025_50_FGF2_bFGF.txt.gz	FGF2	P09038	PBC	IVW	81	0.310168146	0.074496891	3.13E-05
3431_54_EPHA1_EphA1.txt.gz	EPHA1	P21709	PBC	IVW	64	−0.216252104	0.047195482	4.60E-06
4964_67_ERAP1_ARTS1.txt.gz	ERAP1	Q9NZ08	PBC	IVW	138	−0.114925345	0.027587082	3.10E-05
5036_50_TNFAIP6_TSG_6.txt.gz	TNFAIP6	P98066	PBC	IVW	79	0.258985304	0.06156953	2.59E-05
8233_2_ITIH5_ITIH5.txt.gz	ITIH5	Q86UX2	PBC	IVW	94	0.312619443	0.069467875	6.79E-06

Abbreviation: PBC: primary biliary cholangitis.

**Table 2:** The result of Mendelian randomization between protein and primary sclerosing cholangitis.

**Exposure**	**Gene**	**UniProt**	**Outcome**	**Method**	**Nsnp**	**b**	**se**	***p*-val**
12436_84_GSTO1_GST_omega_1.txt.gz	GSTO1	P78417	PSC	IVW	112	−0.155230396	0.028185457	3.64E-08
15388_24_FCGR3A_FcRIIIa.txt.gz	FCGR3A	P08637	PSC	IVW	176	0.108085588	0.019235973	1.92E-08
16913_8_RNASET2_RNT2.txt.gz	RNASET2	O00584	PSC	IVW	54	−0.225056815	0.050975082	1.01E-05
3309_2_FCGR2A_FCG2A.txt.gz	FCGR2A	P12318	PSC	IVW	158	−0.09643699	0.018820999	2.99E-07
3310_62_FCGR2B_FCG2B.txt.gz	FCGR2B	P31994	PSC	IVW	164	−0.091293154	0.019245418	2.10E-06
3311_27_FCGR3B_FCG3B.txt.gz	FCGR3B	O75015	PSC	IVW	45	0.271596135	0.064005135	2.20E-05
5646_20_RNASE6_RNAS6.txt.gz	RNASE6	Q93091	PSC	IVW	150	−0.116694162	0.025940446	6.84E-06
8007_19_CTSB_Cathepsin_B.txt.gz	CTSB	P07858	PSC	IVW	87	−0.167020198	0.032992719	4.14E-07

Abbreviation: PSC: primary sclerosing cholangitis.

### Sensitivity analysis on the proteins and PBC

**Figure 2:**
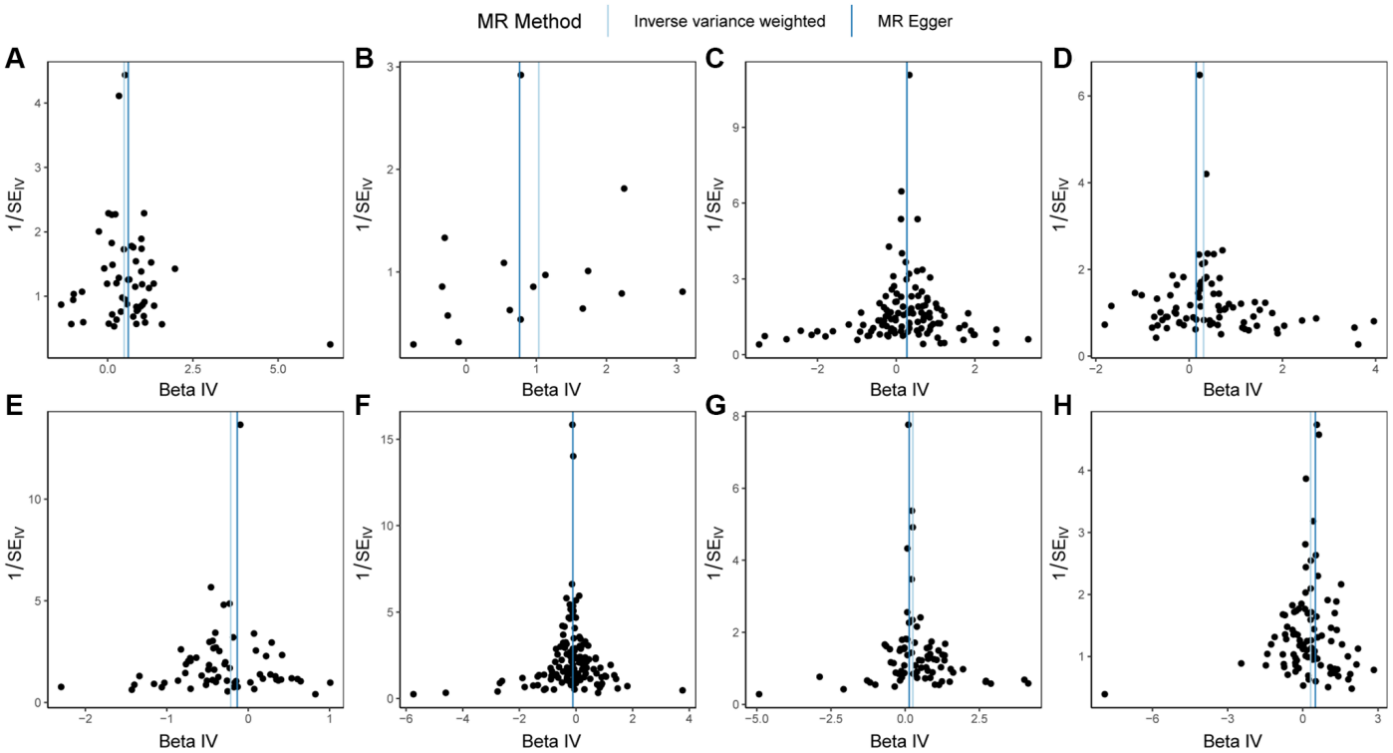
**Funnel plots of Mendelian randomization (MR) analysis on PBC.** (**A**) MR funnel plot for HSPB1 and PBC; (**B**) MR funnel plot for LEFTY2 with primer 15503_15 and PBC; (**C**) MR funnel plot for LEFTY2 with primer 15503_20 and PBC; (**D**) MR funnel plot for FGF2 and PBC; (**E**) MR funnel plot for EPHA1 and PBC; (**F**) MR funnel plot for ERAP1 and PBC; (**G**) MR funnel plot for TNFAIP6 and PBC; (**H**) MR funnel plot for ITIH5 and PBC. Abbreviations: MR: Mendelian randomization; PBC: primary biliary cholangitis.

First, we conducted heterogeneity tests on HSPB1, LEFTY2, LEFTY2, FGF2, TNFAIP6, ITIH5, EPHA1, ERAP1, and PBC (Table 3). The *q*-values of the heterogeneity tests for the 8 proteins were all greater than 0.05, regardless of whether the MR Egger method or IVW method was used. This indicates that there was no significant heterogeneity among the instrumental variables we used, providing reliable results. The funnel plot in Figure 2 demonstrated an even distribution of instrumental variables for the 8 proteins without obvious heterogeneity, consistent with the calculation results.

**Table 3:** The result of heterogeneity test between protein and primary biliary cholangitis.

**Exposure**	**Gene**	**UniProt**	**Outcome**	**Method**	**Q**	**Q_df**	**Q_*p*-val**
11103_24_HSPB1_HSP_27.txt.gz	HSPB1	P04792	PBC	MR Egger	33.57942018	53	0.982844971
11103_24_HSPB1_HSP_27.txt.gz	HSPB1	P04792	PBC	IVW	34.28677871	54	0.983326124
15503_15_LEFTY2_Lefty_A.txt.gz	LEFTY2	O00292	PBC	MR Egger	15.34154428	14	0.355212901
15503_15_LEFTY2_Lefty_A.txt.gz	LEFTY2	O00292	PBC	IVW	15.63659144	15	0.406606267
15503_20_LEFTY2_Lefty_A.txt.gz	LEFTY2	O00292	PBC	MR Egger	127.1386199	113	0.171611091
15503_20_LEFTY2_Lefty_A.txt.gz	LEFTY2	O00292	PBC	IVW	127.1858557	114	0.187957209
3025_50_FGF2_bFGF.txt.gz	FGF2	P09038	PBC	MR Egger	67.26242789	79	0.824010298
3025_50_FGF2_bFGF.txt.gz	FGF2	P09038	PBC	IVW	69.3766221	80	0.795841665
3431_54_EPHA1_EphA1.txt.gz	EPHA1	P21709	PBC	MR Egger	36.30233909	62	0.996253655
3431_54_EPHA1_EphA1.txt.gz	EPHA1	P21709	PBC	IVW	38.80564268	63	0.992956538
4964_67_ERAP1_ARTS1.txt.gz	ERAP1	Q9NZ08	PBC	MR Egger	124.9118521	136	0.742409772
4964_67_ERAP1_ARTS1.txt.gz	ERAP1	Q9NZ08	PBC	IVW	124.9186857	137	0.761735543
5036_50_TNFAIP6_TSG_6.txt.gz	TNFAIP6	P98066	PBC	MR Egger	67.40211869	77	0.774509578
5036_50_TNFAIP6_TSG_6.txt.gz	TNFAIP6	P98066	PBC	IVW	69.59190645	78	0.740574298
8233_2_ITIH5_ITIH5.txt.gz	ITIH5	Q86UX2	PBC	MR Egger	101.1836469	92	0.24055945
8233_2_ITIH5_ITIH5.txt.gz	ITIH5	Q86UX2	PBC	IVW	104.0331413	93	0.203980833

Abbreviation: PBC: primary biliary cholangitis.

Next, a pleiotropy test was performed on HSPB1, LEFTY2, LEFTY2, FGF2, TNFAIP6, ITIH5, EPHA1, ERAP1, and PBC (Table 4). The *q*-values of the pleiotropy tests for the 8 proteins were all greater than 0.05, suggesting that the selected SNPs do not act on PBC through other pathways.

**Table 4:** The result of pleiotropy test between protein and primary biliary cholangitis.

**Exposure**	**Gene**	**UniProt**	**Outcome**	**Egger_intercept**	**se**	***p*-val**
11103_24_HSPB1_HSP_27.txt.gz	HSPB1	P04792	PBC	−0.022169912	0.026359926	0.404100774
15503_15_LEFTY2_Lefty_A.txt.gz	LEFTY2	O00292	PBC	0.036260473	0.069880865	0.611942028
15503_20_LEFTY2_Lefty_A.txt.gz	LEFTY2	O00292	PBC	0.003522901	0.017193505	0.83802105
3025_50_FGF2_bFGF.txt.gz	FGF2	P09038	PBC	0.035640536	0.024511607	0.149900303
3431_54_EPHA1_EphA1.txt.gz	EPHA1	P21709	PBC	−0.028371055	0.017931587	0.118696115
4964_67_ERAP1_ARTS1.txt.gz	ERAP1	Q9NZ08	PBC	−0.001297117	0.015691191	0.934239228
5036_50_TNFAIP6_TSG_6.txt.gz	TNFAIP6	P98066	PBC	0.032322808	0.021842788	0.14300839
8233_2_ITIH5_ITIH5.txt.gz	ITIH5	Q86UX2	PBC	−0.034397758	0.021370146	0.110907565

Abbreviation: PBC: primary biliary cholangitis.

Furthermore, leave-one-out tests were conducted on the SNPs of the 8 proteins. Supplementary Figure 1 showed that the beta values of all the results lay on one side of 0, indicating that individual SNPs had minimal impact on the results of Mendelian randomization, and the results remained stable. Additional analysis using the Steiger direction test revealed that, except for the direction between the protein encoded by ERAP1 and PBC being uncertain, the *p*-values of the Steiger direction tests for HSPB1, LEFTY2, LEFTY2, FGF2, TNFAIP6, ITIH5, EPHA1, and PBC were all much smaller than 0.05. This indicated that the direction was correct and that the protein products of these 7 genes potentially contributed to the occurrence of PBC (Table 5). To further verify the direction between the protein encoded by ERAP1 and PBC, a reverse Mendelian randomization analysis was conducted, resulting in a *p*-value of 0.18, indicating no reverse causal relationship existed (Supplementary Table 6).

**Table 5:** The result of Steiger direction test between protein and primary biliary cholangitis.

**Exposure**	**Gene**	**UniProt**	**Outcome**	**Snp_r2. exposure**	**Snp_r2. outcome**	**Correct_causal_direction**	**Steiger_*p*-val**
11103_24_HSPB1_HSP_27.txt.gz	HSPB1	P04792	PBC	0.179613954	0.000256323	TRUE	0
15503_15_LEFTY2_Lefty_A.txt.gz	LEFTY2	O00292	PBC	0.037895293	0.000135877	TRUE	4.08E-237
15503_20_LEFTY2_Lefty_A.txt.gz	LEFTY2	O00292	PBC	0.545890549	0.000617874	TRUE	0
3025_50_FGF2_bFGF.txt.gz	FGF2	P09038	PBC	0.30229971	0.000317004	TRUE	0
3431_54_EPHA1_EphA1.txt.gz	EPHA1	P21709	PBC	0.318174206	0.000212982	TRUE	0
4964_67_ERAP1_ARTS1.txt.gz	ERAP1	Q9NZ08	PBC	1.036182549	0.000535196	TRUE	NA
5036_50_TNFAIP6_TSG_6.txt.gz	TNFAIP6	P98066	PBC	0.290984971	0.000323357	TRUE	0
8233_2_ITIH5_ITIH5.txt.gz	ITIH5	Q86UX2	PBC	0.328213373	0.000496922	TRUE	0

Abbreviation: PBC: primary biliary cholangitis.

**Figure 3:**
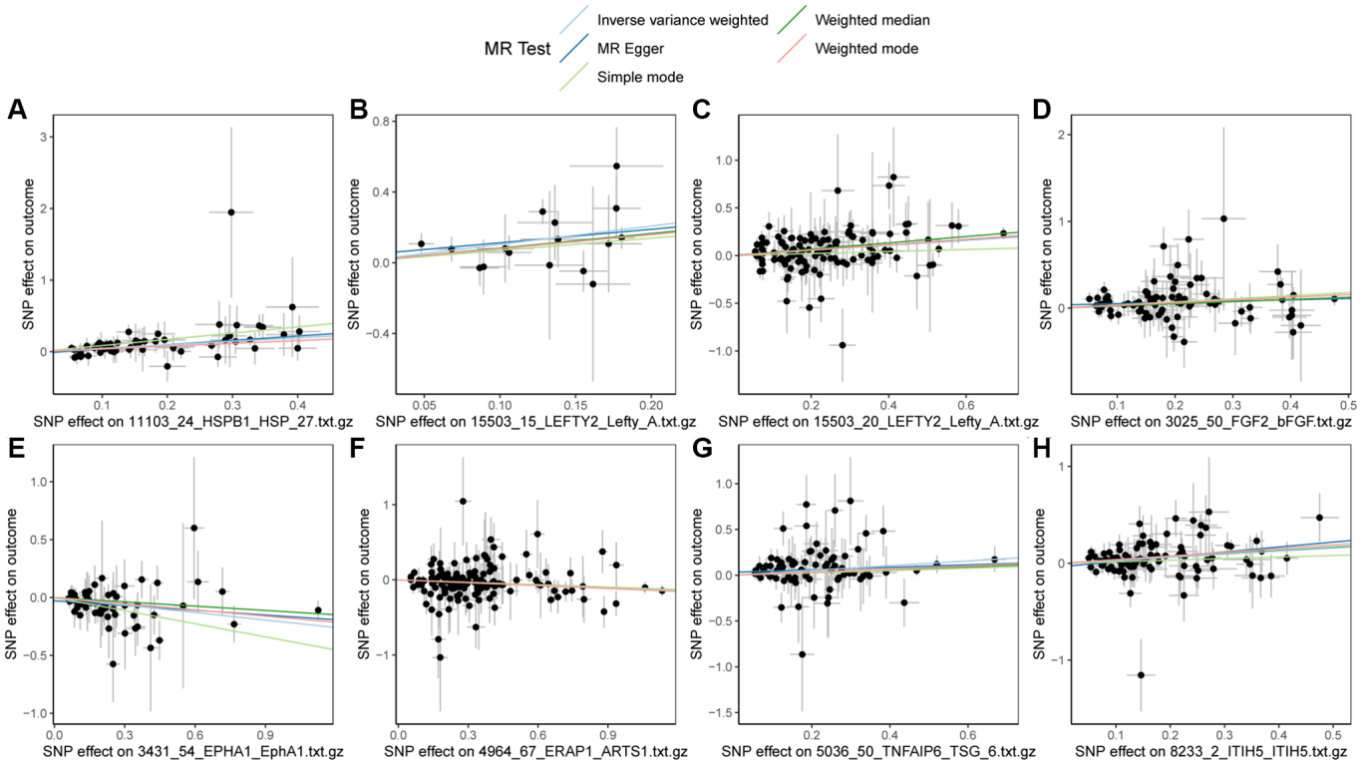
**Scatter plots of Mendelian randomization (MR) results on PBC.** (**A**) Scatter plot of MR results for HSPB1 and PBC; (**B**) Scatter plot of MR results for LEFTY2 with primer 15503_15 and PBC; (**C**) Scatter plot of MR results for LEFTY2 with primer 15503_20 and PBC; (**D**) Scatter plot of MR results for FGF2 and PBC; (**E**) Scatter plot of MR results for EPHA1 and PBC; (**F**) Scatter plot of MR results for ERAP1 and PBC; (**G**) Scatter plot of MR results for TNFAIP6 and PBC; (**H**) Scatter plot of MR results for ITIH5 and PBC. Abbreviations: MR: Mendelian randomization; PBC: primary biliary cholangitis.

Finally, the scatter plot of the Mendelian randomization results for HSPB1, LEFTY2, LEFTY2, FGF2, TNFAIP6, ITIH5, EPHA1, ERAP1, and PBC was plotted. As shown in Figure 3, HSPB1, LEFTY2, FGF2, TNFAIP6, ITIH5, and PBC exhibited positive correlation, while EPHA1, ERAP1, and PBC showed negative correlation, consistent with the results of the Mendelian randomization.

### Sensitivity analysis on the proteins and PSC

**Figure 4:**
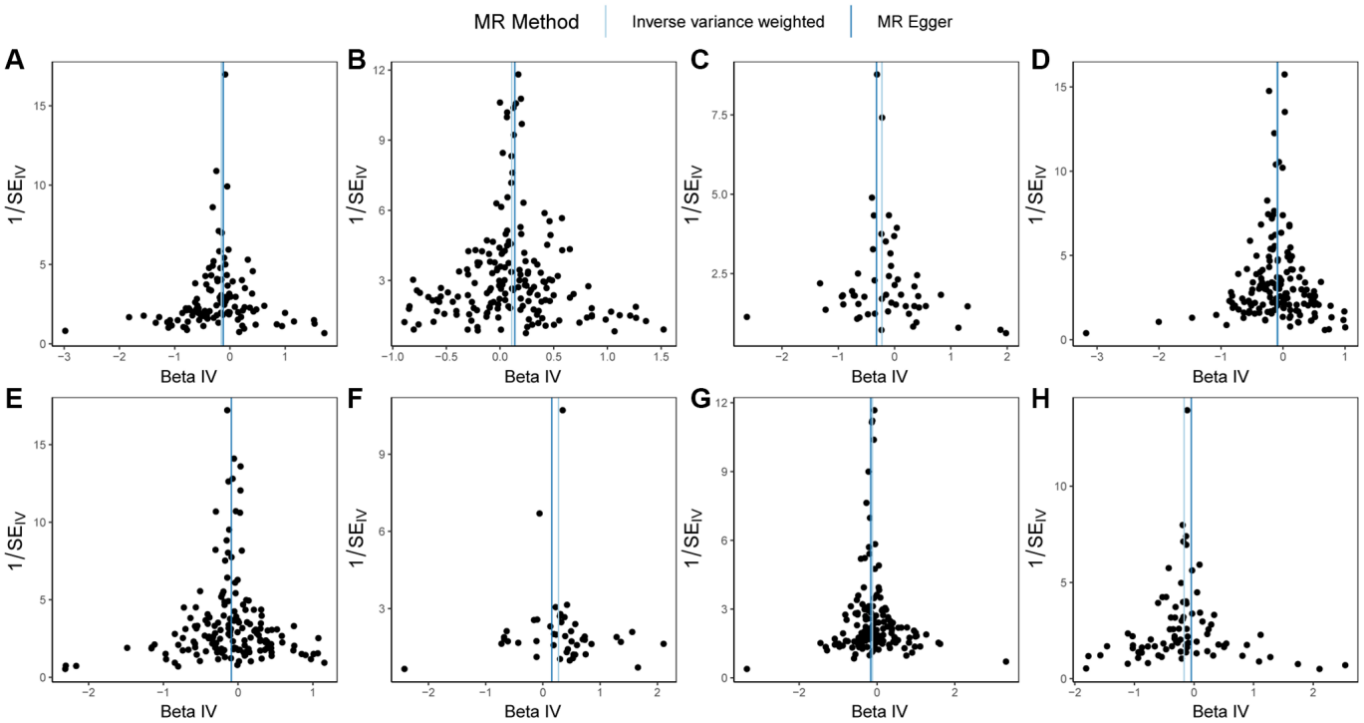
**Funnel plots of Mendelian randomization (MR) analysis on PSC.** (**A**) MR funnel plot for GSTO1 and PSC; (**B**) MR funnel plot for FCGR3A and PSC; (**C**) MR funnel plot for RNASET2 and PSC; (**D**) MR funnel plot for FCGR2A and PSC; (**E**) MR funnel plot for FCGR2B and PSC; (**F**) MR funnel plot for FCGR3B and PSC; (**G**) MR funnel plot for RNASE6 and PSC; (**H**) MR funnel plot for CTSB and PSC. Abbreviations: MR: Mendelian randomization; PSC: primary sclerosing cholangitis.

Firstly, heterogeneity tests were conducted on GSTO1, FCGR3A, RNASET2, FCGR2A, FCGR2B, FCGR3B, RNASE6, CTSB, and PSC (Table 6). The results from both the MR Egger method and the IVW method indicated that all genes, except FCGR2B, demonstrated q-values greater than 0.05. This implies that there was no significant heterogeneity among the instrumental variables of GSTO1, FCGR3A, RNASET2, FCGR2A, FCGR3B, RNASE6, and CTSB, ensuring the reliability of the outcomes. The uniform distribution of instrumental variables without significant heterogeneity can be observed in the funnel plot displayed in Figure 4. Therefore, for the Mendelian randomization analysis between FCGR2B and PSC, we rely on the random-effects model results obtained using the Inverse Variance Weighted method, which revealed a *p*-value of 2.10e-06, suggesting a causal relationship between the two.

**Table 6:** The result of heterogeneity test between protein and primary sclerosing cholangitis.

**Exposure**	**Gene**	**UniProt**	**Outcome**	**Method**	**Q**	**Q_df**	**Q_*p*-val**
12436_84_GSTO1_GST_omega_1.txt.gz	GSTO1	P78417	PSC	MR Egger	130.5352222	110	0.088355553
12436_84_GSTO1_GST_omega_1.txt.gz	GSTO1	P78417	PSC	IVW	131.409545	111	0.090455909
15388_24_FCGR3A_FcRIIIa.txt.gz	FCGR3A	P08637	PSC	MR Egger	164.2225705	174	0.690884965
15388_24_FCGR3A_FcRIIIa.txt.gz	FCGR3A	P08637	PSC	IVW	165.1510566	175	0.691760694
16913_8_RNASET2_RNT2.txt.gz	RNASET2	O00584	PSC	MR Egger	51.66881974	52	0.486868401
16913_8_RNASET2_RNT2.txt.gz	RNASET2	O00584	PSC	IVW	53.89309321	53	0.439994777
3309_2_FCGR2A_FCG2A.txt.gz	FCGR2A	P12318	PSC	MR Egger	161.4126697	156	0.366700706
3309_2_FCGR2A_FCG2A.txt.gz	FCGR2A	P12318	PSC	IVW	161.6123403	157	0.383872503
3310_62_FCGR2B_FCG2B.txt.gz	FCGR2B	P31994	PSC	MR Egger	195.8890975	162	0.035781113
3310_62_FCGR2B_FCG2B.txt.gz	FCGR2B	P31994	PSC	IVW	195.8895765	163	0.040224913
3311_27_FCGR3B_FCG3B.txt.gz	FCGR3B	O75015	PSC	MR Egger	55.14172947	43	0.101425523
3311_27_FCGR3B_FCG3B.txt.gz	FCGR3B	O75015	PSC	IVW	57.33132758	44	0.085637624
5646_20_RNASE6_RNAS6.txt.gz	RNASE6	Q93091	PSC	MR Egger	148.4066695	148	0.475134707
5646_20_RNASE6_RNAS6.txt.gz	RNASE6	Q93091	PSC	IVW	150.3312716	149	0.454027288
8007_19_CTSB_Cathepsin_B.txt.gz	CTSB	P07858	PSC	MR Egger	82.79503252	85	0.547491994
8007_19_CTSB_Cathepsin_B.txt.gz	CTSB	P07858	PSC	IVW	89.71617707	86	0.370679142

Abbreviation: PSC: primary sclerosing cholangitis.

Next, a pleiotropy test was conducted on GSTO1, FCGR3A, RNASET2, FCGR2A, FCGR2B, FCGR3B, RNASE6, CTSB, and PSC (Table 7). It was noticeable that all genes, except CTSB, exhibited q-values greater than 0.05 in the pleiotropy tests, indicating that the selected SNPs do not exert their effects on PSC through other pathways. However, CTSB’s SNP demonstrates horizontal pleiotropy, rendering the results unreliable, and consequently it is excluded from subsequent analyses.

**Table 7:** The result of pleiotropy test between protein and primary sclerosing cholangitis.

**Exposure**	**Gene**	**UniProt**	**Outcome**	**Egger_intercept**	**se**	***p*-val**
12436_84_GSTO1_GST_omega_1.txt.gz	GSTO1	P78417	PSC	−0.010567731	0.012311568	0.392561392
15388_24_FCGR3A_FcRIIIa.txt.gz	FCGR3A	P08637	PSC	−0.008928035	0.009265486	0.336593845
16913_8_RNASET2_RNT2.txt.gz	RNASET2	O00584	PSC	0.019043534	0.012768899	0.141898527
3309_2_FCGR2A_FCG2A.txt.gz	FCGR2A	P12318	PSC	−0.004008726	0.009125482	0.661059868
3310_62_FCGR2B_FCG2B.txt.gz	FCGR2B	P31994	PSC	−0.000175424	0.008813695	0.98414481
3311_27_FCGR3B_FCG3B.txt.gz	FCGR3B	O75015	PSC	0.023774026	0.018193929	0.198259968
5646_20_RNASE6_RNAS6.txt.gz	RNASE6	Q93091	PSC	0.010788007	0.007786936	0.168015118
8007_19_CTSB_Cathepsin_B.txt.gz	CTSB	P07858	PSC	−0.028469396	0.010821546	0.01011224

Abbreviation: PSC: primary sclerosing cholangitis.

Furthermore, leave-one-out tests were performed on the SNPs of the 8 proteins, as depicted in Supplementary Figure 2. The outcomes revealed that the beta values for individual SNPs in the MR analysis lay on one side of zero, suggesting that individual SNPs had negligible impact on the analysis results and ensuring their stability. Steiger direction tests were additionally conducted, indicating that the *p*-values for the Steiger direction tests between these 8 proteins and PSC are significantly less than 0.05, implying the correct directionality, namely, the protein products of these 8 genes influence the occurrence of PSC (Table 8).

**Table 8:** The result of Steiger direction test between protein and primary sclerosing cholangitis.

**Exposure**	**Gene**	**UniProt**	**Outcome**	**Snp_r2. exposure**	**Snp_r2. outcome**	**Correct_causal_direction**	**Steiger_*p*-val**
12436_84_GSTO1_GST_omega_1.txt.gz	GSTO1	P78417	PSC	0.592299575	0.000537659	TRUE	0
15388_24_FCGR3A_FcRIIIa.txt.gz	FCGR3A	P08637	PSC	0.934075888	0.000699525	TRUE	0
16913_8_RNASET2_RNT2.txt.gz	RNASET2	O00584	PSC	0.178739732	0.000236886	TRUE	0
3309_2_FCGR2A_FCG2A.txt.gz	FCGR2A	P12318	PSC	0.855566158	0.000697265	TRUE	0
3310_62_FCGR2B_FCG2B.txt.gz	FCGR2B	P31994	PSC	0.77594143	0.000765493	TRUE	0
3311_27_FCGR3B_FCG3B.txt.gz	FCGR3B	O75015	PSC	0.122381344	0.000278588	TRUE	0
5646_20_RNASE6_RNAS6.txt.gz	RNASE6	Q93091	PSC	0.505588478	0.000564529	TRUE	0
8007_19_CTSB_Cathepsin_B.txt.gz	CTSB	P07858	PSC	0.500938131	0.000420383	TRUE	0

Abbreviation: PSC: primary sclerosing cholangitis.

**Figure 5:**
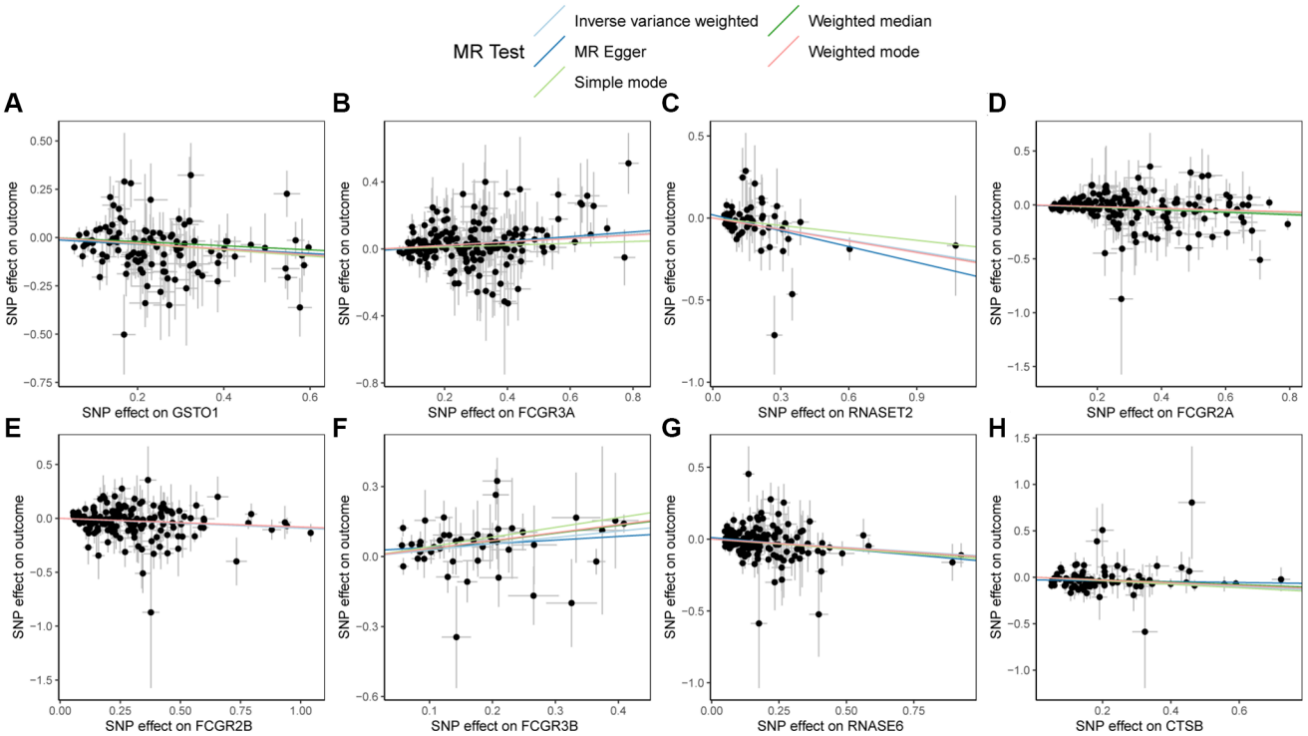
**Scatter plots of Mendelian randomization (MR) results on PSC.** (**A**) Scatter plot of MR results for GSTO1 and PSC; (**B**) Scatter plot of MR results for FCGR3A and PSC; (**C**) Scatter plot of MR results for RNASET2 and PSC; (**D**) Scatter plot of MR results for FCGR2A and PSC; (**E**) Scatter plot of MR results for FCGR2B and PSC; (**F**) Scatter plot of MR results for FCGR3B and PSC; (**G**) Scatter plot of MR results for RNASE6 and PSC; (**H**) Scatter plot of MR results for CTSB and PSC. Abbreviations: MR: Mendelian randomization; PSC: primary sclerosing cholangitis.

Lastly, scatter plots illustrating the MR results for GSTO1, FCGR3A, RNASET2, FCGR2A, FCGR2B, FCGR3B, RNASE6, CTSB, and PSC were generated in Figure 5. From the figure, a positive correlation among FCGR3A, FCGR3B, and PSC could be observed, while GSTO1, RNASET2, FCGR2A, FCGR2B, RNASE6, CTSB, and PSC exhibited negative correlations, consistent with the MR results.

### SMR and colocalization analysis

The presence of pleiotropy was further confirmed through the HEIDI test in the SMR analysis. The results showed that except for the protein encoded by LEFTY2 with primer 15503_15 and the protein encoded by FCGR2A, all other HEIDI test *p*-values were greater than 0.05 (Table 9). Therefore, the SNPs of the protein encoded by LEFTY2 with primer 15503_15 and the protein encoded by FCGR2A demonstrated pleiotropy.

**Table 9:** The result of summary-data-based Mendelian randomization.

**Exposure**	**Gene**	**Topsnp**	**Outcome**	**p_HEIDI**	**nsnp_HEIDI**
15503_15_LEFTY2_Lefty_A.txt.gz	LEFTY2	rs360058	PBC	0.01569885	20
15503_20_LEFTY2_Lefty_A.txt.gz	LEFTY2	rs10915893	PBC	0.0537142	20
5036_50_TNFAIP6_TSG_6.txt.gz	TNFAIP6	rs2278089	PBC	0.890924	20
3025_50_FGF2_bFGF.txt.gz	FGF2	rs308403	PBC	0.9544156	20
4964_67_ERAP1_ARTS1.txt.gz	ERAP1	rs30185	PBC	0.9706626	20
11103_24_HSPB1_HSP_27.txt.gz	HSPB1	rs2868371	PBC	0.8863009	20
3431_54_EPHA1_EphA1.txt.gz	EPHA1	rs4725617	PBC	0.1184055	20
8233_2_ITIH5_ITIH5.txt.gz	ITIH5	rs10795561	PBC	0.1364776	20
3309_2_FCGR2A_FCG2A.txt.gz	FCGR2A	rs1801274	PSC	0.01835392	20
15388_24_FCGR3A_FcRIIIa.txt.gz	FCGR3A	rs10919543	PSC	0.2464716	20
3311_27_FCGR3B_FCG3B.txt.gz	FCGR3B	rs2099684	PSC	0.1935976	20
3310_62_FCGR2B_FCG2B.txt.gz	FCGR2B	rs6665610	PSC	0.1977813	20
16913_8_RNASET2_RNT2.txt.gz	RNASET2	rs35614625	PSC	0.2032446	20
8007_19_CTSB_Cathepsin_B.txt.gz	CTSB	rs2645425	PSC	0.8469198	20
12436_84_GSTO1_GST_omega_1.txt.gz	GSTO1	rs45596840	PSC	0.6104043	20
5646_20_RNASE6_RNAS6.txt.gz	RNASE6	rs986193	PSC	0.2111333	20

Abbreviations: PSC: primary sclerosing cholangitis; PBC: primary biliary cholangitis.

The coloc analysis results (Table 10) indicated a strong colocalization between the protein encoded by LEFTY2 with primer 15503_20 and PBC, and between FCGR3B and PSC (PP.H4 > 0.8). There was a moderate colocalization between HSPB1 and PBC, and between FCGR2A and PSC (0.5 < PP.H4 < 0.8).

**Table 10:** The result of colocalization.

**Exposure**	**Gene**	**UniProt**	**Outcome**	**Nsnps**	**PP.H0.abf**	**PP.H1.abf**	**PP.H2.abf**	**PP.H3.abf**	**PP.H4.abf**
11103_24_HSPB1_HSP_27.txt.gz	HSPB1	P04792	PBC	7604	1.02E-293	0.244948657	6.43E-294	0.154662126	0.600389217
15503_15_LEFTY2_Lefty_A.txt.gz	LEFTY2	O00292	PBC	7702	9.72E-96	0.359608508	1.37E-95	0.505349337	0.135042155
15503_20_LEFTY2_Lefty_A.txt.gz	LEFTY2	O00292	PBC	7702	9.30E-303	0.044440725	1.31E-302	0.06189221	0.893667065
3025_50_FGF2_bFGF.txt.gz	FGF2	P09038	PBC	8401	3.86E-302	0.612355464	2.12E-302	0.336159162	0.051485374
3431_54_EPHA1_EphA1.txt.gz	EPHA1	P21709	PBC	5680	2.38E-295	0.617372726	1.03E-295	0.266127574	0.1164997
4964_67_ERAP1_ARTS1.txt.gz	ERAP1	Q9NZ08	PBC	9300	4.37E-306	0.589286656	2.39E-306	0.321659709	0.089053635
5036_50_TNFAIP6_TSG_6.txt.gz	TNFAIP6	P98066	PBC	8110	1.25E-303	0.630683591	6.43E-304	0.325367544	0.043948865
8233_2_ITIH5_ITIH5.txt.gz	ITIH5	Q86UX2	PBC	11520	4.55E-294	0.473506481	3.39E-294	0.352917859	0.17357566
12436_84_GSTO1_GST_omega_1.txt.gz	GSTO1	P78417	PSC	7830	6.39E-305	0.580905661	3.24E-305	0.294613073	0.124481266
15388_24_FCGR3A_FcRIIIa.txt.gz	FCGR3A	P08637	PSC	8778	7.19E-306	0.270997462	6.89E-306	0.259484414	0.469518124
16913_8_RNASET2_RNT2.txt.gz	RNASET2	O00584	PSC	10388	3.76E-216	0.415100579	1.93E-216	0.213417558	0.371481863
3309_2_FCGR2A_FCG2A.txt.gz	FCGR2A	P12318	PSC	8928	1.48E-306	0.240712985	1.41E-306	0.230091232	0.529195782
3310_62_FCGR2B_FCG2B.txt.gz	FCGR2B	P31994	PSC	8634	2.06E-306	0.296974467	1.96E-306	0.282948095	0.420077438
3311_27_FCGR3B_FCG3B.txt.gz	FCGR3B	O75015	PSC	8619	4.07E-304	0.051060059	3.91E-304	0.04855231	0.900387631
5646_20_RNASE6_RNAS6.txt.gz	RNASE6	Q93091	PSC	10149	2.00E-305	0.518012159	1.09E-305	0.282150919	0.199836922
8007_19_CTSB_Cathepsin_B.txt.gz	CTSB	P07858	PSC	9602	5.30E-302	0.586984533	2.95E-302	0.326537269	0.086478198

Abbreviations: PSC: primary sclerosing cholangitis; PBC: primary biliary cholangitis.

### Validation of external datasets

For further verification of the results, we validated using pQTL data from “Phenome-wide Mendelian randomization: mapping the influence of the plasma proteome on complex diseases”. The protein dataset in this data differs from the deCODE dataset. We used this new protein data to validate five genes (ITIH5, HSPB1, FGF2, TNFAIP6, ERAP1) encoding proteins in PBC, where HSPB1 had a *p*-value of < 0.05. Furthermore, in PSC, we validated five genes (FCGR3B, FCGR2B, GSTO1, CTSB, RNASE6) encoding proteins, where FCGR3B, FCGR2B, and RNASE6 had a *p*-value of < 0.05 (Table 11). Nearly half of the genes encoding proteins were able to reproduce positive results. However, due to the smaller number of SNPs and sample size in the validation dataset compared to the deCODE dataset, we relied on the data from the deCODE database.

**Table 11:** The result of Mendelian randomization between protein and primary cholangitis (validation).

**Gene**	**UniProt**	**Outcome**	**Method**	**Nsnp**	**b**	**se**	***p*-val**
ITIH5	G5E9D8; Q86UX2; Q96JW9; C9J2H1; A0A096LP62	PBC	Wald ratio	1	0.281800435	0.21906797	0.198317196
HSPB1	P04792; V9HW43	PBC	Wald ratio	1	−0.465030556	0.204754444	0.02313716
FGF2	P09038	PBC	Wald ratio	1	0.229518318	0.120573948	0.056969056
TNFAIP6	P98066	PBC	Wald ratio	1	0.170681511	0.113453002	0.132472021
ERAP1	Q9NZ08	PBC	Wald ratio	1	−0.112176807	0.078958294	0.155401199
FCGR3B	O75015; M9MML6; A0A087WZR4; A0A087WU90	PSC	Wald ratio	1	0.323937514	0.087694693	0.00022082
FCGR2B	P31994; P31995	PSC	Wald ratio	1	−0.074429666	0.03395003	0.028355693
GSTO1	P78417; V9HWG9	PSC	Wald ratio	1	−0.062942688	0.039904699	0.114720716
CTSB	Q5HYG5; A0A024R374; P07858; B4DMY4	PSC	Wald ratio	1	−0.143471974	0.101695949	0.158305542
RNASE6	Q6IB39; Q93091	PSC	Wald ratio	1	−0.08948982	0.039917461	0.024969673

Abbreviations: PSC: primary sclerosing cholangitis; PBC: primary biliary cholangitis.

### Pharmaceutical targets prediction

To identify potential drug targets, we categorized potential target proteins into three groups based on the results of our analysis. Group one included proteins with a *p*-value < 0.05 after Mendelian randomization correction, a HEIDI test *p*-value > 0.05, and a posterior probability of H4 (PP.H4) for colocalization analysis of ≥ 0.8. The second group consisted of proteins with a *p*-value < 0.05 after Mendelian randomization correction, a HEIDI test *p*-value > 0.05, and a PP.H4 for colocalization analysis between 0.5 and < 0.8. The third group included proteins with a *p*-value < 0.05 after Mendelian randomization correction, a HEIDI test *p*-value > 0.05, and a PP.H4 for colocalization analysis < 0.5. Using these criteria, we identified LEFTY2-encoded protein (targeted by primer 15503_20) as a potential drug target in group one, HSPB1 as a potential drug target in group two, and LEFTY2-encoded protein (targeted by primer 15503_15), TNFAIP6, FGF2, ERAP1, EPHA1, and ITIH5 as potential drug targets in group three for PBC. For PSC, we identified FCGR3B as a potential drug target in group one, and FCGR3A, FCGR2B, RNASET2, CTSB, GSTO1, and RNASE6 as potential drug targets in group three. FCGR2A was excluded from the analysis due to a HEIDI test *p*-value < 0.05, while CTSB was not considered for further analysis due to its SNP’s horizontal pleiotropy.

We analyzed the potential pharmaceuticals for group one and two druggable targets in DURGBANK database and found that LEFTY2 did not have corresponding drugs in PBC, while HSPB1 had three potential drugs: Apatorsen, Phenethyl Isothiocyanate, and Artenimol. In PSC, FCGR3B corresponded to 12 potential drugs: Alefacept, Alemtuzumab, Catumaxomab, Cetuximab, Daclizumab, Etanercept, Gemtuzumab ozogamicin, Human immunoglobulin G, Muromonab, Natalizumab, Palivizumab, and Sarilumab (Table 12).

**Table 12:** Protein corresponding drug in DRUGBANK.

**Disease**	**Tier**	**Target**	**Drugbank id**	**Name**	**Durg group**	**Pharmacological action**	**Actions**
PBC	1	LEFTY2	NA				
	2	HSPB1	DB06094	Apatorsen	Investigational	Unknown	
		DB12695	Phenethyl Isothiocyanate	Investigational	Unknown	
		DB11638	Artenimol	Approved, experimental, investigational	Unknown	Ligand
PSC	1	FCGR3B	DB00092	Alefacept	Approved, investigational, withdrawn	Unknown	
			DB00002	Cetuximab	Approved	Unknown	Binder
			DB00005	Etanercept	Approved, investigational	Unknown	Ligand
			DB00028	Human immunoglobulin G	Approved, investigational	Yes	Antagonist
			DB00056	Gemtuzumab ozogamicin	Approved, investigational	Unknown	
			DB00075	Muromonab	Approved, investigational	Unknown	
			DB00087	Alemtuzumab	Approved, investigational	Unknown	Binder
			DB00108	Natalizumab	Approved, investigational	Unknown	
			DB00110	Palivizumab	Approved, investigational	Unknown	
			DB00111	Daclizumab	Investigational, withdrawn	Unknown	
			DB11767	Sarilumab	Approved, investigational	Unknown	Unknown
			DB06607	Catumaxomab	Approved, investigational, withdrawn		

Abbreviations: PSC: primary sclerosing cholangitis; PBC: primary biliary cholangitis.

### Differential expression verification

**Figure 6:**
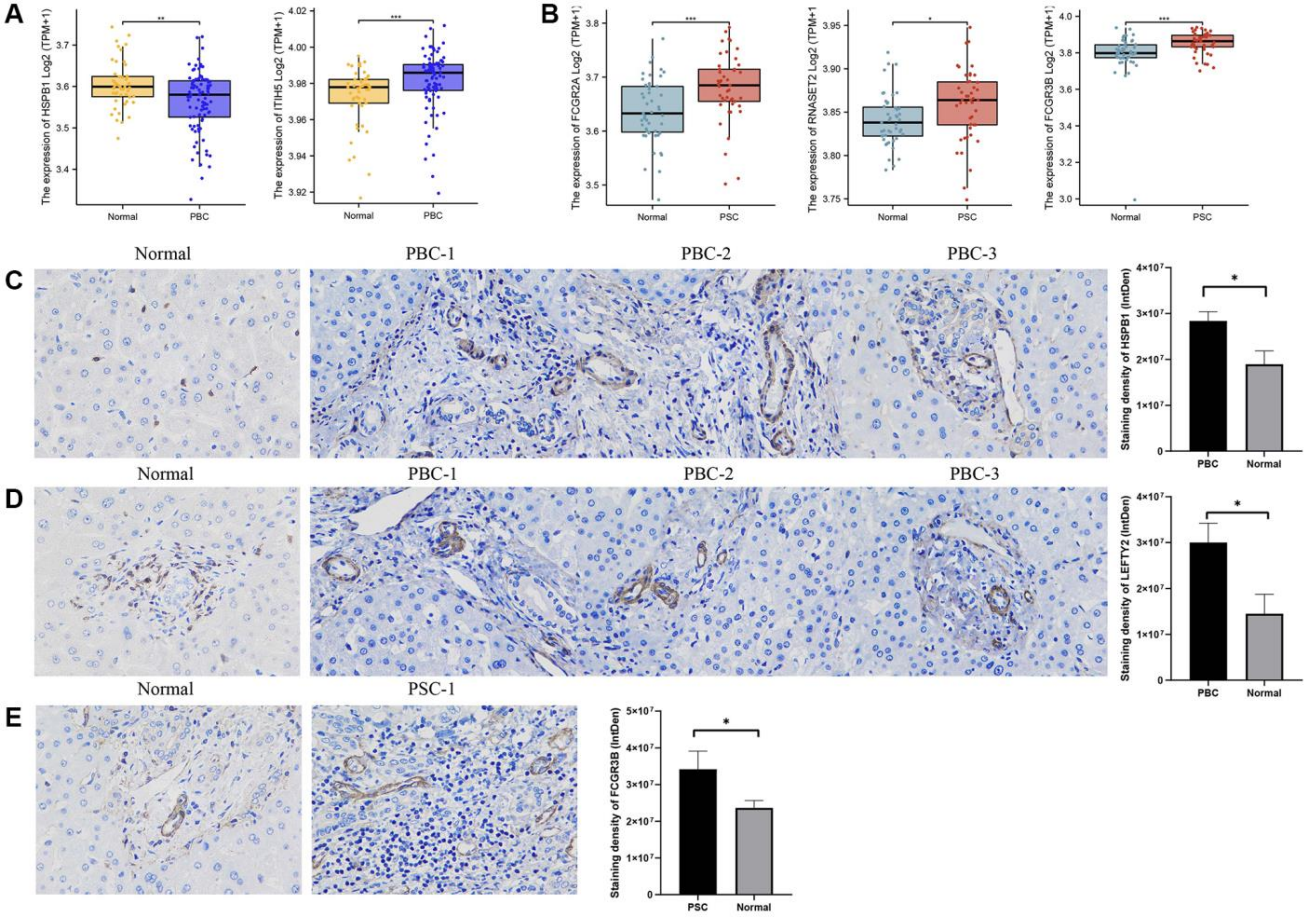
**Validation of external dataset and IHC experiment.** (**A**) Expression levels of HSPB1 and ITIH5 between PBC and control groups in GSE119600 dataset; (**B**) Expression levels of FCGR2A, FCGR3B, and RNASET2 between PSC and control groups in GSE119600 dataset; (**C**) IHC staining of HSPB1 encoding proteins in liver samples of PBC patients and normal individuals; (**D**) IHC staining of LEFTY2 encoding proteins in liver samples of PBC patients and normal individuals (**E**) IHC staining of FCGR3B encoding proteins in liver samples of PSC patients and normal individuals. Abbreviations: IHC: immunohistochemistry; PBC: primary biliary cholangitis; PSC: primary sclerosing cholangitis. ^*^p-value < 0.05, ^**^p-value < 0.01, ^***^p-value < 0.001.

Following the data normalization procedure, Top DEGs in PBC and PSC, respectively, were selected using a threshold of |log2 fold change (FC)| ≥ 1 and a *p*-value < 0.05. By intersecting with the genes identified in previous MR analysis, we found two differentially expressed genes with drug targets associated with PBC and three differentially expressed genes with drug targets associated with PSC. The expression levels of ITIH5 (*p*-value < 0.001) were increased and HSPB1 (*p*-value < 0.01) were decreased in the PBC group compared to the control group (Figure 6A). Regarding PSC, all of the expression levels of FCGR2A (*p*-value < 0.001), FCGR3B (*p*-value < 0.001), and RNASET2 (*p*-value < 0.05) were higher compared to the control group (Figure 6B).

Immunohistochemistry (IHC) was also utilized for examining the protein expression of HSPB1 and LEFTY2 in liver tissues from patients with PBC, as well as FCGR3B in liver tissues from patients with PSC. Based on the staining intensity in the cytoplasm and nuclei, we clearly observed an increased expression of HSPB1 and LEFTY2 in PBC tissues compared to normal tissues (*p*-value < 0.05) (Figure 6C, 6D). Likewise, FCGR3B exhibited higher expression in the PSC group compared to the normal group (*p*-value < 0.05) (Figure 6E).

## DISCUSSION

PBC is characterized by autoimmune destruction of intrahepatic bile ducts and primarily affects middle-aged women, with a prevalence rate of 10/100,000. On the other hand, PSC is associated with chronic inflammation and fibrosis of both intra- and extrahepatic bile ducts, with a prevalence range of 2.4–7.5/100,000 and no gender predilection [4, 7]. Although substantial progress has been made in comprehending PBC and PSC, they still present various research challenges. The precise mechanisms responsible for disease onset and progression, the availability of biomarkers for early diagnosis and disease monitoring, and effective therapeutic modalities remain limited [35–37]. Managing PBC and PSC is challenging due to multiple factors. While the current therapies primarily focus on symptom relief, slowing disease progression, and complications management, a curative treatment remains absent. The identification of novel therapeutic targets becomes crucial in meeting the unmet clinical requirements. Revealing the underlying molecular mechanisms and identifying the pathways involved in the pathogenesis of these diseases could lead to the discovery of potential targets for future interventions. Promisingly, novel therapies that specifically target these pathways have the potential to enhance outcomes for patients suffering from PBC and PSC.

This study employed an extensive Mendelian randomization analysis, integrating GWAS datasets, FINNGEN datasets, pharmacogenomic, and gene expression data (pQTL) to identify potential druggable targets for PBC and PSC. The analysis encompassed 557 patients with PBC and 281,127 controls, along with 1,715 patients with PSC and 330,903 controls. Our findings identified LEFTY2 and HSPB1 encoding proteins, with high and moderate confidence, respectively, as potential drug targets for PBC, and FCGR3B encoding protein, with high confidence, as a potential target for PSC.

HSPB1, also known as αβ-binding protein or HSP27, encodes a heat shock protein. HSPB1 serves as a molecular chaperone, regulating protein folding, promoting protein homodimerization, and binding to protein kinases. Additionally, HSP27 can be activated and participate in cellular stress responses like the heat shock response and oxidative stress. These functions enable the HSPB1 gene to play a crucial protective role in maintaining cell homeostasis. Research has demonstrated the significant involvement of the HSPB1 gene in various diseases [38–40]. Notably, diseases affecting the nervous system are particularly prominent. According to reports, mutations in the HSPB1 gene have been associated with neurodegenerative diseases, including Charcot-Marie-Tooth disease (CMT) [41] and late-onset amyotrophic lateral sclerosis (ALS) [42]. Such mutations could impair the function of the HSP27 protein, leading to nerve cell fragility and death.

Furthermore, the HSPB1 gene plays a role in the onset and progression of numerous liver diseases. In a study, notable variations in HSPB1 expression levels were observed between individuals with liver fibrosis and healthy controls, with higher HSPB1 expression showing a positive correlation with the severity of liver fibrosis [43]. Lu et al. conducted a study exploring the association between the HSPB1 gene and non-alcoholic fatty liver disease (NAFLD). The findings revealed a substantial increase in HSPB1 expression among patients with NAFLD, which exhibited a positive correlation with the extent of liver damage [44]. Our study revealed, for the first time, a causal relationship between HSPB1 and PBC. By integrating SMR and colocalization analyses, we confirmed that HSPB1 has a positive estimate effect (*p*-value = 7.24e-08 (IVW)) and a moderate co-localization relationship with PBC (*p*-HEIDI = 0.886, PP.H4 = 0.600). These findings were consistent with previous researches, suggesting that upregulated HSPB1 expression might promote the onset and progression of PBC. However, despite pQTL data from external datasets verifying the causal association between HSPB1 and PBC, a negative estimate effect was observed (Table 11). Additionally, we observed decreased HSPB1 expression in patients with PBC compared to normal individuals through DEG analysis of a GEO dataset. Contrarily, IHC analysis showed a higher expression intensity in PBC samples than control samples (Figure 6C). We speculate that the inconsistencies in these results are mainly attributed to an insufficient sample size in validation datasets, emphasizing the need for randomized controlled studies with larger sample sizes for further verification.

LEFTY2, also known as Left-right determination factor 2, emerged as a potential therapeutic target for PBC due to its strong association with the disease (*p*-HEIDI = 0.054, PP.H4 = 0.894) based on comprehensive analyses. LEFTY2 is known to exert significant effects on embryonic development and tissue repair [45, 46]. Recent evidence has increasingly implicated LEFTY2 in liver diseases. Investigating liver fibrosis, a study revealed substantial downregulation of LEFTY2 expression in affected patients. Subsequent functional studies elucidated that LEFTY2 mitigates liver fibrosis by suppressing hepatic stellate cell (HSC) activation and collagen synthesis [47]. Furthermore, a recently uncovered pathway involving the circCREBBP-has-miR-1291/LEFTY2 axis was shown to alleviate liver fibrosis [48]. Moreover, LEFTY2 has been implicated in the pathogenesis and advancement of endometrial cancer [49]. Nevertheless, this is the pioneering study to suggest LEFTY2 as a potential therapeutic target for PBC. Despite the lack of differential expression of LEFTY2 in the PBC group versus the control group in the external dataset, analysis of clinical liver samples from PBC patients revealed varying expression levels of LEFTY2 compared to normal samples. The limited sample size remains the primary limitation in terms of validation rigor.

According to our comprehensive analysis, among the potential druggable targets, FCGR3B emerged as a unique and promising candidate on PSC. The FCGR3B gene encodes Fcγ receptor IIIb (FcγRIIIb), which is highly expressed on immune cells, particularly neutrophils, thereby regulating the immune responses. As a result, FCGR3B has been implicated in various autoimmune diseases, leading to extensive research in this field [50–52]. Notably, variations in the FCGR3B gene have been closely linked to the occurrence and progression of viral hepatitis infections [53] and autoimmune liver diseases [54], in addition to systemic lupus erythematosus and pulmonary fibrosis. A deficiency of the FCGR3B gene has been shown to increase the risk of chronic hepatitis B [55]. Furthermore, it has been reported that monocytes with high expression of FCGR3B are positively correlated with disease progression, liver damage indicators, and serum C-reactive protein levels in patients with PBC [54]. Consistent with these findings, our analysis of the GEO dataset and experimental validation revealed upregulated expression of FCGR3B in the PSC group, which further strengthens the reliability of its potential positive impact on PSC (p-HEIDI = 0.194, PP.H4 = 0.900).

Furthermore, we utilized the DRUGBANK database, a comprehensive and accurate tool encompasses various drug information, to obtain a list of approved drugs corresponding to HSPB1 and FCGR3B, respectively. Apatorsen [56], Phenethyl Isothiocyanate [57], and Artemisinin Derivatives (anti-malaria drugs) [58] have been studied as cancer treatment drugs capable of inducing cell apoptosis and modulating immune response. However, currently, there are no clear studies showing a direct correlation or effect between these drugs and PBC. Our study initially revealed the potential therapeutic function of these drugs on PBC by targeting HSPB1. Based on current research and clinical practice, there is no clear evidence directly linking the 12 FCGR3B-targeted drugs to PSC. These drugs are primarily used for treating other diseases such as cancer [59], autoimmune diseases [60], and transplant rejection [61]. Nevertheless, the mechanisms of action of some drugs might have relevance to the treatment and research of PSC. For instance, Etanercept [62] and Sarilumab [63] are drugs used for treating rheumatoid arthritis, which have potential connections to PSC. Additionally, Natalizumab is a medication used for Crohn’s disease and also shows some association with PSC [64] However, treating PBC and PSC remains challenging, and further research is needed to determine the most effective treatment methods.

Our research utilized Mendelian randomization as a reliable research method and predictive tool for predicting drug target molecules. It has the advantages of randomized design, experimental control, and prospective study, potential causal relationships, and providing new perspectives for research. This contributes to drug development and personalized therapies, thus avoiding time-consuming, expensive and associated with a high failure rate in the conventional drug development process. Using MR, we successfully identified two druggable targets (LEFTY2 and HSPB1) that are correlated with PBC and one druggable target (FCGR3B) that is associated with PSC. These findings establish these genes as potential drug targets for PBC and PSC, significantly enhancing our understanding of these complex disorders. Nevertheless, it is important to note that the development of novel drugs is a highly rigorous, scientific, and intricate process. Our current work represents merely the initial stage of a lengthy endeavor. Subsequent investigations could capitalize on extensive genetic and proteomic datasets, in conjunction with comprehensive phenotypic data and clinical records, to elucidate the intricate genetic and environmental regulatory mechanisms that underlie PBC and PSC. Adopting this approach shows great potential for devising more precise and personalized treatment strategies, ultimately resulting in enhanced therapeutic outcomes for individuals affected by PBC and PSC.

However, our research is not without limitations. First, while we have made every effort to minimize genetic pleiotropy, it cannot be entirely eliminated. Moreover, some phenotypes/biomarkers might only be causative during specific stages of life, leading to the possible omission of certain causal associations. Second, despite the high degree of homogeneity in our study population, the generalizability of our findings to individuals from diverse ancestral backgrounds remains uncertain. Third, the modest sample sizes of our validation datasets and clinical samples might have compromised the reliability of our validations to some extent. Therefore, large-scale RCTs and fundamental research are necessary for further clarification. Additionally, prior to clinical trials, drug candidates ought to undergo validation *in vitro* cell models or animal models to assess their efficacy, safety, pharmacokinetics, and other relevant factors.

In summary, our research provides support for the therapeutic potential of targeting LEFTY2 and HSPB1 in intervening or halting the progression of PBC, as well as targeting FCGR3B in PSC. These novel druggable targets have the potential to effectively address the challenging nature of PBC and PSC treatments and offer fresh insights into exploring underlying mechanisms. Nevertheless, it is essential to conduct fundamental experiments and RCTs to assess the effectiveness and safety of preventive strategies for PBC and PSC.

## Supplementary Materials

Supplementary Figures

Supplementary Table 1

Supplementary Tables 2 and 6

Supplementary Table 3

Supplementary Table 4

Supplementary Table 5
