# Supplementary material for: Leveraging pQTL-based Mendelian randomization to identify new treatment prospects for primary biliary cholangitis and primary sclerosing cholangitis
Source: Aging (Albany NY). 2024 May 27;16(10):9228–50. doi: 10.18632/aging.205867 (PMC11164478; doi:10.18632/aging.205867)
Supplement: Supplementary Figures [file aging-16-205867-s001.pdf]

SUPPLEMENTARY FIGURES

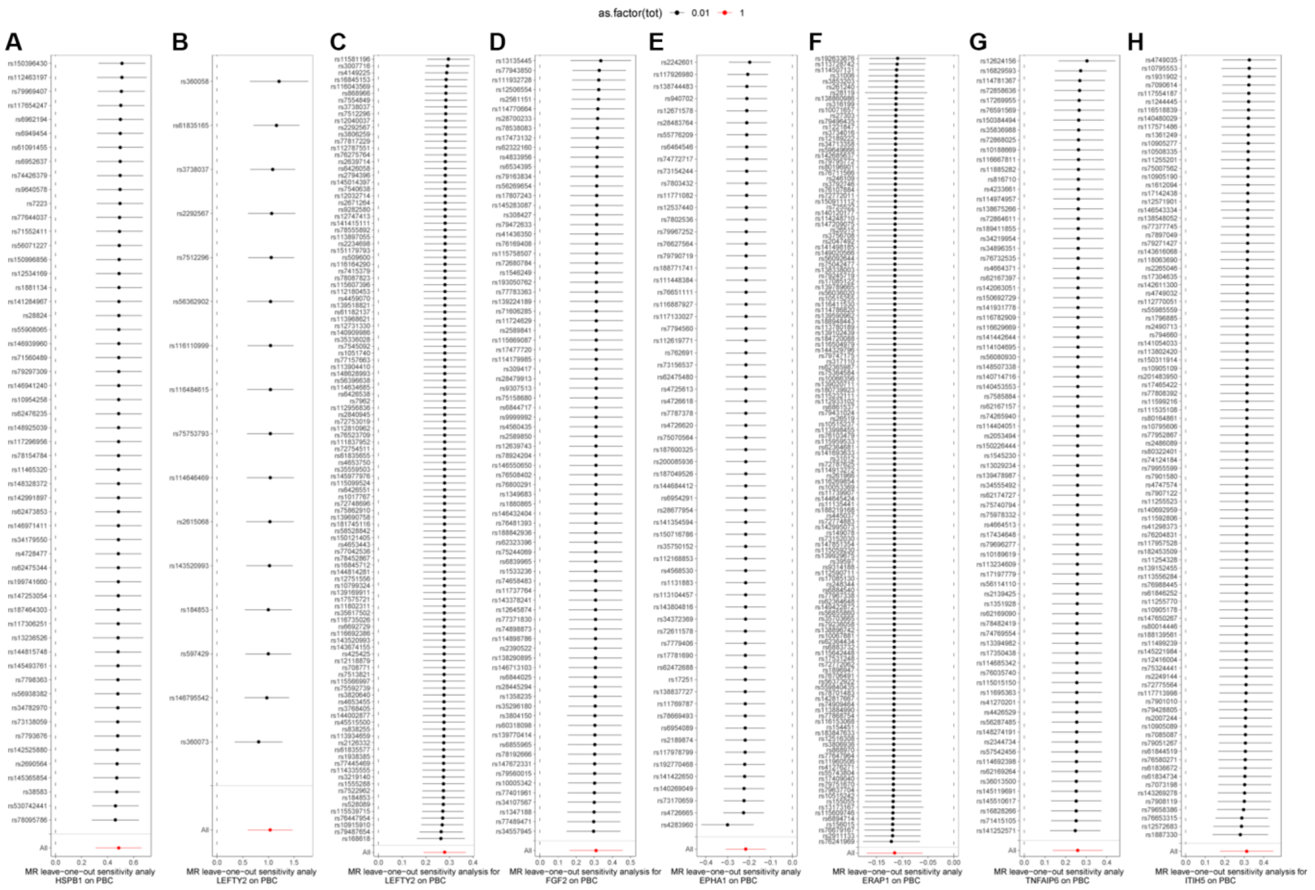

Supplementary Figure 1. Leave-one-out sensitivity analysis for potential druggable genes and PBC. (**A**) HSPB1; (**B**) LEFTY2 with primer 15503_15; (**C**) LEFTY2 with primer 15503_20; (**D**) FGF2; (**E**) EPHA1; (**F**) ERAP1; (**G**) TNFAIP6; (**H**) ITIH5. The dot and bar demonstrate the sensitivity of IV by removing SNPs one at a time. IV, instrument variable; SNPs, single nucleotide polymorphisms; PBC: primary biliary cholangitis.

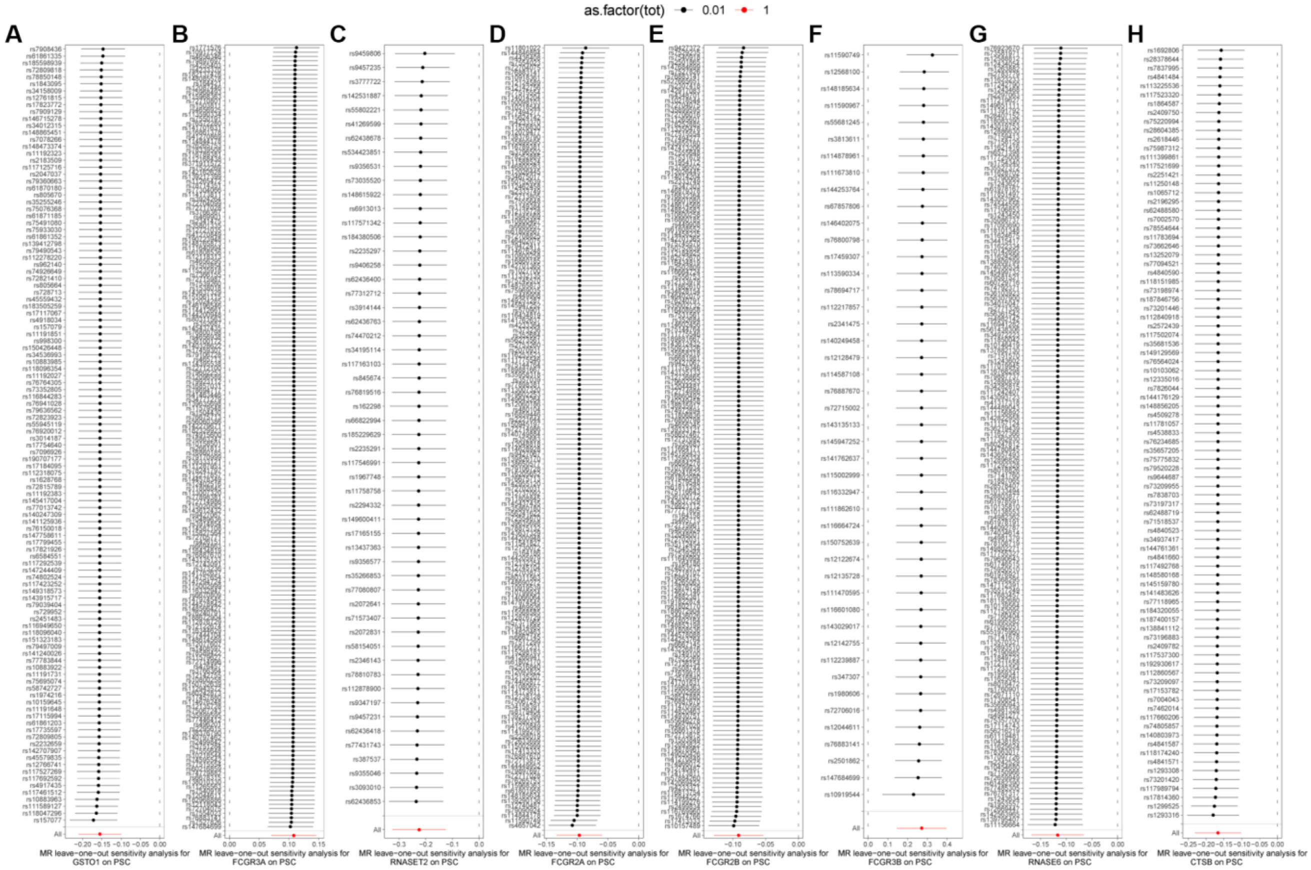

Supplementary Figure 2. Leave-one-out sensitivity analysis for potential druggable genes and PSC. (**A**) GSTO1; (**B**) FCGR3A; (**C**) RNASET2; (**D**) FCGR2A; (**E**) FCGR2B; (**F**) FCGR3B; (**G**) RNASE6; (**H**) CTSB. The dot and bar demonstrate the sensitivity of IV by removing SNPs one at a time. IV, instrument variable; SNPs, single nucleotide polymorphisms; PSC: primary sclerosing cholangitis.
