# Supplementary material for: Leveraging pQTL-based Mendelian randomization to identify new treatment prospects for primary biliary cholangitis and primary sclerosing cholangitis
Source: Aging (Albany NY). 2024 May 27;16(10):9228–50. doi: 10.18632/aging.205867 (PMC11164478; doi:10.18632/aging.205867)
Supplement: Supplementary Tables 2 and 6 [file aging-16-205867-s003.pdf]

SUPPLEMENTARY TABLES

Supplementary Table 2. Information of two outcome datasets (PBC and PSC) from FINNGEN database.

Name	Ncase	Ncontrol	Url
Primary biliary cholangitis (PBC)	557	281127	https://storage.googleapis.com/finngen-public-data-r9/summary_stats/finngen_R9_CHIRBIL_PRIM.gz
Cholangitis (primary sclerosing, PSC)	1715	330903	https://storage.googleapis.com/finngen-public-data-r9/summary_stats/finngen_R9_K11_CHOLANGI.gz

Supplementary Table 6. The results of reverse Mendelian randomization between ERAP1 coded protein and PBC.

id.exposure	id.outcome	Outcome	Exposure	Method	nsnp	b	se	*p*-val
KxOEMA	ERAP1	ERAP1	PBC	MR Egger	3	−0.01979	0.089422	0.861327
KxOEMA	ERAP1	ERAP1	PBC	Weighted median	3	0.013847	0.012106	0.252686
KxOEMA	ERAP1	ERAP1	PBC	Inverse variance weighted	3	0.023478	0.017492	0.179524
KxOEMA	ERAP1	ERAP1	PBC	Simple mode	3	0.010633	0.016608	0.587571
KxOEMA	ERAP1	ERAP1	PBC	Weighted mode	3	0.00895	0.014435	0.598468
