# Supplementary material for: Leveraging pQTL-based Mendelian randomization to identify new treatment prospects for primary biliary cholangitis and primary sclerosing cholangitis
Source: Aging (Albany NY). 2024 May 27;16(10):9228–50. doi: 10.18632/aging.205867 (PMC11164478; doi:10.18632/aging.205867)
Supplement: Supplementary Table 5 [file aging-16-205867-s006.pdf]

Supplementary Table 5. The detailed results of Mendelian randomization between 1046 druggable proteins and primary sclerosing cholangitis.

id.exposur	id.outcom	outcome	exposure	method	nsnp	b	se	pval
10037_98_	finngen_R	outcome	10037_98_	MR Egger	128	0.038122	0.039215	0.33285
10037_98_	finngen_R	outcome	10037_98_	Weighted	128	0.036696	0.04522	0.417072
10037_98_	finngen_R	outcome	10037_98_	Inverse va	128	0.020002	0.026433	0.449221
10037_98_	finngen_R	outcome	10037_98_	Simple mc	128	0.106074	0.077898	0.175701
10037_98_	finngen_R	outcome	10037_98_	Weighted	128	0.057225	0.036959	0.124027
10042_8_S	finngen_R	outcome	10042_8_S	Wald ratic	1	0.060078	1.420504	0.966265
10070_22_	finngen_R	outcome	10070_22_	MR Egger	9	-0.21015	0.394866	0.611044
10070_22_	finngen_R	outcome	10070_22_	Weighted	9	-0.38704	0.213252	0.069535
10070_22_	finngen_R	outcome	10070_22_	Inverse va	9	-0.22141	0.165647	0.181344
10070_22_	finngen_R	outcome	10070_22_	Simple mc	9	-0.44334	0.33474	0.221944
10070_22_	finngen_R	outcome	10070_22_	Weighted	9	-0.44334	0.275924	0.146777
10346_5_S	finngen_R	outcome	10346_5_S	MR Egger	11	0.795964	0.507678	0.151359
10346_5_S	finngen_R	outcome	10346_5_S	Weighted	11	0.352169	0.196277	0.072774
10346_5_S	finngen_R	outcome	10346_5_S	Inverse va	11	0.356831	0.165518	0.031095
10346_5_S	finngen_R	outcome	10346_5_S	Simple mc	11	0.056106	0.289231	0.850072
10346_5_S	finngen_R	outcome	10346_5_S	Weighted	11	0.403337	0.206864	0.079779
10366_11_	finngen_R	outcome	10366_11_	MR Egger	6	0.664376	1.22145	0.615392
10366_11_	finngen_R	outcome	10366_11_	Weighted	6	0.311927	0.298318	0.295736
10366_11_	finngen_R	outcome	10366_11_	Inverse va	6	0.176544	0.260927	0.498658
10366_11_	finngen_R	outcome	10366_11_	Simple mc	6	0.275095	0.398801	0.521019
10366_11_	finngen_R	outcome	10366_11_	Weighted	6	0.293837	0.313433	0.39155
10372_18_	finngen_R	outcome	10372_18_	MR Egger	11	0.19275	0.537315	0.72807
10372_18_	finngen_R	outcome	10372_18_	Weighted	11	0.087411	0.238346	0.713812
10372_18_	finngen_R	outcome	10372_18_	Inverse va	11	-0.09325	0.234308	0.690645
10372_18_	finngen_R	outcome	10372_18_	Simple mc	11	0.092041	0.384587	0.815689
10372_18_	finngen_R	outcome	10372_18_	Weighted	11	0.068864	0.298593	0.822253
10391_1_A	finngen_R	outcome	10391_1_A	MR Egger	42	0.101819	0.116612	0.3878
10391_1_A	finngen_R	outcome	10391_1_A	Weighted	42	-0.05365	0.09925	0.588787
10391_1_A	finngen_R	outcome	10391_1_A	Inverse va	42	0.074818	0.071615	0.296147
10391_1_A	finngen_R	outcome	10391_1_A	Simple mc	42	-0.19168	0.20289	0.350323
10391_1_A	finngen_R	outcome	10391_1_A	Weighted	42	0.030246	0.108309	0.78145
10396_6_N	finngen_R	outcome	10396_6_N	MR Egger	3	-0.0585	0.722256	0.948552
10396_6_N	finngen_R	outcome	10396_6_N	Weighted	3	-0.07523	0.232884	0.746661
10396_6_N	finngen_R	outcome	10396_6_N	Inverse va	3	-0.02905	0.24621	0.906066
10396_6_N	finngen_R	outcome	10396_6_N	Simple mc	3	-0.14083	0.325286	0.707266
10396_6_N	finngen_R	outcome	10396_6_N	Weighted	3	-0.08314	0.239004	0.761155
10451_11_	finngen_R	outcome	10451_11_	MR Egger	9	-0.75648	0.363163	0.075744
10451_11_	finngen_R	outcome	10451_11_	Weighted	9	-0.24414	0.212981	0.251663
10451_11_	finngen_R	outcome	10451_11_	Inverse va	9	-0.16799	0.174854	0.336668
10451_11_	finngen_R	outcome	10451_11_	Simple mc	9	-0.15697	0.312835	0.629347
10451_11_	finngen_R	outcome	10451_11_	Weighted	9	-0.32704	0.219672	0.174877
10462_14_	finngen_R	outcome	10462_14_	Wald ratic	1	-0.96266	0.619432	0.12016
10490_3_F	finngen_R	outcome	10490_3_F	MR Egger	9	-0.5025	0.499402	0.347824
10490_3_F	finngen_R	outcome	10490_3_F	Weighted	9	-0.21229	0.211265	0.31496
10490_3_F	finngen_R	outcome	10490_3_F	Inverse va	9	-0.13718	0.171506	0.423793
10490_3_F	finngen_R	outcome	10490_3_F	Simple mc	9	-0.30734	0.31807	0.362203
10490_3_F	finngen_R	outcome	10490_3_F	Weighted	9	-0.23435	0.211278	0.299572
10511_10_	finngen_R	outcome	10511_10_	MR Egger	6	-0.40169	1.050758	0.721693
10511_10_	finngen_R	outcome	10511_10_	Weighted	6	-0.14648	0.286741	0.609455
10511_10_	finngen_R	outcome	10511_10_	Inverse va	6	-0.21793	0.255853	0.394339
10511_10_	finngen_R	outcome	10511_10_	Simple mc	6	0.013692	0.491417	0.97885
10511_10_	finngen_R	outcome	10511_10_	Weighted	6	-0.05799	0.308881	0.85845
10512_13_	finngen_R	outcome	10512_13_	MR Egger	70	0.012909	0.113951	0.910138
10512_13_	finngen_R	outcome	10512_13_	Weighted	70	-0.12243	0.083512	0.142648
10512_13_	finngen_R	outcome	10512_13_	Inverse va	70	-0.054	0.058588	0.356684
10512_13_	finngen_R	outcome	10512_13_	Simple mc	70	-0.15855	0.144686	0.276981
10512_13_	finngen_R	outcome	10512_13_	Weighted	70	-0.13221	0.095047	0.16868

10514_5_F	finngen_R	outcome	10514_5_F	MR Egger	7	-0.07375	0.431274	0.870919
10514_5_F	finngen_R	outcome	10514_5_F	Weighted	7	0.1469	0.197394	0.456758
10514_5_F	finngen_R	outcome	10514_5_F	Inverse va	7	0.157012	0.166889	0.346798
10514_5_F	finngen_R	outcome	10514_5_F	Simple mc	7	0.089486	0.275978	0.756755
10514_5_F	finngen_R	outcome	10514_5_F	Weighted	7	0.104672	0.22759	0.661771
10534_40_	finngen_R	outcome	10534_40_	Wald ratic	1	-0.68681	0.642801	0.285313
10554_23_	finngen_R	outcome	10554_23_	Inverse va	2	-0.1499	0.573869	0.793932
10561_5_F	finngen_R	outcome	10561_5_F	MR Egger	3	0.125103	0.716985	0.890027
10561_5_F	finngen_R	outcome	10561_5_F	Weighted	3	-0.68983	0.430916	0.109409
10561_5_F	finngen_R	outcome	10561_5_F	Inverse va	3	-0.60411	0.39679	0.127885
10561_5_F	finngen_R	outcome	10561_5_F	Simple mc	3	-1.17014	0.696124	0.234794
10561_5_F	finngen_R	outcome	10561_5_F	Weighted	3	-0.0608	0.462817	0.907507
10569_28_	finngen_R	outcome	10569_28_	MR Egger	32	0.2009	0.203741	0.331992
10569_28_	finngen_R	outcome	10569_28_	Weighted	32	0.121265	0.128869	0.34671
10569_28_	finngen_R	outcome	10569_28_	Inverse va	32	0.054659	0.088641	0.537472
10569_28_	finngen_R	outcome	10569_28_	Simple mc	32	0.098043	0.225379	0.666567
10569_28_	finngen_R	outcome	10569_28_	Weighted	32	0.113162	0.147481	0.448709
10603_1_H	finngen_R	outcome	10603_1_H	MR Egger	4	0.313917	0.617738	0.661837
10603_1_H	finngen_R	outcome	10603_1_H	Weighted	4	0.077055	0.242523	0.750695
10603_1_H	finngen_R	outcome	10603_1_H	Inverse va	4	0.169556	0.299222	0.570947
10603_1_H	finngen_R	outcome	10603_1_H	Simple mc	4	-0.0397	0.340318	0.914504
10603_1_H	finngen_R	outcome	10603_1_H	Weighted	4	0.073486	0.253832	0.791049
10620_21_	finngen_R	outcome	10620_21_	MR Egger	93	0.049711	0.039855	0.21549
10620_21_	finngen_R	outcome	10620_21_	Weighted	93	0.048306	0.034443	0.160771
10620_21_	finngen_R	outcome	10620_21_	Inverse va	93	-0.00196	0.022036	0.928949
10620_21_	finngen_R	outcome	10620_21_	Simple mc	93	0.048687	0.062804	0.440196
10620_21_	finngen_R	outcome	10620_21_	Weighted	93	0.02916	0.032918	0.378018
10666_7_C	finngen_R	outcome	10666_7_C	MR Egger	26	0.16907	0.139796	0.238285
10666_7_C	finngen_R	outcome	10666_7_C	Weighted	26	0.101521	0.118928	0.393309
10666_7_C	finngen_R	outcome	10666_7_C	Inverse va	26	0.062262	0.084608	0.461801
10666_7_C	finngen_R	outcome	10666_7_C	Simple mc	26	-0.00264	0.211276	0.990126
10666_7_C	finngen_R	outcome	10666_7_C	Weighted	26	0.078569	0.111381	0.487077
10672_75_	finngen_R	outcome	10672_75_	MR Egger	37	0.023042	0.106636	0.830177
10672_75_	finngen_R	outcome	10672_75_	Weighted	37	0.051486	0.081981	0.529987
10672_75_	finngen_R	outcome	10672_75_	Inverse va	37	0.039923	0.061901	0.518963
10672_75_	finngen_R	outcome	10672_75_	Simple mc	37	0.074153	0.172792	0.670373
10672_75_	finngen_R	outcome	10672_75_	Weighted	37	0.057582	0.082847	0.491489
10708_3_C	finngen_R	outcome	10708_3_C	MR Egger	7	0.370432	0.28248	0.246725
10708_3_C	finngen_R	outcome	10708_3_C	Weighted	7	0.286861	0.198338	0.148087
10708_3_C	finngen_R	outcome	10708_3_C	Inverse va	7	0.283864	0.168635	0.092316
10708_3_C	finngen_R	outcome	10708_3_C	Simple mc	7	-0.0431	0.322512	0.898065
10708_3_C	finngen_R	outcome	10708_3_C	Weighted	7	0.291472	0.206956	0.208668
10714_7_A	finngen_R	outcome	10714_7_A	MR Egger	85	0.064661	0.05913	0.277319
10714_7_A	finngen_R	outcome	10714_7_A	Weighted	85	0.029937	0.05197	0.564588
10714_7_A	finngen_R	outcome	10714_7_A	Inverse va	85	0.050158	0.03285	0.126787
10714_7_A	finngen_R	outcome	10714_7_A	Simple mc	85	-0.01295	0.09205	0.888415
10714_7_A	finngen_R	outcome	10714_7_A	Weighted	85	0.056379	0.04175	0.180513
10722_13_	finngen_R	outcome	10722_13_	MR Egger	4	1.572483	0.976873	0.248747
10722_13_	finngen_R	outcome	10722_13_	Weighted	4	-0.65824	0.361732	0.068807
10722_13_	finngen_R	outcome	10722_13_	Inverse va	4	-0.69707	0.401337	0.082409
10722_13_	finngen_R	outcome	10722_13_	Simple mc	4	-0.62474	0.601604	0.375404
10722_13_	finngen_R	outcome	10722_13_	Weighted	4	-0.49534	0.488523	0.385269
10737_96_	finngen_R	outcome	10737_96_	Wald ratic	1	0.62062	0.438878	0.15733
10754_113	finngen_R	outcome	10754_113	MR Egger	74	0.024568	0.059524	0.68102
10754_113	finngen_R	outcome	10754_113	Weighted	74	0.008441	0.056772	0.881804
10754_113	finngen_R	outcome	10754_113	Inverse va	74	-0.01856	0.037649	0.621959
10754_113	finngen_R	outcome	10754_113	Simple mc	74	-0.40592	0.118584	0.001019
10754_113	finngen_R	outcome	10754_113	Weighted	74	0.015353	0.050094	0.760105

10809_14_	finngen_R	outcome	10809_14_	Inverse va	2	0.699582	0.782587	0.371357
10815_2_I	finngen_R	outcome	10815_2_I	MR Egger	35	-0.03939	0.195652	0.841697
10815_2_I	finngen_R	outcome	10815_2_I	Weighted	35	-0.20824	0.121797	0.087313
10815_2_I	finngen_R	outcome	10815_2_I	Inverse va	35	-0.2357	0.086066	0.006169
10815_2_I	finngen_R	outcome	10815_2_I	Simple mc	35	-0.50404	0.202747	0.017991
10815_2_I	finngen_R	outcome	10815_2_I	Weighted	35	-0.13688	0.166812	0.417616
10818_36_	finngen_R	outcome	10818_36_	MR Egger	43	0.048995	0.123574	0.693803
10818_36_	finngen_R	outcome	10818_36_	Weighted	43	0.043097	0.103664	0.677599
10818_36_	finngen_R	outcome	10818_36_	Inverse va	43	-0.00251	0.069289	0.97106
10818_36_	finngen_R	outcome	10818_36_	Simple mc	43	0.162388	0.215431	0.455184
10818_36_	finngen_R	outcome	10818_36_	Weighted	43	0.056783	0.112946	0.617768
10833_64_	finngen_R	outcome	10833_64_	MR Egger	9	-0.27324	0.346932	0.456767
10833_64_	finngen_R	outcome	10833_64_	Weighted	9	-0.19397	0.224254	0.387056
10833_64_	finngen_R	outcome	10833_64_	Inverse va	9	-0.09037	0.171266	0.597717
10833_64_	finngen_R	outcome	10833_64_	Simple mc	9	-0.09119	0.365231	0.809121
10833_64_	finngen_R	outcome	10833_64_	Weighted	9	-0.25077	0.246183	0.338185
10851_77_	finngen_R	outcome	10851_77_	MR Egger	181	0.023801	0.04338	0.583924
10851_77_	finngen_R	outcome	10851_77_	Weighted	181	-0.01472	0.044639	0.741664
10851_77_	finngen_R	outcome	10851_77_	Inverse va	181	-0.00768	0.02788	0.783023
10851_77_	finngen_R	outcome	10851_77_	Simple mc	181	0.048914	0.095929	0.610752
10851_77_	finngen_R	outcome	10851_77_	Weighted	181	0.003144	0.047555	0.94736
10907_11(	finngen_R	outcome	10907_11(	MR Egger	73	0.020364	0.090026	0.821696
10907_11(	finngen_R	outcome	10907_11(	Weighted	73	0.020083	0.077758	0.796198
10907_11(	finngen_R	outcome	10907_11(	Inverse va	73	0.058811	0.049398	0.233834
10907_11(	finngen_R	outcome	10907_11(	Simple mc	73	0.102505	0.167911	0.543472
10907_11(	finngen_R	outcome	10907_11(	Weighted	73	0.019176	0.080634	0.8127
10916_44_	finngen_R	outcome	10916_44_	MR Egger	120	-0.00677	0.047943	0.887891
10916_44_	finngen_R	outcome	10916_44_	Weighted	120	-0.04792	0.037677	0.203374
10916_44_	finngen_R	outcome	10916_44_	Inverse va	120	-0.04041	0.024867	0.104142
10916_44_	finngen_R	outcome	10916_44_	Simple mc	120	0.015216	0.075024	0.839631
10916_44_	finngen_R	outcome	10916_44_	Weighted	120	0.005576	0.040667	0.891178
10924_25{	finngen_R	outcome	10924_25{	MR Egger	14	-0.00708	0.157845	0.964975
10924_25{	finngen_R	outcome	10924_25{	Weighted	14	-0.01965	0.112854	0.861767
10924_25{	finngen_R	outcome	10924_25{	Inverse va	14	-0.00231	0.088665	0.979255
10924_25{	finngen_R	outcome	10924_25{	Simple mc	14	-0.08596	0.166574	0.614504
10924_25{	finngen_R	outcome	10924_25{	Weighted	14	-0.0136	0.120028	0.911534
10938_13_	finngen_R	outcome	10938_13_	MR Egger	4	-0.15884	0.186794	0.484697
10938_13_	finngen_R	outcome	10938_13_	Weighted	4	-0.10289	0.126119	0.414617
10938_13_	finngen_R	outcome	10938_13_	Inverse va	4	-0.091	0.120821	0.451335
10938_13_	finngen_R	outcome	10938_13_	Simple mc	4	-0.11486	0.196336	0.599642
10938_13_	finngen_R	outcome	10938_13_	Weighted	4	-0.10444	0.142168	0.515799
10977_55_	finngen_R	outcome	10977_55_	MR Egger	63	0.056372	0.106539	0.598638
10977_55_	finngen_R	outcome	10977_55_	Weighted	63	0.045417	0.083223	0.585251
10977_55_	finngen_R	outcome	10977_55_	Inverse va	63	-0.0366	0.054036	0.498187
10977_55_	finngen_R	outcome	10977_55_	Simple mc	63	-0.00039	0.157526	0.998039
10977_55_	finngen_R	outcome	10977_55_	Weighted	63	0.089212	0.118199	0.453249
11102_22_	finngen_R	outcome	11102_22_	MR Egger	10	-0.13263	0.429896	0.765578
11102_22_	finngen_R	outcome	11102_22_	Weighted	10	0.14662	0.181529	0.419267
11102_22_	finngen_R	outcome	11102_22_	Inverse va	10	0.312267	0.146122	0.032596
11102_22_	finngen_R	outcome	11102_22_	Simple mc	10	0.30768	0.300128	0.332051
11102_22_	finngen_R	outcome	11102_22_	Weighted	10	0.097303	0.204556	0.645638
11103_24_	finngen_R	outcome	11103_24_	MR Egger	55	0.067619	0.096619	0.487079
11103_24_	finngen_R	outcome	11103_24_	Weighted	55	0.060711	0.073792	0.41066
11103_24_	finngen_R	outcome	11103_24_	Inverse va	55	0.045909	0.051573	0.373371
11103_24_	finngen_R	outcome	11103_24_	Simple mc	55	0.109007	0.120768	0.370739
11103_24_	finngen_R	outcome	11103_24_	Weighted	55	0.015724	0.080862	0.846553
11104_13_	finngen_R	outcome	11104_13_	MR Egger	149	-0.04779	0.043754	0.276491
11104_13_	finngen_R	outcome	11104_13_	Weighted	149	-0.06787	0.039144	0.082948

11104_13_	finngen_R	outcome	11104_13_	Inverse va	149	-0.06609	0.025078	0.008408
11104_13_	finngen_R	outcome	11104_13_	Simple mc	149	-0.00669	0.081535	0.934727
11104_13_	finngen_R	outcome	11104_13_	Weighted	149	-0.07883	0.041632	0.06024
11126_102	finngen_R	outcome	11126_102	Wald ratic	1	0.321494	0.795018	0.685929
11140_56_	finngen_R	outcome	11140_56_	MR Egger	8	-0.22997	0.266857	0.421908
11140_56_	finngen_R	outcome	11140_56_	Weighted	8	0.059426	0.166821	0.721673
11140_56_	finngen_R	outcome	11140_56_	Inverse va	8	-0.07145	0.156783	0.648575
11140_56_	finngen_R	outcome	11140_56_	Simple mc	8	0.061141	0.268721	0.826518
11140_56_	finngen_R	outcome	11140_56_	Weighted	8	0.094672	0.21142	0.667835
11142_11_	finngen_R	outcome	11142_11_	MR Egger	51	-0.09691	0.111046	0.387099
11142_11_	finngen_R	outcome	11142_11_	Weighted	51	-0.01922	0.075134	0.79812
11142_11_	finngen_R	outcome	11142_11_	Inverse va	51	-0.043	0.056534	0.446946
11142_11_	finngen_R	outcome	11142_11_	Simple mc	51	0.068379	0.134122	0.612417
11142_11_	finngen_R	outcome	11142_11_	Weighted	51	-0.03023	0.074107	0.685076
11152_46_	finngen_R	outcome	11152_46_	MR Egger	32	0.179902	0.090189	0.055228
11152_46_	finngen_R	outcome	11152_46_	Weighted	32	0.104245	0.075393	0.16676
11152_46_	finngen_R	outcome	11152_46_	Inverse va	32	0.105206	0.054463	0.053396
11152_46_	finngen_R	outcome	11152_46_	Simple mc	32	0.000877	0.169662	0.995908
11152_46_	finngen_R	outcome	11152_46_	Weighted	32	0.097987	0.073525	0.192342
11192_168	finngen_R	outcome	11192_168	MR Egger	3	0.922308	0.69063	0.409179
11192_168	finngen_R	outcome	11192_168	Weighted	3	0.041662	0.354555	0.90646
11192_168	finngen_R	outcome	11192_168	Inverse va	3	0.184101	0.290539	0.526307
11192_168	finngen_R	outcome	11192_168	Simple mc	3	-0.14038	0.476415	0.79602
11192_168	finngen_R	outcome	11192_168	Weighted	3	-0.12129	0.479776	0.824035
11196_31_	finngen_R	outcome	11196_31_	MR Egger	3	0.790434	2.315282	0.790557
11196_31_	finngen_R	outcome	11196_31_	Weighted	3	-0.17262	0.414511	0.677093
11196_31_	finngen_R	outcome	11196_31_	Inverse va	3	-0.21183	0.368049	0.564912
11196_31_	finngen_R	outcome	11196_31_	Simple mc	3	-0.17849	0.509189	0.759414
11196_31_	finngen_R	outcome	11196_31_	Weighted	3	-0.12247	0.477021	0.821375
11219_95_	finngen_R	outcome	11219_95_	MR Egger	27	-0.04789	0.211182	0.82245
11219_95_	finngen_R	outcome	11219_95_	Weighted	27	0.205448	0.141503	0.146529
11219_95_	finngen_R	outcome	11219_95_	Inverse va	27	0.206581	0.10751	0.054668
11219_95_	finngen_R	outcome	11219_95_	Simple mc	27	0.152	0.229119	0.512907
11219_95_	finngen_R	outcome	11219_95_	Weighted	27	0.152	0.185047	0.418881
11237_49_	finngen_R	outcome	11237_49_	MR Egger	15	0.119431	0.275893	0.67219
11237_49_	finngen_R	outcome	11237_49_	Weighted	15	-0.29713	0.171377	0.082953
11237_49_	finngen_R	outcome	11237_49_	Inverse va	15	-0.13612	0.125297	0.27731
11237_49_	finngen_R	outcome	11237_49_	Simple mc	15	-0.17061	0.318336	0.600417
11237_49_	finngen_R	outcome	11237_49_	Weighted	15	-0.30257	0.216004	0.183057
11257_1_(	finngen_R	outcome	11257_1_(	MR Egger	36	0.019956	0.115599	0.863967
11257_1_(	finngen_R	outcome	11257_1_(	Weighted	36	-0.01015	0.093326	0.913415
11257_1_(	finngen_R	outcome	11257_1_(	Inverse va	36	0.034739	0.0655	0.595861
11257_1_(	finngen_R	outcome	11257_1_(	Simple mc	36	-0.03858	0.16572	0.81726
11257_1_(	finngen_R	outcome	11257_1_(	Weighted	36	-0.002	0.083374	0.981016
11265_8_/	finngen_R	outcome	11265_8_/	MR Egger	5	-1.03854	0.799711	0.284874
11265_8_/	finngen_R	outcome	11265_8_/	Weighted	5	-0.39843	0.319823	0.212846
11265_8_/	finngen_R	outcome	11265_8_/	Inverse va	5	-0.36934	0.272944	0.176007
11265_8_/	finngen_R	outcome	11265_8_/	Simple mc	5	-0.47645	0.486979	0.38328
11265_8_/	finngen_R	outcome	11265_8_/	Weighted	5	-0.39094	0.379533	0.361196
11266_8_S	finngen_R	outcome	11266_8_S	MR Egger	32	0.15701	0.125482	0.220512
11266_8_S	finngen_R	outcome	11266_8_S	Weighted	32	-0.043	0.112625	0.702605
11266_8_S	finngen_R	outcome	11266_8_S	Inverse va	32	-0.12938	0.07779	0.096283
11266_8_S	finngen_R	outcome	11266_8_S	Simple mc	32	-0.03265	0.167108	0.846374
11266_8_S	finngen_R	outcome	11266_8_S	Weighted	32	0.031142	0.104334	0.767323
11273_176	finngen_R	outcome	11273_176	MR Egger	23	-0.10702	0.153143	0.492316
11273_176	finngen_R	outcome	11273_176	Weighted	23	-0.15685	0.121328	0.1961
11273_176	finngen_R	outcome	11273_176	Inverse va	23	-0.25691	0.089196	0.003973
11273_176	finngen_R	outcome	11273_176	Simple mc	23	-0.35845	0.217777	0.113992

11273_17(	finngen_R	outcome	11273_17(	Weighted	23	-0.24396	0.117069	0.04899
11302_237	finngen_R	outcome	11302_237	MR Egger	5	0.040455	0.746982	0.960215
11302_237	finngen_R	outcome	11302_237	Weighted	5	-0.24695	0.224575	0.271499
11302_237	finngen_R	outcome	11302_237	Inverse va	5	-0.21507	0.220563	0.329524
11302_237	finngen_R	outcome	11302_237	Simple mc	5	-0.25531	0.321665	0.471782
11302_237	finngen_R	outcome	11302_237	Weighted	5	-0.26278	0.252132	0.356151
11360_39_	finngen_R	outcome	11360_39_	Wald ratic	1	-0.58924	0.626349	0.34683
11369_23_	finngen_R	outcome	11369_23_	MR Egger	15	-0.08138	0.266799	0.765164
11369_23_	finngen_R	outcome	11369_23_	Weighted	15	-0.15986	0.179318	0.372657
11369_23_	finngen_R	outcome	11369_23_	Inverse va	15	-0.04315	0.132599	0.744884
11369_23_	finngen_R	outcome	11369_23_	Simple mc	15	0.502313	0.333066	0.153749
11369_23_	finngen_R	outcome	11369_23_	Weighted	15	-0.20974	0.22196	0.360709
11377_19_	finngen_R	outcome	11377_19_	MR Egger	29	0.05993	0.153841	0.699921
11377_19_	finngen_R	outcome	11377_19_	Weighted	29	0.135183	0.108179	0.211434
11377_19_	finngen_R	outcome	11377_19_	Inverse va	29	0.206722	0.093824	0.027575
11377_19_	finngen_R	outcome	11377_19_	Simple mc	29	0.183808	0.224432	0.419707
11377_19_	finngen_R	outcome	11377_19_	Weighted	29	0.117238	0.10807	0.287246
11378_37_	finngen_R	outcome	11378_37_	MR Egger	6	-0.1517	0.647053	0.826148
11378_37_	finngen_R	outcome	11378_37_	Weighted	6	-0.46944	0.292296	0.108263
11378_37_	finngen_R	outcome	11378_37_	Inverse va	6	-0.41895	0.231969	0.070907
11378_37_	finngen_R	outcome	11378_37_	Simple mc	6	-0.73581	0.447976	0.161404
11378_37_	finngen_R	outcome	11378_37_	Weighted	6	-0.74977	0.468192	0.170185
11388_75_	finngen_R	outcome	11388_75_	Inverse va	2	-0.09554	0.719996	0.894432
11390_24_	finngen_R	outcome	11390_24_	MR Egger	55	0.078593	0.095451	0.41398
11390_24_	finngen_R	outcome	11390_24_	Weighted	55	0.040852	0.073827	0.580022
11390_24_	finngen_R	outcome	11390_24_	Inverse va	55	-0.02739	0.052741	0.603556
11390_24_	finngen_R	outcome	11390_24_	Simple mc	55	-0.13921	0.145142	0.341766
11390_24_	finngen_R	outcome	11390_24_	Weighted	55	-0.0083	0.07253	0.909271
11431_23!	finngen_R	outcome	11431_23!	MR Egger	34	0.098009	0.116219	0.405313
11431_23!	finngen_R	outcome	11431_23!	Weighted	34	0.165722	0.109708	0.130897
11431_23!	finngen_R	outcome	11431_23!	Inverse va	34	0.209407	0.074213	0.004777
11431_23!	finngen_R	outcome	11431_23!	Simple mc	34	0.175481	0.190789	0.36437
11431_23!	finngen_R	outcome	11431_23!	Weighted	34	0.189664	0.157375	0.236709
11441_11_	finngen_R	outcome	11441_11_	MR Egger	3	0.043585	0.527858	0.947554
11441_11_	finngen_R	outcome	11441_11_	Weighted	3	0.060824	0.238204	0.798459
11441_11_	finngen_R	outcome	11441_11_	Inverse va	3	0.065312	0.225909	0.7725
11441_11_	finngen_R	outcome	11441_11_	Simple mc	3	0.139354	0.301841	0.689662
11441_11_	finngen_R	outcome	11441_11_	Weighted	3	0.074686	0.294434	0.823454
11480_1_/	finngen_R	outcome	11480_1_/	MR Egger	3	-0.10133	0.683695	0.906327
11480_1_/	finngen_R	outcome	11480_1_/	Weighted	3	-0.23211	0.262206	0.376032
11480_1_/	finngen_R	outcome	11480_1_/	Inverse va	3	-0.26748	0.2473	0.279426
11480_1_/	finngen_R	outcome	11480_1_/	Simple mc	3	-0.12653	0.359485	0.758487
11480_1_/	finngen_R	outcome	11480_1_/	Weighted	3	-0.20693	0.295597	0.556367
11510_31_	finngen_R	outcome	11510_31_	MR Egger	34	-0.02709	0.126868	0.832254
11510_31_	finngen_R	outcome	11510_31_	Weighted	34	-0.09951	0.093896	0.289253
11510_31_	finngen_R	outcome	11510_31_	Inverse va	34	0.006607	0.075935	0.930662
11510_31_	finngen_R	outcome	11510_31_	Simple mc	34	-0.10732	0.194099	0.584054
11510_31_	finngen_R	outcome	11510_31_	Weighted	34	-0.09474	0.085952	0.278355
11514_19(	finngen_R	outcome	11514_19(	MR Egger	8	-0.29024	0.29357	0.361004
11514_19(	finngen_R	outcome	11514_19(	Weighted	8	0.14127	0.158015	0.371306
11514_19(	finngen_R	outcome	11514_19(	Inverse va	8	0.15112	0.134663	0.261772
11514_19(	finngen_R	outcome	11514_19(	Simple mc	8	0.299724	0.263171	0.292217
11514_19(	finngen_R	outcome	11514_19(	Weighted	8	0.143698	0.161254	0.402449
11516_7_F	finngen_R	outcome	11516_7_F	Wald ratic	1	-0.43672	0.314486	0.164934
11531_24_	finngen_R	outcome	11531_24_	MR Egger	26	0.080678	0.177439	0.653423
11531_24_	finngen_R	outcome	11531_24_	Weighted	26	0.108432	0.114447	0.343414
11531_24_	finngen_R	outcome	11531_24_	Inverse va	26	0.084727	0.089184	0.3421
11531_24_	finngen_R	outcome	11531_24_	Simple mc	26	0.056509	0.215612	0.795401

11531_24_	finngen_R	outcome	11531_24_	Weighted	26	0.130848	0.114953	0.265796
11547_84_	finngen_R	outcome	11547_84_	Inverse va	2	0.733813	0.405896	0.070624
11568_2_F	finngen_R	outcome	11568_2_F	MR Egger	17	0.355768	0.20255	0.099402
11568_2_F	finngen_R	outcome	11568_2_F	Weighted	17	0.278038	0.13387	0.037809
11568_2_F	finngen_R	outcome	11568_2_F	Inverse va	17	0.325357	0.097872	0.000886
11568_2_F	finngen_R	outcome	11568_2_F	Simple mc	17	0.215202	0.229595	0.362533
11568_2_F	finngen_R	outcome	11568_2_F	Weighted	17	0.28918	0.150727	0.073062
11646_4_(	finngen_R	outcome	11646_4_(	MR Egger	30	0.013327	0.189363	0.944392
11646_4_(	finngen_R	outcome	11646_4_(	Weighted	30	0.092865	0.121568	0.444931
11646_4_(	finngen_R	outcome	11646_4_(	Inverse va	30	0.081333	0.081743	0.319745
11646_4_(	finngen_R	outcome	11646_4_(	Simple mc	30	0.176297	0.178412	0.331255
11646_4_(	finngen_R	outcome	11646_4_(	Weighted	30	0.080213	0.116153	0.495317
11708_2_L	finngen_R	outcome	11708_2_L	Wald ratic	1	-1.20379	0.541698	0.026266
12008_3_(	finngen_R	outcome	12008_3_(	MR Egger	12	0.193946	0.21398	0.386058
12008_3_(	finngen_R	outcome	12008_3_(	Weighted	12	0.155719	0.170234	0.360332
12008_3_(	finngen_R	outcome	12008_3_(	Inverse va	12	0.10595	0.126796	0.403382
12008_3_(	finngen_R	outcome	12008_3_(	Simple mc	12	0.212388	0.272706	0.452515
12008_3_(	finngen_R	outcome	12008_3_(	Weighted	12	0.187437	0.170177	0.294231
12329_21_	finngen_R	outcome	12329_21_	MR Egger	4	2.169619	1.170966	0.20509
12329_21_	finngen_R	outcome	12329_21_	Weighted	4	-0.63077	0.313354	0.044117
12329_21_	finngen_R	outcome	12329_21_	Inverse va	4	-0.79595	0.439235	0.069968
12329_21_	finngen_R	outcome	12329_21_	Simple mc	4	-0.55777	0.453951	0.306781
12329_21_	finngen_R	outcome	12329_21_	Weighted	4	-0.54566	0.346968	0.213848
12338_27_	finngen_R	outcome	12338_27_	MR Egger	16	-0.01459	0.172342	0.933746
12338_27_	finngen_R	outcome	12338_27_	Weighted	16	-0.21835	0.15719	0.164805
12338_27_	finngen_R	outcome	12338_27_	Inverse va	16	-0.34161	0.104759	0.00111
12338_27_	finngen_R	outcome	12338_27_	Simple mc	16	-0.34885	0.28938	0.246681
12338_27_	finngen_R	outcome	12338_27_	Weighted	16	-0.06976	0.1881	0.715903
12357_41_	finngen_R	outcome	12357_41_	Inverse va	2	0.127136	0.358982	0.723222
12370_30_	finngen_R	outcome	12370_30_	MR Egger	16	-0.06496	0.338965	0.850777
12370_30_	finngen_R	outcome	12370_30_	Weighted	16	-0.12828	0.192348	0.504834
12370_30_	finngen_R	outcome	12370_30_	Inverse va	16	-0.21151	0.189768	0.265024
12370_30_	finngen_R	outcome	12370_30_	Simple mc	16	-0.21438	0.424775	0.621104
12370_30_	finngen_R	outcome	12370_30_	Weighted	16	-0.04621	0.210664	0.829332
12381_26_	finngen_R	outcome	12381_26_	MR Egger	18	0.235677	0.206169	0.269804
12381_26_	finngen_R	outcome	12381_26_	Weighted	18	0.245535	0.174082	0.158407
12381_26_	finngen_R	outcome	12381_26_	Inverse va	18	0.074114	0.118643	0.532181
12381_26_	finngen_R	outcome	12381_26_	Simple mc	18	0.336646	0.283972	0.252132
12381_26_	finngen_R	outcome	12381_26_	Weighted	18	0.211393	0.179473	0.255083
12386_11_	finngen_R	outcome	12386_11_	MR Egger	53	0.176935	0.103078	0.092138
12386_11_	finngen_R	outcome	12386_11_	Weighted	53	0.087176	0.082356	0.289818
12386_11_	finngen_R	outcome	12386_11_	Inverse va	53	0.119357	0.056175	0.033609
12386_11_	finngen_R	outcome	12386_11_	Simple mc	53	0.02933	0.143772	0.839149
12386_11_	finngen_R	outcome	12386_11_	Weighted	53	0.098304	0.084967	0.252571
12422_14:	finngen_R	outcome	12422_14:	Wald ratic	1	0.23893	0.509713	0.639245
12436_84_	finngen_R	outcome	12436_84_	MR Egger	112	-0.11984	0.049966	0.018151
12436_84_	finngen_R	outcome	12436_84_	Weighted	112	-0.10658	0.041902	0.010972
12436_84_	finngen_R	outcome	12436_84_	Inverse va	112	-0.15523	0.028185	3.64E-08
12436_84_	finngen_R	outcome	12436_84_	Simple mc	112	-0.16005	0.072936	0.030293
12436_84_	finngen_R	outcome	12436_84_	Weighted	112	-0.14886	0.037381	0.000122
12437_18_	finngen_R	outcome	12437_18_	Wald ratic	1	-0.22504	0.423068	0.594771
12449_16_	finngen_R	outcome	12449_16_	MR Egger	3	0.61299	2.927389	0.868592
12449_16_	finngen_R	outcome	12449_16_	Weighted	3	0.593431	0.388551	0.126688
12449_16_	finngen_R	outcome	12449_16_	Inverse va	3	0.597775	0.339657	0.078418
12449_16_	finngen_R	outcome	12449_16_	Simple mc	3	0.515505	0.458537	0.377715
12449_16_	finngen_R	outcome	12449_16_	Weighted	3	0.651264	0.471415	0.301213
12549_33_	finngen_R	outcome	12549_33_	MR Egger	65	0.00552	0.055968	0.921741
12549_33_	finngen_R	outcome	12549_33_	Weighted	65	0.025931	0.05568	0.641423

12549_33_	finngen_R	outcome	12549_33_	Inverse va	65	0.020871	0.03946	0.596866
12549_33_	finngen_R	outcome	12549_33_	Simple mc	65	0.171175	0.131509	0.197713
12549_33_	finngen_R	outcome	12549_33_	Weighted	65	0.037069	0.049423	0.455987
12612_37_	finngen_R	outcome	12612_37_	MR Egger	25	-0.20801	0.166274	0.2235
12612_37_	finngen_R	outcome	12612_37_	Weighted	25	-0.15975	0.103913	0.124212
12612_37_	finngen_R	outcome	12612_37_	Inverse va	25	-0.10567	0.076192	0.165473
12612_37_	finngen_R	outcome	12612_37_	Simple mc	25	-0.28241	0.208981	0.189183
12612_37_	finngen_R	outcome	12612_37_	Weighted	25	-0.14495	0.095952	0.143937
12616_45_	finngen_R	outcome	12616_45_	Wald ratic	1	-0.64236	0.716342	0.369871
12703_6_N	finngen_R	outcome	12703_6_N	Wald ratic	1	0.298038	0.509651	0.558689
12727_7_F	finngen_R	outcome	12727_7_F	MR Egger	100	0.092232	0.041243	0.027602
12727_7_F	finngen_R	outcome	12727_7_F	Weighted	100	0.067965	0.045032	0.131228
12727_7_F	finngen_R	outcome	12727_7_F	Inverse va	100	0.075664	0.027134	0.005295
12727_7_F	finngen_R	outcome	12727_7_F	Simple mc	100	-0.00322	0.082975	0.969143
12727_7_F	finngen_R	outcome	12727_7_F	Weighted	100	0.099876	0.03714	0.008407
12940_35_	finngen_R	outcome	12940_35_	MR Egger	3	0.156766	0.911746	0.891599
12940_35_	finngen_R	outcome	12940_35_	Weighted	3	0.248806	0.268764	0.354581
12940_35_	finngen_R	outcome	12940_35_	Inverse va	3	0.27124	0.288304	0.346801
12940_35_	finngen_R	outcome	12940_35_	Simple mc	3	0.024695	0.417728	0.958235
12940_35_	finngen_R	outcome	12940_35_	Weighted	3	0.197845	0.308502	0.587005
12990_39_	finngen_R	outcome	12990_39_	Wald ratic	1	-0.16075	0.747552	0.829736
13044_5_T	finngen_R	outcome	13044_5_T	MR Egger	6	-0.56645	0.603108	0.400797
13044_5_T	finngen_R	outcome	13044_5_T	Weighted	6	-0.62367	0.221798	0.004925
13044_5_T	finngen_R	outcome	13044_5_T	Inverse va	6	-0.60958	0.233229	0.008959
13044_5_T	finngen_R	outcome	13044_5_T	Simple mc	6	0.102115	0.485471	0.841703
13044_5_T	finngen_R	outcome	13044_5_T	Weighted	6	-0.62516	0.224927	0.03893
13088_397	finngen_R	outcome	13088_397	MR Egger	11	0.123878	0.33968	0.723765
13088_397	finngen_R	outcome	13088_397	Weighted	11	-0.09135	0.173707	0.598949
13088_397	finngen_R	outcome	13088_397	Inverse va	11	-0.17862	0.135542	0.187566
13088_397	finngen_R	outcome	13088_397	Simple mc	11	-0.22452	0.240922	0.373333
13088_397	finngen_R	outcome	13088_397	Weighted	11	-0.04445	0.189751	0.819528
13093_6_S	finngen_R	outcome	13093_6_S	MR Egger	15	0.237261	0.268629	0.393152
13093_6_S	finngen_R	outcome	13093_6_S	Weighted	15	0.345123	0.140299	0.013897
13093_6_S	finngen_R	outcome	13093_6_S	Inverse va	15	0.354278	0.11508	0.00208
13093_6_S	finngen_R	outcome	13093_6_S	Simple mc	15	0.314562	0.22623	0.1861
13093_6_S	finngen_R	outcome	13093_6_S	Weighted	15	0.328425	0.131008	0.025129
13094_75_	finngen_R	outcome	13094_75_	MR Egger	17	0.366708	0.253929	0.169264
13094_75_	finngen_R	outcome	13094_75_	Weighted	17	0.215109	0.13886	0.121357
13094_75_	finngen_R	outcome	13094_75_	Inverse va	17	0.386964	0.108718	0.000372
13094_75_	finngen_R	outcome	13094_75_	Simple mc	17	0.504922	0.220754	0.036139
13094_75_	finngen_R	outcome	13094_75_	Weighted	17	0.25057	0.135385	0.08275
13095_51_	finngen_R	outcome	13095_51_	MR Egger	48	0.119504	0.116129	0.308831
13095_51_	finngen_R	outcome	13095_51_	Weighted	48	0.126597	0.093772	0.177003
13095_51_	finngen_R	outcome	13095_51_	Inverse va	48	0.142354	0.064008	0.026149
13095_51_	finngen_R	outcome	13095_51_	Simple mc	48	0.125362	0.181319	0.492721
13095_51_	finngen_R	outcome	13095_51_	Weighted	48	0.192167	0.109851	0.08676
13109_82_	finngen_R	outcome	13109_82_	MR Egger	10	0.362859	0.517523	0.503093
13109_82_	finngen_R	outcome	13109_82_	Weighted	10	0.028447	0.224903	0.899347
13109_82_	finngen_R	outcome	13109_82_	Inverse va	10	0.055353	0.164344	0.73626
13109_82_	finngen_R	outcome	13109_82_	Simple mc	10	0.288346	0.331507	0.407007
13109_82_	finngen_R	outcome	13109_82_	Weighted	10	0.05135	0.240687	0.835809
13112_179	finngen_R	outcome	13112_179	MR Egger	8	0.285053	0.81162	0.737431
13112_179	finngen_R	outcome	13112_179	Weighted	8	0.380898	0.21158	0.071821
13112_179	finngen_R	outcome	13112_179	Inverse va	8	0.36645	0.224981	0.103354
13112_179	finngen_R	outcome	13112_179	Simple mc	8	0.046297	0.490812	0.927493
13112_179	finngen_R	outcome	13112_179	Weighted	8	0.470438	0.238527	0.089194
13113_7_S	finngen_R	outcome	13113_7_S	MR Egger	8	-0.52176	0.802635	0.539729
13113_7_S	finngen_R	outcome	13113_7_S	Weighted	8	0.165322	0.288849	0.567087

13113_7_S	finngen_R	outcome	13113_7_S	Inverse va	8	0.17158	0.244437	0.482719
13113_7_S	finngen_R	outcome	13113_7_S	Simple mc	8	0.653921	0.489135	0.223069
13113_7_S	finngen_R	outcome	13113_7_S	Weighted	8	0.583621	0.478436	0.26202
13114_50_	finngen_R	outcome	13114_50_	MR Egger	17	0.210939	0.181289	0.26278
13114_50_	finngen_R	outcome	13114_50_	Weighted	17	0.14728	0.156661	0.347155
13114_50_	finngen_R	outcome	13114_50_	Inverse va	17	0.084012	0.113018	0.457269
13114_50_	finngen_R	outcome	13114_50_	Simple mc	17	0.154013	0.256387	0.55645
13114_50_	finngen_R	outcome	13114_50_	Weighted	17	0.17662	0.185244	0.354547
13116_25_	finngen_R	outcome	13116_25_	MR Egger	126	-0.09278	0.038119	0.016366
13116_25_	finngen_R	outcome	13116_25_	Weighted	126	-0.0303	0.033292	0.362823
13116_25_	finngen_R	outcome	13116_25_	Inverse va	126	-0.0643	0.023283	0.005748
13116_25_	finngen_R	outcome	13116_25_	Simple mc	126	-0.00375	0.065624	0.954462
13116_25_	finngen_R	outcome	13116_25_	Weighted	126	-0.03905	0.031868	0.222694
13118_5_S	finngen_R	outcome	13118_5_S	MR Egger	21	0.188429	0.237248	0.436872
13118_5_S	finngen_R	outcome	13118_5_S	Weighted	21	-0.10261	0.140428	0.464978
13118_5_S	finngen_R	outcome	13118_5_S	Inverse va	21	-0.07559	0.097871	0.439894
13118_5_S	finngen_R	outcome	13118_5_S	Simple mc	21	-0.31533	0.237272	0.19882
13118_5_S	finngen_R	outcome	13118_5_S	Weighted	21	-0.19753	0.186397	0.301898
13119_26_	finngen_R	outcome	13119_26_	MR Egger	119	0.111655	0.04756	0.020571
13119_26_	finngen_R	outcome	13119_26_	Weighted	119	0.085078	0.051847	0.100804
13119_26_	finngen_R	outcome	13119_26_	Inverse va	119	0.093492	0.029684	0.001635
13119_26_	finngen_R	outcome	13119_26_	Simple mc	119	0.119971	0.098686	0.226532
13119_26_	finngen_R	outcome	13119_26_	Weighted	119	0.106924	0.048471	0.029327
13122_19_	finngen_R	outcome	13122_19_	MR Egger	43	-0.01422	0.178546	0.936923
13122_19_	finngen_R	outcome	13122_19_	Weighted	43	-0.0122	0.115109	0.915582
13122_19_	finngen_R	outcome	13122_19_	Inverse va	43	-0.01276	0.081015	0.874827
13122_19_	finngen_R	outcome	13122_19_	Simple mc	43	0.10597	0.191596	0.583132
13122_19_	finngen_R	outcome	13122_19_	Weighted	43	0.027187	0.123634	0.827018
13124_20_	finngen_R	outcome	13124_20_	MR Egger	32	0.13753	0.248075	0.583422
13124_20_	finngen_R	outcome	13124_20_	Weighted	32	0.093709	0.136361	0.49195
13124_20_	finngen_R	outcome	13124_20_	Inverse va	32	-0.08964	0.10152	0.37726
13124_20_	finngen_R	outcome	13124_20_	Simple mc	32	0.040945	0.25415	0.873055
13124_20_	finngen_R	outcome	13124_20_	Weighted	32	0.081003	0.144464	0.579024
13133_73_	finngen_R	outcome	13133_73_	Inverse va	2	-0.03067	0.256904	0.904974
13381_49_	finngen_R	outcome	13381_49_	MR Egger	13	-0.0901	0.261504	0.736924
13381_49_	finngen_R	outcome	13381_49_	Weighted	13	-0.13333	0.157182	0.396301
13381_49_	finngen_R	outcome	13381_49_	Inverse va	13	-0.1226	0.135259	0.36474
13381_49_	finngen_R	outcome	13381_49_	Simple mc	13	-0.2891	0.238703	0.249163
13381_49_	finngen_R	outcome	13381_49_	Weighted	13	-0.14476	0.149067	0.35065
13388_57_	finngen_R	outcome	13388_57_	MR Egger	124	0.113202	0.048788	0.021986
13388_57_	finngen_R	outcome	13388_57_	Weighted	124	0.079294	0.044377	0.073962
13388_57_	finngen_R	outcome	13388_57_	Inverse va	124	0.071007	0.027666	0.010272
13388_57_	finngen_R	outcome	13388_57_	Simple mc	124	-0.03012	0.086367	0.727913
13388_57_	finngen_R	outcome	13388_57_	Weighted	124	0.042561	0.043242	0.326915
13397_88_	finngen_R	outcome	13397_88_	MR Egger	20	0.520225	0.373758	0.180921
13397_88_	finngen_R	outcome	13397_88_	Weighted	20	-0.25754	0.215314	0.231642
13397_88_	finngen_R	outcome	13397_88_	Inverse va	20	-0.21225	0.193688	0.273142
13397_88_	finngen_R	outcome	13397_88_	Simple mc	20	-0.49701	0.443741	0.27665
13397_88_	finngen_R	outcome	13397_88_	Weighted	20	-0.2387	0.296886	0.431338
13405_61_	finngen_R	outcome	13405_61_	MR Egger	47	-0.10659	0.098814	0.286478
13405_61_	finngen_R	outcome	13405_61_	Weighted	47	-0.0158	0.087877	0.857329
13405_61_	finngen_R	outcome	13405_61_	Inverse va	47	-0.0053	0.057758	0.926823
13405_61_	finngen_R	outcome	13405_61_	Simple mc	47	0.008662	0.143087	0.951991
13405_61_	finngen_R	outcome	13405_61_	Weighted	47	-0.01494	0.096251	0.877343
13438_115	finngen_R	outcome	13438_115	Inverse va	2	0.386583	0.638903	0.54513
13460_4_C	finngen_R	outcome	13460_4_C	Wald ratic	1	-0.38125	0.748466	0.610489
13463_1_F	finngen_R	outcome	13463_1_F	MR Egger	11	-0.70881	0.460797	0.158375
13463_1_F	finngen_R	outcome	13463_1_F	Weighted	11	-0.17351	0.188358	0.356966

13463_1_F	finngen_R	outcome	13463_1_F	Inverse va	11	-0.16031	0.176478	0.363663
13463_1_F	finngen_R	outcome	13463_1_F	Simple mc	11	0.217563	0.321403	0.513813
13463_1_F	finngen_R	outcome	13463_1_F	Weighted	11	-0.16001	0.195982	0.433267
13666_222	finngen_R	outcome	13666_222	MR Egger	18	0.276307	0.128785	0.047604
13666_222	finngen_R	outcome	13666_222	Weighted	18	0.096324	0.113167	0.394677
13666_222	finngen_R	outcome	13666_222	Inverse va	18	0.048227	0.091988	0.600084
13666_222	finngen_R	outcome	13666_222	Simple mc	18	-0.43338	0.247977	0.098562
13666_222	finngen_R	outcome	13666_222	Weighted	18	0.10567	0.112437	0.360476
13669_6_F	finngen_R	outcome	13669_6_F	MR Egger	23	0.410289	0.33083	0.228587
13669_6_F	finngen_R	outcome	13669_6_F	Weighted	23	0.142872	0.167299	0.39311
13669_6_F	finngen_R	outcome	13669_6_F	Inverse va	23	0.15392	0.130485	0.238159
13669_6_F	finngen_R	outcome	13669_6_F	Simple mc	23	0.010203	0.274944	0.970732
13669_6_F	finngen_R	outcome	13669_6_F	Weighted	23	0.221251	0.180462	0.233152
13671_40_	finngen_R	outcome	13671_40_	MR Egger	60	0.171531	0.098145	0.085804
13671_40_	finngen_R	outcome	13671_40_	Weighted	60	0.066382	0.079075	0.401195
13671_40_	finngen_R	outcome	13671_40_	Inverse va	60	0.049375	0.055942	0.377445
13671_40_	finngen_R	outcome	13671_40_	Simple mc	60	0.040599	0.144315	0.779449
13671_40_	finngen_R	outcome	13671_40_	Weighted	60	0.067304	0.080409	0.40596
13676_46_	finngen_R	outcome	13676_46_	MR Egger	23	0.031493	0.129473	0.81018
13676_46_	finngen_R	outcome	13676_46_	Weighted	23	0.032986	0.091696	0.719043
13676_46_	finngen_R	outcome	13676_46_	Inverse va	23	0.061869	0.069235	0.371535
13676_46_	finngen_R	outcome	13676_46_	Simple mc	23	0.127214	0.199914	0.531118
13676_46_	finngen_R	outcome	13676_46_	Weighted	23	0.030589	0.089105	0.734639
13682_47_	finngen_R	outcome	13682_47_	MR Egger	10	0.5267	0.534885	0.353613
13682_47_	finngen_R	outcome	13682_47_	Weighted	10	0.236049	0.232207	0.30937
13682_47_	finngen_R	outcome	13682_47_	Inverse va	10	-0.05902	0.177841	0.73999
13682_47_	finngen_R	outcome	13682_47_	Simple mc	10	0.33327	0.384086	0.408101
13682_47_	finngen_R	outcome	13682_47_	Weighted	10	0.288547	0.293291	0.350909
13686_2_II	finngen_R	outcome	13686_2_II	MR Egger	86	-0.05522	0.063609	0.387797
13686_2_II	finngen_R	outcome	13686_2_II	Weighted	86	-0.09232	0.054417	0.089778
13686_2_II	finngen_R	outcome	13686_2_II	Inverse va	86	-0.02696	0.036957	0.465754
13686_2_II	finngen_R	outcome	13686_2_II	Simple mc	86	-0.0869	0.101133	0.392602
13686_2_II	finngen_R	outcome	13686_2_II	Weighted	86	-0.09899	0.061987	0.11398
13692_154	finngen_R	outcome	13692_154	MR Egger	125	-0.11759	0.056069	0.038021
13692_154	finngen_R	outcome	13692_154	Weighted	125	-0.03224	0.049675	0.516342
13692_154	finngen_R	outcome	13692_154	Inverse va	125	-0.01171	0.0313	0.708267
13692_154	finngen_R	outcome	13692_154	Simple mc	125	0.042054	0.092119	0.648819
13692_154	finngen_R	outcome	13692_154	Weighted	125	-0.0425	0.046727	0.364879
13700_10_	finngen_R	outcome	13700_10_	MR Egger	110	0.056327	0.051515	0.276649
13700_10_	finngen_R	outcome	13700_10_	Weighted	110	-0.00862	0.052269	0.86907
13700_10_	finngen_R	outcome	13700_10_	Inverse va	110	0.037555	0.031478	0.232855
13700_10_	finngen_R	outcome	13700_10_	Simple mc	110	-0.02252	0.106206	0.832496
13700_10_	finngen_R	outcome	13700_10_	Weighted	110	0.003672	0.05194	0.943772
13717_15_	finngen_R	outcome	13717_15_	MR Egger	131	-0.03228	0.042403	0.447939
13717_15_	finngen_R	outcome	13717_15_	Weighted	131	-0.00156	0.04574	0.972815
13717_15_	finngen_R	outcome	13717_15_	Inverse va	131	-0.03304	0.027436	0.228522
13717_15_	finngen_R	outcome	13717_15_	Simple mc	131	0.06299	0.081859	0.442996
13717_15_	finngen_R	outcome	13717_15_	Weighted	131	-0.00995	0.038532	0.796717
13719_19_	finngen_R	outcome	13719_19_	Wald ratic	1	0.418633	0.77589	0.589506
13720_95_	finngen_R	outcome	13720_95_	MR Egger	65	-0.065	0.108724	0.552066
13720_95_	finngen_R	outcome	13720_95_	Weighted	65	-0.0719	0.081984	0.380463
13720_95_	finngen_R	outcome	13720_95_	Inverse va	65	-0.01922	0.053508	0.719507
13720_95_	finngen_R	outcome	13720_95_	Simple mc	65	-0.20452	0.153975	0.188813
13720_95_	finngen_R	outcome	13720_95_	Weighted	65	-0.14652	0.109739	0.186546
13733_5_II	finngen_R	outcome	13733_5_II	MR Egger	52	-0.06169	0.077195	0.427994
13733_5_II	finngen_R	outcome	13733_5_II	Weighted	52	0.002446	0.067687	0.971175
13733_5_II	finngen_R	outcome	13733_5_II	Inverse va	52	-0.00963	0.049831	0.846736
13733_5_II	finngen_R	outcome	13733_5_II	Simple mc	52	0.11	0.137149	0.426246

13733_5_I	finngen_R	outcome	13733_5_I	Weighted	52	0.000903	0.064187	0.98883
13738_8_I	finngen_R	outcome	13738_8_I	Wald ratic	1	0.368978	0.409441	0.367496
13740_51_	finngen_R	outcome	13740_51_	MR Egger	55	0.091291	0.09732	0.352476
13740_51_	finngen_R	outcome	13740_51_	Weighted	55	0.098694	0.093946	0.29347
13740_51_	finngen_R	outcome	13740_51_	Inverse va	55	0.116018	0.055836	0.037723
13740_51_	finngen_R	outcome	13740_51_	Simple mc	55	0.315271	0.204596	0.12917
13740_51_	finngen_R	outcome	13740_51_	Weighted	55	0.094188	0.102138	0.360546
13748_4_(	finngen_R	outcome	13748_4_(	MR Egger	159	-0.0599	0.036464	0.102453
13748_4_(	finngen_R	outcome	13748_4_(	Weighted	159	-0.04907	0.038956	0.207772
13748_4_(	finngen_R	outcome	13748_4_(	Inverse va	159	-0.07535	0.023891	0.00161
13748_4_(	finngen_R	outcome	13748_4_(	Simple mc	159	-0.05656	0.076514	0.460901
13748_4_(	finngen_R	outcome	13748_4_(	Weighted	159	-0.06728	0.034753	0.054676
13930_3_/	finngen_R	outcome	13930_3_/	MR Egger	4	0.158231	0.74866	0.852193
13930_3_/	finngen_R	outcome	13930_3_/	Weighted	4	0.055876	0.244156	0.818982
13930_3_/	finngen_R	outcome	13930_3_/	Inverse va	4	0.0958	0.217076	0.658981
13930_3_/	finngen_R	outcome	13930_3_/	Simple mc	4	0.000212	0.338402	0.99954
13930_3_/	finngen_R	outcome	13930_3_/	Weighted	4	0.015907	0.286659	0.959235
13944_3_S	finngen_R	outcome	13944_3_S	MR Egger	17	-0.29288	0.272357	0.299217
13944_3_S	finngen_R	outcome	13944_3_S	Weighted	17	-0.0699	0.169124	0.679391
13944_3_S	finngen_R	outcome	13944_3_S	Inverse va	17	0.02711	0.12593	0.829551
13944_3_S	finngen_R	outcome	13944_3_S	Simple mc	17	-0.19524	0.248548	0.443619
13944_3_S	finngen_R	outcome	13944_3_S	Weighted	17	-0.08722	0.197589	0.664807
13959_7_L	finngen_R	outcome	13959_7_L	MR Egger	8	0.341886	0.307821	0.309229
13959_7_L	finngen_R	outcome	13959_7_L	Weighted	8	0.072725	0.245608	0.767152
13959_7_L	finngen_R	outcome	13959_7_L	Inverse va	8	0.174322	0.197735	0.377998
13959_7_L	finngen_R	outcome	13959_7_L	Simple mc	8	0.068688	0.376207	0.860301
13959_7_L	finngen_R	outcome	13959_7_L	Weighted	8	0.051663	0.305326	0.870421
13972_4_H	finngen_R	outcome	13972_4_H	MR Egger	4	0.029954	1.533402	0.986188
13972_4_H	finngen_R	outcome	13972_4_H	Weighted	4	-0.05339	0.472594	0.910055
13972_4_H	finngen_R	outcome	13972_4_H	Inverse va	4	0.005457	0.491947	0.991149
13972_4_H	finngen_R	outcome	13972_4_H	Simple mc	4	0.813515	0.857434	0.412729
13972_4_H	finngen_R	outcome	13972_4_H	Weighted	4	-0.24177	0.574029	0.701977
13973_62_	finngen_R	outcome	13973_62_	Wald ratic	1	-0.49588	0.882597	0.57422
14047_78_	finngen_R	outcome	14047_78_	Inverse va	2	0.304868	0.285972	0.28639
14048_7_I	finngen_R	outcome	14048_7_I	MR Egger	142	0.034753	0.026498	0.191824
14048_7_I	finngen_R	outcome	14048_7_I	Weighted	142	-0.00021	0.032013	0.994648
14048_7_I	finngen_R	outcome	14048_7_I	Inverse va	142	0.008171	0.020075	0.683992
14048_7_I	finngen_R	outcome	14048_7_I	Simple mc	142	-0.02969	0.068073	0.663431
14048_7_I	finngen_R	outcome	14048_7_I	Weighted	142	0.007362	0.025231	0.770877
14054_17_	finngen_R	outcome	14054_17_	MR Egger	86	-0.04547	0.066656	0.496975
14054_17_	finngen_R	outcome	14054_17_	Weighted	86	-0.04603	0.053126	0.386235
14054_17_	finngen_R	outcome	14054_17_	Inverse va	86	-0.0342	0.035236	0.331807
14054_17_	finngen_R	outcome	14054_17_	Simple mc	86	-0.04055	0.096667	0.675921
14054_17_	finngen_R	outcome	14054_17_	Weighted	86	-0.04055	0.054168	0.456163
14076_74_	finngen_R	outcome	14076_74_	MR Egger	42	0.172249	0.158816	0.2846
14076_74_	finngen_R	outcome	14076_74_	Weighted	42	0.1536	0.106385	0.148794
14076_74_	finngen_R	outcome	14076_74_	Inverse va	42	0.039735	0.079374	0.61665
14076_74_	finngen_R	outcome	14076_74_	Simple mc	42	0.046363	0.185596	0.803983
14076_74_	finngen_R	outcome	14076_74_	Weighted	42	0.123959	0.105614	0.24729
14079_14_	finngen_R	outcome	14079_14_	MR Egger	71	-0.0371	0.059632	0.535912
14079_14_	finngen_R	outcome	14079_14_	Weighted	71	-0.08172	0.050336	0.104479
14079_14_	finngen_R	outcome	14079_14_	Inverse va	71	-0.04903	0.032557	0.132098
14079_14_	finngen_R	outcome	14079_14_	Simple mc	71	-0.06895	0.087434	0.432982
14079_14_	finngen_R	outcome	14079_14_	Weighted	71	-0.07616	0.049459	0.128104
14091_42_	finngen_R	outcome	14091_42_	MR Egger	40	-0.14062	0.086598	0.112684
14091_42_	finngen_R	outcome	14091_42_	Weighted	40	-0.02744	0.076543	0.719952
14091_42_	finngen_R	outcome	14091_42_	Inverse va	40	-0.04954	0.053434	0.353828
14091_42_	finngen_R	outcome	14091_42_	Simple mc	40	-0.00895	0.147173	0.951841

14091_42_	finngen_R	outcome	14091_42_	Weighted	40	-0.0811	0.065054	0.219935
14094_29_	finngen_R	outcome	14094_29_	Wald ratic	1	-0.19847	0.688745	0.773221
14100_63_	finngen_R	outcome	14100_63_	MR Egger	97	-0.1444	0.051357	0.005986
14100_63_	finngen_R	outcome	14100_63_	Weighted	97	-0.13564	0.054317	0.012519
14100_63_	finngen_R	outcome	14100_63_	Inverse va	97	-0.05042	0.033063	0.12725
14100_63_	finngen_R	outcome	14100_63_	Simple mc	97	0.005831	0.093279	0.950282
14100_63_	finngen_R	outcome	14100_63_	Weighted	97	-0.13522	0.046559	0.004569
14101_2_(	finngen_R	outcome	14101_2_(	MR Egger	13	0.074547	0.154082	0.638007
14101_2_(	finngen_R	outcome	14101_2_(	Weighted	13	0.047314	0.122827	0.700081
14101_2_(	finngen_R	outcome	14101_2_(	Inverse va	13	0.108664	0.094633	0.250862
14101_2_(	finngen_R	outcome	14101_2_(	Simple mc	13	0.062197	0.191095	0.750424
14101_2_(	finngen_R	outcome	14101_2_(	Weighted	13	0.051886	0.122131	0.678472
14111_15_	finngen_R	outcome	14111_15_	Wald ratic	1	0.041198	0.44487	0.926216
14112_40_	finngen_R	outcome	14112_40_	Wald ratic	1	0.12542	0.632635	0.842849
14123_34_	finngen_R	outcome	14123_34_	MR Egger	35	0.035188	0.133407	0.793603
14123_34_	finngen_R	outcome	14123_34_	Weighted	35	0.062837	0.101126	0.534351
14123_34_	finngen_R	outcome	14123_34_	Inverse va	35	0.049545	0.075218	0.510095
14123_34_	finngen_R	outcome	14123_34_	Simple mc	35	0.185511	0.202504	0.366074
14123_34_	finngen_R	outcome	14123_34_	Weighted	35	0.110628	0.086441	0.209277
14133_93_	finngen_R	outcome	14133_93_	MR Egger	64	0.011486	0.098874	0.907897
14133_93_	finngen_R	outcome	14133_93_	Weighted	64	-0.03421	0.069238	0.621274
14133_93_	finngen_R	outcome	14133_93_	Inverse va	64	-0.02562	0.043856	0.559129
14133_93_	finngen_R	outcome	14133_93_	Simple mc	64	-0.09104	0.110367	0.412532
14133_93_	finngen_R	outcome	14133_93_	Weighted	64	-0.04337	0.068784	0.53064
14151_4_I	finngen_R	outcome	14151_4_I	MR Egger	25	0.116163	0.159285	0.473193
14151_4_I	finngen_R	outcome	14151_4_I	Weighted	25	0.133756	0.101004	0.185417
14151_4_I	finngen_R	outcome	14151_4_I	Inverse va	25	0.132325	0.076839	0.085049
14151_4_I	finngen_R	outcome	14151_4_I	Simple mc	25	0.042601	0.22834	0.853566
14151_4_I	finngen_R	outcome	14151_4_I	Weighted	25	0.128791	0.095408	0.189647
14273_19_	finngen_R	outcome	14273_19_	MR Egger	15	0.064618	0.328845	0.847257
14273_19_	finngen_R	outcome	14273_19_	Weighted	15	0.08271	0.178481	0.643072
14273_19_	finngen_R	outcome	14273_19_	Inverse va	15	0.012902	0.134744	0.923717
14273_19_	finngen_R	outcome	14273_19_	Simple mc	15	-0.0997	0.343149	0.775664
14273_19_	finngen_R	outcome	14273_19_	Weighted	15	0.070252	0.199563	0.73006
14684_17_	finngen_R	outcome	14684_17_	MR Egger	14	0.234369	0.179827	0.216924
14684_17_	finngen_R	outcome	14684_17_	Weighted	14	0.161489	0.157337	0.304708
14684_17_	finngen_R	outcome	14684_17_	Inverse va	14	0.149583	0.123675	0.226479
14684_17_	finngen_R	outcome	14684_17_	Simple mc	14	0.081492	0.246043	0.745765
14684_17_	finngen_R	outcome	14684_17_	Weighted	14	0.153792	0.163372	0.363685
14688_6_F	finngen_R	outcome	14688_6_F	MR Egger	3	3.303065	4.263059	0.580345
14688_6_F	finngen_R	outcome	14688_6_F	Weighted	3	-0.35239	0.507312	0.487296
14688_6_F	finngen_R	outcome	14688_6_F	Inverse va	3	-0.27638	0.472245	0.558379
14688_6_F	finngen_R	outcome	14688_6_F	Simple mc	3	-0.34378	0.571878	0.608803
14688_6_F	finngen_R	outcome	14688_6_F	Weighted	3	-0.36219	0.545259	0.574861
14708_59_	finngen_R	outcome	14708_59_	MR Egger	77	0.130055	0.082096	0.117362
14708_59_	finngen_R	outcome	14708_59_	Weighted	77	0.114818	0.081948	0.161182
14708_59_	finngen_R	outcome	14708_59_	Inverse va	77	0.064878	0.046776	0.165448
14708_59_	finngen_R	outcome	14708_59_	Simple mc	77	0.110827	0.131636	0.402475
14708_59_	finngen_R	outcome	14708_59_	Weighted	77	0.121235	0.077915	0.123866
14711_27_	finngen_R	outcome	14711_27_	MR Egger	6	-0.0107	0.246252	0.967438
14711_27_	finngen_R	outcome	14711_27_	Weighted	6	-0.06962	0.187982	0.711116
14711_27_	finngen_R	outcome	14711_27_	Inverse va	6	-0.16347	0.163287	0.316761
14711_27_	finngen_R	outcome	14711_27_	Simple mc	6	-0.33675	0.376357	0.411909
14711_27_	finngen_R	outcome	14711_27_	Weighted	6	-0.04189	0.189059	0.833399
14747_9_(	finngen_R	outcome	14747_9_(	MR Egger	27	0.063746	0.188625	0.738221
14747_9_(	finngen_R	outcome	14747_9_(	Weighted	27	0.092218	0.125438	0.462237
14747_9_(	finngen_R	outcome	14747_9_(	Inverse va	27	0.124544	0.089954	0.166197
14747_9_(	finngen_R	outcome	14747_9_(	Simple mc	27	0.164574	0.191588	0.398192

14747_9_(	finngen_R	outcome	14747_9_(	Weighted	27	0.105555	0.130152	0.424722
15304_1_F	finngen_R	outcome	15304_1_F	MR Egger	34	0.108085	0.143979	0.458318
15304_1_F	finngen_R	outcome	15304_1_F	Weighted	34	0.144773	0.112828	0.199446
15304_1_F	finngen_R	outcome	15304_1_F	Inverse va	34	0.098315	0.076137	0.196602
15304_1_F	finngen_R	outcome	15304_1_F	Simple mc	34	0.033304	0.19565	0.865873
15304_1_F	finngen_R	outcome	15304_1_F	Weighted	34	0.179489	0.129279	0.174321
15305_7_S	finngen_R	outcome	15305_7_S	MR Egger	3	-1.29774	2.890326	0.731335
15305_7_S	finngen_R	outcome	15305_7_S	Weighted	3	-0.86908	0.453862	0.055512
15305_7_S	finngen_R	outcome	15305_7_S	Inverse va	3	-1.03116	0.401504	0.010221
15305_7_S	finngen_R	outcome	15305_7_S	Simple mc	3	-0.8571	0.537728	0.251981
15305_7_S	finngen_R	outcome	15305_7_S	Weighted	3	-0.8249	0.473221	0.223428
15308_108	finngen_R	outcome	15308_108	MR Egger	44	0.063142	0.131434	0.633431
15308_108	finngen_R	outcome	15308_108	Weighted	44	0.098369	0.104294	0.345587
15308_108	finngen_R	outcome	15308_108	Inverse va	44	0.161506	0.068411	0.018235
15308_108	finngen_R	outcome	15308_108	Simple mc	44	0.136322	0.176588	0.44435
15308_108	finngen_R	outcome	15308_108	Weighted	44	0.146433	0.120862	0.232294
15333_11_	finngen_R	outcome	15333_11_	MR Egger	20	0.099206	0.256622	0.703597
15333_11_	finngen_R	outcome	15333_11_	Weighted	20	0.13344	0.129302	0.302071
15333_11_	finngen_R	outcome	15333_11_	Inverse va	20	0.115828	0.102684	0.259317
15333_11_	finngen_R	outcome	15333_11_	Simple mc	20	0.023282	0.24058	0.923919
15333_11_	finngen_R	outcome	15333_11_	Weighted	20	0.081472	0.143398	0.576584
15343_337	finngen_R	outcome	15343_337	MR Egger	45	-0.08058	0.081295	0.327126
15343_337	finngen_R	outcome	15343_337	Weighted	45	-0.01983	0.071201	0.78065
15343_337	finngen_R	outcome	15343_337	Inverse va	45	0.040906	0.052724	0.437839
15343_337	finngen_R	outcome	15343_337	Simple mc	45	0.020686	0.131508	0.875726
15343_337	finngen_R	outcome	15343_337	Weighted	45	-0.01367	0.06531	0.835222
15363_32_	finngen_R	outcome	15363_32_	MR Egger	72	0.144484	0.048491	0.003965
15363_32_	finngen_R	outcome	15363_32_	Weighted	72	0.126344	0.048254	0.008837
15363_32_	finngen_R	outcome	15363_32_	Inverse va	72	0.09369	0.03337	0.004992
15363_32_	finngen_R	outcome	15363_32_	Simple mc	72	0.106308	0.085824	0.219545
15363_32_	finngen_R	outcome	15363_32_	Weighted	72	0.121727	0.042235	0.005222
15364_101	finngen_R	outcome	15364_101	MR Egger	38	0.065552	0.209754	0.75645
15364_101	finngen_R	outcome	15364_101	Weighted	38	0.031603	0.13532	0.815338
15364_101	finngen_R	outcome	15364_101	Inverse va	38	0.064235	0.104259	0.537824
15364_101	finngen_R	outcome	15364_101	Simple mc	38	0.187658	0.294179	0.527464
15364_101	finngen_R	outcome	15364_101	Weighted	38	-0.02923	0.20823	0.889128
15367_38_	finngen_R	outcome	15367_38_	MR Egger	56	-0.08009	0.091811	0.386866
15367_38_	finngen_R	outcome	15367_38_	Weighted	56	-0.08846	0.075761	0.242964
15367_38_	finngen_R	outcome	15367_38_	Inverse va	56	-0.04387	0.046895	0.349567
15367_38_	finngen_R	outcome	15367_38_	Simple mc	56	0.030976	0.143441	0.829827
15367_38_	finngen_R	outcome	15367_38_	Weighted	56	-0.05993	0.077283	0.441368
15368_3_E	finngen_R	outcome	15368_3_E	MR Egger	12	-0.45865	0.390894	0.267854
15368_3_E	finngen_R	outcome	15368_3_E	Weighted	12	-0.23205	0.181897	0.202055
15368_3_E	finngen_R	outcome	15368_3_E	Inverse va	12	-0.13067	0.145911	0.370496
15368_3_E	finngen_R	outcome	15368_3_E	Simple mc	12	-0.0897	0.271923	0.747704
15368_3_E	finngen_R	outcome	15368_3_E	Weighted	12	-0.2347	0.197732	0.260246
15370_5_E	finngen_R	outcome	15370_5_E	MR Egger	18	0.007409	0.121204	0.952017
15370_5_E	finngen_R	outcome	15370_5_E	Weighted	18	0.009829	0.076508	0.897776
15370_5_E	finngen_R	outcome	15370_5_E	Inverse va	18	0.023621	0.087671	0.787606
15370_5_E	finngen_R	outcome	15370_5_E	Simple mc	18	0.160866	0.167453	0.350187
15370_5_E	finngen_R	outcome	15370_5_E	Weighted	18	0.022663	0.065665	0.734225
15375_49_	finngen_R	outcome	15375_49_	MR Egger	11	-0.0749	0.424024	0.863697
15375_49_	finngen_R	outcome	15375_49_	Weighted	11	-0.06133	0.160286	0.701987
15375_49_	finngen_R	outcome	15375_49_	Inverse va	11	0.06702	0.205794	0.744679
15375_49_	finngen_R	outcome	15375_49_	Simple mc	11	0.336507	0.342173	0.348585
15375_49_	finngen_R	outcome	15375_49_	Weighted	11	-0.05362	0.160713	0.745563
15376_134	finngen_R	outcome	15376_134	MR Egger	12	0.178695	0.111558	0.140278
15376_134	finngen_R	outcome	15376_134	Weighted	12	0.136665	0.080167	0.088239

15376_134	finngen_R	outcome	15376_134	Inverse va	12	0.108334	0.073566	0.140856
15376_134	finngen_R	outcome	15376_134	Simple mc	12	0.032667	0.182747	0.861379
15376_134	finngen_R	outcome	15376_134	Weighted	12	0.127468	0.078639	0.133319
15384_15_	finngen_R	outcome	15384_15_	MR Egger	24	-0.09681	0.184847	0.605713
15384_15_	finngen_R	outcome	15384_15_	Weighted	24	-0.1441	0.125202	0.249773
15384_15_	finngen_R	outcome	15384_15_	Inverse va	24	-0.06716	0.091914	0.464947
15384_15_	finngen_R	outcome	15384_15_	Simple mc	24	-0.15483	0.178382	0.394374
15384_15_	finngen_R	outcome	15384_15_	Weighted	24	-0.13701	0.135981	0.324138
15385_116	finngen_R	outcome	15385_116	MR Egger	89	-0.00452	0.058306	0.938335
15385_116	finngen_R	outcome	15385_116	Weighted	89	-0.00135	0.046211	0.976638
15385_116	finngen_R	outcome	15385_116	Inverse va	89	-0.02201	0.030625	0.472284
15385_116	finngen_R	outcome	15385_116	Simple mc	89	0.020796	0.074096	0.779627
15385_116	finngen_R	outcome	15385_116	Weighted	89	0.007886	0.042199	0.852182
15388_24_	finngen_R	outcome	15388_24_	MR Egger	176	0.137299	0.035905	0.000183
15388_24_	finngen_R	outcome	15388_24_	Weighted	176	0.108587	0.030967	0.000454
15388_24_	finngen_R	outcome	15388_24_	Inverse va	176	0.108086	0.019236	1.92E-08
15388_24_	finngen_R	outcome	15388_24_	Simple mc	176	0.054903	0.054818	0.317951
15388_24_	finngen_R	outcome	15388_24_	Weighted	176	0.103029	0.029912	0.000716
15391_114	finngen_R	outcome	15391_114	MR Egger	25	-0.15083	0.185395	0.424237
15391_114	finngen_R	outcome	15391_114	Weighted	25	-0.0255	0.126908	0.840778
15391_114	finngen_R	outcome	15391_114	Inverse va	25	-0.07508	0.091439	0.411614
15391_114	finngen_R	outcome	15391_114	Simple mc	25	-0.00413	0.248605	0.986869
15391_114	finngen_R	outcome	15391_114	Weighted	25	-0.01841	0.135787	0.893291
15395_15_	finngen_R	outcome	15395_15_	MR Egger	132	-0.04311	0.041061	0.295759
15395_15_	finngen_R	outcome	15395_15_	Weighted	132	-0.01579	0.043196	0.714764
15395_15_	finngen_R	outcome	15395_15_	Inverse va	132	-0.02864	0.028087	0.307901
15395_15_	finngen_R	outcome	15395_15_	Simple mc	132	-0.06774	0.088628	0.446063
15395_15_	finngen_R	outcome	15395_15_	Weighted	132	-0.03275	0.037661	0.386086
15398_2_(	finngen_R	outcome	15398_2_(	Wald ratic	1	-0.0328	0.610812	0.957178
15414_316	finngen_R	outcome	15414_316	Inverse va	2	-0.20508	0.7913	0.795501
15417_3_S	finngen_R	outcome	15417_3_S	MR Egger	5	-0.01174	0.481624	0.982091
15417_3_S	finngen_R	outcome	15417_3_S	Weighted	5	0.183768	0.245546	0.454214
15417_3_S	finngen_R	outcome	15417_3_S	Inverse va	5	0.165332	0.223373	0.459204
15417_3_S	finngen_R	outcome	15417_3_S	Simple mc	5	0.155557	0.360235	0.68813
15417_3_S	finngen_R	outcome	15417_3_S	Weighted	5	0.187074	0.264693	0.518713
15427_35_	finngen_R	outcome	15427_35_	Inverse va	2	-0.31385	0.620296	0.612883
15435_4_F	finngen_R	outcome	15435_4_F	MR Egger	10	-0.17102	0.318008	0.605353
15435_4_F	finngen_R	outcome	15435_4_F	Weighted	10	-0.1151	0.200983	0.566867
15435_4_F	finngen_R	outcome	15435_4_F	Inverse va	10	-0.03396	0.153477	0.824903
15435_4_F	finngen_R	outcome	15435_4_F	Simple mc	10	0.151888	0.305465	0.630942
15435_4_F	finngen_R	outcome	15435_4_F	Weighted	10	-0.21546	0.248257	0.408009
15440_57_	finngen_R	outcome	15440_57_	MR Egger	5	1.880771	1.326096	0.251143
15440_57_	finngen_R	outcome	15440_57_	Weighted	5	0.491299	0.340919	0.149556
15440_57_	finngen_R	outcome	15440_57_	Inverse va	5	0.369516	0.275225	0.179403
15440_57_	finngen_R	outcome	15440_57_	Simple mc	5	0.684126	0.472029	0.220838
15440_57_	finngen_R	outcome	15440_57_	Weighted	5	0.467806	0.395561	0.302446
15444_45_	finngen_R	outcome	15444_45_	MR Egger	13	-0.04647	0.206732	0.826266
15444_45_	finngen_R	outcome	15444_45_	Weighted	13	-0.07245	0.179749	0.686886
15444_45_	finngen_R	outcome	15444_45_	Inverse va	13	0.008317	0.135153	0.950931
15444_45_	finngen_R	outcome	15444_45_	Simple mc	13	0.009741	0.282604	0.973071
15444_45_	finngen_R	outcome	15444_45_	Weighted	13	-0.08558	0.189911	0.660295
15447_45_	finngen_R	outcome	15447_45_	MR Egger	33	-0.04762	0.161856	0.770556
15447_45_	finngen_R	outcome	15447_45_	Weighted	33	0.021208	0.140176	0.879745
15447_45_	finngen_R	outcome	15447_45_	Inverse va	33	-0.11011	0.091673	0.229706
15447_45_	finngen_R	outcome	15447_45_	Simple mc	33	0.034916	0.236661	0.883634
15447_45_	finngen_R	outcome	15447_45_	Weighted	33	0.020834	0.153284	0.892736
15452_5_N	finngen_R	outcome	15452_5_N	MR Egger	46	-0.21903	0.093641	0.023942
15452_5_N	finngen_R	outcome	15452_5_N	Weighted	46	-0.11278	0.076709	0.14149

15452_5_N	finngen_R	outcome	15452_5_N	Inverse va	46	-0.07559	0.051996	0.146021
15452_5_N	finngen_R	outcome	15452_5_N	Simple mc	46	-0.13242	0.132717	0.32373
15452_5_N	finngen_R	outcome	15452_5_N	Weighted	46	-0.10807	0.077319	0.169055
15453_3_A	finngen_R	outcome	15453_3_A	MR Egger	6	0.311002	0.839391	0.729789
15453_3_A	finngen_R	outcome	15453_3_A	Weighted	6	0.065531	0.284904	0.818082
15453_3_A	finngen_R	outcome	15453_3_A	Inverse va	6	-0.0807	0.244696	0.741542
15453_3_A	finngen_R	outcome	15453_3_A	Simple mc	6	-0.01209	0.45629	0.979889
15453_3_A	finngen_R	outcome	15453_3_A	Weighted	6	0.051157	0.316869	0.878065
15462_28_	finngen_R	outcome	15462_28_	MR Egger	31	-0.22504	0.133552	0.102716
15462_28_	finngen_R	outcome	15462_28_	Weighted	31	-0.04865	0.109382	0.656479
15462_28_	finngen_R	outcome	15462_28_	Inverse va	31	0.06798	0.090874	0.454421
15462_28_	finngen_R	outcome	15462_28_	Simple mc	31	-0.06537	0.218517	0.766869
15462_28_	finngen_R	outcome	15462_28_	Weighted	31	-0.10737	0.118932	0.373811
15466_30_	finngen_R	outcome	15466_30_	MR Egger	5	-0.34293	1.720467	0.854754
15466_30_	finngen_R	outcome	15466_30_	Weighted	5	-0.55689	0.35474	0.116448
15466_30_	finngen_R	outcome	15466_30_	Inverse va	5	-0.6571	0.30149	0.029293
15466_30_	finngen_R	outcome	15466_30_	Simple mc	5	-0.51207	0.476051	0.34263
15466_30_	finngen_R	outcome	15466_30_	Weighted	5	-0.4504	0.451331	0.37479
15468_14_	finngen_R	outcome	15468_14_	MR Egger	61	0.060839	0.066985	0.367439
15468_14_	finngen_R	outcome	15468_14_	Weighted	61	0.061416	0.056113	0.273734
15468_14_	finngen_R	outcome	15468_14_	Inverse va	61	0.026803	0.040183	0.504759
15468_14_	finngen_R	outcome	15468_14_	Simple mc	61	0.055305	0.088076	0.532439
15468_14_	finngen_R	outcome	15468_14_	Weighted	61	0.049586	0.04906	0.316207
15471_29_	finngen_R	outcome	15471_29_	MR Egger	149	-0.099	0.043017	0.022769
15471_29_	finngen_R	outcome	15471_29_	Weighted	149	-0.02341	0.042245	0.579477
15471_29_	finngen_R	outcome	15471_29_	Inverse va	149	-0.0654	0.025538	0.010446
15471_29_	finngen_R	outcome	15471_29_	Simple mc	149	-0.22354	0.089634	0.013733
15471_29_	finngen_R	outcome	15471_29_	Weighted	149	-0.07195	0.042824	0.095031
15475_4_F	finngen_R	outcome	15475_4_F	MR Egger	78	0.11122	0.078806	0.162235
15475_4_F	finngen_R	outcome	15475_4_F	Weighted	78	0.092849	0.069695	0.182787
15475_4_F	finngen_R	outcome	15475_4_F	Inverse va	78	0.119351	0.043399	0.005958
15475_4_F	finngen_R	outcome	15475_4_F	Simple mc	78	0.181442	0.130876	0.169636
15475_4_F	finngen_R	outcome	15475_4_F	Weighted	78	0.11768	0.065078	0.074467
15476_6_F	finngen_R	outcome	15476_6_F	MR Egger	86	-0.07892	0.057069	0.170348
15476_6_F	finngen_R	outcome	15476_6_F	Weighted	86	-0.13361	0.053957	0.013278
15476_6_F	finngen_R	outcome	15476_6_F	Inverse va	86	-0.08846	0.034212	0.00972
15476_6_F	finngen_R	outcome	15476_6_F	Simple mc	86	-0.13116	0.096403	0.177263
15476_6_F	finngen_R	outcome	15476_6_F	Weighted	86	-0.11264	0.046499	0.017537
15481_45_	finngen_R	outcome	15481_45_	Wald ratic	1	-0.24031	0.384131	0.531589
15483_377	finngen_R	outcome	15483_377	MR Egger	29	0.1128	0.168603	0.509158
15483_377	finngen_R	outcome	15483_377	Weighted	29	-0.04335	0.114685	0.705461
15483_377	finngen_R	outcome	15483_377	Inverse va	29	-0.07312	0.078715	0.352941
15483_377	finngen_R	outcome	15483_377	Simple mc	29	0.043119	0.183493	0.815925
15483_377	finngen_R	outcome	15483_377	Weighted	29	-0.01384	0.115078	0.905125
15486_126	finngen_R	outcome	15486_126	MR Egger	191	-0.02024	0.033094	0.541518
15486_126	finngen_R	outcome	15486_126	Weighted	191	-0.02252	0.034118	0.509134
15486_126	finngen_R	outcome	15486_126	Inverse va	191	-0.0044	0.021344	0.83652
15486_126	finngen_R	outcome	15486_126	Simple mc	191	-0.05751	0.064284	0.372107
15486_126	finngen_R	outcome	15486_126	Weighted	191	-0.00369	0.03155	0.907008
15487_164	finngen_R	outcome	15487_164	Wald ratic	1	-0.29814	0.682436	0.662202
15491_20_	finngen_R	outcome	15491_20_	Inverse va	2	-0.18429	0.320049	0.564746
15494_11_	finngen_R	outcome	15494_11_	MR Egger	4	0.066268	0.857711	0.945449
15494_11_	finngen_R	outcome	15494_11_	Weighted	4	-0.53639	0.391573	0.170739
15494_11_	finngen_R	outcome	15494_11_	Inverse va	4	-0.46092	0.322887	0.153438
15494_11_	finngen_R	outcome	15494_11_	Simple mc	4	-0.55253	0.49867	0.348707
15494_11_	finngen_R	outcome	15494_11_	Weighted	4	-0.57103	0.450201	0.294141
15495_9_F	finngen_R	outcome	15495_9_F	MR Egger	77	0.020588	0.034932	0.557383
15495_9_F	finngen_R	outcome	15495_9_F	Weighted	77	0.020497	0.034333	0.550498

15495_9_F	finngen_R	outcome	15495_9_F	Inverse va	77	0.01553	0.023942	0.516585
15495_9_F	finngen_R	outcome	15495_9_F	Simple mc	77	0.03131	0.050179	0.534513
15495_9_F	finngen_R	outcome	15495_9_F	Weighted	77	0.0237	0.032387	0.466554
15499_11_	finngen_R	outcome	15499_11_	MR Egger	57	0.049138	0.056603	0.389108
15499_11_	finngen_R	outcome	15499_11_	Weighted	57	0.017696	0.056491	0.754087
15499_11_	finngen_R	outcome	15499_11_	Inverse va	57	-0.01328	0.04098	0.745933
15499_11_	finngen_R	outcome	15499_11_	Simple mc	57	-0.02715	0.109552	0.805189
15499_11_	finngen_R	outcome	15499_11_	Weighted	57	-0.00076	0.048144	0.987425
15503_15_	finngen_R	outcome	15503_15_	MR Egger	16	0.137982	0.312688	0.665751
15503_15_	finngen_R	outcome	15503_15_	Weighted	16	0.153896	0.168854	0.362075
15503_15_	finngen_R	outcome	15503_15_	Inverse va	16	0.240389	0.125624	0.055676
15503_15_	finngen_R	outcome	15503_15_	Simple mc	16	0.257662	0.241603	0.303078
15503_15_	finngen_R	outcome	15503_15_	Weighted	16	0.151593	0.158722	0.354675
15503_20_	finngen_R	outcome	15503_20_	MR Egger	115	0.031596	0.041002	0.44256
15503_20_	finngen_R	outcome	15503_20_	Weighted	115	0.014493	0.042842	0.735154
15503_20_	finngen_R	outcome	15503_20_	Inverse va	115	0.019041	0.024736	0.441428
15503_20_	finngen_R	outcome	15503_20_	Simple mc	115	0.006079	0.064929	0.925573
15503_20_	finngen_R	outcome	15503_20_	Weighted	115	0.027964	0.034468	0.418877
15509_2_N	finngen_R	outcome	15509_2_N	MR Egger	50	0.010282	0.064809	0.874615
15509_2_N	finngen_R	outcome	15509_2_N	Weighted	50	0.01724	0.053299	0.746347
15509_2_N	finngen_R	outcome	15509_2_N	Inverse va	50	0.025937	0.047978	0.58878
15509_2_N	finngen_R	outcome	15509_2_N	Simple mc	50	-0.00669	0.111727	0.952486
15509_2_N	finngen_R	outcome	15509_2_N	Weighted	50	0.018686	0.04578	0.684931
15513_108	finngen_R	outcome	15513_108	MR Egger	4	-0.66373	0.596005	0.38133
15513_108	finngen_R	outcome	15513_108	Weighted	4	-0.16192	0.342616	0.636503
15513_108	finngen_R	outcome	15513_108	Inverse va	4	-0.16191	0.296609	0.585159
15513_108	finngen_R	outcome	15513_108	Simple mc	4	-0.02499	0.489657	0.962498
15513_108	finngen_R	outcome	15513_108	Weighted	4	-0.16076	0.434136	0.735763
15514_26_	finngen_R	outcome	15514_26_	MR Egger	52	-0.12764	0.075188	0.095807
15514_26_	finngen_R	outcome	15514_26_	Weighted	52	-0.08595	0.062976	0.172333
15514_26_	finngen_R	outcome	15514_26_	Inverse va	52	-0.03994	0.04305	0.353563
15514_26_	finngen_R	outcome	15514_26_	Simple mc	52	0.061054	0.120226	0.613767
15514_26_	finngen_R	outcome	15514_26_	Weighted	52	-0.04991	0.060078	0.40996
15515_2_S	finngen_R	outcome	15515_2_S	MR Egger	51	0.092383	0.112549	0.415723
15515_2_S	finngen_R	outcome	15515_2_S	Weighted	51	0.07492	0.097116	0.440442
15515_2_S	finngen_R	outcome	15515_2_S	Inverse va	51	0.065547	0.064255	0.307681
15515_2_S	finngen_R	outcome	15515_2_S	Simple mc	51	-0.04811	0.18042	0.790821
15515_2_S	finngen_R	outcome	15515_2_S	Weighted	51	0.053186	0.097143	0.586473
15516_12_	finngen_R	outcome	15516_12_	MR Egger	93	-0.1419	0.037913	0.000318
15516_12_	finngen_R	outcome	15516_12_	Weighted	93	-0.07107	0.036877	0.053938
15516_12_	finngen_R	outcome	15516_12_	Inverse va	93	-0.05022	0.025036	0.044856
15516_12_	finngen_R	outcome	15516_12_	Simple mc	93	-0.05705	0.066245	0.391357
15516_12_	finngen_R	outcome	15516_12_	Weighted	93	-0.08904	0.036695	0.0172
15521_4_C	finngen_R	outcome	15521_4_C	MR Egger	20	0.034733	0.143549	0.811544
15521_4_C	finngen_R	outcome	15521_4_C	Weighted	20	0.066137	0.124134	0.594181
15521_4_C	finngen_R	outcome	15521_4_C	Inverse va	20	-0.02127	0.09567	0.824092
15521_4_C	finngen_R	outcome	15521_4_C	Simple mc	20	-0.26288	0.210914	0.227763
15521_4_C	finngen_R	outcome	15521_4_C	Weighted	20	-0.0423	0.14448	0.772868
15525_294	finngen_R	outcome	15525_294	MR Egger	5	1.538553	0.587303	0.079023
15525_294	finngen_R	outcome	15525_294	Weighted	5	0.139169	0.248704	0.575767
15525_294	finngen_R	outcome	15525_294	Inverse va	5	-0.03399	0.30296	0.91067
15525_294	finngen_R	outcome	15525_294	Simple mc	5	-0.03882	0.389815	0.925466
15525_294	finngen_R	outcome	15525_294	Weighted	5	0.174762	0.233014	0.494956
15526_33_	finngen_R	outcome	15526_33_	MR Egger	11	0.047347	0.634563	0.942154
15526_33_	finngen_R	outcome	15526_33_	Weighted	11	0.020067	0.219191	0.927054
15526_33_	finngen_R	outcome	15526_33_	Inverse va	11	-0.01334	0.204122	0.947887
15526_33_	finngen_R	outcome	15526_33_	Simple mc	11	-0.05527	0.306493	0.860499
15526_33_	finngen_R	outcome	15526_33_	Weighted	11	0.109191	0.268708	0.69304

15530_33_	finngen_R	outcome	15530_33_	MR Egger	7	0.021431	0.682766	0.976174
15530_33_	finngen_R	outcome	15530_33_	Weighted	7	-0.21477	0.266726	0.420708
15530_33_	finngen_R	outcome	15530_33_	Inverse va	7	-0.17254	0.20234	0.393826
15530_33_	finngen_R	outcome	15530_33_	Simple mc	7	-0.2135	0.398435	0.611331
15530_33_	finngen_R	outcome	15530_33_	Weighted	7	-0.19652	0.278006	0.506143
15533_97_	finngen_R	outcome	15533_97_	MR Egger	27	-0.08524	0.164925	0.609819
15533_97_	finngen_R	outcome	15533_97_	Weighted	27	-0.00982	0.13373	0.941468
15533_97_	finngen_R	outcome	15533_97_	Inverse va	27	-0.09359	0.089724	0.29692
15533_97_	finngen_R	outcome	15533_97_	Simple mc	27	-0.10021	0.214153	0.643738
15533_97_	finngen_R	outcome	15533_97_	Weighted	27	0.013831	0.134204	0.918704
15535_3_F	finngen_R	outcome	15535_3_F	MR Egger	9	-0.10764	0.230082	0.654115
15535_3_F	finngen_R	outcome	15535_3_F	Weighted	9	-0.0443	0.160535	0.782599
15535_3_F	finngen_R	outcome	15535_3_F	Inverse va	9	-0.05919	0.14022	0.672924
15535_3_F	finngen_R	outcome	15535_3_F	Simple mc	9	-0.07631	0.279136	0.791491
15535_3_F	finngen_R	outcome	15535_3_F	Weighted	9	-0.02174	0.171675	0.90236
15540_6_\	finngen_R	outcome	15540_6_\	MR Egger	5	0.098808	0.874301	0.917158
15540_6_\	finngen_R	outcome	15540_6_\	Weighted	5	-0.0949	0.330314	0.773888
15540_6_\	finngen_R	outcome	15540_6_\	Inverse va	5	-0.20548	0.272151	0.450228
15540_6_\	finngen_R	outcome	15540_6_\	Simple mc	5	-0.03151	0.468599	0.949619
15540_6_\	finngen_R	outcome	15540_6_\	Weighted	5	0.001549	0.401404	0.997106
15544_25_	finngen_R	outcome	15544_25_	MR Egger	43	-0.0728	0.133793	0.589279
15544_25_	finngen_R	outcome	15544_25_	Weighted	43	-0.09313	0.093946	0.321533
15544_25_	finngen_R	outcome	15544_25_	Inverse va	43	-0.04347	0.063884	0.496242
15544_25_	finngen_R	outcome	15544_25_	Simple mc	43	-0.03321	0.160956	0.83755
15544_25_	finngen_R	outcome	15544_25_	Weighted	43	-0.1188	0.108514	0.279846
15556_49_	finngen_R	outcome	15556_49_	MR Egger	52	0.023953	0.089852	0.790889
15556_49_	finngen_R	outcome	15556_49_	Weighted	52	-0.00042	0.070396	0.995251
15556_49_	finngen_R	outcome	15556_49_	Inverse va	52	0.044666	0.047057	0.342522
15556_49_	finngen_R	outcome	15556_49_	Simple mc	52	0.056028	0.101985	0.585145
15556_49_	finngen_R	outcome	15556_49_	Weighted	52	0.006097	0.070913	0.931823
15558_63_	finngen_R	outcome	15558_63_	MR Egger	88	-0.08103	0.078389	0.304182
15558_63_	finngen_R	outcome	15558_63_	Weighted	88	-0.08973	0.061495	0.144536
15558_63_	finngen_R	outcome	15558_63_	Inverse va	88	-0.00914	0.04657	0.84439
15558_63_	finngen_R	outcome	15558_63_	Simple mc	88	0.044286	0.116264	0.704196
15558_63_	finngen_R	outcome	15558_63_	Weighted	88	-0.10539	0.062423	0.094928
15559_5_/	finngen_R	outcome	15559_5_/	MR Egger	16	0.105625	0.252678	0.682271
15559_5_/	finngen_R	outcome	15559_5_/	Weighted	16	0.01273	0.138547	0.926791
15559_5_/	finngen_R	outcome	15559_5_/	Inverse va	16	-0.02945	0.117646	0.802338
15559_5_/	finngen_R	outcome	15559_5_/	Simple mc	16	-0.27903	0.212357	0.208601
15559_5_/	finngen_R	outcome	15559_5_/	Weighted	16	0.008866	0.133541	0.947946
15560_52_	finngen_R	outcome	15560_52_	MR Egger	108	0.011864	0.036932	0.748663
15560_52_	finngen_R	outcome	15560_52_	Weighted	108	0.020792	0.026623	0.434807
15560_52_	finngen_R	outcome	15560_52_	Inverse va	108	0.027399	0.018201	0.132223
15560_52_	finngen_R	outcome	15560_52_	Simple mc	108	-0.0041	0.047146	0.930797
15560_52_	finngen_R	outcome	15560_52_	Weighted	108	0.017769	0.032058	0.580558
15562_24_	finngen_R	outcome	15562_24_	MR Egger	9	-0.43765	0.519045	0.426993
15562_24_	finngen_R	outcome	15562_24_	Weighted	9	-0.52148	0.185222	0.004872
15562_24_	finngen_R	outcome	15562_24_	Inverse va	9	-0.46357	0.145012	0.00139
15562_24_	finngen_R	outcome	15562_24_	Simple mc	9	-0.50376	0.238619	0.067752
15562_24_	finngen_R	outcome	15562_24_	Weighted	9	-0.52967	0.204455	0.032082
15565_102	finngen_R	outcome	15565_102	MR Egger	5	0.781073	0.777628	0.389174
15565_102	finngen_R	outcome	15565_102	Weighted	5	-0.34941	0.272975	0.20054
15565_102	finngen_R	outcome	15565_102	Inverse va	5	-0.48841	0.244301	0.045584
15565_102	finngen_R	outcome	15565_102	Simple mc	5	-0.61955	0.60048	0.360485
15565_102	finngen_R	outcome	15565_102	Weighted	5	-0.31843	0.26926	0.302452
15570_99_	finngen_R	outcome	15570_99_	MR Egger	34	-0.22681	0.201584	0.268905
15570_99_	finngen_R	outcome	15570_99_	Weighted	34	-0.09739	0.125349	0.437186
15570_99_	finngen_R	outcome	15570_99_	Inverse va	34	-0.00351	0.087074	0.967806

15570_99_	finngen_R	outcome	15570_99_	Simple mc	34	-0.14335	0.214574	0.508727
15570_99_	finngen_R	outcome	15570_99_	Weighted	34	-0.12239	0.146337	0.408983
15573_11(	finngen_R	outcome	15573_11(	MR Egger	9	-0.03061	0.235901	0.900421
15573_11(	finngen_R	outcome	15573_11(	Weighted	9	0.081992	0.173879	0.63725
15573_11(	finngen_R	outcome	15573_11(	Inverse va	9	0.072627	0.14536	0.617331
15573_11(	finngen_R	outcome	15573_11(	Simple mc	9	0.120539	0.263574	0.659599
15573_11(	finngen_R	outcome	15573_11(	Weighted	9	0.087533	0.186127	0.650706
15576_15ε	finngen_R	outcome	15576_15ε	MR Egger	41	-0.11673	0.120442	0.338437
15576_15ε	finngen_R	outcome	15576_15ε	Weighted	41	-0.14585	0.097091	0.13304
15576_15ε	finngen_R	outcome	15576_15ε	Inverse va	41	-0.14454	0.072318	0.045652
15576_15ε	finngen_R	outcome	15576_15ε	Simple mc	41	-0.09804	0.169845	0.567025
15576_15ε	finngen_R	outcome	15576_15ε	Weighted	41	-0.19735	0.09307	0.040221
15579_26_	finngen_R	outcome	15579_26_	Inverse va	2	-0.93614	0.453089	0.038816
15582_25_	finngen_R	outcome	15582_25_	MR Egger	60	0.058316	0.113186	0.608352
15582_25_	finngen_R	outcome	15582_25_	Weighted	60	0.032287	0.092189	0.726167
15582_25_	finngen_R	outcome	15582_25_	Inverse va	60	0.019869	0.062741	0.751491
15582_25_	finngen_R	outcome	15582_25_	Simple mc	60	0.331689	0.203258	0.108034
15582_25_	finngen_R	outcome	15582_25_	Weighted	60	0.016362	0.086915	0.85132
15584_9_(	finngen_R	outcome	15584_9_(	MR Egger	87	0.062947	0.036182	0.085523
15584_9_(	finngen_R	outcome	15584_9_(	Weighted	87	0.024719	0.031839	0.437518
15584_9_(	finngen_R	outcome	15584_9_(	Inverse va	87	0.013383	0.021639	0.536268
15584_9_(	finngen_R	outcome	15584_9_(	Simple mc	87	0.05543	0.048308	0.254381
15584_9_(	finngen_R	outcome	15584_9_(	Weighted	87	0.037925	0.030471	0.216648
15585_30ʼ	finngen_R	outcome	15585_30ʼ	MR Egger	4	0.137369	1.161913	0.916692
15585_30ʼ	finngen_R	outcome	15585_30ʼ	Weighted	4	0.563378	0.368935	0.126752
15585_30ʼ	finngen_R	outcome	15585_30ʼ	Inverse va	4	0.459687	0.317196	0.147276
15585_30ʼ	finngen_R	outcome	15585_30ʼ	Simple mc	4	0.560766	0.485647	0.331849
15585_30ʼ	finngen_R	outcome	15585_30ʼ	Weighted	4	0.570509	0.443712	0.288784
15587_20_	finngen_R	outcome	15587_20_	MR Egger	4	-0.21303	0.487002	0.704498
15587_20_	finngen_R	outcome	15587_20_	Weighted	4	-0.05277	0.311346	0.86541
15587_20_	finngen_R	outcome	15587_20_	Inverse va	4	0.075103	0.279405	0.788085
15587_20_	finngen_R	outcome	15587_20_	Simple mc	4	-0.0343	0.457264	0.944934
15587_20_	finngen_R	outcome	15587_20_	Weighted	4	-0.09224	0.351259	0.809868
15589_1_(	finngen_R	outcome	15589_1_(	MR Egger	8	0.308415	0.763976	0.700429
15589_1_(	finngen_R	outcome	15589_1_(	Weighted	8	0.174897	0.247259	0.479353
15589_1_(	finngen_R	outcome	15589_1_(	Inverse va	8	0.21856	0.200365	0.275357
15589_1_(	finngen_R	outcome	15589_1_(	Simple mc	8	0.21347	0.324769	0.531999
15589_1_(	finngen_R	outcome	15589_1_(	Weighted	8	0.181905	0.24322	0.478886
15594_47_	finngen_R	outcome	15594_47_	MR Egger	8	-0.10514	0.593475	0.86521
15594_47_	finngen_R	outcome	15594_47_	Weighted	8	0.176694	0.216031	0.413407
15594_47_	finngen_R	outcome	15594_47_	Inverse va	8	0.195506	0.180775	0.27948
15594_47_	finngen_R	outcome	15594_47_	Simple mc	8	0.151944	0.304797	0.633405
15594_47_	finngen_R	outcome	15594_47_	Weighted	8	0.173707	0.286944	0.564038
15602_43_	finngen_R	outcome	15602_43_	MR Egger	118	-0.03295	0.040101	0.412886
15602_43_	finngen_R	outcome	15602_43_	Weighted	118	-0.02794	0.039411	0.478396
15602_43_	finngen_R	outcome	15602_43_	Inverse va	118	-0.00865	0.024164	0.720276
15602_43_	finngen_R	outcome	15602_43_	Simple mc	118	-0.07443	0.070334	0.292146
15602_43_	finngen_R	outcome	15602_43_	Weighted	118	-0.02717	0.03508	0.440119
15603_20_	finngen_R	outcome	15603_20_	Inverse va	2	-0.27556	0.350915	0.432301
15604_18_	finngen_R	outcome	15604_18_	MR Egger	10	0.608906	0.329028	0.10138
15604_18_	finngen_R	outcome	15604_18_	Weighted	10	0.539616	0.188041	0.004109
15604_18_	finngen_R	outcome	15604_18_	Inverse va	10	0.553985	0.150652	0.000236
15604_18_	finngen_R	outcome	15604_18_	Simple mc	10	0.463538	0.276615	0.128106
15604_18_	finngen_R	outcome	15604_18_	Weighted	10	0.529887	0.218614	0.038368
15607_56_	finngen_R	outcome	15607_56_	MR Egger	6	-0.10855	0.350663	0.772357
15607_56_	finngen_R	outcome	15607_56_	Weighted	6	-0.03376	0.239197	0.887752
15607_56_	finngen_R	outcome	15607_56_	Inverse va	6	0.011241	0.206129	0.956509
15607_56_	finngen_R	outcome	15607_56_	Simple mc	6	-0.09188	0.353998	0.805568

15607_56_	finngen_R	outcome	15607_56_	Weighted	6	-0.03162	0.261455	0.908459
15610_72_	finngen_R	outcome	15610_72_	MR Egger	8	0.494799	0.386617	0.247864
15610_72_	finngen_R	outcome	15610_72_	Weighted	8	0.074743	0.260697	0.77434
15610_72_	finngen_R	outcome	15610_72_	Inverse va	8	0.062995	0.262153	0.810099
15610_72_	finngen_R	outcome	15610_72_	Simple mc	8	0.066434	0.333388	0.847716
15610_72_	finngen_R	outcome	15610_72_	Weighted	8	0.057754	0.269199	0.836242
15613_16_	finngen_R	outcome	15613_16_	MR Egger	5	0.905487	2.263923	0.715959
15613_16_	finngen_R	outcome	15613_16_	Weighted	5	0.555844	0.313905	0.076604
15613_16_	finngen_R	outcome	15613_16_	Inverse va	5	0.649774	0.357552	0.069173
15613_16_	finngen_R	outcome	15613_16_	Simple mc	5	0.831404	0.516058	0.182456
15613_16_	finngen_R	outcome	15613_16_	Weighted	5	0.613681	0.346877	0.151587
15614_168	finngen_R	outcome	15614_168	MR Egger	70	0.082602	0.073774	0.266792
15614_168	finngen_R	outcome	15614_168	Weighted	70	0.035968	0.070895	0.611915
15614_168	finngen_R	outcome	15614_168	Inverse va	70	0.040769	0.04736	0.389338
15614_168	finngen_R	outcome	15614_168	Simple mc	70	0.032927	0.119452	0.78364
15614_168	finngen_R	outcome	15614_168	Weighted	70	0.052977	0.064675	0.415537
15620_4_N	finngen_R	outcome	15620_4_N	MR Egger	8	-0.75503	0.534707	0.20764
15620_4_N	finngen_R	outcome	15620_4_N	Weighted	8	-0.39586	0.25366	0.118624
15620_4_N	finngen_R	outcome	15620_4_N	Inverse va	8	-0.27183	0.185909	0.143698
15620_4_N	finngen_R	outcome	15620_4_N	Simple mc	8	-0.46316	0.40983	0.29565
15620_4_N	finngen_R	outcome	15620_4_N	Weighted	8	-0.49123	0.257673	0.098274
15622_13_	finngen_R	outcome	15622_13_	MR Egger	14	0.36942	0.529035	0.498304
15622_13_	finngen_R	outcome	15622_13_	Weighted	14	-0.17337	0.24144	0.47272
15622_13_	finngen_R	outcome	15622_13_	Inverse va	14	0.112295	0.18684	0.547824
15622_13_	finngen_R	outcome	15622_13_	Simple mc	14	-0.38591	0.426916	0.382468
15622_13_	finngen_R	outcome	15622_13_	Weighted	14	-0.3667	0.392309	0.366966
15626_223	finngen_R	outcome	15626_223	MR Egger	26	0.159147	0.142126	0.273899
15626_223	finngen_R	outcome	15626_223	Weighted	26	0.063074	0.13725	0.645834
15626_223	finngen_R	outcome	15626_223	Inverse va	26	0.121453	0.085306	0.154523
15626_223	finngen_R	outcome	15626_223	Simple mc	26	0.209905	0.257113	0.421987
15626_223	finngen_R	outcome	15626_223	Weighted	26	0.089706	0.148765	0.551937
15633_6_F	finngen_R	outcome	15633_6_F	Inverse va	2	0.332022	0.37221	0.372378
15635_4_S	finngen_R	outcome	15635_4_S	MR Egger	64	0.111865	0.103435	0.283663
15635_4_S	finngen_R	outcome	15635_4_S	Weighted	64	0.067629	0.088947	0.44706
15635_4_S	finngen_R	outcome	15635_4_S	Inverse va	64	0.015671	0.054426	0.773402
15635_4_S	finngen_R	outcome	15635_4_S	Simple mc	64	-0.13681	0.17921	0.448066
15635_4_S	finngen_R	outcome	15635_4_S	Weighted	64	0.019589	0.096565	0.8399
15644_1_E	finngen_R	outcome	15644_1_E	MR Egger	71	-0.05708	0.065183	0.384229
15644_1_E	finngen_R	outcome	15644_1_E	Weighted	71	-0.0405	0.060569	0.503701
15644_1_E	finngen_R	outcome	15644_1_E	Inverse va	71	-0.03253	0.044485	0.464663
15644_1_E	finngen_R	outcome	15644_1_E	Simple mc	71	-0.05595	0.147346	0.705325
15644_1_E	finngen_R	outcome	15644_1_E	Weighted	71	-0.04655	0.056159	0.409934
15653_9_(	finngen_R	outcome	15653_9_(	MR Egger	28	-0.21789	0.174451	0.222792
15653_9_(	finngen_R	outcome	15653_9_(	Weighted	28	-0.12019	0.128361	0.349096
15653_9_(	finngen_R	outcome	15653_9_(	Inverse va	28	-0.12872	0.08879	0.147138
15653_9_(	finngen_R	outcome	15653_9_(	Simple mc	28	-0.32834	0.220412	0.1479
15653_9_(	finngen_R	outcome	15653_9_(	Weighted	28	-0.14716	0.132345	0.275965
15686_49_	finngen_R	outcome	15686_49_	MR Egger	113	0.039799	0.037731	0.293803
15686_49_	finngen_R	outcome	15686_49_	Weighted	113	-0.01462	0.030691	0.633859
15686_49_	finngen_R	outcome	15686_49_	Inverse va	113	-0.00568	0.018946	0.764149
15686_49_	finngen_R	outcome	15686_49_	Simple mc	113	-0.09335	0.056062	0.098685
15686_49_	finngen_R	outcome	15686_49_	Weighted	113	-0.03604	0.03647	0.325124
16035_8_F	finngen_R	outcome	16035_8_F	MR Egger	10	-0.64143	0.411717	0.15786
16035_8_F	finngen_R	outcome	16035_8_F	Weighted	10	-0.39546	0.219463	0.071552
16035_8_F	finngen_R	outcome	16035_8_F	Inverse va	10	-0.2802	0.174856	0.109052
16035_8_F	finngen_R	outcome	16035_8_F	Simple mc	10	-0.34532	0.345182	0.343257
16035_8_F	finngen_R	outcome	16035_8_F	Weighted	10	-0.40775	0.243279	0.128041
16049_43_	finngen_R	outcome	16049_43_	Wald ratic	1	0.22488	0.750083	0.764324

16055_3_(	finngen_R	outcome	16055_3_(	MR Egger	38	0.0558	0.061895	0.373303
16055_3_(	finngen_R	outcome	16055_3_(	Weighted	38	0.038362	0.058467	0.511742
16055_3_(	finngen_R	outcome	16055_3_(	Inverse va	38	-0.00353	0.042149	0.933246
16055_3_(	finngen_R	outcome	16055_3_(	Simple mc	38	-0.07654	0.105265	0.471722
16055_3_(	finngen_R	outcome	16055_3_(	Weighted	38	0.016868	0.052823	0.751271
16057_6_I	finngen_R	outcome	16057_6_I	MR Egger	62	-0.00258	0.076391	0.97314
16057_6_I	finngen_R	outcome	16057_6_I	Weighted	62	-0.00184	0.069128	0.978801
16057_6_I	finngen_R	outcome	16057_6_I	Inverse va	62	0.00859	0.047016	0.855023
16057_6_I	finngen_R	outcome	16057_6_I	Simple mc	62	-0.08637	0.135212	0.525341
16057_6_I	finngen_R	outcome	16057_6_I	Weighted	62	-0.0146	0.065145	0.823468
16060_99_	finngen_R	outcome	16060_99_	MR Egger	94	0.068961	0.058139	0.238623
16060_99_	finngen_R	outcome	16060_99_	Weighted	94	0.077956	0.049126	0.112548
16060_99_	finngen_R	outcome	16060_99_	Inverse va	94	0.066692	0.033454	0.046204
16060_99_	finngen_R	outcome	16060_99_	Simple mc	94	0.139319	0.088274	0.117901
16060_99_	finngen_R	outcome	16060_99_	Weighted	94	0.078921	0.048315	0.105749
16070_7_V	finngen_R	outcome	16070_7_V	Inverse va	2	-0.75378	0.475174	0.112665
16079_2_T	finngen_R	outcome	16079_2_T	MR Egger	10	-0.32748	0.526352	0.551147
16079_2_T	finngen_R	outcome	16079_2_T	Weighted	10	0.295878	0.205958	0.150833
16079_2_T	finngen_R	outcome	16079_2_T	Inverse va	10	0.20003	0.16756	0.232564
16079_2_T	finngen_R	outcome	16079_2_T	Simple mc	10	0.416315	0.295331	0.192248
16079_2_T	finngen_R	outcome	16079_2_T	Weighted	10	0.354988	0.266397	0.21543
16288_17_	finngen_R	outcome	16288_17_	MR Egger	22	-0.1901	0.210528	0.377286
16288_17_	finngen_R	outcome	16288_17_	Weighted	22	0.017914	0.155096	0.908048
16288_17_	finngen_R	outcome	16288_17_	Inverse va	22	-0.01725	0.10496	0.869452
16288_17_	finngen_R	outcome	16288_17_	Simple mc	22	0.015149	0.251638	0.952564
16288_17_	finngen_R	outcome	16288_17_	Weighted	22	#######	0.22206	0.999697
16296_43_	finngen_R	outcome	16296_43_	Inverse va	2	0.057188	0.485173	0.906169
16302_11_	finngen_R	outcome	16302_11_	MR Egger	5	0.356769	0.865445	0.707853
16302_11_	finngen_R	outcome	16302_11_	Weighted	5	0.44415	0.325637	0.172586
16302_11_	finngen_R	outcome	16302_11_	Inverse va	5	0.358486	0.274519	0.191596
16302_11_	finngen_R	outcome	16302_11_	Simple mc	5	0.489659	0.434682	0.32297
16302_11_	finngen_R	outcome	16302_11_	Weighted	5	0.519876	0.365786	0.22829
16304_6_L	finngen_R	outcome	16304_6_L	Inverse va	2	0.095045	0.423674	0.822496
16318_12_	finngen_R	outcome	16318_12_	MR Egger	14	-0.10044	0.236536	0.678608
16318_12_	finngen_R	outcome	16318_12_	Weighted	14	-0.03396	0.153639	0.825049
16318_12_	finngen_R	outcome	16318_12_	Inverse va	14	-0.02122	0.117099	0.856199
16318_12_	finngen_R	outcome	16318_12_	Simple mc	14	-0.12846	0.277461	0.651033
16318_12_	finngen_R	outcome	16318_12_	Weighted	14	-0.06959	0.160121	0.670976
16323_8_N	finngen_R	outcome	16323_8_N	MR Egger	21	-0.16612	0.225502	0.470316
16323_8_N	finngen_R	outcome	16323_8_N	Weighted	21	0.102877	0.139521	0.460904
16323_8_N	finngen_R	outcome	16323_8_N	Inverse va	21	0.183528	0.100878	0.068865
16323_8_N	finngen_R	outcome	16323_8_N	Simple mc	21	0.090503	0.221991	0.687833
16323_8_N	finngen_R	outcome	16323_8_N	Weighted	21	0.021879	0.166146	0.896551
16324_38_	finngen_R	outcome	16324_38_	MR Egger	8	1.102188	0.595697	0.113751
16324_38_	finngen_R	outcome	16324_38_	Weighted	8	0.814642	0.2768	0.00325
16324_38_	finngen_R	outcome	16324_38_	Inverse va	8	0.782341	0.221857	0.000421
16324_38_	finngen_R	outcome	16324_38_	Simple mc	8	0.876643	0.393072	0.06095
16324_38_	finngen_R	outcome	16324_38_	Weighted	8	0.808595	0.302742	0.031958
16558_2_N	finngen_R	outcome	16558_2_N	MR Egger	47	-0.00324	0.127993	0.979936
16558_2_N	finngen_R	outcome	16558_2_N	Weighted	47	0.073362	0.088145	0.405248
16558_2_N	finngen_R	outcome	16558_2_N	Inverse va	47	0.05387	0.059493	0.365205
16558_2_N	finngen_R	outcome	16558_2_N	Simple mc	47	-0.00783	0.156451	0.960287
16558_2_N	finngen_R	outcome	16558_2_N	Weighted	47	0.052801	0.100181	0.600689
16561_9_A	finngen_R	outcome	16561_9_A	MR Egger	19	-0.09176	0.111967	0.423825
16561_9_A	finngen_R	outcome	16561_9_A	Weighted	19	-0.10982	0.113258	0.332223
16561_9_A	finngen_R	outcome	16561_9_A	Inverse va	19	-0.13655	0.079182	0.084618
16561_9_A	finngen_R	outcome	16561_9_A	Simple mc	19	-0.29618	0.171277	0.100874
16561_9_A	finngen_R	outcome	16561_9_A	Weighted	19	-0.13533	0.111233	0.239452

16605_2_(	finngen_R	outcome	16605_2_(	MR Egger	15	0.290354	0.234544	0.237626
16605_2_(	finngen_R	outcome	16605_2_(	Weighted	15	0.324446	0.154245	0.035427
16605_2_(	finngen_R	outcome	16605_2_(	Inverse va	15	0.310338	0.117982	0.008529
16605_2_(	finngen_R	outcome	16605_2_(	Simple mc	15	0.200919	0.248583	0.432463
16605_2_(	finngen_R	outcome	16605_2_(	Weighted	15	0.314308	0.169905	0.085546
16606_85_	finngen_R	outcome	16606_85_	Inverse va	2	-0.69586	0.358033	0.051948
16607_78_	finngen_R	outcome	16607_78_	MR Egger	14	-0.08222	0.156069	0.607916
16607_78_	finngen_R	outcome	16607_78_	Weighted	14	-0.11956	0.140091	0.393411
16607_78_	finngen_R	outcome	16607_78_	Inverse va	14	-0.0141	0.104592	0.892756
16607_78_	finngen_R	outcome	16607_78_	Simple mc	14	0.026222	0.278888	0.926525
16607_78_	finngen_R	outcome	16607_78_	Weighted	14	-0.12564	0.147732	0.410456
16609_10(	finngen_R	outcome	16609_10(	MR Egger	53	0.146983	0.065164	0.028414
16609_10(	finngen_R	outcome	16609_10(	Weighted	53	0.127023	0.071474	0.075539
16609_10(	finngen_R	outcome	16609_10(	Inverse va	53	0.082934	0.045879	0.070658
16609_10(	finngen_R	outcome	16609_10(	Simple mc	53	0.06196	0.129592	0.63457
16609_10(	finngen_R	outcome	16609_10(	Weighted	53	0.125984	0.064325	0.055537
16614_27_	finngen_R	outcome	16614_27_	MR Egger	7	-0.07442	0.221551	0.750578
16614_27_	finngen_R	outcome	16614_27_	Weighted	7	-0.01399	0.200009	0.944233
16614_27_	finngen_R	outcome	16614_27_	Inverse va	7	-0.08593	0.156243	0.582341
16614_27_	finngen_R	outcome	16614_27_	Simple mc	7	-0.13059	0.290196	0.668499
16614_27_	finngen_R	outcome	16614_27_	Weighted	7	0.004605	0.20772	0.983031
16620_26_	finngen_R	outcome	16620_26_	MR Egger	119	-0.0217	0.041994	0.606314
16620_26_	finngen_R	outcome	16620_26_	Weighted	119	0.018907	0.032694	0.563069
16620_26_	finngen_R	outcome	16620_26_	Inverse va	119	-0.00315	0.021813	0.885051
16620_26_	finngen_R	outcome	16620_26_	Simple mc	119	-0.00266	0.051214	0.95866
16620_26_	finngen_R	outcome	16620_26_	Weighted	119	0.01424	0.029351	0.628468
16621_77_	finngen_R	outcome	16621_77_	Inverse va	2	-0.38221	0.195573	0.050665
16751_15_	finngen_R	outcome	16751_15_	MR Egger	3	1.609598	1.149539	0.394818
16751_15_	finngen_R	outcome	16751_15_	Weighted	3	0.295225	0.34505	0.392219
16751_15_	finngen_R	outcome	16751_15_	Inverse va	3	0.154273	0.306384	0.614594
16751_15_	finngen_R	outcome	16751_15_	Simple mc	3	0.39095	0.476608	0.498267
16751_15_	finngen_R	outcome	16751_15_	Weighted	3	0.331708	0.391055	0.485632
16753_46_	finngen_R	outcome	16753_46_	MR Egger	37	0.186664	0.133639	0.171274
16753_46_	finngen_R	outcome	16753_46_	Weighted	37	0.124826	0.109234	0.253147
16753_46_	finngen_R	outcome	16753_46_	Inverse va	37	0.093975	0.074214	0.205416
16753_46_	finngen_R	outcome	16753_46_	Simple mc	37	-0.07076	0.214327	0.743187
16753_46_	finngen_R	outcome	16753_46_	Weighted	37	0.075408	0.133086	0.574493
16758_96_	finngen_R	outcome	16758_96_	Wald ratic	1	-0.88141	0.477662	0.065001
16763_11_	finngen_R	outcome	16763_11_	MR Egger	102	0.002061	0.046969	0.96509
16763_11_	finngen_R	outcome	16763_11_	Weighted	102	-0.00985	0.048745	0.83993
16763_11_	finngen_R	outcome	16763_11_	Inverse va	102	0.018624	0.029988	0.534568
16763_11_	finngen_R	outcome	16763_11_	Simple mc	102	0.02011	0.112414	0.858384
16763_11_	finngen_R	outcome	16763_11_	Weighted	102	0.02011	0.046166	0.664062
16770_3_F	finngen_R	outcome	16770_3_F	MR Egger	56	0.134648	0.096912	0.170421
16770_3_F	finngen_R	outcome	16770_3_F	Weighted	56	0.138706	0.087766	0.114016
16770_3_F	finngen_R	outcome	16770_3_F	Inverse va	56	0.096039	0.0567	0.090304
16770_3_F	finngen_R	outcome	16770_3_F	Simple mc	56	-0.01871	0.177847	0.916618
16770_3_F	finngen_R	outcome	16770_3_F	Weighted	56	0.18458	0.094112	0.054917
16773_29_	finngen_R	outcome	16773_29_	MR Egger	18	-0.26726	0.272569	0.341428
16773_29_	finngen_R	outcome	16773_29_	Weighted	18	-0.39188	0.158125	0.013201
16773_29_	finngen_R	outcome	16773_29_	Inverse va	18	-0.41743	0.14531	0.00407
16773_29_	finngen_R	outcome	16773_29_	Simple mc	18	-0.76694	0.333437	0.03438
16773_29_	finngen_R	outcome	16773_29_	Weighted	18	-0.4108	0.158032	0.018699
16781_2_E	finngen_R	outcome	16781_2_E	MR Egger	92	0.005657	0.053554	0.916113
16781_2_E	finngen_R	outcome	16781_2_E	Weighted	92	-0.05231	0.049121	0.286938
16781_2_E	finngen_R	outcome	16781_2_E	Inverse va	92	-0.08573	0.035793	0.016609
16781_2_E	finngen_R	outcome	16781_2_E	Simple mc	92	-0.21473	0.092443	0.022417
16781_2_E	finngen_R	outcome	16781_2_E	Weighted	92	-0.03707	0.048388	0.445651

16785_45_	finngen_R	outcome	16785_45_	Inverse va	2	0.34315	0.29544	0.245443
16792_4_S	finngen_R	outcome	16792_4_S	MR Egger	195	-0.04742	0.024282	0.052268
16792_4_S	finngen_R	outcome	16792_4_S	Weighted	195	-0.04324	0.028235	0.12563
16792_4_S	finngen_R	outcome	16792_4_S	Inverse va	195	-0.01623	0.016407	0.322587
16792_4_S	finngen_R	outcome	16792_4_S	Simple mc	195	-0.00508	0.044498	0.909159
16792_4_S	finngen_R	outcome	16792_4_S	Weighted	195	-0.03217	0.023242	0.167977
16805_5_F	finngen_R	outcome	16805_5_F	MR Egger	26	-0.32502	0.253769	0.212511
16805_5_F	finngen_R	outcome	16805_5_F	Weighted	26	-0.22279	0.147474	0.130862
16805_5_F	finngen_R	outcome	16805_5_F	Inverse va	26	-0.14682	0.10795	0.173812
16805_5_F	finngen_R	outcome	16805_5_F	Simple mc	26	0.02677	0.276971	0.923772
16805_5_F	finngen_R	outcome	16805_5_F	Weighted	26	-0.20251	0.153459	0.198905
16818_20(	finngen_R	outcome	16818_20(	MR Egger	32	-0.17953	0.165457	0.286536
16818_20(	finngen_R	outcome	16818_20(	Weighted	32	-0.04539	0.12589	0.718415
16818_20(	finngen_R	outcome	16818_20(	Inverse va	32	-0.05461	0.078399	0.486054
16818_20(	finngen_R	outcome	16818_20(	Simple mc	32	0.033875	0.205499	0.87014
16818_20(	finngen_R	outcome	16818_20(	Weighted	32	-0.02793	0.128142	0.828907
16828_8_(	finngen_R	outcome	16828_8_(	MR Egger	151	0.015902	0.031789	0.617652
16828_8_(	finngen_R	outcome	16828_8_(	Weighted	151	0.010624	0.028466	0.708978
16828_8_(	finngen_R	outcome	16828_8_(	Inverse va	151	0.005611	0.018267	0.758727
16828_8_(	finngen_R	outcome	16828_8_(	Simple mc	151	0.004581	0.049324	0.926127
16828_8_(	finngen_R	outcome	16828_8_(	Weighted	151	0.004581	0.026201	0.861444
16836_1_(	finngen_R	outcome	16836_1_(	MR Egger	4	0.142421	0.745498	0.866129
16836_1_(	finngen_R	outcome	16836_1_(	Weighted	4	0.15864	0.297289	0.593602
16836_1_(	finngen_R	outcome	16836_1_(	Inverse va	4	0.134656	0.268196	0.61561
16836_1_(	finngen_R	outcome	16836_1_(	Simple mc	4	-0.27972	0.434115	0.565235
16836_1_(	finngen_R	outcome	16836_1_(	Weighted	4	0.17251	0.324528	0.631867
16872_24ξ	finngen_R	outcome	16872_24ξ	MR Egger	178	0.025575	0.026313	0.332416
16872_24ξ	finngen_R	outcome	16872_24ξ	Weighted	178	-0.00927	0.023795	0.696854
16872_24ξ	finngen_R	outcome	16872_24ξ	Inverse va	178	0.003404	0.015787	0.82927
16872_24ξ	finngen_R	outcome	16872_24ξ	Simple mc	178	0.006581	0.039369	0.867429
16872_24ξ	finngen_R	outcome	16872_24ξ	Weighted	178	0.006581	0.020704	0.750955
16890_37_	finngen_R	outcome	16890_37_	MR Egger	53	-0.10847	0.106694	0.314121
16890_37_	finngen_R	outcome	16890_37_	Weighted	53	-0.06291	0.088323	0.476294
16890_37_	finngen_R	outcome	16890_37_	Inverse va	53	-0.0353	0.057192	0.53713
16890_37_	finngen_R	outcome	16890_37_	Simple mc	53	-0.12131	0.141533	0.395309
16890_37_	finngen_R	outcome	16890_37_	Weighted	53	-0.10967	0.103469	0.294067
16892_23_	finngen_R	outcome	16892_23_	MR Egger	20	0.227405	0.389412	0.566488
16892_23_	finngen_R	outcome	16892_23_	Weighted	20	0.06341	0.163494	0.698134
16892_23_	finngen_R	outcome	16892_23_	Inverse va	20	0.095168	0.119863	0.42721
16892_23_	finngen_R	outcome	16892_23_	Simple mc	20	0.029259	0.257799	0.910827
16892_23_	finngen_R	outcome	16892_23_	Weighted	20	0.005773	0.230965	0.980318
16908_5_(	finngen_R	outcome	16908_5_(	Wald ratic	1	-0.25524	0.937832	0.785501
16913_8_F	finngen_R	outcome	16913_8_F	MR Egger	54	-0.32115	0.081896	0.000259
16913_8_F	finngen_R	outcome	16913_8_F	Weighted	54	-0.22554	0.074574	0.002492
16913_8_F	finngen_R	outcome	16913_8_F	Inverse va	54	-0.22506	0.050975	1.01E-05
16913_8_F	finngen_R	outcome	16913_8_F	Simple mc	54	-0.15065	0.124301	0.230912
16913_8_F	finngen_R	outcome	16913_8_F	Weighted	54	-0.23324	0.070146	0.00161
16914_10⁄	finngen_R	outcome	16914_10⁄	MR Egger	32	-0.21561	0.198476	0.28599
16914_10⁄	finngen_R	outcome	16914_10⁄	Weighted	32	-0.33063	0.123318	0.007338
16914_10⁄	finngen_R	outcome	16914_10⁄	Inverse va	32	-0.16904	0.089884	0.060015
16914_10⁄	finngen_R	outcome	16914_10⁄	Simple mc	32	-0.57637	0.228595	0.017045
16914_10⁄	finngen_R	outcome	16914_10⁄	Weighted	32	-0.38886	0.132193	0.006129
16918_19ξ	finngen_R	outcome	16918_19ξ	MR Egger	161	-0.02761	0.052943	0.602721
16918_19ξ	finngen_R	outcome	16918_19ξ	Weighted	161	-0.0383	0.048193	0.426806
16918_19ξ	finngen_R	outcome	16918_19ξ	Inverse va	161	-0.0694	0.029286	0.017802
16918_19ξ	finngen_R	outcome	16918_19ξ	Simple mc	161	-0.03061	0.092972	0.742412
16918_19ξ	finngen_R	outcome	16918_19ξ	Weighted	161	-0.03061	0.04992	0.540639
17138_8_(	finngen_R	outcome	17138_8_(	MR Egger	39	0.101616	0.110052	0.361807

17138_8_(	finngen_R	outcome	17138_8_(	Weighted	39	0.051218	0.091256	0.574619
17138_8_(	finngen_R	outcome	17138_8_(	Inverse va	39	-0.0161	0.065122	0.804676
17138_8_(	finngen_R	outcome	17138_8_(	Simple mc	39	-0.00558	0.159706	0.972316
17138_8_(	finngen_R	outcome	17138_8_(	Weighted	39	0.00835	0.083252	0.920638
17140_57_	finngen_R	outcome	17140_57_	MR Egger	57	-0.15916	0.109189	0.150619
17140_57_	finngen_R	outcome	17140_57_	Weighted	57	-0.03657	0.086517	0.672484
17140_57_	finngen_R	outcome	17140_57_	Inverse va	57	0.008567	0.053131	0.871897
17140_57_	finngen_R	outcome	17140_57_	Simple mc	57	-0.12861	0.136015	0.348422
17140_57_	finngen_R	outcome	17140_57_	Weighted	57	-0.08682	0.074649	0.249724
17153_46_	finngen_R	outcome	17153_46_	MR Egger	117	-0.09475	0.062363	0.131424
17153_46_	finngen_R	outcome	17153_46_	Weighted	117	-0.05015	0.055588	0.36694
17153_46_	finngen_R	outcome	17153_46_	Inverse va	117	-0.00466	0.037235	0.90048
17153_46_	finngen_R	outcome	17153_46_	Simple mc	117	-0.04849	0.104114	0.642305
17153_46_	finngen_R	outcome	17153_46_	Weighted	117	-0.1318	0.061908	0.035379
17156_72_	finngen_R	outcome	17156_72_	MR Egger	9	0.225875	0.301739	0.478504
17156_72_	finngen_R	outcome	17156_72_	Weighted	9	0.036812	0.167349	0.825893
17156_72_	finngen_R	outcome	17156_72_	Inverse va	9	-0.02956	0.134451	0.825984
17156_72_	finngen_R	outcome	17156_72_	Simple mc	9	0.066295	0.23512	0.78513
17156_72_	finngen_R	outcome	17156_72_	Weighted	9	0.038779	0.17573	0.830873
17161_1_[	finngen_R	outcome	17161_1_[	MR Egger	3	-0.91549	1.190393	0.582637
17161_1_[	finngen_R	outcome	17161_1_[	Weighted	3	0.253123	0.404789	0.531761
17161_1_[	finngen_R	outcome	17161_1_[	Inverse va	3	0.355379	0.355401	0.317341
17161_1_[	finngen_R	outcome	17161_1_[	Simple mc	3	-0.05912	0.60825	0.931437
17161_1_[	finngen_R	outcome	17161_1_[	Weighted	3	-0.04442	0.585092	0.946393
17170_15_	finngen_R	outcome	17170_15_	MR Egger	18	0.115131	0.254919	0.657591
17170_15_	finngen_R	outcome	17170_15_	Weighted	18	-0.11846	0.158048	0.453534
17170_15_	finngen_R	outcome	17170_15_	Inverse va	18	-0.00354	0.13308	0.978793
17170_15_	finngen_R	outcome	17170_15_	Simple mc	18	-0.2736	0.266331	0.318682
17170_15_	finngen_R	outcome	17170_15_	Weighted	18	-0.17306	0.158673	0.290636
17224_12_	finngen_R	outcome	17224_12_	MR Egger	63	-0.03574	0.106774	0.738979
17224_12_	finngen_R	outcome	17224_12_	Weighted	63	-0.0489	0.079117	0.536512
17224_12_	finngen_R	outcome	17224_12_	Inverse va	63	-0.11353	0.051798	0.028395
17224_12_	finngen_R	outcome	17224_12_	Simple mc	63	-0.2857	0.159738	0.078573
17224_12_	finngen_R	outcome	17224_12_	Weighted	63	-0.09761	0.082815	0.243039
17377_1_/	finngen_R	outcome	17377_1_/	MR Egger	31	-0.06572	0.21806	0.765275
17377_1_/	finngen_R	outcome	17377_1_/	Weighted	31	0.012682	0.149974	0.932609
17377_1_/	finngen_R	outcome	17377_1_/	Inverse va	31	0.085395	0.10694	0.424565
17377_1_/	finngen_R	outcome	17377_1_/	Simple mc	31	0.02502	0.277603	0.928784
17377_1_/	finngen_R	outcome	17377_1_/	Weighted	31	0.006386	0.235288	0.978525
17396_23_	finngen_R	outcome	17396_23_	MR Egger	4	-0.95722	0.537821	0.217066
17396_23_	finngen_R	outcome	17396_23_	Weighted	4	-1.21691	0.39386	0.002004
17396_23_	finngen_R	outcome	17396_23_	Inverse va	4	-1.15377	0.320784	0.000322
17396_23_	finngen_R	outcome	17396_23_	Simple mc	4	-1.43486	0.522731	0.071043
17396_23_	finngen_R	outcome	17396_23_	Weighted	4	-0.82643	0.460959	0.1709
17398_55_	finngen_R	outcome	17398_55_	MR Egger	9	-0.87251	0.402227	0.066697
17398_55_	finngen_R	outcome	17398_55_	Weighted	9	-0.23149	0.298172	0.437537
17398_55_	finngen_R	outcome	17398_55_	Inverse va	9	-0.12891	0.218173	0.554615
17398_55_	finngen_R	outcome	17398_55_	Simple mc	9	-0.28317	0.421463	0.520593
17398_55_	finngen_R	outcome	17398_55_	Weighted	9	-0.35752	0.371878	0.364511
17447_52_	finngen_R	outcome	17447_52_	MR Egger	8	-0.01409	0.234046	0.953942
17447_52_	finngen_R	outcome	17447_52_	Weighted	8	0.079804	0.147957	0.58963
17447_52_	finngen_R	outcome	17447_52_	Inverse va	8	0.015585	0.130308	0.904797
17447_52_	finngen_R	outcome	17447_52_	Simple mc	8	0.152481	0.205419	0.48207
17447_52_	finngen_R	outcome	17447_52_	Weighted	8	0.071582	0.161817	0.671572
17453_34_	finngen_R	outcome	17453_34_	Wald ratic	1	-1.12124	0.819902	0.17146
17672_18‹	finngen_R	outcome	17672_18‹	MR Egger	10	0.135558	0.188152	0.491748
17672_18‹	finngen_R	outcome	17672_18‹	Weighted	10	0.102277	0.169986	0.547388
17672_18‹	finngen_R	outcome	17672_18‹	Inverse va	10	0.24638	0.139636	0.077657

17672_184	finngen_R	outcome	17672_184	Simple mc	10	0.406501	0.347	0.271487
17672_184	finngen_R	outcome	17672_184	Weighted	10	0.082857	0.188421	0.670492
17677_47_	finngen_R	outcome	17677_47_	MR Egger	4	1.238555	2.259234	0.638558
17677_47_	finngen_R	outcome	17677_47_	Weighted	4	-0.09819	0.250541	0.695135
17677_47_	finngen_R	outcome	17677_47_	Inverse va	4	-0.17974	0.301806	0.55147
17677_47_	finngen_R	outcome	17677_47_	Simple mc	4	-0.03678	0.433806	0.937773
17677_47_	finngen_R	outcome	17677_47_	Weighted	4	-0.04876	0.2657	0.866105
17680_12_	finngen_R	outcome	17680_12_	MR Egger	39	-0.14378	0.141704	0.316863
17680_12_	finngen_R	outcome	17680_12_	Weighted	39	-0.15342	0.095313	0.107474
17680_12_	finngen_R	outcome	17680_12_	Inverse va	39	-0.02501	0.065641	0.703192
17680_12_	finngen_R	outcome	17680_12_	Simple mc	39	-0.12804	0.187907	0.499757
17680_12_	finngen_R	outcome	17680_12_	Weighted	39	-0.13529	0.101849	0.191989
17682_1_(	finngen_R	outcome	17682_1_(	MR Egger	16	-0.2631	0.249117	0.308797
17682_1_(	finngen_R	outcome	17682_1_(	Weighted	16	0.020213	0.126408	0.872958
17682_1_(	finngen_R	outcome	17682_1_(	Inverse va	16	0.002377	0.088187	0.978492
17682_1_(	finngen_R	outcome	17682_1_(	Simple mc	16	0.17398	0.187418	0.367951
17682_1_(	finngen_R	outcome	17682_1_(	Weighted	16	0.024824	0.130415	0.851593
17685_9_/	finngen_R	outcome	17685_9_/	MR Egger	6	0.688823	0.418812	0.175376
17685_9_/	finngen_R	outcome	17685_9_/	Weighted	6	0.44048	0.301122	0.143523
17685_9_/	finngen_R	outcome	17685_9_/	Inverse va	6	0.20436	0.23351	0.381482
17685_9_/	finngen_R	outcome	17685_9_/	Simple mc	6	0.356015	0.432436	0.447823
17685_9_/	finngen_R	outcome	17685_9_/	Weighted	6	0.451652	0.346729	0.249475
17691_1_T	finngen_R	outcome	17691_1_T	MR Egger	12	-0.13293	0.302386	0.669577
17691_1_T	finngen_R	outcome	17691_1_T	Weighted	12	0.199544	0.202264	0.323864
17691_1_T	finngen_R	outcome	17691_1_T	Inverse va	12	0.070744	0.15461	0.647265
17691_1_T	finngen_R	outcome	17691_1_T	Simple mc	12	0.311628	0.329703	0.364868
17691_1_T	finngen_R	outcome	17691_1_T	Weighted	12	0.145895	0.249602	0.570671
17692_2_E	finngen_R	outcome	17692_2_E	MR Egger	24	-0.25842	0.107045	0.024539
17692_2_E	finngen_R	outcome	17692_2_E	Weighted	24	-0.27816	0.074303	0.000181
17692_2_E	finngen_R	outcome	17692_2_E	Inverse va	24	-0.19912	0.063372	0.001678
17692_2_E	finngen_R	outcome	17692_2_E	Simple mc	24	-0.03271	0.161764	0.841544
17692_2_E	finngen_R	outcome	17692_2_E	Weighted	24	-0.27292	0.073658	0.001166
17702_53_	finngen_R	outcome	17702_53_	MR Egger	17	-0.46616	0.270959	0.105917
17702_53_	finngen_R	outcome	17702_53_	Weighted	17	-0.21233	0.145875	0.145515
17702_53_	finngen_R	outcome	17702_53_	Inverse va	17	-0.14919	0.117008	0.202289
17702_53_	finngen_R	outcome	17702_53_	Simple mc	17	-0.12742	0.298962	0.675627
17702_53_	finngen_R	outcome	17702_53_	Weighted	17	-0.22528	0.149559	0.151474
17722_5_F	finngen_R	outcome	17722_5_F	Inverse va	2	-0.09416	1.029114	0.927099
17786_5_(	finngen_R	outcome	17786_5_(	Inverse va	2	-0.6545	0.365344	0.073219
17813_21_	finngen_R	outcome	17813_21_	MR Egger	3	0.7631	0.727646	0.484862
17813_21_	finngen_R	outcome	17813_21_	Weighted	3	-0.00466	0.260848	0.985755
17813_21_	finngen_R	outcome	17813_21_	Inverse va	3	-0.0027	0.250159	0.991378
17813_21_	finngen_R	outcome	17813_21_	Simple mc	3	0.256838	0.363322	0.552883
17813_21_	finngen_R	outcome	17813_21_	Weighted	3	0.055758	0.283279	0.862149
17814_8_E	finngen_R	outcome	17814_8_E	Wald ratic	1	0.024753	0.614586	0.967874
18162_167	finngen_R	outcome	18162_167	Wald ratic	1	-0.21141	0.544678	0.697917
18206_18_	finngen_R	outcome	18206_18_	MR Egger	15	-0.04365	0.255893	0.867179
18206_18_	finngen_R	outcome	18206_18_	Weighted	15	-0.1376	0.177473	0.438146
18206_18_	finngen_R	outcome	18206_18_	Inverse va	15	-0.05337	0.129033	0.67916
18206_18_	finngen_R	outcome	18206_18_	Simple mc	15	-0.16073	0.305021	0.606478
18206_18_	finngen_R	outcome	18206_18_	Weighted	15	-0.18026	0.213088	0.411813
18216_22_	finngen_R	outcome	18216_22_	MR Egger	22	0.117841	0.093543	0.222261
18216_22_	finngen_R	outcome	18216_22_	Weighted	22	0.056644	0.08567	0.508495
18216_22_	finngen_R	outcome	18216_22_	Inverse va	22	0.007624	0.064488	0.905888
18216_22_	finngen_R	outcome	18216_22_	Simple mc	22	-0.0073	0.125678	0.954212
18216_22_	finngen_R	outcome	18216_22_	Weighted	22	0.069135	0.085454	0.427567
18225_13_	finngen_R	outcome	18225_13_	MR Egger	59	-0.017	0.056575	0.764924
18225_13_	finngen_R	outcome	18225_13_	Weighted	59	-0.03888	0.057322	0.497612

18225_13_	finngen_R	outcome	18225_13_	Inverse va	59	0.006354	0.039932	0.873567
18225_13_	finngen_R	outcome	18225_13_	Simple mc	59	0.162209	0.116805	0.170229
18225_13_	finngen_R	outcome	18225_13_	Weighted	59	-0.04687	0.050335	0.355583
18340_2_F	finngen_R	outcome	18340_2_F	MR Egger	38	-0.10229	0.072306	0.16577
18340_2_F	finngen_R	outcome	18340_2_F	Weighted	38	-0.11587	0.06466	0.073147
18340_2_F	finngen_R	outcome	18340_2_F	Inverse va	38	-0.16711	0.050654	0.00097
18340_2_F	finngen_R	outcome	18340_2_F	Simple mc	38	-0.10239	0.148043	0.493472
18340_2_F	finngen_R	outcome	18340_2_F	Weighted	38	-0.12209	0.062183	0.057142
18381_16_	finngen_R	outcome	18381_16_	MR Egger	6	-0.23827	0.753688	0.767709
18381_16_	finngen_R	outcome	18381_16_	Weighted	6	-0.26555	0.311938	0.3946
18381_16_	finngen_R	outcome	18381_16_	Inverse va	6	-0.21388	0.299965	0.475835
18381_16_	finngen_R	outcome	18381_16_	Simple mc	6	-0.52179	0.512721	0.355519
18381_16_	finngen_R	outcome	18381_16_	Weighted	6	-0.38894	0.439527	0.416724
18382_10	finngen_R	outcome	18382_10	Wald ratic	1	0.575033	0.420103	0.171065
18422_41_	finngen_R	outcome	18422_41_	Inverse va	2	1.100354	0.634239	0.082755
18819_21_	finngen_R	outcome	18819_21_	MR Egger	80	-0.03694	0.082699	0.65631
18819_21_	finngen_R	outcome	18819_21_	Weighted	80	0.017039	0.066729	0.798451
18819_21_	finngen_R	outcome	18819_21_	Inverse va	80	0.034384	0.040382	0.394512
18819_21_	finngen_R	outcome	18819_21_	Simple mc	80	0.08103	0.119889	0.501096
18819_21_	finngen_R	outcome	18819_21_	Weighted	80	0.054718	0.071073	0.443669
18830_1_I	finngen_R	outcome	18830_1_I	MR Egger	9	-0.11922	0.410349	0.779823
18830_1_I	finngen_R	outcome	18830_1_I	Weighted	9	-0.19775	0.276437	0.474397
18830_1_I	finngen_R	outcome	18830_1_I	Inverse va	9	-0.06702	0.218311	0.758859
18830_1_I	finngen_R	outcome	18830_1_I	Simple mc	9	-0.11155	0.39936	0.787082
18830_1_I	finngen_R	outcome	18830_1_I	Weighted	9	-0.16106	0.336314	0.644825
18832_65_	finngen_R	outcome	18832_65_	MR Egger	45	-0.04195	0.103373	0.686908
18832_65_	finngen_R	outcome	18832_65_	Weighted	45	-0.1239	0.0799	0.120972
18832_65_	finngen_R	outcome	18832_65_	Inverse va	45	0.011137	0.068069	0.87003
18832_65_	finngen_R	outcome	18832_65_	Simple mc	45	-0.08105	0.164958	0.62562
18832_65_	finngen_R	outcome	18832_65_	Weighted	45	-0.06986	0.070645	0.32816
18839_24_	finngen_R	outcome	18839_24_	Wald ratic	1	0.156334	0.711258	0.826027
18841_1_S	finngen_R	outcome	18841_1_S	MR Egger	15	0.315996	0.275594	0.272213
18841_1_S	finngen_R	outcome	18841_1_S	Weighted	15	0.202899	0.180513	0.261008
18841_1_S	finngen_R	outcome	18841_1_S	Inverse va	15	0.118497	0.138543	0.392382
18841_1_S	finngen_R	outcome	18841_1_S	Simple mc	15	0.129907	0.268368	0.635825
18841_1_S	finngen_R	outcome	18841_1_S	Weighted	15	0.177095	0.216673	0.427427
18864_7_F	finngen_R	outcome	18864_7_F	MR Egger	52	0.052111	0.08882	0.560041
18864_7_F	finngen_R	outcome	18864_7_F	Weighted	52	0.059929	0.059621	0.314813
18864_7_F	finngen_R	outcome	18864_7_F	Inverse va	52	0.068864	0.048669	0.157081
18864_7_F	finngen_R	outcome	18864_7_F	Simple mc	52	0.043224	0.102421	0.67478
18864_7_F	finngen_R	outcome	18864_7_F	Weighted	52	0.043224	0.055866	0.442675
18873_8_(	finngen_R	outcome	18873_8_(	MR Egger	4	-1.37479	1.162806	0.358603
18873_8_(	finngen_R	outcome	18873_8_(	Weighted	4	-0.63516	0.372222	0.087936
18873_8_(	finngen_R	outcome	18873_8_(	Inverse va	4	-0.57377	0.316234	0.069618
18873_8_(	finngen_R	outcome	18873_8_(	Simple mc	4	-0.67333	0.479288	0.254705
18873_8_(	finngen_R	outcome	18873_8_(	Weighted	4	-0.66353	0.44453	0.232353
18875_12	finngen_R	outcome	18875_12	MR Egger	107	-0.08684	0.051002	0.091595
18875_12	finngen_R	outcome	18875_12	Weighted	107	-0.06945	0.050787	0.171455
18875_12	finngen_R	outcome	18875_12	Inverse va	107	-0.03868	0.031532	0.219928
18875_12	finngen_R	outcome	18875_12	Simple mc	107	-0.01039	0.095187	0.913261
18875_12	finngen_R	outcome	18875_12	Weighted	107	-0.0624	0.04572	0.175176
18876_77_	finngen_R	outcome	18876_77_	Inverse va	2	0.237487	0.370713	0.521768
18878_15_	finngen_R	outcome	18878_15_	MR Egger	78	0.039484	0.059084	0.505988
18878_15_	finngen_R	outcome	18878_15_	Weighted	78	-0.06382	0.055682	0.251734
18878_15_	finngen_R	outcome	18878_15_	Inverse va	78	-0.03186	0.035663	0.37162
18878_15_	finngen_R	outcome	18878_15_	Simple mc	78	-0.11211	0.106232	0.294578
18878_15_	finngen_R	outcome	18878_15_	Weighted	78	-0.02032	0.054006	0.707766
18880_81_	finngen_R	outcome	18880_81_	MR Egger	3	2.270944	2.062842	0.469454

18880_81_	finngen_R	outcome	18880_81_	Weighted	3	-0.36175	0.483312	0.454164
18880_81_	finngen_R	outcome	18880_81_	Inverse va	3	-0.39439	0.42625	0.354827
18880_81_	finngen_R	outcome	18880_81_	Simple mc	3	-0.03299	0.60897	0.961718
18880_81_	finngen_R	outcome	18880_81_	Weighted	3	-0.26832	0.534945	0.665728
18881_7_(	finngen_R	outcome	18881_7_(	MR Egger	11	0.264955	0.242718	0.303361
18881_7_(	finngen_R	outcome	18881_7_(	Weighted	11	0.051967	0.199459	0.794447
18881_7_(	finngen_R	outcome	18881_7_(	Inverse va	11	0.13856	0.146894	0.345544
18881_7_(	finngen_R	outcome	18881_7_(	Simple mc	11	-0.17729	0.316296	0.587455
18881_7_(	finngen_R	outcome	18881_7_(	Weighted	11	-0.06615	0.268047	0.810056
18893_26_	finngen_R	outcome	18893_26_	Wald ratic	1	0.623702	0.575099	0.278138
18895_54_	finngen_R	outcome	18895_54_	MR Egger	128	-0.00454	0.039481	0.908642
18895_54_	finngen_R	outcome	18895_54_	Weighted	128	-0.01107	0.04129	0.788577
18895_54_	finngen_R	outcome	18895_54_	Inverse va	128	0.007773	0.026437	0.768738
18895_54_	finngen_R	outcome	18895_54_	Simple mc	128	-0.00184	0.078107	0.981203
18895_54_	finngen_R	outcome	18895_54_	Weighted	128	-0.00184	0.037761	0.961131
18917_53_	finngen_R	outcome	18917_53_	MR Egger	50	0.052925	0.078238	0.501993
18917_53_	finngen_R	outcome	18917_53_	Weighted	50	0.103601	0.073758	0.160137
18917_53_	finngen_R	outcome	18917_53_	Inverse va	50	0.071966	0.049843	0.148779
18917_53_	finngen_R	outcome	18917_53_	Simple mc	50	0.078246	0.113127	0.492414
18917_53_	finngen_R	outcome	18917_53_	Weighted	50	0.092357	0.072732	0.210141
18918_86_	finngen_R	outcome	18918_86_	MR Egger	6	0.574558	0.498397	0.313191
18918_86_	finngen_R	outcome	18918_86_	Weighted	6	0.28741	0.292321	0.325509
18918_86_	finngen_R	outcome	18918_86_	Inverse va	6	0.210994	0.229353	0.357597
18918_86_	finngen_R	outcome	18918_86_	Simple mc	6	0.360959	0.369203	0.373138
18918_86_	finngen_R	outcome	18918_86_	Weighted	6	0.31148	0.391588	0.462437
18922_27_	finngen_R	outcome	18922_27_	MR Egger	9	0.792693	0.487121	0.147699
18922_27_	finngen_R	outcome	18922_27_	Weighted	9	0.345195	0.290729	0.235091
18922_27_	finngen_R	outcome	18922_27_	Inverse va	9	0.068627	0.209798	0.743586
18922_27_	finngen_R	outcome	18922_27_	Simple mc	9	0.433502	0.489022	0.401227
18922_27_	finngen_R	outcome	18922_27_	Weighted	9	0.485725	0.42225	0.283231
18930_28_	finngen_R	outcome	18930_28_	MR Egger	8	0.971154	0.866726	0.305342
18930_28_	finngen_R	outcome	18930_28_	Weighted	8	0.530565	0.224488	0.018106
18930_28_	finngen_R	outcome	18930_28_	Inverse va	8	0.328354	0.169161	0.052249
18930_28_	finngen_R	outcome	18930_28_	Simple mc	8	-0.13212	0.368898	0.730788
18930_28_	finngen_R	outcome	18930_28_	Weighted	8	0.618199	0.294924	0.074295
18934_50_	finngen_R	outcome	18934_50_	MR Egger	5	0.20173	0.482532	0.704021
18934_50_	finngen_R	outcome	18934_50_	Weighted	5	0.226863	0.277166	0.413066
18934_50_	finngen_R	outcome	18934_50_	Inverse va	5	0.195214	0.226673	0.38912
18934_50_	finngen_R	outcome	18934_50_	Simple mc	5	0.230069	0.364712	0.562403
18934_50_	finngen_R	outcome	18934_50_	Weighted	5	0.244426	0.338446	0.510136
19130_81_	finngen_R	outcome	19130_81_	MR Egger	16	0.109894	0.286576	0.70713
19130_81_	finngen_R	outcome	19130_81_	Weighted	16	0.007189	0.13637	0.957958
19130_81_	finngen_R	outcome	19130_81_	Inverse va	16	0.127818	0.101034	0.205837
19130_81_	finngen_R	outcome	19130_81_	Simple mc	16	-0.03518	0.219688	0.874911
19130_81_	finngen_R	outcome	19130_81_	Weighted	16	-0.01309	0.140785	0.927127
19136_22_	finngen_R	outcome	19136_22_	MR Egger	6	-0.89078	3.019118	0.78264
19136_22_	finngen_R	outcome	19136_22_	Weighted	6	0.124772	0.316577	0.693486
19136_22_	finngen_R	outcome	19136_22_	Inverse va	6	0.111192	0.263171	0.672655
19136_22_	finngen_R	outcome	19136_22_	Simple mc	6	0.366879	0.448397	0.45046
19136_22_	finngen_R	outcome	19136_22_	Weighted	6	0.127716	0.376482	0.748217
19154_41_	finngen_R	outcome	19154_41_	MR Egger	78	-0.00097	0.07578	0.98987
19154_41_	finngen_R	outcome	19154_41_	Weighted	78	0.051519	0.060519	0.394616
19154_41_	finngen_R	outcome	19154_41_	Inverse va	78	0.073516	0.045029	0.102548
19154_41_	finngen_R	outcome	19154_41_	Simple mc	78	0.200388	0.131389	0.131317
19154_41_	finngen_R	outcome	19154_41_	Weighted	78	0.047982	0.055598	0.39081
19207_11(	finngen_R	outcome	19207_11(	Inverse va	2	0.028038	0.549425	0.959301
19213_1_S	finngen_R	outcome	19213_1_S	MR Egger	10	0.272555	0.223408	0.257211
19213_1_S	finngen_R	outcome	19213_1_S	Weighted	10	0.128078	0.165839	0.439937

19213_1_S	finngen_R	outcome	19213_1_S	Inverse va	10	0.16871	0.139832	0.227617
19213_1_S	finngen_R	outcome	19213_1_S	Simple mc	10	0.13371	0.346049	0.708183
19213_1_S	finngen_R	outcome	19213_1_S	Weighted	10	0.126923	0.172493	0.480579
19238_12_	finngen_R	outcome	19238_12_	Wald ratic	1	0.543526	0.712879	0.445799
19251_56_	finngen_R	outcome	19251_56_	MR Egger	20	0.140475	0.232342	0.55299
19251_56_	finngen_R	outcome	19251_56_	Weighted	20	0.178894	0.163489	0.273858
19251_56_	finngen_R	outcome	19251_56_	Inverse va	20	-0.00683	0.121835	0.955301
19251_56_	finngen_R	outcome	19251_56_	Simple mc	20	0.084892	0.348673	0.81025
19251_56_	finngen_R	outcome	19251_56_	Weighted	20	0.150581	0.188917	0.435256
19267_14_	finngen_R	outcome	19267_14_	MR Egger	10	0.179011	0.300144	0.567386
19267_14_	finngen_R	outcome	19267_14_	Weighted	10	0.032199	0.17562	0.854528
19267_14_	finngen_R	outcome	19267_14_	Inverse va	10	-0.05186	0.144799	0.720215
19267_14_	finngen_R	outcome	19267_14_	Simple mc	10	-0.07992	0.279095	0.781084
19267_14_	finngen_R	outcome	19267_14_	Weighted	10	0.071522	0.200076	0.728975
19273_3_(	finngen_R	outcome	19273_3_(	MR Egger	7	0.228001	0.581924	0.711344
19273_3_(	finngen_R	outcome	19273_3_(	Weighted	7	0.469809	0.262777	0.073798
19273_3_(	finngen_R	outcome	19273_3_(	Inverse va	7	0.353775	0.217531	0.103882
19273_3_(	finngen_R	outcome	19273_3_(	Simple mc	7	0.455727	0.357025	0.248972
19273_3_(	finngen_R	outcome	19273_3_(	Weighted	7	0.512492	0.278388	0.11523
19334_62_	finngen_R	outcome	19334_62_	Inverse va	2	0.511144	0.3366	0.128876
19347_37_	finngen_R	outcome	19347_37_	Wald ratic	1	0.138122	0.764067	0.856546
19361_78_	finngen_R	outcome	19361_78_	MR Egger	138	0.034368	0.043538	0.431258
19361_78_	finngen_R	outcome	19361_78_	Weighted	138	0.052044	0.048579	0.284023
19361_78_	finngen_R	outcome	19361_78_	Inverse va	138	0.074899	0.026971	0.005486
19361_78_	finngen_R	outcome	19361_78_	Simple mc	138	0.102567	0.081439	0.210016
19361_78_	finngen_R	outcome	19361_78_	Weighted	138	0.063089	0.0427	0.14184
19376_74_	finngen_R	outcome	19376_74_	Inverse va	2	-0.62849	0.957999	0.511797
19377_14_	finngen_R	outcome	19377_14_	MR Egger	84	0.009514	0.070512	0.892999
19377_14_	finngen_R	outcome	19377_14_	Weighted	84	0.026623	0.054712	0.626539
19377_14_	finngen_R	outcome	19377_14_	Inverse va	84	0.04296	0.038525	0.264801
19377_14_	finngen_R	outcome	19377_14_	Simple mc	84	-0.12234	0.104949	0.247057
19377_14_	finngen_R	outcome	19377_14_	Weighted	84	0.060961	0.049069	0.217608
19437_61_	finngen_R	outcome	19437_61_	MR Egger	86	-0.14829	0.084159	0.081699
19437_61_	finngen_R	outcome	19437_61_	Weighted	86	-0.05211	0.0647	0.420611
19437_61_	finngen_R	outcome	19437_61_	Inverse va	86	-0.10925	0.049381	0.026937
19437_61_	finngen_R	outcome	19437_61_	Simple mc	86	-0.14284	0.142276	0.318259
19437_61_	finngen_R	outcome	19437_61_	Weighted	86	-0.10444	0.064198	0.107457
19448_10	finngen_R	outcome	19448_10	MR Egger	4	-1.10644	0.690414	0.250203
19448_10	finngen_R	outcome	19448_10	Weighted	4	-0.1521	0.27428	0.579202
19448_10	finngen_R	outcome	19448_10	Inverse va	4	-0.20728	0.231466	0.370507
19448_10	finngen_R	outcome	19448_10	Simple mc	4	0.078236	0.388765	0.853381
19448_10	finngen_R	outcome	19448_10	Weighted	4	-0.16572	0.302217	0.62162
19556_12_	finngen_R	outcome	19556_12_	MR Egger	85	0.023823	0.065064	0.715183
19556_12_	finngen_R	outcome	19556_12_	Weighted	85	0.015809	0.062912	0.801588
19556_12_	finngen_R	outcome	19556_12_	Inverse va	85	0.012179	0.042447	0.77417
19556_12_	finngen_R	outcome	19556_12_	Simple mc	85	0.017073	0.105869	0.872274
19556_12_	finngen_R	outcome	19556_12_	Weighted	85	0.005074	0.053568	0.924758
19581_15_	finngen_R	outcome	19581_15_	Inverse va	2	0.809727	0.557223	0.146183
19590_46_	finngen_R	outcome	19590_46_	MR Egger	91	-0.01707	0.064492	0.791813
19590_46_	finngen_R	outcome	19590_46_	Weighted	91	0.029832	0.057794	0.605727
19590_46_	finngen_R	outcome	19590_46_	Inverse va	91	0.037679	0.038158	0.323422
19590_46_	finngen_R	outcome	19590_46_	Simple mc	91	0.090809	0.084993	0.288188
19590_46_	finngen_R	outcome	19590_46_	Weighted	91	0.029806	0.053778	0.580787
19606_28_	finngen_R	outcome	19606_28_	MR Egger	3	1.960522	1.028791	0.307649
19606_28_	finngen_R	outcome	19606_28_	Weighted	3	0.920342	0.543966	0.090663
19606_28_	finngen_R	outcome	19606_28_	Inverse va	3	0.747122	0.453095	0.099162
19606_28_	finngen_R	outcome	19606_28_	Simple mc	3	1.271324	0.729985	0.223708
19606_28_	finngen_R	outcome	19606_28_	Weighted	3	1.259338	0.79294	0.253172

19614_8_T	finngen_R	outcome	19614_8_T	MR Egger	58	0.057801	0.070697	0.417059
19614_8_T	finngen_R	outcome	19614_8_T	Weighted	58	0.02195	0.069053	0.750577
19614_8_T	finngen_R	outcome	19614_8_T	Inverse va	58	-0.0465	0.045343	0.305068
19614_8_T	finngen_R	outcome	19614_8_T	Simple mc	58	0.038949	0.113772	0.733354
19614_8_T	finngen_R	outcome	19614_8_T	Weighted	58	0.023115	0.063514	0.71725
19622_7_I	finngen_R	outcome	19622_7_I	Wald ratic	1	0.372128	0.412937	0.367496
19631_13_	finngen_R	outcome	19631_13_	MR Egger	20	-0.16574	0.186971	0.387065
19631_13_	finngen_R	outcome	19631_13_	Weighted	20	-0.05821	0.126812	0.646225
19631_13_	finngen_R	outcome	19631_13_	Inverse va	20	-0.05823	0.090489	0.519893
19631_13_	finngen_R	outcome	19631_13_	Simple mc	20	-0.00121	0.195677	0.99512
19631_13_	finngen_R	outcome	19631_13_	Weighted	20	-0.04786	0.130025	0.716886
19635_69_	finngen_R	outcome	19635_69_	Wald ratic	1	0.128855	0.81363	0.874165
19637_9_(	finngen_R	outcome	19637_9_(	Wald ratic	1	-0.5563	1.035643	0.591158
2190_55_F	finngen_R	outcome	2190_55_F	MR Egger	39	-0.0102	0.099138	0.918624
2190_55_F	finngen_R	outcome	2190_55_F	Weighted	39	-0.03243	0.078887	0.681034
2190_55_F	finngen_R	outcome	2190_55_F	Inverse va	39	0.004356	0.054147	0.935884
2190_55_F	finngen_R	outcome	2190_55_F	Simple mc	39	0.032483	0.135451	0.811763
2190_55_F	finngen_R	outcome	2190_55_F	Weighted	39	-0.0427	0.079232	0.593125
2192_63_(	finngen_R	outcome	2192_63_(	Wald ratic	1	-0.69653	0.708966	0.325873
2201_17_(	finngen_R	outcome	2201_17_(	MR Egger	23	-0.44932	0.211052	0.045259
2201_17_(	finngen_R	outcome	2201_17_(	Weighted	23	-0.15006	0.14582	0.303441
2201_17_(	finngen_R	outcome	2201_17_(	Inverse va	23	0.012353	0.103386	0.904895
2201_17_(	finngen_R	outcome	2201_17_(	Simple mc	23	-0.22977	0.241077	0.350899
2201_17_(	finngen_R	outcome	2201_17_(	Weighted	23	-0.22318	0.175443	0.216624
2212_69_F	finngen_R	outcome	2212_69_F	MR Egger	13	-0.22286	0.287307	0.454286
2212_69_F	finngen_R	outcome	2212_69_F	Weighted	13	-0.11687	0.211493	0.580554
2212_69_F	finngen_R	outcome	2212_69_F	Inverse va	13	-0.01952	0.169227	0.908178
2212_69_F	finngen_R	outcome	2212_69_F	Simple mc	13	-0.40209	0.337512	0.256554
2212_69_F	finngen_R	outcome	2212_69_F	Weighted	13	-0.19153	0.296301	0.53018
2278_61_T	finngen_R	outcome	2278_61_T	Inverse va	2	0.294296	0.376098	0.433922
2381_52_(	finngen_R	outcome	2381_52_(	MR Egger	14	-0.30715	0.328073	0.367613
2381_52_(	finngen_R	outcome	2381_52_(	Weighted	14	-0.18214	0.183606	0.321192
2381_52_(	finngen_R	outcome	2381_52_(	Inverse va	14	-0.11174	0.138268	0.419002
2381_52_(	finngen_R	outcome	2381_52_(	Simple mc	14	-0.26774	0.288323	0.370014
2381_52_(	finngen_R	outcome	2381_52_(	Weighted	14	-0.23009	0.247478	0.36945
2418_55_A	finngen_R	outcome	2418_55_A	MR Egger	35	0.147771	0.155234	0.348058
2418_55_A	finngen_R	outcome	2418_55_A	Weighted	35	0.026152	0.105854	0.804861
2418_55_A	finngen_R	outcome	2418_55_A	Inverse va	35	-0.01715	0.072049	0.811808
2418_55_A	finngen_R	outcome	2418_55_A	Simple mc	35	-0.11733	0.183427	0.526696
2418_55_A	finngen_R	outcome	2418_55_A	Weighted	35	0.027513	0.104683	0.794277
2421_7_BL	finngen_R	outcome	2421_7_BL	Inverse va	2	0.373328	0.353596	0.291057
2436_49_(	finngen_R	outcome	2436_49_(	MR Egger	27	-0.0853	0.169619	0.619436
2436_49_(	finngen_R	outcome	2436_49_(	Weighted	27	0.015877	0.127416	0.900832
2436_49_(	finngen_R	outcome	2436_49_(	Inverse va	27	-0.09531	0.085955	0.267505
2436_49_(	finngen_R	outcome	2436_49_(	Simple mc	27	-0.29869	0.23186	0.209018
2436_49_(	finngen_R	outcome	2436_49_(	Weighted	27	-0.0047	0.158094	0.976492
2474_54_A	finngen_R	outcome	2474_54_A	MR Egger	14	0.284684	0.176518	0.132766
2474_54_A	finngen_R	outcome	2474_54_A	Weighted	14	0.184117	0.140335	0.189526
2474_54_A	finngen_R	outcome	2474_54_A	Inverse va	14	0.198016	0.108867	0.068928
2474_54_A	finngen_R	outcome	2474_54_A	Simple mc	14	0.437762	0.232008	0.081714
2474_54_A	finngen_R	outcome	2474_54_A	Weighted	14	0.187776	0.127534	0.164718
2475_1_KI	finngen_R	outcome	2475_1_KI	Wald ratic	1	1.264562	0.75367	0.093372
2480_58_T	finngen_R	outcome	2480_58_T	MR Egger	109	0.041471	0.057017	0.468606
2480_58_T	finngen_R	outcome	2480_58_T	Weighted	109	0.066168	0.054304	0.22305
2480_58_T	finngen_R	outcome	2480_58_T	Inverse va	109	0.053257	0.034132	0.118679
2480_58_T	finngen_R	outcome	2480_58_T	Simple mc	109	0.117806	0.106249	0.269993
2480_58_T	finngen_R	outcome	2480_58_T	Weighted	109	0.088047	0.046388	0.060359
2516_57_(	finngen_R	outcome	2516_57_(	MR Egger	7	-0.08855	0.774876	0.913466

2516_57_(	finngen_R	outcome	2516_57_(	Weighted	7	-0.3256	0.234184	0.164413
2516_57_(	finngen_R	outcome	2516_57_(	Inverse va	7	-0.28409	0.228881	0.214523
2516_57_(	finngen_R	outcome	2516_57_(	Simple mc	7	-0.19771	0.348713	0.591306
2516_57_(	finngen_R	outcome	2516_57_(	Weighted	7	-0.30132	0.274831	0.31496
2567_5_CI	finngen_R	outcome	2567_5_CI	MR Egger	14	-0.37085	0.55207	0.514469
2567_5_CI	finngen_R	outcome	2567_5_CI	Weighted	14	0.092542	0.206678	0.654327
2567_5_CI	finngen_R	outcome	2567_5_CI	Inverse va	14	0.110966	0.159342	0.48618
2567_5_CI	finngen_R	outcome	2567_5_CI	Simple mc	14	0.132249	0.333337	0.697992
2567_5_CI	finngen_R	outcome	2567_5_CI	Weighted	14	0.104324	0.283276	0.718593
2571_12_I	finngen_R	outcome	2571_12_I	MR Egger	37	0.037463	0.100897	0.71265
2571_12_I	finngen_R	outcome	2571_12_I	Weighted	37	0.04453	0.093738	0.634755
2571_12_I	finngen_R	outcome	2571_12_I	Inverse va	37	0.117909	0.06982	0.091265
2571_12_I	finngen_R	outcome	2571_12_I	Simple mc	37	0.160236	0.225063	0.481077
2571_12_I	finngen_R	outcome	2571_12_I	Weighted	37	0.042275	0.089419	0.639229
2579_17_N	finngen_R	outcome	2579_17_N	Wald ratic	1	-0.42665	0.39372	0.278523
2580_83_N	finngen_R	outcome	2580_83_N	MR Egger	42	0.119462	0.07581	0.122945
2580_83_N	finngen_R	outcome	2580_83_N	Weighted	42	0.13016	0.071685	0.069414
2580_83_N	finngen_R	outcome	2580_83_N	Inverse va	42	0.138544	0.051042	0.006641
2580_83_N	finngen_R	outcome	2580_83_N	Simple mc	42	0.094404	0.110762	0.398989
2580_83_N	finngen_R	outcome	2580_83_N	Weighted	42	0.139816	0.065724	0.039456
2590_69_F	finngen_R	outcome	2590_69_F	MR Egger	32	-0.03196	0.139791	0.820731
2590_69_F	finngen_R	outcome	2590_69_F	Weighted	32	-0.12251	0.099137	0.216538
2590_69_F	finngen_R	outcome	2590_69_F	Inverse va	32	-0.02858	0.070921	0.68691
2590_69_F	finngen_R	outcome	2590_69_F	Simple mc	32	-0.14448	0.193742	0.461431
2590_69_F	finngen_R	outcome	2590_69_F	Weighted	32	-0.07033	0.088934	0.435045
2597_8_VE	finngen_R	outcome	2597_8_VE	MR Egger	23	-0.15863	0.196106	0.427654
2597_8_VE	finngen_R	outcome	2597_8_VE	Weighted	23	-0.12493	0.110406	0.257826
2597_8_VE	finngen_R	outcome	2597_8_VE	Inverse va	23	-0.22011	0.10093	0.029201
2597_8_VE	finngen_R	outcome	2597_8_VE	Simple mc	23	-0.25253	0.27095	0.361435
2597_8_VE	finngen_R	outcome	2597_8_VE	Weighted	23	-0.12634	0.112501	0.273521
2602_2_AI	finngen_R	outcome	2602_2_AI	MR Egger	7	0.11594	0.549161	0.841128
2602_2_AI	finngen_R	outcome	2602_2_AI	Weighted	7	-0.14122	0.248539	0.569887
2602_2_AI	finngen_R	outcome	2602_2_AI	Inverse va	7	-0.17112	0.203986	0.401536
2602_2_AI	finngen_R	outcome	2602_2_AI	Simple mc	7	-0.12262	0.373396	0.753783
2602_2_AI	finngen_R	outcome	2602_2_AI	Weighted	7	-0.13257	0.253668	0.619968
2609_59_(	finngen_R	outcome	2609_59_(	MR Egger	21	0.255656	0.185761	0.184747
2609_59_(	finngen_R	outcome	2609_59_(	Weighted	21	0.346268	0.12541	0.005761
2609_59_(	finngen_R	outcome	2609_59_(	Inverse va	21	0.207221	0.089208	0.020186
2609_59_(	finngen_R	outcome	2609_59_(	Simple mc	21	0.2519	0.24804	0.321967
2609_59_(	finngen_R	outcome	2609_59_(	Weighted	21	0.323221	0.138003	0.029634
2611_72_T	finngen_R	outcome	2611_72_T	MR Egger	8	-0.27117	0.311738	0.417809
2611_72_T	finngen_R	outcome	2611_72_T	Weighted	8	-0.27955	0.195118	0.151942
2611_72_T	finngen_R	outcome	2611_72_T	Inverse va	8	-0.20145	0.165304	0.222962
2611_72_T	finngen_R	outcome	2611_72_T	Simple mc	8	-0.32981	0.268517	0.259043
2611_72_T	finngen_R	outcome	2611_72_T	Weighted	8	-0.29699	0.218502	0.216237
2615_60_E	finngen_R	outcome	2615_60_E	MR Egger	11	0.736271	0.626458	0.270033
2615_60_E	finngen_R	outcome	2615_60_E	Weighted	11	-0.1402	0.232567	0.546626
2615_60_E	finngen_R	outcome	2615_60_E	Inverse va	11	-0.07311	0.228495	0.748986
2615_60_E	finngen_R	outcome	2615_60_E	Simple mc	11	-0.44341	0.379684	0.269957
2615_60_E	finngen_R	outcome	2615_60_E	Weighted	11	-0.16338	0.286235	0.58075
2617_56_E	finngen_R	outcome	2617_56_E	Inverse va	2	0.529284	0.319986	0.09811
2620_4_IL(	finngen_R	outcome	2620_4_IL(	MR Egger	30	-0.04478	0.144869	0.75954
2620_4_IL(	finngen_R	outcome	2620_4_IL(	Weighted	30	-0.13317	0.119979	0.267032
2620_4_IL(	finngen_R	outcome	2620_4_IL(	Inverse va	30	0.007649	0.081498	0.925224
2620_4_IL(	finngen_R	outcome	2620_4_IL(	Simple mc	30	-0.06396	0.238123	0.790147
2620_4_IL(	finngen_R	outcome	2620_4_IL(	Weighted	30	-0.08483	0.12865	0.514866
2631_50_II	finngen_R	outcome	2631_50_II	Inverse va	2	0.996673	0.353806	0.004847
2632_5_IL:	finngen_R	outcome	2632_5_IL:	Inverse va	2	0.462447	0.406129	0.254841

2637_77_M	finngen_R	outcome	2637_77_M	MR Egger	100	-0.00526	0.042788	0.90235
2637_77_M	finngen_R	outcome	2637_77_M	Weighted	100	0.059789	0.048239	0.215187
2637_77_M	finngen_R	outcome	2637_77_M	Inverse va	100	-0.01527	0.02855	0.592718
2637_77_M	finngen_R	outcome	2637_77_M	Simple mc	100	-0.01276	0.092474	0.890559
2637_77_M	finngen_R	outcome	2637_77_M	Weighted	100	0.030098	0.040928	0.463837
2644_11_F	finngen_R	outcome	2644_11_F	MR Egger	3	1.138608	1.512958	0.589288
2644_11_F	finngen_R	outcome	2644_11_F	Weighted	3	0.296372	0.354551	0.403208
2644_11_F	finngen_R	outcome	2644_11_F	Inverse va	3	0.328459	0.333238	0.3243
2644_11_F	finngen_R	outcome	2644_11_F	Simple mc	3	-0.06423	0.488996	0.907513
2644_11_F	finngen_R	outcome	2644_11_F	Weighted	3	0.625753	0.458092	0.305261
2652_15_F	finngen_R	outcome	2652_15_F	MR Egger	17	-0.00618	0.269045	0.981977
2652_15_F	finngen_R	outcome	2652_15_F	Weighted	17	-0.00476	0.161872	0.976534
2652_15_F	finngen_R	outcome	2652_15_F	Inverse va	17	-0.13443	0.132892	0.311743
2652_15_F	finngen_R	outcome	2652_15_F	Simple mc	17	0.057005	0.266856	0.833545
2652_15_F	finngen_R	outcome	2652_15_F	Weighted	17	0.019622	0.175576	0.912404
2654_19_T	finngen_R	outcome	2654_19_T	MR Egger	3	0.463024	0.472157	0.506217
2654_19_T	finngen_R	outcome	2654_19_T	Weighted	3	0.11457	0.302214	0.704612
2654_19_T	finngen_R	outcome	2654_19_T	Inverse va	3	0.098596	0.281555	0.7262
2654_19_T	finngen_R	outcome	2654_19_T	Simple mc	3	0.366522	0.43241	0.485907
2654_19_T	finngen_R	outcome	2654_19_T	Weighted	3	0.32577	0.364717	0.465996
2658_27_M	finngen_R	outcome	2658_27_M	MR Egger	24	-0.01122	0.210364	0.957949
2658_27_M	finngen_R	outcome	2658_27_M	Weighted	24	0.024363	0.143006	0.864724
2658_27_M	finngen_R	outcome	2658_27_M	Inverse va	24	0.092937	0.098766	0.346714
2658_27_M	finngen_R	outcome	2658_27_M	Simple mc	24	0.055888	0.257742	0.830248
2658_27_M	finngen_R	outcome	2658_27_M	Weighted	24	-0.09278	0.16959	0.589598
2665_26_T	finngen_R	outcome	2665_26_T	MR Egger	4	-0.23165	0.563826	0.721018
2665_26_T	finngen_R	outcome	2665_26_T	Weighted	4	0.130388	0.230958	0.572379
2665_26_T	finngen_R	outcome	2665_26_T	Inverse va	4	0.193918	0.213188	0.36303
2665_26_T	finngen_R	outcome	2665_26_T	Simple mc	4	0.158852	0.338605	0.670962
2665_26_T	finngen_R	outcome	2665_26_T	Weighted	4	0.12236	0.256151	0.66552
2677_1_EC	finngen_R	outcome	2677_1_EC	MR Egger	11	-0.29718	0.471752	0.544389
2677_1_EC	finngen_R	outcome	2677_1_EC	Weighted	11	-0.47908	0.204858	0.019357
2677_1_EC	finngen_R	outcome	2677_1_EC	Inverse va	11	-0.58693	0.155552	0.000161
2677_1_EC	finngen_R	outcome	2677_1_EC	Simple mc	11	-0.56775	0.298369	0.086217
2677_1_EC	finngen_R	outcome	2677_1_EC	Weighted	11	-0.49905	0.256262	0.080092
2681_23_H	finngen_R	outcome	2681_23_H	Inverse va	2	0.50355	0.280765	0.072894
2683_1_C3	finngen_R	outcome	2683_1_C3	Wald ratic	1	0.731646	0.537305	0.173294
2687_2_M	finngen_R	outcome	2687_2_M	MR Egger	79	-0.00826	0.03756	0.826506
2687_2_M	finngen_R	outcome	2687_2_M	Weighted	79	0.000913	0.042247	0.982757
2687_2_M	finngen_R	outcome	2687_2_M	Inverse va	79	0.024464	0.026672	0.359041
2687_2_M	finngen_R	outcome	2687_2_M	Simple mc	79	0.001957	0.073284	0.978764
2687_2_M	finngen_R	outcome	2687_2_M	Weighted	79	-0.01517	0.036046	0.675018
2692_74_F	finngen_R	outcome	2692_74_F	MR Egger	109	-0.12692	0.059973	0.036632
2692_74_F	finngen_R	outcome	2692_74_F	Weighted	109	-0.11301	0.05222	0.030463
2692_74_F	finngen_R	outcome	2692_74_F	Inverse va	109	-0.07687	0.03299	0.019807
2692_74_F	finngen_R	outcome	2692_74_F	Simple mc	109	-0.07285	0.088253	0.410958
2692_74_F	finngen_R	outcome	2692_74_F	Weighted	109	-0.11298	0.051306	0.029778
2697_7_PF	finngen_R	outcome	2697_7_PF	MR Egger	3	0.827332	1.657217	0.705225
2697_7_PF	finngen_R	outcome	2697_7_PF	Weighted	3	-0.32659	0.324146	0.313668
2697_7_PF	finngen_R	outcome	2697_7_PF	Inverse va	3	-0.31273	0.287873	0.277323
2697_7_PF	finngen_R	outcome	2697_7_PF	Simple mc	3	-0.51424	0.404285	0.331272
2697_7_PF	finngen_R	outcome	2697_7_PF	Weighted	3	-0.50902	0.387652	0.319581
2700_56_F	finngen_R	outcome	2700_56_F	Inverse va	2	0.111142	0.451998	0.805767
2705_5_C(	finngen_R	outcome	2705_5_C(	MR Egger	92	0.024815	0.049415	0.616768
2705_5_C(	finngen_R	outcome	2705_5_C(	Weighted	92	0.041494	0.048484	0.392089
2705_5_C(	finngen_R	outcome	2705_5_C(	Inverse va	92	0.008382	0.032128	0.794165
2705_5_C(	finngen_R	outcome	2705_5_C(	Simple mc	92	-0.17172	0.076878	0.027958
2705_5_C(	finngen_R	outcome	2705_5_C(	Weighted	92	0.032765	0.041372	0.430448

2731_29_F	finngen_R	outcome	2731_29_F	MR Egger	14	0.134621	0.30958	0.671389
2731_29_F	finngen_R	outcome	2731_29_F	Weighted	14	-0.05109	0.170836	0.764913
2731_29_F	finngen_R	outcome	2731_29_F	Inverse va	14	-0.09174	0.140269	0.513092
2731_29_F	finngen_R	outcome	2731_29_F	Simple mc	14	-0.15459	0.265171	0.56987
2731_29_F	finngen_R	outcome	2731_29_F	Weighted	14	-0.0458	0.172939	0.795296
2737_22_N	finngen_R	outcome	2737_22_N	Wald ratic	1	-0.25634	0.579271	0.658113
2741_22_S	finngen_R	outcome	2741_22_S	MR Egger	130	-0.01451	0.044661	0.745835
2741_22_S	finngen_R	outcome	2741_22_S	Weighted	130	-0.00223	0.048802	0.963504
2741_22_S	finngen_R	outcome	2741_22_S	Inverse va	130	0.0051	0.028598	0.858458
2741_22_S	finngen_R	outcome	2741_22_S	Simple mc	130	-0.01926	0.09163	0.833814
2741_22_S	finngen_R	outcome	2741_22_S	Weighted	130	0.007632	0.041397	0.854027
2742_68_S	finngen_R	outcome	2742_68_S	MR Egger	25	-0.16868	0.126532	0.195543
2742_68_S	finngen_R	outcome	2742_68_S	Weighted	25	-0.10866	0.101822	0.285904
2742_68_S	finngen_R	outcome	2742_68_S	Inverse va	25	0.005503	0.072412	0.939421
2742_68_S	finngen_R	outcome	2742_68_S	Simple mc	25	0.02061	0.18018	0.909883
2742_68_S	finngen_R	outcome	2742_68_S	Weighted	25	-0.10963	0.104999	0.306825
2743_5_SH	finngen_R	outcome	2743_5_SH	MR Egger	6	-0.33896	0.716572	0.660854
2743_5_SH	finngen_R	outcome	2743_5_SH	Weighted	6	-0.08594	0.278404	0.757565
2743_5_SH	finngen_R	outcome	2743_5_SH	Inverse va	6	-0.07006	0.220065	0.750196
2743_5_SH	finngen_R	outcome	2743_5_SH	Simple mc	6	-0.10961	0.379766	0.784449
2743_5_SH	finngen_R	outcome	2743_5_SH	Weighted	6	-0.12092	0.334	0.732126
2750_3_AF	finngen_R	outcome	2750_3_AF	MR Egger	24	0.045591	0.169952	0.791
2750_3_AF	finngen_R	outcome	2750_3_AF	Weighted	24	0.09802	0.12263	0.424108
2750_3_AF	finngen_R	outcome	2750_3_AF	Inverse va	24	-0.04637	0.109779	0.672732
2750_3_AF	finngen_R	outcome	2750_3_AF	Simple mc	24	0.236595	0.197898	0.244058
2750_3_AF	finngen_R	outcome	2750_3_AF	Weighted	24	0.162611	0.123384	0.200504
2754_50_C	finngen_R	outcome	2754_50_C	Wald ratic	1	0.619371	0.454853	0.173294
2771_35_I	finngen_R	outcome	2771_35_I	MR Egger	3	-2.79298	9.192493	0.812217
2771_35_I	finngen_R	outcome	2771_35_I	Weighted	3	-0.34061	0.34834	0.328167
2771_35_I	finngen_R	outcome	2771_35_I	Inverse va	3	-0.31493	0.329714	0.339497
2771_35_I	finngen_R	outcome	2771_35_I	Simple mc	3	-0.32763	0.432082	0.527468
2771_35_I	finngen_R	outcome	2771_35_I	Weighted	3	-0.37277	0.444041	0.489547
2774_10_II	finngen_R	outcome	2774_10_II	MR Egger	48	-0.17622	0.110389	0.11725
2774_10_II	finngen_R	outcome	2774_10_II	Weighted	48	-0.15068	0.099165	0.12864
2774_10_II	finngen_R	outcome	2774_10_II	Inverse va	48	-0.11887	0.065191	0.068244
2774_10_II	finngen_R	outcome	2774_10_II	Simple mc	48	-0.01086	0.193143	0.955383
2774_10_II	finngen_R	outcome	2774_10_II	Weighted	48	-0.14078	0.116051	0.231173
2778_10_II	finngen_R	outcome	2778_10_II	Inverse va	2	0.121397	0.678516	0.858004
2780_35_L	finngen_R	outcome	2780_35_L	MR Egger	15	0.27201	0.185663	0.166663
2780_35_L	finngen_R	outcome	2780_35_L	Weighted	15	0.107864	0.14782	0.465573
2780_35_L	finngen_R	outcome	2780_35_L	Inverse va	15	0.109037	0.112013	0.33034
2780_35_L	finngen_R	outcome	2780_35_L	Simple mc	15	0.151754	0.214718	0.491313
2780_35_L	finngen_R	outcome	2780_35_L	Weighted	15	0.110393	0.171143	0.529337
2783_18_C	finngen_R	outcome	2783_18_C	Inverse va	2	0.016029	0.415792	0.969248
2789_26_N	finngen_R	outcome	2789_26_N	MR Egger	34	0.048741	0.119499	0.686082
2789_26_N	finngen_R	outcome	2789_26_N	Weighted	34	0.029033	0.092511	0.75365
2789_26_N	finngen_R	outcome	2789_26_N	Inverse va	34	-0.0642	0.062836	0.306926
2789_26_N	finngen_R	outcome	2789_26_N	Simple mc	34	-0.14197	0.158845	0.377935
2789_26_N	finngen_R	outcome	2789_26_N	Weighted	34	0.010453	0.101142	0.918313
2797_56_A	finngen_R	outcome	2797_56_A	Wald ratic	1	0.147347	0.503387	0.769743
2811_27_A	finngen_R	outcome	2811_27_A	Wald ratic	1	-0.63953	0.525805	0.223873
2813_11_A	finngen_R	outcome	2813_11_A	MR Egger	10	0.033408	0.179783	0.857208
2813_11_A	finngen_R	outcome	2813_11_A	Weighted	10	-0.00313	0.150752	0.983435
2813_11_A	finngen_R	outcome	2813_11_A	Inverse va	10	-0.07618	0.121731	0.531452
2813_11_A	finngen_R	outcome	2813_11_A	Simple mc	10	-0.08436	0.266584	0.758891
2813_11_A	finngen_R	outcome	2813_11_A	Weighted	10	-0.01039	0.148487	0.945747
2816_50_E	finngen_R	outcome	2816_50_E	MR Egger	34	-0.1142	0.094494	0.235711
2816_50_E	finngen_R	outcome	2816_50_E	Weighted	34	-0.21395	0.092255	0.020389

2816_50_E	finngen_R	outcome	2816_50_E	Inverse va	34	-0.18459	0.06367	0.003742
2816_50_E	finngen_R	outcome	2816_50_E	Simple mc	34	-0.08706	0.161937	0.59444
2816_50_E	finngen_R	outcome	2816_50_E	Weighted	34	-0.17961	0.091321	0.057666
2819_23_(	finngen_R	outcome	2819_23_(	MR Egger	7	0.390758	0.551664	0.510389
2819_23_(	finngen_R	outcome	2819_23_(	Weighted	7	0.274909	0.201874	0.173266
2819_23_(	finngen_R	outcome	2819_23_(	Inverse va	7	0.160033	0.16861	0.342553
2819_23_(	finngen_R	outcome	2819_23_(	Simple mc	7	0.322714	0.317817	0.349094
2819_23_(	finngen_R	outcome	2819_23_(	Weighted	7	0.297618	0.222268	0.229066
2827_23_(	finngen_R	outcome	2827_23_(	Inverse va	2	0.130263	0.353966	0.712866
2828_82_S	finngen_R	outcome	2828_82_S	MR Egger	8	0.521276	0.315218	0.149273
2828_82_S	finngen_R	outcome	2828_82_S	Weighted	8	0.207874	0.261991	0.427523
2828_82_S	finngen_R	outcome	2828_82_S	Inverse va	8	0.00557	0.238497	0.981366
2828_82_S	finngen_R	outcome	2828_82_S	Simple mc	8	-0.00287	0.540364	0.995917
2828_82_S	finngen_R	outcome	2828_82_S	Weighted	8	0.46123	0.26012	0.119486
2831_29_k	finngen_R	outcome	2831_29_k	MR Egger	73	0.067005	0.078849	0.3983
2831_29_k	finngen_R	outcome	2831_29_k	Weighted	73	0.07409	0.078608	0.345924
2831_29_k	finngen_R	outcome	2831_29_k	Inverse va	73	0.049768	0.046496	0.284454
2831_29_k	finngen_R	outcome	2831_29_k	Simple mc	73	0.00966	0.129895	0.940924
2831_29_k	finngen_R	outcome	2831_29_k	Weighted	73	0.067836	0.071193	0.343856
2834_54_k	finngen_R	outcome	2834_54_k	MR Egger	29	-0.05071	0.119325	0.674199
2834_54_k	finngen_R	outcome	2834_54_k	Weighted	29	0.053432	0.109475	0.625496
2834_54_k	finngen_R	outcome	2834_54_k	Inverse va	29	0.040481	0.084282	0.631007
2834_54_k	finngen_R	outcome	2834_54_k	Simple mc	29	0.116349	0.217268	0.596525
2834_54_k	finngen_R	outcome	2834_54_k	Weighted	29	0.005243	0.117412	0.964701
2836_68_L	finngen_R	outcome	2836_68_L	Inverse va	2	-0.13732	0.40968	0.737475
2837_3_M	finngen_R	outcome	2837_3_M	MR Egger	16	0.751097	0.483451	0.142585
2837_3_M	finngen_R	outcome	2837_3_M	Weighted	16	0.506051	0.198466	0.010778
2837_3_M	finngen_R	outcome	2837_3_M	Inverse va	16	0.378523	0.149459	0.011321
2837_3_M	finngen_R	outcome	2837_3_M	Simple mc	16	0.428344	0.326299	0.209003
2837_3_M	finngen_R	outcome	2837_3_M	Weighted	16	0.535628	0.234719	0.037506
2843_13_S	finngen_R	outcome	2843_13_S	MR Egger	79	0.124085	0.054188	0.024766
2843_13_S	finngen_R	outcome	2843_13_S	Weighted	79	0.12659	0.052718	0.016338
2843_13_S	finngen_R	outcome	2843_13_S	Inverse va	79	0.096771	0.034244	0.004714
2843_13_S	finngen_R	outcome	2843_13_S	Simple mc	79	0.115513	0.102417	0.262829
2843_13_S	finngen_R	outcome	2843_13_S	Weighted	79	0.115513	0.044713	0.01165
2844_53_T	finngen_R	outcome	2844_53_T	MR Egger	26	-0.03915	0.197454	0.844501
2844_53_T	finngen_R	outcome	2844_53_T	Weighted	26	0.120062	0.12509	0.337154
2844_53_T	finngen_R	outcome	2844_53_T	Inverse va	26	0.16692	0.089253	0.061458
2844_53_T	finngen_R	outcome	2844_53_T	Simple mc	26	0.218286	0.22481	0.340861
2844_53_T	finngen_R	outcome	2844_53_T	Weighted	26	0.100786	0.151714	0.512571
2851_63_(	finngen_R	outcome	2851_63_(	MR Egger	11	0.149612	0.398146	0.715791
2851_63_(	finngen_R	outcome	2851_63_(	Weighted	11	-0.02285	0.219146	0.916949
2851_63_(	finngen_R	outcome	2851_63_(	Inverse va	11	-0.04727	0.172237	0.783728
2851_63_(	finngen_R	outcome	2851_63_(	Simple mc	11	-0.24264	0.319453	0.465057
2851_63_(	finngen_R	outcome	2851_63_(	Weighted	11	-0.03037	0.236478	0.900345
2855_49_N	finngen_R	outcome	2855_49_N	MR Egger	17	0.1733	0.598057	0.775958
2855_49_N	finngen_R	outcome	2855_49_N	Weighted	17	0.051701	0.211975	0.807307
2855_49_N	finngen_R	outcome	2855_49_N	Inverse va	17	-0.02107	0.171597	0.902259
2855_49_N	finngen_R	outcome	2855_49_N	Simple mc	17	0.382688	0.401962	0.355234
2855_49_N	finngen_R	outcome	2855_49_N	Weighted	17	0.199652	0.27242	0.474231
2864_2_M	finngen_R	outcome	2864_2_M	Inverse va	2	-0.22564	1.387199	0.870784
2879_9_SE	finngen_R	outcome	2879_9_SE	MR Egger	21	-0.15192	0.207517	0.473054
2879_9_SE	finngen_R	outcome	2879_9_SE	Weighted	21	-0.04507	0.164355	0.783915
2879_9_SE	finngen_R	outcome	2879_9_SE	Inverse va	21	-0.05187	0.110553	0.638935
2879_9_SE	finngen_R	outcome	2879_9_SE	Simple mc	21	0.269484	0.26156	0.315171
2879_9_SE	finngen_R	outcome	2879_9_SE	Weighted	21	0.029376	0.177015	0.869859
2888_49_(	finngen_R	outcome	2888_49_(	MR Egger	60	0.011752	0.103833	0.910275
2888_49_(	finngen_R	outcome	2888_49_(	Weighted	60	0.115692	0.071051	0.103465

2888_49_(	finngen_R	outcome	2888_49_(	Inverse va	60	0.052807	0.0464	0.255092
2888_49_(	finngen_R	outcome	2888_49_(	Simple mc	60	0.180191	0.137038	0.193631
2888_49_(	finngen_R	outcome	2888_49_(	Weighted	60	0.124643	0.08276	0.137382
2890_59_(	finngen_R	outcome	2890_59_(	Inverse va	2	0.272557	0.483417	0.572881
2900_53_(	finngen_R	outcome	2900_53_(	MR Egger	37	-0.00511	0.084985	0.952399
2900_53_(	finngen_R	outcome	2900_53_(	Weighted	37	-0.02671	0.080849	0.741109
2900_53_(	finngen_R	outcome	2900_53_(	Inverse va	37	-0.01696	0.055959	0.761857
2900_53_(	finngen_R	outcome	2900_53_(	Simple mc	37	-0.03644	0.162264	0.823564
2900_53_(	finngen_R	outcome	2900_53_(	Weighted	37	0.008642	0.073226	0.906715
2911_27_M	finngen_R	outcome	2911_27_M	MR Egger	4	-0.65146	0.683573	0.441162
2911_27_M	finngen_R	outcome	2911_27_M	Weighted	4	-0.02946	0.421794	0.944311
2911_27_M	finngen_R	outcome	2911_27_M	Inverse va	4	-0.12593	0.365835	0.730671
2911_27_M	finngen_R	outcome	2911_27_M	Simple mc	4	0.079957	0.554558	0.894498
2911_27_M	finngen_R	outcome	2911_27_M	Weighted	4	-0.07935	0.509666	0.88617
2913_1_C(	finngen_R	outcome	2913_1_C(	MR Egger	35	-0.06746	0.077824	0.392301
2913_1_C(	finngen_R	outcome	2913_1_C(	Weighted	35	-0.12593	0.068005	0.064048
2913_1_C(	finngen_R	outcome	2913_1_C(	Inverse va	35	-0.08184	0.049645	0.099239
2913_1_C(	finngen_R	outcome	2913_1_C(	Simple mc	35	-0.23501	0.126785	0.072495
2913_1_C(	finngen_R	outcome	2913_1_C(	Weighted	35	-0.13266	0.064583	0.047721
2925_9_SE	finngen_R	outcome	2925_9_SE	MR Egger	3	-3.00118	3.169504	0.517362
2925_9_SE	finngen_R	outcome	2925_9_SE	Weighted	3	0.467269	0.470991	0.321149
2925_9_SE	finngen_R	outcome	2925_9_SE	Inverse va	3	0.311667	0.568266	0.583381
2925_9_SE	finngen_R	outcome	2925_9_SE	Simple mc	3	1.001567	0.680839	0.279097
2925_9_SE	finngen_R	outcome	2925_9_SE	Weighted	3	0.932026	0.714336	0.321912
2944_66_M	finngen_R	outcome	2944_66_M	Wald ratic	1	-0.35426	0.693583	0.609516
2946_52_(	finngen_R	outcome	2946_52_(	MR Egger	32	-0.03335	0.19414	0.864772
2946_52_(	finngen_R	outcome	2946_52_(	Weighted	32	-0.10357	0.123679	0.402356
2946_52_(	finngen_R	outcome	2946_52_(	Inverse va	32	-0.07436	0.088012	0.398153
2946_52_(	finngen_R	outcome	2946_52_(	Simple mc	32	-0.15355	0.202951	0.455007
2946_52_(	finngen_R	outcome	2946_52_(	Weighted	32	-0.17394	0.163551	0.295748
2948_58_(	finngen_R	outcome	2948_58_(	MR Egger	14	-0.16441	0.273199	0.558512
2948_58_(	finngen_R	outcome	2948_58_(	Weighted	14	-0.11727	0.164992	0.477248
2948_58_(	finngen_R	outcome	2948_58_(	Inverse va	14	-0.19243	0.121596	0.11352
2948_58_(	finngen_R	outcome	2948_58_(	Simple mc	14	-0.22333	0.261718	0.408923
2948_58_(	finngen_R	outcome	2948_58_(	Weighted	14	-0.19531	0.193877	0.332138
2950_57_I(	finngen_R	outcome	2950_57_I(	Wald ratic	1	1.326532	0.623788	0.033455
2961_1_PF	finngen_R	outcome	2961_1_PF	MR Egger	15	-0.06424	0.472067	0.893845
2961_1_PF	finngen_R	outcome	2961_1_PF	Weighted	15	0.072399	0.194135	0.7092
2961_1_PF	finngen_R	outcome	2961_1_PF	Inverse va	15	0.007858	0.157345	0.960167
2961_1_PF	finngen_R	outcome	2961_1_PF	Simple mc	15	-0.01224	0.29624	0.967633
2961_1_PF	finngen_R	outcome	2961_1_PF	Weighted	15	0.028881	0.198931	0.886638
2962_50_F	finngen_R	outcome	2962_50_F	MR Egger	7	-0.14146	0.542835	0.804802
2962_50_F	finngen_R	outcome	2962_50_F	Weighted	7	-0.15779	0.262396	0.547602
2962_50_F	finngen_R	outcome	2962_50_F	Inverse va	7	0.029936	0.201961	0.882163
2962_50_F	finngen_R	outcome	2962_50_F	Simple mc	7	-0.15847	0.461102	0.742806
2962_50_F	finngen_R	outcome	2962_50_F	Weighted	7	-0.30081	0.339048	0.40911
2966_65_(	finngen_R	outcome	2966_65_(	MR Egger	17	0.044984	0.146479	0.762988
2966_65_(	finngen_R	outcome	2966_65_(	Weighted	17	-0.00817	0.106777	0.939029
2966_65_(	finngen_R	outcome	2966_65_(	Inverse va	17	0.033864	0.107696	0.753189
2966_65_(	finngen_R	outcome	2966_65_(	Simple mc	17	0.292768	0.266579	0.288352
2966_65_(	finngen_R	outcome	2966_65_(	Weighted	17	-0.00844	0.102335	0.935309
2968_61_T	finngen_R	outcome	2968_61_T	Wald ratic	1	-0.29128	0.785804	0.710879
2972_57_E	finngen_R	outcome	2972_57_E	Wald ratic	1	0.312896	0.637471	0.62354
2973_15_(	finngen_R	outcome	2973_15_(	MR Egger	20	-0.25519	0.204387	0.227821
2973_15_(	finngen_R	outcome	2973_15_(	Weighted	20	-0.07299	0.12887	0.571112
2973_15_(	finngen_R	outcome	2973_15_(	Inverse va	20	0.005994	0.098347	0.951401
2973_15_(	finngen_R	outcome	2973_15_(	Simple mc	20	-0.07987	0.204659	0.700678
2973_15_(	finngen_R	outcome	2973_15_(	Weighted	20	-0.07987	0.13618	0.564432

2974_61_(	finngen_R	outcome	2974_61_(	MR Egger	49	-0.42714	0.158642	0.009796
2974_61_(	finngen_R	outcome	2974_61_(	Weighted	49	-0.2158	0.091033	0.017758
2974_61_(	finngen_R	outcome	2974_61_(	Inverse va	49	-0.08225	0.078575	0.295228
2974_61_(	finngen_R	outcome	2974_61_(	Simple mc	49	-0.15159	0.206203	0.465828
2974_61_(	finngen_R	outcome	2974_61_(	Weighted	49	-0.27212	0.096739	0.007093
2977_7_EI	finngen_R	outcome	2977_7_EI	MR Egger	41	0.023952	0.090671	0.793043
2977_7_EI	finngen_R	outcome	2977_7_EI	Weighted	41	0.054976	0.086712	0.526075
2977_7_EI	finngen_R	outcome	2977_7_EI	Inverse va	41	0.011645	0.062598	0.85242
2977_7_EI	finngen_R	outcome	2977_7_EI	Simple mc	41	-0.00588	0.170969	0.97273
2977_7_EI	finngen_R	outcome	2977_7_EI	Weighted	41	-0.00588	0.08461	0.944931
2979_8_C>	finngen_R	outcome	2979_8_C>	MR Egger	6	0.147962	0.460971	0.764297
2979_8_C>	finngen_R	outcome	2979_8_C>	Weighted	6	-0.2136	0.241715	0.376875
2979_8_C>	finngen_R	outcome	2979_8_C>	Inverse va	6	-0.18709	0.210938	0.375113
2979_8_C>	finngen_R	outcome	2979_8_C>	Simple mc	6	-0.54753	0.396108	0.225446
2979_8_C>	finngen_R	outcome	2979_8_C>	Weighted	6	-0.17961	0.266755	0.530605
2985_35_(	finngen_R	outcome	2985_35_(	MR Egger	74	-0.0489	0.049742	0.328838
2985_35_(	finngen_R	outcome	2985_35_(	Weighted	74	-0.04819	0.052314	0.356949
2985_35_(	finngen_R	outcome	2985_35_(	Inverse va	74	-0.01142	0.035854	0.75
2985_35_(	finngen_R	outcome	2985_35_(	Simple mc	74	-0.02052	0.09765	0.834114
2985_35_(	finngen_R	outcome	2985_35_(	Weighted	74	-0.04512	0.044807	0.317296
2991_9_IL:	finngen_R	outcome	2991_9_IL:	MR Egger	27	-0.22522	0.200781	0.272644
2991_9_IL:	finngen_R	outcome	2991_9_IL:	Weighted	27	-0.08112	0.130129	0.533011
2991_9_IL:	finngen_R	outcome	2991_9_IL:	Inverse va	27	0.064236	0.092088	0.485455
2991_9_IL:	finngen_R	outcome	2991_9_IL:	Simple mc	27	-0.04591	0.202911	0.822774
2991_9_IL:	finngen_R	outcome	2991_9_IL:	Weighted	27	-0.04591	0.141326	0.747897
2992_59_I	finngen_R	outcome	2992_59_I	MR Egger	157	-0.00821	0.032892	0.803224
2992_59_I	finngen_R	outcome	2992_59_I	Weighted	157	0.01112	0.030729	0.717451
2992_59_I	finngen_R	outcome	2992_59_I	Inverse va	157	0.004327	0.019963	0.828413
2992_59_I	finngen_R	outcome	2992_59_I	Simple mc	157	-0.02003	0.060938	0.74286
2992_59_I	finngen_R	outcome	2992_59_I	Weighted	157	-0.00242	0.028564	0.932524
2993_1_IL:	finngen_R	outcome	2993_1_IL:	Inverse va	2	-0.01693	0.394508	0.965777
2994_71_I	finngen_R	outcome	2994_71_I	MR Egger	16	-0.42416	0.281036	0.15346
2994_71_I	finngen_R	outcome	2994_71_I	Weighted	16	-0.28346	0.154805	0.067093
2994_71_I	finngen_R	outcome	2994_71_I	Inverse va	16	-0.40635	0.129382	0.001685
2994_71_I	finngen_R	outcome	2994_71_I	Simple mc	16	-0.63503	0.242949	0.019552
2994_71_I	finngen_R	outcome	2994_71_I	Weighted	16	-0.31795	0.151587	0.053311
3000_66_N	finngen_R	outcome	3000_66_N	MR Egger	153	-0.00299	0.035975	0.933806
3000_66_N	finngen_R	outcome	3000_66_N	Weighted	153	-0.00381	0.036628	0.917093
3000_66_N	finngen_R	outcome	3000_66_N	Inverse va	153	0.011479	0.021933	0.600715
3000_66_N	finngen_R	outcome	3000_66_N	Simple mc	153	-0.04684	0.066223	0.480448
3000_66_N	finngen_R	outcome	3000_66_N	Weighted	153	-0.00405	0.032339	0.900593
3004_67_F	finngen_R	outcome	3004_67_F	MR Egger	46	0.139456	0.114957	0.231558
3004_67_F	finngen_R	outcome	3004_67_F	Weighted	46	0.17133	0.09455	0.069977
3004_67_F	finngen_R	outcome	3004_67_F	Inverse va	46	0.192759	0.059596	0.001219
3004_67_F	finngen_R	outcome	3004_67_F	Simple mc	46	0.338411	0.170918	0.053843
3004_67_F	finngen_R	outcome	3004_67_F	Weighted	46	0.168676	0.094518	0.081072
3007_7_SI	finngen_R	outcome	3007_7_SI	MR Egger	179	0.00899	0.030081	0.765391
3007_7_SI	finngen_R	outcome	3007_7_SI	Weighted	179	0.033416	0.032461	0.303278
3007_7_SI	finngen_R	outcome	3007_7_SI	Inverse va	179	0.008505	0.018488	0.645509
3007_7_SI	finngen_R	outcome	3007_7_SI	Simple mc	179	0.046647	0.062373	0.455523
3007_7_SI	finngen_R	outcome	3007_7_SI	Weighted	179	0.039143	0.029772	0.190275
3009_3_TC	finngen_R	outcome	3009_3_TC	MR Egger	8	-0.35322	0.612	0.584826
3009_3_TC	finngen_R	outcome	3009_3_TC	Weighted	8	-0.03171	0.230184	0.890437
3009_3_TC	finngen_R	outcome	3009_3_TC	Inverse va	8	-0.09583	0.184904	0.604278
3009_3_TC	finngen_R	outcome	3009_3_TC	Simple mc	8	0.045202	0.381398	0.908987
3009_3_TC	finngen_R	outcome	3009_3_TC	Weighted	8	0.112261	0.304068	0.722894
3024_18_S	finngen_R	outcome	3024_18_S	MR Egger	17	-0.23968	0.329584	0.478295
3024_18_S	finngen_R	outcome	3024_18_S	Weighted	17	0.128388	0.184428	0.486341

3024_18_S	finngen_R	outcome	3024_18_S	Inverse va	17	0.196444	0.143258	0.170294
3024_18_S	finngen_R	outcome	3024_18_S	Simple mc	17	0.270257	0.307972	0.39318
3024_18_S	finngen_R	outcome	3024_18_S	Weighted	17	0.06847	0.195332	0.730512
3025_50_F	finngen_R	outcome	3025_50_F	MR Egger	81	0.154802	0.082161	0.063228
3025_50_F	finngen_R	outcome	3025_50_F	Weighted	81	0.132407	0.073417	0.071311
3025_50_F	finngen_R	outcome	3025_50_F	Inverse va	81	0.122723	0.045543	0.007047
3025_50_F	finngen_R	outcome	3025_50_F	Simple mc	81	0.214343	0.137793	0.123765
3025_50_F	finngen_R	outcome	3025_50_F	Weighted	81	0.147873	0.063209	0.021808
3028_36_C	finngen_R	outcome	3028_36_C	MR Egger	48	-0.00134	0.052067	0.979579
3028_36_C	finngen_R	outcome	3028_36_C	Weighted	48	-0.06347	0.050886	0.212288
3028_36_C	finngen_R	outcome	3028_36_C	Inverse va	48	-0.06022	0.037511	0.108397
3028_36_C	finngen_R	outcome	3028_36_C	Simple mc	48	-0.13352	0.110299	0.232125
3028_36_C	finngen_R	outcome	3028_36_C	Weighted	48	-0.06041	0.039404	0.131931
3029_52_C	finngen_R	outcome	3029_52_C	MR Egger	33	0.011088	0.099285	0.9118
3029_52_C	finngen_R	outcome	3029_52_C	Weighted	33	0.019088	0.083384	0.818934
3029_52_C	finngen_R	outcome	3029_52_C	Inverse va	33	0.058747	0.059319	0.321994
3029_52_C	finngen_R	outcome	3029_52_C	Simple mc	33	0.058154	0.157022	0.713557
3029_52_C	finngen_R	outcome	3029_52_C	Weighted	33	0.015537	0.068581	0.82222
3038_9_C)	finngen_R	outcome	3038_9_C)	MR Egger	45	-0.1787	0.130797	0.178976
3038_9_C)	finngen_R	outcome	3038_9_C)	Weighted	45	0.001532	0.098773	0.987626
3038_9_C)	finngen_R	outcome	3038_9_C)	Inverse va	45	0.011374	0.069014	0.869093
3038_9_C)	finngen_R	outcome	3038_9_C)	Simple mc	45	0.214852	0.193032	0.271737
3038_9_C)	finngen_R	outcome	3038_9_C)	Weighted	45	0.004046	0.089729	0.964242
3040_59_C	finngen_R	outcome	3040_59_C	MR Egger	10	-0.05732	0.117164	0.637832
3040_59_C	finngen_R	outcome	3040_59_C	Weighted	10	-0.03784	0.098021	0.699504
3040_59_C	finngen_R	outcome	3040_59_C	Inverse va	10	-0.05786	0.076935	0.452007
3040_59_C	finngen_R	outcome	3040_59_C	Simple mc	10	-0.30015	0.174937	0.120344
3040_59_C	finngen_R	outcome	3040_59_C	Weighted	10	0.02091	0.098703	0.836946
3041_55_N	finngen_R	outcome	3041_55_N	MR Egger	20	-0.17318	0.180366	0.349705
3041_55_N	finngen_R	outcome	3041_55_N	Weighted	20	-0.01895	0.141537	0.893476
3041_55_N	finngen_R	outcome	3041_55_N	Inverse va	20	-0.13121	0.101886	0.197825
3041_55_N	finngen_R	outcome	3041_55_N	Simple mc	20	0.002367	0.237917	0.992165
3041_55_N	finngen_R	outcome	3041_55_N	Weighted	20	0.021468	0.196329	0.914073
3043_49_S	finngen_R	outcome	3043_49_S	Wald ratic	1	1.236276	1.396476	0.376004
3044_3_C(	finngen_R	outcome	3044_3_C(	MR Egger	49	-0.00854	0.055314	0.877932
3044_3_C(	finngen_R	outcome	3044_3_C(	Weighted	49	0.010153	0.05486	0.853169
3044_3_C(	finngen_R	outcome	3044_3_C(	Inverse va	49	-0.00901	0.040462	0.823737
3044_3_C(	finngen_R	outcome	3044_3_C(	Simple mc	49	-0.03659	0.11012	0.741152
3044_3_C(	finngen_R	outcome	3044_3_C(	Weighted	49	-0.00478	0.047273	0.919819
3045_72_F	finngen_R	outcome	3045_72_F	MR Egger	22	-0.01985	0.231325	0.93248
3045_72_F	finngen_R	outcome	3045_72_F	Weighted	22	0.023552	0.156509	0.880386
3045_72_F	finngen_R	outcome	3045_72_F	Inverse va	22	-0.06908	0.110999	0.533725
3045_72_F	finngen_R	outcome	3045_72_F	Simple mc	22	0.097618	0.268626	0.719941
3045_72_F	finngen_R	outcome	3045_72_F	Weighted	22	0.030166	0.158975	0.851327
3046_31_F	finngen_R	outcome	3046_31_F	MR Egger	50	-0.17822	0.109826	0.111204
3046_31_F	finngen_R	outcome	3046_31_F	Weighted	50	-0.1738	0.086684	0.044961
3046_31_F	finngen_R	outcome	3046_31_F	Inverse va	50	-0.10367	0.06183	0.093614
3046_31_F	finngen_R	outcome	3046_31_F	Simple mc	50	-0.21923	0.15207	0.155757
3046_31_F	finngen_R	outcome	3046_31_F	Weighted	50	-0.16986	0.110422	0.130405
3049_61_F	finngen_R	outcome	3049_61_F	Wald ratic	1	0.222267	0.718141	0.756939
3050_7_V\	finngen_R	outcome	3050_7_V\	MR Egger	7	0.285291	0.433936	0.539947
3050_7_V\	finngen_R	outcome	3050_7_V\	Weighted	7	0.077815	0.214795	0.717147
3050_7_V\	finngen_R	outcome	3050_7_V\	Inverse va	7	-0.0094	0.178492	0.957984
3050_7_V\	finngen_R	outcome	3050_7_V\	Simple mc	7	0.036116	0.279787	0.90151
3050_7_V\	finngen_R	outcome	3050_7_V\	Weighted	7	0.075627	0.25442	0.776297
3054_3_HI	finngen_R	outcome	3054_3_HI	MR Egger	81	0.026454	0.046846	0.573874
3054_3_HI	finngen_R	outcome	3054_3_HI	Weighted	81	-0.00484	0.039894	0.903348
3054_3_HI	finngen_R	outcome	3054_3_HI	Inverse va	81	-0.00687	0.025963	0.791243

3054_3_HI	finngen_R	outcome	3054_3_HI	Simple mc	81	0.004011	0.067476	0.952744
3054_3_HI	finngen_R	outcome	3054_3_HI	Weighted	81	0.004011	0.044595	0.928552
3059_50_T	finngen_R	outcome	3059_50_T	MR Egger	3	1.333659	0.841723	0.358417
3059_50_T	finngen_R	outcome	3059_50_T	Weighted	3	-0.96193	0.480877	0.04546
3059_50_T	finngen_R	outcome	3059_50_T	Inverse va	3	-0.62663	0.685617	0.360733
3059_50_T	finngen_R	outcome	3059_50_T	Simple mc	3	-1.41197	0.819807	0.227153
3059_50_T	finngen_R	outcome	3059_50_T	Weighted	3	-1.45281	1.059089	0.30375
3060_43_C	finngen_R	outcome	3060_43_C	MR Egger	19	-0.03493	0.166944	0.836756
3060_43_C	finngen_R	outcome	3060_43_C	Weighted	19	-0.01821	0.141011	0.897256
3060_43_C	finngen_R	outcome	3060_43_C	Inverse va	19	0.020067	0.098285	0.838216
3060_43_C	finngen_R	outcome	3060_43_C	Simple mc	19	-0.09161	0.234062	0.700106
3060_43_C	finngen_R	outcome	3060_43_C	Weighted	19	-0.08667	0.179605	0.635218
3066_12_L	finngen_R	outcome	3066_12_L	MR Egger	23	0.031697	0.149494	0.834131
3066_12_L	finngen_R	outcome	3066_12_L	Weighted	23	0.010966	0.113025	0.922711
3066_12_L	finngen_R	outcome	3066_12_L	Inverse va	23	-0.00853	0.089504	0.924114
3066_12_L	finngen_R	outcome	3066_12_L	Simple mc	23	-0.19563	0.194203	0.324726
3066_12_L	finngen_R	outcome	3066_12_L	Weighted	23	0.019531	0.109283	0.859797
3074_6_LE	finngen_R	outcome	3074_6_LE	MR Egger	80	-0.00055	0.052646	0.991662
3074_6_LE	finngen_R	outcome	3074_6_LE	Weighted	80	0.003172	0.046001	0.945027
3074_6_LE	finngen_R	outcome	3074_6_LE	Inverse va	80	0.000149	0.031799	0.996269
3074_6_LE	finngen_R	outcome	3074_6_LE	Simple mc	80	0.041769	0.086887	0.632036
3074_6_LE	finngen_R	outcome	3074_6_LE	Weighted	80	-0.01188	0.047977	0.805112
3077_66_F	finngen_R	outcome	3077_66_F	MR Egger	15	0.262001	0.2805	0.367303
3077_66_F	finngen_R	outcome	3077_66_F	Weighted	15	0.126776	0.194359	0.514222
3077_66_F	finngen_R	outcome	3077_66_F	Inverse va	15	0.159382	0.141174	0.258909
3077_66_F	finngen_R	outcome	3077_66_F	Simple mc	15	0.205895	0.293561	0.494566
3077_66_F	finngen_R	outcome	3077_66_F	Weighted	15	0.185825	0.207858	0.386433
3132_1_VE	finngen_R	outcome	3132_1_VE	Wald ratic	1	0.015375	0.184937	0.933745
3143_3_CI	finngen_R	outcome	3143_3_CI	Wald ratic	1	0.075238	0.706007	0.915131
3151_6_IL:	finngen_R	outcome	3151_6_IL:	Wald ratic	1	0.717015	0.643978	0.26553
3152_57_T	finngen_R	outcome	3152_57_T	MR Egger	6	0.606167	0.35053	0.158815
3152_57_T	finngen_R	outcome	3152_57_T	Weighted	6	0.487549	0.268705	0.069611
3152_57_T	finngen_R	outcome	3152_57_T	Inverse va	6	0.393349	0.212691	0.064401
3152_57_T	finngen_R	outcome	3152_57_T	Simple mc	6	0.692064	0.364535	0.116089
3152_57_T	finngen_R	outcome	3152_57_T	Weighted	6	0.453398	0.344508	0.245253
3166_92_C	finngen_R	outcome	3166_92_C	MR Egger	182	-0.10196	0.033918	0.003024
3166_92_C	finngen_R	outcome	3166_92_C	Weighted	182	-0.04888	0.032935	0.137812
3166_92_C	finngen_R	outcome	3166_92_C	Inverse va	182	-0.05107	0.020622	0.013273
3166_92_C	finngen_R	outcome	3166_92_C	Simple mc	182	-0.08076	0.067409	0.23244
3166_92_C	finngen_R	outcome	3166_92_C	Weighted	182	-0.07408	0.031901	0.021341
3168_8_AI	finngen_R	outcome	3168_8_AI	MR Egger	20	-0.11934	0.140971	0.408349
3168_8_AI	finngen_R	outcome	3168_8_AI	Weighted	20	-0.0163	0.11569	0.887978
3168_8_AI	finngen_R	outcome	3168_8_AI	Inverse va	20	-0.0876	0.084975	0.302588
3168_8_AI	finngen_R	outcome	3168_8_AI	Simple mc	20	-0.03468	0.226763	0.880047
3168_8_AI	finngen_R	outcome	3168_8_AI	Weighted	20	0.020652	0.131152	0.876538
3171_57_A	finngen_R	outcome	3171_57_A	Inverse va	2	0.194948	0.380691	0.608588
3173_49_N	finngen_R	outcome	3173_49_N	MR Egger	89	-0.06897	0.047012	0.145972
3173_49_N	finngen_R	outcome	3173_49_N	Weighted	89	-0.04648	0.05192	0.370702
3173_49_N	finngen_R	outcome	3173_49_N	Inverse va	89	-0.01925	0.029346	0.511857
3173_49_N	finngen_R	outcome	3173_49_N	Simple mc	89	0.015124	0.107195	0.888119
3173_49_N	finngen_R	outcome	3173_49_N	Weighted	89	-0.04136	0.047519	0.386422
3175_51_A	finngen_R	outcome	3175_51_A	MR Egger	139	-0.09966	0.049197	0.044724
3175_51_A	finngen_R	outcome	3175_51_A	Weighted	139	-0.08542	0.05107	0.094393
3175_51_A	finngen_R	outcome	3175_51_A	Inverse va	139	-0.05685	0.030342	0.060995
3175_51_A	finngen_R	outcome	3175_51_A	Simple mc	139	-0.0446	0.113906	0.695972
3175_51_A	finngen_R	outcome	3175_51_A	Weighted	139	-0.10117	0.052095	0.054161
3181_50_C	finngen_R	outcome	3181_50_C	MR Egger	34	-0.09822	0.111368	0.384386
3181_50_C	finngen_R	outcome	3181_50_C	Weighted	34	-0.04365	0.080655	0.58837

3181_50_(	finngen_R	outcome	3181_50_(	Inverse va	34	-0.01377	0.059332	0.816513
3181_50_(	finngen_R	outcome	3181_50_(	Simple mc	34	0.006295	0.126611	0.960646
3181_50_(	finngen_R	outcome	3181_50_(	Weighted	34	-0.05071	0.082123	0.54117
3184_25_F	finngen_R	outcome	3184_25_F	MR Egger	97	0.059972	0.042507	0.161544
3184_25_F	finngen_R	outcome	3184_25_F	Weighted	97	0.044465	0.044193	0.314336
3184_25_F	finngen_R	outcome	3184_25_F	Inverse va	97	0.082391	0.027057	0.002326
3184_25_F	finngen_R	outcome	3184_25_F	Simple mc	97	0.17413	0.071091	0.016122
3184_25_F	finngen_R	outcome	3184_25_F	Weighted	97	0.077321	0.037512	0.041986
3191_50_V	finngen_R	outcome	3191_50_V	MR Egger	8	-0.08237	0.502936	0.875276
3191_50_V	finngen_R	outcome	3191_50_V	Weighted	8	0.033441	0.203681	0.869586
3191_50_V	finngen_R	outcome	3191_50_V	Inverse va	8	0.093972	0.168633	0.577351
3191_50_V	finngen_R	outcome	3191_50_V	Simple mc	8	0.113333	0.301649	0.718259
3191_50_V	finngen_R	outcome	3191_50_V	Weighted	8	-0.01727	0.221933	0.940142
3195_50_(	finngen_R	outcome	3195_50_(	MR Egger	90	0.049589	0.048305	0.307432
3195_50_(	finngen_R	outcome	3195_50_(	Weighted	90	0.086494	0.050648	0.087686
3195_50_(	finngen_R	outcome	3195_50_(	Inverse va	90	0.011577	0.031145	0.710093
3195_50_(	finngen_R	outcome	3195_50_(	Simple mc	90	-0.00905	0.094579	0.923979
3195_50_(	finngen_R	outcome	3195_50_(	Weighted	90	0.058755	0.042243	0.167724
3196_6_H/	finngen_R	outcome	3196_6_H/	MR Egger	12	-0.1913	0.503687	0.712041
3196_6_H/	finngen_R	outcome	3196_6_H/	Weighted	12	0.06245	0.216964	0.773471
3196_6_H/	finngen_R	outcome	3196_6_H/	Inverse va	12	0.150503	0.170193	0.37653
3196_6_H/	finngen_R	outcome	3196_6_H/	Simple mc	12	0.056744	0.307469	0.856938
3196_6_H/	finngen_R	outcome	3196_6_H/	Weighted	12	-0.00688	0.268367	0.980003
3199_54_k	finngen_R	outcome	3199_54_k	Inverse va	2	0.497495	0.229866	0.030442
3210_1_M	finngen_R	outcome	3210_1_M	Wald ratic	1	-1.1847	0.53291	0.02621
3213_65_N	finngen_R	outcome	3213_65_N	MR Egger	20	0.140461	0.218269	0.528
3213_65_N	finngen_R	outcome	3213_65_N	Weighted	20	-0.14155	0.170179	0.405539
3213_65_N	finngen_R	outcome	3213_65_N	Inverse va	20	-0.18112	0.137404	0.187458
3213_65_N	finngen_R	outcome	3213_65_N	Simple mc	20	-0.11694	0.404361	0.775565
3213_65_N	finngen_R	outcome	3213_65_N	Weighted	20	0.027687	0.163257	0.867126
3216_2_Pl	finngen_R	outcome	3216_2_Pl	MR Egger	4	-0.10995	0.916108	0.915436
3216_2_Pl	finngen_R	outcome	3216_2_Pl	Weighted	4	-0.08829	0.197539	0.654895
3216_2_Pl	finngen_R	outcome	3216_2_Pl	Inverse va	4	-0.05539	0.186027	0.765875
3216_2_Pl	finngen_R	outcome	3216_2_Pl	Simple mc	4	-0.07265	0.285239	0.815419
3216_2_Pl	finngen_R	outcome	3216_2_Pl	Weighted	4	-0.11415	0.231836	0.656226
3220_40_F	finngen_R	outcome	3220_40_F	MR Egger	48	0.242329	0.090544	0.010278
3220_40_F	finngen_R	outcome	3220_40_F	Weighted	48	0.142397	0.072465	0.049408
3220_40_F	finngen_R	outcome	3220_40_F	Inverse va	48	0.104763	0.047165	0.026336
3220_40_F	finngen_R	outcome	3220_40_F	Simple mc	48	0.118439	0.111288	0.292652
3220_40_F	finngen_R	outcome	3220_40_F	Weighted	48	0.154307	0.069664	0.031642
3221_54_S	finngen_R	outcome	3221_54_S	MR Egger	27	0.059913	0.260057	0.819668
3221_54_S	finngen_R	outcome	3221_54_S	Weighted	27	0.106254	0.148098	0.473092
3221_54_S	finngen_R	outcome	3221_54_S	Inverse va	27	0.123199	0.094289	0.191344
3221_54_S	finngen_R	outcome	3221_54_S	Simple mc	27	-0.07329	0.270981	0.788953
3221_54_S	finngen_R	outcome	3221_54_S	Weighted	27	-0.0282	0.197825	0.887736
3232_28_A	finngen_R	outcome	3232_28_A	MR Egger	17	-0.27991	0.190025	0.161421
3232_28_A	finngen_R	outcome	3232_28_A	Weighted	17	-0.21374	0.105284	0.042348
3232_28_A	finngen_R	outcome	3232_28_A	Inverse va	17	-0.18931	0.084793	0.025577
3232_28_A	finngen_R	outcome	3232_28_A	Simple mc	17	-0.30724	0.160408	0.073492
3232_28_A	finngen_R	outcome	3232_28_A	Weighted	17	-0.22874	0.11847	0.07143
3234_23_(	finngen_R	outcome	3234_23_(	MR Egger	5	0.050127	1.131647	0.967452
3234_23_(	finngen_R	outcome	3234_23_(	Weighted	5	0.697673	0.297571	0.01905
3234_23_(	finngen_R	outcome	3234_23_(	Inverse va	5	0.550441	0.250456	0.027967
3234_23_(	finngen_R	outcome	3234_23_(	Simple mc	5	0.618855	0.39564	0.19282
3234_23_(	finngen_R	outcome	3234_23_(	Weighted	5	0.677624	0.326026	0.10621
3235_50_V	finngen_R	outcome	3235_50_V	MR Egger	72	0.179533	0.065993	0.008216
3235_50_V	finngen_R	outcome	3235_50_V	Weighted	72	0.137247	0.066934	0.040316
3235_50_V	finngen_R	outcome	3235_50_V	Inverse va	72	0.046883	0.042698	0.2722

3235_50_V	finngen_R	outcome	3235_50_V	Simple mc	72	0.037648	0.113664	0.74145
3235_50_V	finngen_R	outcome	3235_50_V	Weighted	72	0.12806	0.057603	0.029389
3280_49_A	finngen_R	outcome	3280_49_A	MR Egger	3	-1.00637	0.685182	0.380543
3280_49_A	finngen_R	outcome	3280_49_A	Weighted	3	-0.69866	0.306846	0.022792
3280_49_A	finngen_R	outcome	3280_49_A	Inverse va	3	-0.62496	0.288757	0.030441
3280_49_A	finngen_R	outcome	3280_49_A	Simple mc	3	-0.71937	0.41709	0.226716
3280_49_A	finngen_R	outcome	3280_49_A	Weighted	3	-0.70642	0.364987	0.192576
3283_21_T	finngen_R	outcome	3283_21_T	MR Egger	74	0.051521	0.074193	0.489656
3283_21_T	finngen_R	outcome	3283_21_T	Weighted	74	-0.00168	0.061423	0.978241
3283_21_T	finngen_R	outcome	3283_21_T	Inverse va	74	-0.01979	0.041776	0.635709
3283_21_T	finngen_R	outcome	3283_21_T	Simple mc	74	-0.06038	0.134249	0.654198
3283_21_T	finngen_R	outcome	3283_21_T	Weighted	74	0.022726	0.061229	0.711594
3285_23_C	finngen_R	outcome	3285_23_C	MR Egger	4	0.054982	0.756794	0.948696
3285_23_C	finngen_R	outcome	3285_23_C	Weighted	4	0.377252	0.374595	0.31389
3285_23_C	finngen_R	outcome	3285_23_C	Inverse va	4	0.243094	0.352511	0.490442
3285_23_C	finngen_R	outcome	3285_23_C	Simple mc	4	0.511922	0.501096	0.382165
3285_23_C	finngen_R	outcome	3285_23_C	Weighted	4	0.359221	0.440488	0.474509
3290_50_C	finngen_R	outcome	3290_50_C	MR Egger	97	0.001599	0.060672	0.979035
3290_50_C	finngen_R	outcome	3290_50_C	Weighted	97	0.003059	0.051113	0.952283
3290_50_C	finngen_R	outcome	3290_50_C	Inverse va	97	0.007902	0.033282	0.812322
3290_50_C	finngen_R	outcome	3290_50_C	Simple mc	97	0.060782	0.083388	0.467833
3290_50_C	finngen_R	outcome	3290_50_C	Weighted	97	-0.00713	0.046892	0.879457
3291_30_F	finngen_R	outcome	3291_30_F	MR Egger	47	-0.0471	0.105753	0.658186
3291_30_F	finngen_R	outcome	3291_30_F	Weighted	47	0.015879	0.086354	0.854102
3291_30_F	finngen_R	outcome	3291_30_F	Inverse va	47	0.013915	0.060404	0.81781
3291_30_F	finngen_R	outcome	3291_30_F	Simple mc	47	0.041121	0.154368	0.791137
3291_30_F	finngen_R	outcome	3291_30_F	Weighted	47	0.02167	0.079245	0.785728
3292_75_C	finngen_R	outcome	3292_75_C	MR Egger	25	-0.24699	0.145344	0.102744
3292_75_C	finngen_R	outcome	3292_75_C	Weighted	25	-0.1123	0.114742	0.32773
3292_75_C	finngen_R	outcome	3292_75_C	Inverse va	25	-0.04031	0.082578	0.625435
3292_75_C	finngen_R	outcome	3292_75_C	Simple mc	25	-0.01956	0.19294	0.92009
3292_75_C	finngen_R	outcome	3292_75_C	Weighted	25	-0.09199	0.116951	0.439256
3293_2_CI	finngen_R	outcome	3293_2_CI	MR Egger	50	-0.13327	0.119464	0.27016
3293_2_CI	finngen_R	outcome	3293_2_CI	Weighted	50	-0.02035	0.098183	0.835823
3293_2_CI	finngen_R	outcome	3293_2_CI	Inverse va	50	-0.00797	0.060165	0.894653
3293_2_CI	finngen_R	outcome	3293_2_CI	Simple mc	50	0.036553	0.174041	0.834519
3293_2_CI	finngen_R	outcome	3293_2_CI	Weighted	50	-0.02678	0.09406	0.777061
3296_92_C	finngen_R	outcome	3296_92_C	MR Egger	119	0.019103	0.048921	0.696889
3296_92_C	finngen_R	outcome	3296_92_C	Weighted	119	-0.01147	0.043562	0.792278
3296_92_C	finngen_R	outcome	3296_92_C	Inverse va	119	-0.01303	0.029539	0.659226
3296_92_C	finngen_R	outcome	3296_92_C	Simple mc	119	-0.13524	0.087164	0.123448
3296_92_C	finngen_R	outcome	3296_92_C	Weighted	119	-0.02775	0.038272	0.469804
3298_52_C	finngen_R	outcome	3298_52_C	MR Egger	69	0.040244	0.103489	0.698602
3298_52_C	finngen_R	outcome	3298_52_C	Weighted	69	0.057488	0.082279	0.484741
3298_52_C	finngen_R	outcome	3298_52_C	Inverse va	69	0.027325	0.053132	0.607046
3298_52_C	finngen_R	outcome	3298_52_C	Simple mc	69	-0.14604	0.146154	0.321233
3298_52_C	finngen_R	outcome	3298_52_C	Weighted	69	-0.01055	0.084217	0.900647
3302_58_C	finngen_R	outcome	3302_58_C	MR Egger	80	-0.01788	0.066897	0.789934
3302_58_C	finngen_R	outcome	3302_58_C	Weighted	80	-0.03944	0.055696	0.47885
3302_58_C	finngen_R	outcome	3302_58_C	Inverse va	80	-0.00877	0.035487	0.804838
3302_58_C	finngen_R	outcome	3302_58_C	Simple mc	80	-0.02937	0.097594	0.764252
3302_58_C	finngen_R	outcome	3302_58_C	Weighted	80	-0.03887	0.054679	0.479295
3305_6_DI	finngen_R	outcome	3305_6_DI	Wald ratic	1	0.131915	0.607595	0.828123
3309_2_FC	finngen_R	outcome	3309_2_FC	MR Egger	158	-0.08514	0.031888	0.008385
3309_2_FC	finngen_R	outcome	3309_2_FC	Weighted	158	-0.11462	0.031755	0.000307
3309_2_FC	finngen_R	outcome	3309_2_FC	Inverse va	158	-0.09644	0.018821	2.99E-07
3309_2_FC	finngen_R	outcome	3309_2_FC	Simple mc	158	-0.08931	0.060211	0.139985
3309_2_FC	finngen_R	outcome	3309_2_FC	Weighted	158	-0.07978	0.032102	0.013998

3310_62_F	finngen_R	outcome	3310_62_F	MR Egger	164	-0.0908	0.031284	0.004217
3310_62_F	finngen_R	outcome	3310_62_F	Weighted	164	-0.08563	0.028572	0.002726
3310_62_F	finngen_R	outcome	3310_62_F	Inverse va	164	-0.09129	0.019245	2.10E-06
3310_62_F	finngen_R	outcome	3310_62_F	Simple mc	164	-0.07979	0.056967	0.16321
3310_62_F	finngen_R	outcome	3310_62_F	Weighted	164	-0.07979	0.027238	0.003882
3311_27_F	finngen_R	outcome	3311_27_F	MR Egger	45	0.155322	0.109315	0.162566
3311_27_F	finngen_R	outcome	3311_27_F	Weighted	45	0.333063	0.083785	7.03E-05
3311_27_F	finngen_R	outcome	3311_27_F	Inverse va	45	0.271596	0.064005	2.20E-05
3311_27_F	finngen_R	outcome	3311_27_F	Simple mc	45	0.415843	0.183498	0.02841
3311_27_F	finngen_R	outcome	3311_27_F	Weighted	45	0.3411	0.083882	0.000194
3313_21_F	finngen_R	outcome	3313_21_F	MR Egger	69	0.012472	0.076622	0.871191
3313_21_F	finngen_R	outcome	3313_21_F	Weighted	69	-0.00584	0.069335	0.932897
3313_21_F	finngen_R	outcome	3313_21_F	Inverse va	69	0.005986	0.046769	0.89816
3313_21_F	finngen_R	outcome	3313_21_F	Simple mc	69	0.227315	0.126953	0.077816
3313_21_F	finngen_R	outcome	3313_21_F	Weighted	69	0.042087	0.061017	0.492686
3320_49_I	finngen_R	outcome	3320_49_I	MR Egger	53	0.084416	0.092725	0.366899
3320_49_I	finngen_R	outcome	3320_49_I	Weighted	53	0.008172	0.075679	0.914012
3320_49_I	finngen_R	outcome	3320_49_I	Inverse va	53	0.031016	0.052406	0.55395
3320_49_I	finngen_R	outcome	3320_49_I	Simple mc	53	#######	0.147879	0.999589
3320_49_I	finngen_R	outcome	3320_49_I	Weighted	53	0.008231	0.074084	0.91196
3322_52_L	finngen_R	outcome	3322_52_L	MR Egger	56	-0.19774	0.082641	0.02023
3322_52_L	finngen_R	outcome	3322_52_L	Weighted	56	-0.05	0.068729	0.466881
3322_52_L	finngen_R	outcome	3322_52_L	Inverse va	56	-0.04383	0.052239	0.401453
3322_52_L	finngen_R	outcome	3322_52_L	Simple mc	56	-0.04227	0.101126	0.677574
3322_52_L	finngen_R	outcome	3322_52_L	Weighted	56	-0.09262	0.076007	0.228219
3323_37_L	finngen_R	outcome	3323_37_L	MR Egger	10	0.936967	0.630192	0.17538
3323_37_L	finngen_R	outcome	3323_37_L	Weighted	10	0.252541	0.179859	0.160287
3323_37_L	finngen_R	outcome	3323_37_L	Inverse va	10	0.218833	0.180474	0.225303
3323_37_L	finngen_R	outcome	3323_37_L	Simple mc	10	-0.0869	0.448871	0.850789
3323_37_L	finngen_R	outcome	3323_37_L	Weighted	10	0.29919	0.18947	0.148771
3324_51_L	finngen_R	outcome	3324_51_L	MR Egger	59	-0.07928	0.069385	0.257956
3324_51_L	finngen_R	outcome	3324_51_L	Weighted	59	-0.08364	0.060093	0.163947
3324_51_L	finngen_R	outcome	3324_51_L	Inverse va	59	-0.02953	0.041999	0.48198
3324_51_L	finngen_R	outcome	3324_51_L	Simple mc	59	-0.08914	0.111819	0.428595
3324_51_L	finngen_R	outcome	3324_51_L	Weighted	59	-0.08914	0.054677	0.108455
3325_2_M	finngen_R	outcome	3325_2_M	MR Egger	9	0.531927	0.510017	0.331642
3325_2_M	finngen_R	outcome	3325_2_M	Weighted	9	-0.34006	0.21467	0.113166
3325_2_M	finngen_R	outcome	3325_2_M	Inverse va	9	-0.22532	0.204103	0.269622
3325_2_M	finngen_R	outcome	3325_2_M	Simple mc	9	-0.51636	0.307759	0.131906
3325_2_M	finngen_R	outcome	3325_2_M	Weighted	9	-0.42354	0.267754	0.152346
3327_27_N	finngen_R	outcome	3327_27_N	MR Egger	21	0.090862	0.14274	0.53201
3327_27_N	finngen_R	outcome	3327_27_N	Weighted	21	0.02579	0.114722	0.822128
3327_27_N	finngen_R	outcome	3327_27_N	Inverse va	21	-0.0598	0.083462	0.473676
3327_27_N	finngen_R	outcome	3327_27_N	Simple mc	21	0.002459	0.17677	0.989038
3327_27_N	finngen_R	outcome	3327_27_N	Weighted	21	0.029271	0.115555	0.802619
3329_14_F	finngen_R	outcome	3329_14_F	MR Egger	12	-0.12153	0.309375	0.7027
3329_14_F	finngen_R	outcome	3329_14_F	Weighted	12	0.094663	0.172912	0.58406
3329_14_F	finngen_R	outcome	3329_14_F	Inverse va	12	0.063699	0.14243	0.654708
3329_14_F	finngen_R	outcome	3329_14_F	Simple mc	12	0.146953	0.256403	0.578083
3329_14_F	finngen_R	outcome	3329_14_F	Weighted	12	0.099192	0.184486	0.601513
3336_50_T	finngen_R	outcome	3336_50_T	Wald ratic	1	0.848154	0.956131	0.375042
3339_33_T	finngen_R	outcome	3339_33_T	MR Egger	94	-0.03534	0.060177	0.558421
3339_33_T	finngen_R	outcome	3339_33_T	Weighted	94	-0.04888	0.054174	0.366943
3339_33_T	finngen_R	outcome	3339_33_T	Inverse va	94	-0.06404	0.034169	0.060918
3339_33_T	finngen_R	outcome	3339_33_T	Simple mc	94	-0.08342	0.095613	0.385205
3339_33_T	finngen_R	outcome	3339_33_T	Weighted	94	-0.053	0.047044	0.262799
3340_53_T	finngen_R	outcome	3340_53_T	MR Egger	21	0.162664	0.144663	0.274835
3340_53_T	finngen_R	outcome	3340_53_T	Weighted	21	0.014472	0.115963	0.900684

3340_53_T	finngen_R	outcome	3340_53_T	Inverse va	21	0.006603	0.082603	0.936291
3340_53_T	finngen_R	outcome	3340_53_T	Simple mc	21	-0.06169	0.175248	0.728527
3340_53_T	finngen_R	outcome	3340_53_T	Weighted	21	-0.01973	0.123913	0.875058
3344_60_S	finngen_R	outcome	3344_60_S	Wald ratic	1	-0.30572	0.945022	0.746315
3348_49_E	finngen_R	outcome	3348_49_E	MR Egger	4	-1.98806	2.326598	0.482853
3348_49_E	finngen_R	outcome	3348_49_E	Weighted	4	-0.08891	0.335281	0.790866
3348_49_E	finngen_R	outcome	3348_49_E	Inverse va	4	-0.11227	0.298163	0.706511
3348_49_E	finngen_R	outcome	3348_49_E	Simple mc	4	0.035435	0.499618	0.947922
3348_49_E	finngen_R	outcome	3348_49_E	Weighted	4	-0.0747	0.461218	0.881635
3352_80_(	finngen_R	outcome	3352_80_(	MR Egger	107	-0.01498	0.056676	0.792124
3352_80_(	finngen_R	outcome	3352_80_(	Weighted	107	0.043718	0.046662	0.348796
3352_80_(	finngen_R	outcome	3352_80_(	Inverse va	107	0.04671	0.030768	0.128974
3352_80_(	finngen_R	outcome	3352_80_(	Simple mc	107	0.021508	0.086294	0.80366
3352_80_(	finngen_R	outcome	3352_80_(	Weighted	107	0.040371	0.049551	0.417053
3363_31_(	finngen_R	outcome	3363_31_(	Wald ratic	1	0.040368	0.538696	0.940265
3364_76_(	finngen_R	outcome	3364_76_(	MR Egger	23	-0.11766	0.157304	0.462787
3364_76_(	finngen_R	outcome	3364_76_(	Weighted	23	-0.09615	0.119702	0.42183
3364_76_(	finngen_R	outcome	3364_76_(	Inverse va	23	-0.07705	0.087433	0.378209
3364_76_(	finngen_R	outcome	3364_76_(	Simple mc	23	-0.0654	0.20042	0.747259
3364_76_(	finngen_R	outcome	3364_76_(	Weighted	23	-0.10158	0.121238	0.411111
3366_51_E	finngen_R	outcome	3366_51_E	MR Egger	55	-0.05505	0.069934	0.434685
3366_51_E	finngen_R	outcome	3366_51_E	Weighted	55	-0.00971	0.04401	0.825339
3366_51_E	finngen_R	outcome	3366_51_E	Inverse va	55	-0.02611	0.030594	0.393365
3366_51_E	finngen_R	outcome	3366_51_E	Simple mc	55	0.044151	0.069529	0.528113
3366_51_E	finngen_R	outcome	3366_51_E	Weighted	55	0.012094	0.045728	0.792413
3378_49_k	finngen_R	outcome	3378_49_k	MR Egger	23	-0.24029	0.112413	0.044474
3378_49_k	finngen_R	outcome	3378_49_k	Weighted	23	-0.11141	0.097499	0.253178
3378_49_k	finngen_R	outcome	3378_49_k	Inverse va	23	-0.11586	0.071242	0.103872
3378_49_k	finngen_R	outcome	3378_49_k	Simple mc	23	-0.25772	0.175614	0.156375
3378_49_k	finngen_R	outcome	3378_49_k	Weighted	23	-0.14243	0.086539	0.114018
3389_7_SE	finngen_R	outcome	3389_7_SE	MR Egger	23	0.002136	0.164807	0.989783
3389_7_SE	finngen_R	outcome	3389_7_SE	Weighted	23	-0.07654	0.117911	0.516239
3389_7_SE	finngen_R	outcome	3389_7_SE	Inverse va	23	-0.05542	0.084055	0.509658
3389_7_SE	finngen_R	outcome	3389_7_SE	Simple mc	23	-0.12861	0.191447	0.508735
3389_7_SE	finngen_R	outcome	3389_7_SE	Weighted	23	-0.085	0.128781	0.516095
3391_10_F	finngen_R	outcome	3391_10_F	Wald ratic	1	0.467386	0.708186	0.509269
3396_54_F	finngen_R	outcome	3396_54_F	MR Egger	8	0.941924	0.3535	0.037296
3396_54_F	finngen_R	outcome	3396_54_F	Weighted	8	0.605961	0.232872	0.009265
3396_54_F	finngen_R	outcome	3396_54_F	Inverse va	8	0.39146	0.192445	0.041937
3396_54_F	finngen_R	outcome	3396_54_F	Simple mc	8	-0.22772	0.394725	0.582071
3396_54_F	finngen_R	outcome	3396_54_F	Weighted	8	0.585329	0.263038	0.061398
3415_61_I	finngen_R	outcome	3415_61_I	Inverse va	2	-0.48664	0.721191	0.499819
3419_49_(	finngen_R	outcome	3419_49_(	Wald ratic	1	0.553178	0.790465	0.484044
3420_21_(	finngen_R	outcome	3420_21_(	MR Egger	22	0.006965	0.133054	0.95877
3420_21_(	finngen_R	outcome	3420_21_(	Weighted	22	0.021046	0.093007	0.820983
3420_21_(	finngen_R	outcome	3420_21_(	Inverse va	22	0.05678	0.07179	0.428988
3420_21_(	finngen_R	outcome	3420_21_(	Simple mc	22	0.106761	0.165636	0.526196
3420_21_(	finngen_R	outcome	3420_21_(	Weighted	22	0.012952	0.097174	0.895237
3421_54_T	finngen_R	outcome	3421_54_T	MR Egger	10	-0.10824	0.424687	0.80525
3421_54_T	finngen_R	outcome	3421_54_T	Weighted	10	-0.2358	0.216855	0.276878
3421_54_T	finngen_R	outcome	3421_54_T	Inverse va	10	-0.3483	0.167744	0.03786
3421_54_T	finngen_R	outcome	3421_54_T	Simple mc	10	-0.17999	0.335776	0.604931
3421_54_T	finngen_R	outcome	3421_54_T	Weighted	10	-0.20038	0.231841	0.409863
3431_54_E	finngen_R	outcome	3431_54_E	MR Egger	64	0.04008	0.041198	0.334397
3431_54_E	finngen_R	outcome	3431_54_E	Weighted	64	-0.00661	0.039628	0.867585
3431_54_E	finngen_R	outcome	3431_54_E	Inverse va	64	-0.02588	0.029075	0.373362
3431_54_E	finngen_R	outcome	3431_54_E	Simple mc	64	-0.06096	0.078365	0.439567
3431_54_E	finngen_R	outcome	3431_54_E	Weighted	64	-0.01889	0.035098	0.592367

3434_34_F	finngen_R	outcome	3434_34_F	MR Egger	8	-0.14902	0.441889	0.747427
3434_34_F	finngen_R	outcome	3434_34_F	Weighted	8	-0.26948	0.238315	0.25815
3434_34_F	finngen_R	outcome	3434_34_F	Inverse va	8	-0.4208	0.188573	0.02565
3434_34_F	finngen_R	outcome	3434_34_F	Simple mc	8	-0.19219	0.359544	0.609512
3434_34_F	finngen_R	outcome	3434_34_F	Weighted	8	-0.23632	0.249827	0.375681
3435_53_F	finngen_R	outcome	3435_53_F	MR Egger	10	0.038377	0.438365	0.93239
3435_53_F	finngen_R	outcome	3435_53_F	Weighted	10	-0.25804	0.223268	0.247785
3435_53_F	finngen_R	outcome	3435_53_F	Inverse va	10	-0.36413	0.181894	0.045296
3435_53_F	finngen_R	outcome	3435_53_F	Simple mc	10	-0.17904	0.407499	0.670755
3435_53_F	finngen_R	outcome	3435_53_F	Weighted	10	-0.22809	0.257008	0.397913
3438_10_F	finngen_R	outcome	3438_10_F	MR Egger	9	-0.18923	0.473295	0.701213
3438_10_F	finngen_R	outcome	3438_10_F	Weighted	9	0.072863	0.255981	0.775917
3438_10_F	finngen_R	outcome	3438_10_F	Inverse va	9	0.204833	0.23878	0.390986
3438_10_F	finngen_R	outcome	3438_10_F	Simple mc	9	0.143988	0.427787	0.745086
3438_10_F	finngen_R	outcome	3438_10_F	Weighted	9	0.074601	0.310243	0.816022
3440_7_Gz	finngen_R	outcome	3440_7_Gz	MR Egger	21	0.115029	0.225927	0.616516
3440_7_Gz	finngen_R	outcome	3440_7_Gz	Weighted	21	-0.25815	0.16617	0.120304
3440_7_Gz	finngen_R	outcome	3440_7_Gz	Inverse va	21	-0.22938	0.142194	0.106718
3440_7_Gz	finngen_R	outcome	3440_7_Gz	Simple mc	21	-0.47246	0.2832	0.110842
3440_7_Gz	finngen_R	outcome	3440_7_Gz	Weighted	21	-0.38298	0.258256	0.153675
3445_53_Il	finngen_R	outcome	3445_53_Il	Wald ratic	1	-0.16865	0.67319	0.802187
3448_13_Il	finngen_R	outcome	3448_13_Il	MR Egger	3	0.54476	0.930394	0.662782
3448_13_Il	finngen_R	outcome	3448_13_Il	Weighted	3	-0.25795	0.34364	0.452864
3448_13_Il	finngen_R	outcome	3448_13_Il	Inverse va	3	-0.23945	0.296222	0.418887
3448_13_Il	finngen_R	outcome	3448_13_Il	Simple mc	3	-0.39838	0.439313	0.460214
3448_13_Il	finngen_R	outcome	3448_13_Il	Weighted	3	-0.34286	0.45534	0.530034
3449_58_S	finngen_R	outcome	3449_58_S	MR Egger	94	-0.01196	0.071269	0.86713
3449_58_S	finngen_R	outcome	3449_58_S	Weighted	94	-0.10866	0.064848	0.093803
3449_58_S	finngen_R	outcome	3449_58_S	Inverse va	94	-0.06264	0.040802	0.124739
3449_58_S	finngen_R	outcome	3449_58_S	Simple mc	94	-0.10191	0.110656	0.359429
3449_58_S	finngen_R	outcome	3449_58_S	Weighted	94	-0.08132	0.061407	0.188657
3457_57_F	finngen_R	outcome	3457_57_F	MR Egger	29	0.180216	0.199145	0.373498
3457_57_F	finngen_R	outcome	3457_57_F	Weighted	29	0.120323	0.116663	0.302364
3457_57_F	finngen_R	outcome	3457_57_F	Inverse va	29	0.051978	0.086619	0.548456
3457_57_F	finngen_R	outcome	3457_57_F	Simple mc	29	0.08001	0.187242	0.672423
3457_57_F	finngen_R	outcome	3457_57_F	Weighted	29	0.098079	0.140419	0.490642
3459_49_F	finngen_R	outcome	3459_49_F	MR Egger	167	0.012068	0.033062	0.715573
3459_49_F	finngen_R	outcome	3459_49_F	Weighted	167	0.034549	0.030088	0.250864
3459_49_F	finngen_R	outcome	3459_49_F	Inverse va	167	0.025503	0.019048	0.180615
3459_49_F	finngen_R	outcome	3459_49_F	Simple mc	167	0.045144	0.053442	0.39948
3459_49_F	finngen_R	outcome	3459_49_F	Weighted	167	0.019729	0.02779	0.478749
3461_58_E	finngen_R	outcome	3461_58_E	MR Egger	12	-0.35879	0.393129	0.382898
3461_58_E	finngen_R	outcome	3461_58_E	Weighted	12	-0.17674	0.169066	0.295849
3461_58_E	finngen_R	outcome	3461_58_E	Inverse va	12	-0.16798	0.152431	0.270465
3461_58_E	finngen_R	outcome	3461_58_E	Simple mc	12	-0.36773	0.357762	0.326083
3461_58_E	finngen_R	outcome	3461_58_E	Weighted	12	-0.1162	0.181773	0.535753
3470_1_SE	finngen_R	outcome	3470_1_SE	MR Egger	5	-0.43892	0.803186	0.622762
3470_1_SE	finngen_R	outcome	3470_1_SE	Weighted	5	0.401856	0.311995	0.197739
3470_1_SE	finngen_R	outcome	3470_1_SE	Inverse va	5	0.46637	0.256931	0.0695
3470_1_SE	finngen_R	outcome	3470_1_SE	Simple mc	5	0.188844	0.454256	0.698949
3470_1_SE	finngen_R	outcome	3470_1_SE	Weighted	5	0.304241	0.410289	0.499548
3474_19_T	finngen_R	outcome	3474_19_T	Wald ratic	1	-0.11393	0.8149	0.888816
3484_60_A	finngen_R	outcome	3484_60_A	MR Egger	15	0.163138	0.247614	0.521498
3484_60_A	finngen_R	outcome	3484_60_A	Weighted	15	0.207652	0.163667	0.20453
3484_60_A	finngen_R	outcome	3484_60_A	Inverse va	15	0.098308	0.136043	0.469911
3484_60_A	finngen_R	outcome	3484_60_A	Simple mc	15	0.247158	0.285727	0.401611
3484_60_A	finngen_R	outcome	3484_60_A	Weighted	15	0.23797	0.165171	0.171644
3488_64_C	finngen_R	outcome	3488_64_C	MR Egger	10	0.314856	0.351127	0.39606

3488_64_(	finngen_R	outcome	3488_64_(	Weighted	10	0.299946	0.16297	0.065696
3488_64_(	finngen_R	outcome	3488_64_(	Inverse va	10	0.279696	0.125122	0.025392
3488_64_(	finngen_R	outcome	3488_64_(	Simple mc	10	0.195196	0.223174	0.404509
3488_64_(	finngen_R	outcome	3488_64_(	Weighted	10	0.245648	0.164666	0.169949
3495_15_(	finngen_R	outcome	3495_15_(	MR Egger	88	-0.09587	0.077269	0.218098
3495_15_(	finngen_R	outcome	3495_15_(	Weighted	88	0.003746	0.061646	0.951549
3495_15_(	finngen_R	outcome	3495_15_(	Inverse va	88	-0.02349	0.039734	0.554441
3495_15_(	finngen_R	outcome	3495_15_(	Simple mc	88	0.083002	0.11611	0.476611
3495_15_(	finngen_R	outcome	3495_15_(	Weighted	88	0.015973	0.073104	0.827554
3499_77_I	finngen_R	outcome	3499_77_I	Wald ratic	1	-0.92166	0.547696	0.092414
3508_78_(	finngen_R	outcome	3508_78_(	MR Egger	42	-0.16289	0.148355	0.27877
3508_78_(	finngen_R	outcome	3508_78_(	Weighted	42	0.023567	0.104187	0.821049
3508_78_(	finngen_R	outcome	3508_78_(	Inverse va	42	-0.0545	0.08132	0.502748
3508_78_(	finngen_R	outcome	3508_78_(	Simple mc	42	-0.18215	0.187452	0.336901
3508_78_(	finngen_R	outcome	3508_78_(	Weighted	42	-0.01454	0.128565	0.910512
3516_60_(	finngen_R	outcome	3516_60_(	MR Egger	9	0.996595	0.459668	0.066807
3516_60_(	finngen_R	outcome	3516_60_(	Weighted	9	0.530209	0.203031	0.009015
3516_60_(	finngen_R	outcome	3516_60_(	Inverse va	9	0.34286	0.158304	0.030324
3516_60_(	finngen_R	outcome	3516_60_(	Simple mc	9	0.016962	0.325031	0.959661
3516_60_(	finngen_R	outcome	3516_60_(	Weighted	9	0.602356	0.245955	0.039996
3518_54_(	finngen_R	outcome	3518_54_(	MR Egger	68	0.019419	0.084391	0.818719
3518_54_(	finngen_R	outcome	3518_54_(	Weighted	68	-0.00378	0.067496	0.955394
3518_54_(	finngen_R	outcome	3518_54_(	Inverse va	68	-0.01893	0.046032	0.680843
3518_54_(	finngen_R	outcome	3518_54_(	Simple mc	68	-0.10627	0.129733	0.415591
3518_54_(	finngen_R	outcome	3518_54_(	Weighted	68	-0.05596	0.072396	0.44223
3519_3_C(	finngen_R	outcome	3519_3_C(	MR Egger	15	-0.34663	0.193024	0.0958
3519_3_C(	finngen_R	outcome	3519_3_C(	Weighted	15	-0.26195	0.130311	0.044414
3519_3_C(	finngen_R	outcome	3519_3_C(	Inverse va	15	-0.23242	0.100871	0.021216
3519_3_C(	finngen_R	outcome	3519_3_C(	Simple mc	15	-0.22504	0.19989	0.279178
3519_3_C(	finngen_R	outcome	3519_3_C(	Weighted	15	-0.2499	0.133922	0.083131
3535_84_[	finngen_R	outcome	3535_84_[	MR Egger	33	0.080946	0.107191	0.455856
3535_84_[	finngen_R	outcome	3535_84_[	Weighted	33	0.054724	0.110751	0.621221
3535_84_[	finngen_R	outcome	3535_84_[	Inverse va	33	0.019736	0.073035	0.786985
3535_84_[	finngen_R	outcome	3535_84_[	Simple mc	33	0.126353	0.168208	0.458038
3535_84_[	finngen_R	outcome	3535_84_[	Weighted	33	0.094632	0.102137	0.361112
3554_24_/	finngen_R	outcome	3554_24_/	MR Egger	29	0.160459	0.140522	0.263532
3554_24_/	finngen_R	outcome	3554_24_/	Weighted	29	0.149333	0.112731	0.185275
3554_24_/	finngen_R	outcome	3554_24_/	Inverse va	29	0.049875	0.079568	0.530776
3554_24_/	finngen_R	outcome	3554_24_/	Simple mc	29	0.143462	0.178102	0.427317
3554_24_/	finngen_R	outcome	3554_24_/	Weighted	29	0.1368	0.121755	0.270739
3580_25_S	finngen_R	outcome	3580_25_S	MR Egger	99	-0.07311	0.058003	0.210511
3580_25_S	finngen_R	outcome	3580_25_S	Weighted	99	-0.09708	0.053952	0.071967
3580_25_S	finngen_R	outcome	3580_25_S	Inverse va	99	-0.05744	0.033376	0.085255
3580_25_S	finngen_R	outcome	3580_25_S	Simple mc	99	-0.06987	0.107601	0.517615
3580_25_S	finngen_R	outcome	3580_25_S	Weighted	99	-0.06987	0.048718	0.154688
3581_53_/	finngen_R	outcome	3581_53_/	MR Egger	63	-0.14191	0.103856	0.176838
3581_53_/	finngen_R	outcome	3581_53_/	Weighted	63	-0.03268	0.089803	0.715955
3581_53_/	finngen_R	outcome	3581_53_/	Inverse va	63	-0.06137	0.054323	0.258599
3581_53_/	finngen_R	outcome	3581_53_/	Simple mc	63	-0.06378	0.145107	0.661822
3581_53_/	finngen_R	outcome	3581_53_/	Weighted	63	-0.04496	0.083748	0.59332
3583_54_/	finngen_R	outcome	3583_54_/	Wald ratic	1	-0.38622	1.001155	0.699662
3593_72_(	finngen_R	outcome	3593_72_(	MR Egger	8	0.280932	0.479598	0.579383
3593_72_(	finngen_R	outcome	3593_72_(	Weighted	8	0.150394	0.247263	0.543031
3593_72_(	finngen_R	outcome	3593_72_(	Inverse va	8	0.136401	0.204438	0.504647
3593_72_(	finngen_R	outcome	3593_72_(	Simple mc	8	0.148745	0.333239	0.668818
3593_72_(	finngen_R	outcome	3593_72_(	Weighted	8	0.148745	0.324307	0.660383
3600_2_CI	finngen_R	outcome	3600_2_CI	MR Egger	129	0.004335	0.032253	0.893301
3600_2_CI	finngen_R	outcome	3600_2_CI	Weighted	129	0.001345	0.035552	0.969822

3600_2_Cł	finngen_R	outcome	3600_2_Cł	Inverse va	129	0.0447	0.022212	0.044171
3600_2_Cł	finngen_R	outcome	3600_2_Cł	Simple mc	129	0.047301	0.07173	0.510802
3600_2_Cł	finngen_R	outcome	3600_2_Cł	Weighted	129	0.047301	0.034702	0.175261
3605_77_N	finngen_R	outcome	3605_77_N	MR Egger	3	-0.47853	1.727685	0.827984
3605_77_N	finngen_R	outcome	3605_77_N	Weighted	3	-0.20859	0.346236	0.546881
3605_77_N	finngen_R	outcome	3605_77_N	Inverse va	3	-0.09556	0.316724	0.762876
3605_77_N	finngen_R	outcome	3605_77_N	Simple mc	3	-0.28853	0.473422	0.604236
3605_77_N	finngen_R	outcome	3605_77_N	Weighted	3	-0.25249	0.431498	0.617671
3607_71_[	finngen_R	outcome	3607_71_[	MR Egger	46	0.131466	0.117255	0.268287
3607_71_[	finngen_R	outcome	3607_71_[	Weighted	46	0.108246	0.095381	0.256426
3607_71_[	finngen_R	outcome	3607_71_[	Inverse va	46	0.119474	0.059885	0.046036
3607_71_[	finngen_R	outcome	3607_71_[	Simple mc	46	0.06434	0.158856	0.68738
3607_71_[	finngen_R	outcome	3607_71_[	Weighted	46	0.110269	0.09391	0.246493
3617_80_F	finngen_R	outcome	3617_80_F	MR Egger	149	0.061222	0.036063	0.091689
3617_80_F	finngen_R	outcome	3617_80_F	Weighted	149	0.026309	0.034034	0.439516
3617_80_F	finngen_R	outcome	3617_80_F	Inverse va	149	0.006852	0.02306	0.766355
3617_80_F	finngen_R	outcome	3617_80_F	Simple mc	149	-0.00949	0.05971	0.873939
3617_80_F	finngen_R	outcome	3617_80_F	Weighted	149	0.031885	0.030532	0.298055
3622_33_L	finngen_R	outcome	3622_33_L	MR Egger	21	-0.16765	0.181987	0.368494
3622_33_L	finngen_R	outcome	3622_33_L	Weighted	21	-0.11776	0.133494	0.37771
3622_33_L	finngen_R	outcome	3622_33_L	Inverse va	21	-0.15846	0.095996	0.098805
3622_33_L	finngen_R	outcome	3622_33_L	Simple mc	21	-0.05836	0.218344	0.791991
3622_33_L	finngen_R	outcome	3622_33_L	Weighted	21	-0.08254	0.161478	0.614828
3640_14_L	finngen_R	outcome	3640_14_L	MR Egger	14	-0.06819	0.203751	0.743659
3640_14_L	finngen_R	outcome	3640_14_L	Weighted	14	-0.15148	0.16372	0.354857
3640_14_L	finngen_R	outcome	3640_14_L	Inverse va	14	-0.15991	0.138154	0.247069
3640_14_L	finngen_R	outcome	3640_14_L	Simple mc	14	-0.36524	0.301397	0.247141
3640_14_L	finngen_R	outcome	3640_14_L	Weighted	14	-0.12176	0.162635	0.467393
3651_50_k	finngen_R	outcome	3651_50_k	MR Egger	25	0.084327	0.0993	0.404517
3651_50_k	finngen_R	outcome	3651_50_k	Weighted	25	0.039559	0.092608	0.669258
3651_50_k	finngen_R	outcome	3651_50_k	Inverse va	25	0.063631	0.068984	0.356318
3651_50_k	finngen_R	outcome	3651_50_k	Simple mc	25	-0.02238	0.160138	0.890018
3651_50_k	finngen_R	outcome	3651_50_k	Weighted	25	0.030085	0.083457	0.721637
3710_49_F	finngen_R	outcome	3710_49_F	MR Egger	47	0.074532	0.110365	0.502924
3710_49_F	finngen_R	outcome	3710_49_F	Weighted	47	-0.04079	0.087296	0.640308
3710_49_F	finngen_R	outcome	3710_49_F	Inverse va	47	-0.06448	0.057831	0.264871
3710_49_F	finngen_R	outcome	3710_49_F	Simple mc	47	0.197999	0.170874	0.252541
3710_49_F	finngen_R	outcome	3710_49_F	Weighted	47	-0.05772	0.127667	0.653306
3727_35_F	finngen_R	outcome	3727_35_F	MR Egger	3	-0.73005	1.651708	0.735053
3727_35_F	finngen_R	outcome	3727_35_F	Weighted	3	0.356606	0.480186	0.457699
3727_35_F	finngen_R	outcome	3727_35_F	Inverse va	3	0.336886	0.412733	0.414367
3727_35_F	finngen_R	outcome	3727_35_F	Simple mc	3	0.298077	0.584612	0.660836
3727_35_F	finngen_R	outcome	3727_35_F	Weighted	3	0.41728	0.566871	0.538291
3773_15_T	finngen_R	outcome	3773_15_T	MR Egger	19	0.036973	0.189335	0.847491
3773_15_T	finngen_R	outcome	3773_15_T	Weighted	19	-0.06463	0.13758	0.638546
3773_15_T	finngen_R	outcome	3773_15_T	Inverse va	19	-0.12797	0.102319	0.211063
3773_15_T	finngen_R	outcome	3773_15_T	Simple mc	19	-0.13006	0.224343	0.569275
3773_15_T	finngen_R	outcome	3773_15_T	Weighted	19	-0.04223	0.158031	0.792318
3795_6_AI	finngen_R	outcome	3795_6_AI	Wald ratic	1	-0.43781	1.005111	0.66314
3796_79_A	finngen_R	outcome	3796_79_A	Wald ratic	1	0.096569	0.326395	0.767333
3799_11_(	finngen_R	outcome	3799_11_(	MR Egger	25	0.275758	0.215274	0.212972
3799_11_(	finngen_R	outcome	3799_11_(	Weighted	25	0.123692	0.149365	0.407604
3799_11_(	finngen_R	outcome	3799_11_(	Inverse va	25	0.043298	0.108865	0.690836
3799_11_(	finngen_R	outcome	3799_11_(	Simple mc	25	-0.06459	0.248221	0.79692
3799_11_(	finngen_R	outcome	3799_11_(	Weighted	25	0.088558	0.193529	0.651359
3803_10_(	finngen_R	outcome	3803_10_(	MR Egger	103	-0.02678	0.050598	0.597747
3803_10_(	finngen_R	outcome	3803_10_(	Weighted	103	-0.04679	0.051922	0.367521
3803_10_(	finngen_R	outcome	3803_10_(	Inverse va	103	-0.06828	0.029405	0.020239

3803_10_(	finngen_R	outcome	3803_10_(	Simple mc	103	-0.11526	0.100972	0.256331
3803_10_(	finngen_R	outcome	3803_10_(	Weighted	103	-0.03797	0.045677	0.407784
3805_16_E	finngen_R	outcome	3805_16_E	MR Egger	8	-0.03463	0.525184	0.949573
3805_16_E	finngen_R	outcome	3805_16_E	Weighted	8	0.051697	0.198893	0.794922
3805_16_E	finngen_R	outcome	3805_16_E	Inverse va	8	0.055932	0.160649	0.727717
3805_16_E	finngen_R	outcome	3805_16_E	Simple mc	8	0.181261	0.267682	0.520054
3805_16_E	finngen_R	outcome	3805_16_E	Weighted	8	0.079938	0.215577	0.721745
3806_55_E	finngen_R	outcome	3806_55_E	MR Egger	5	-0.48996	0.844805	0.602621
3806_55_E	finngen_R	outcome	3806_55_E	Weighted	5	0.127889	0.269214	0.634753
3806_55_E	finngen_R	outcome	3806_55_E	Inverse va	5	0.179612	0.294386	0.54178
3806_55_E	finngen_R	outcome	3806_55_E	Simple mc	5	0.219345	0.37221	0.587332
3806_55_E	finngen_R	outcome	3806_55_E	Weighted	5	0.179489	0.343705	0.629105
3807_1_FC	finngen_R	outcome	3807_1_FC	Wald ratic	1	1.115853	0.698692	0.110252
3810_50_F	finngen_R	outcome	3810_50_F	MR Egger	8	-0.58853	0.371535	0.164272
3810_50_F	finngen_R	outcome	3810_50_F	Weighted	8	-0.12995	0.23773	0.584625
3810_50_F	finngen_R	outcome	3810_50_F	Inverse va	8	0.06683	0.191931	0.72769
3810_50_F	finngen_R	outcome	3810_50_F	Simple mc	8	-0.15607	0.414752	0.717842
3810_50_F	finngen_R	outcome	3810_50_F	Weighted	8	-0.16728	0.243585	0.514341
3820_68_N	finngen_R	outcome	3820_68_N	MR Egger	9	0.439659	0.457529	0.368591
3820_68_N	finngen_R	outcome	3820_68_N	Weighted	9	-0.23606	0.187999	0.209253
3820_68_N	finngen_R	outcome	3820_68_N	Inverse va	9	-0.22797	0.158549	0.150472
3820_68_N	finngen_R	outcome	3820_68_N	Simple mc	9	-0.53134	0.344585	0.16165
3820_68_N	finngen_R	outcome	3820_68_N	Weighted	9	-0.1426	0.188187	0.47033
3825_18_N	finngen_R	outcome	3825_18_N	Wald ratic	1	-0.52918	0.634767	0.404469
3835_11_T	finngen_R	outcome	3835_11_T	Wald ratic	1	-0.57094	0.507872	0.260934
3848_14_(	finngen_R	outcome	3848_14_(	Wald ratic	1	0.159654	0.704263	0.820659
3858_5_A(	finngen_R	outcome	3858_5_A(	MR Egger	98	0.018018	0.041516	0.665258
3858_5_A(	finngen_R	outcome	3858_5_A(	Weighted	98	0.025806	0.03626	0.476656
3858_5_A(	finngen_R	outcome	3858_5_A(	Inverse va	98	0.031429	0.025625	0.220018
3858_5_A(	finngen_R	outcome	3858_5_A(	Simple mc	98	0.092558	0.059019	0.120073
3858_5_A(	finngen_R	outcome	3858_5_A(	Weighted	98	0.03998	0.033607	0.237083
3890_8_LD	finngen_R	outcome	3890_8_LD	MR Egger	4	0.531285	0.890405	0.611268
3890_8_LD	finngen_R	outcome	3890_8_LD	Weighted	4	0.316054	0.373299	0.39719
3890_8_LD	finngen_R	outcome	3890_8_LD	Inverse va	4	0.424116	0.299758	0.157108
3890_8_LD	finngen_R	outcome	3890_8_LD	Simple mc	4	0.170725	0.587184	0.790182
3890_8_LD	finngen_R	outcome	3890_8_LD	Weighted	4	0.090602	0.573214	0.884449
3905_62_L	finngen_R	outcome	3905_62_L	Wald ratic	1	0.409776	0.715929	0.567072
4126_22_E	finngen_R	outcome	4126_22_E	MR Egger	88	-0.06129	0.05704	0.285633
4126_22_E	finngen_R	outcome	4126_22_E	Weighted	88	-0.01311	0.053946	0.808032
4126_22_E	finngen_R	outcome	4126_22_E	Inverse va	88	-0.03577	0.035296	0.310883
4126_22_E	finngen_R	outcome	4126_22_E	Simple mc	88	0.069542	0.098202	0.480748
4126_22_E	finngen_R	outcome	4126_22_E	Weighted	88	-0.05003	0.054584	0.361884
4127_75_(	finngen_R	outcome	4127_75_(	MR Egger	16	0.097566	0.309535	0.757258
4127_75_(	finngen_R	outcome	4127_75_(	Weighted	16	0.146933	0.204881	0.473275
4127_75_(	finngen_R	outcome	4127_75_(	Inverse va	16	0.055764	0.151235	0.712336
4127_75_(	finngen_R	outcome	4127_75_(	Simple mc	16	0.462208	0.416288	0.284348
4127_75_(	finngen_R	outcome	4127_75_(	Weighted	16	0.30381	0.336704	0.381155
4131_72_F	finngen_R	outcome	4131_72_F	MR Egger	11	-0.14273	0.364223	0.704262
4131_72_F	finngen_R	outcome	4131_72_F	Weighted	11	-0.25759	0.207717	0.214936
4131_72_F	finngen_R	outcome	4131_72_F	Inverse va	11	-0.43049	0.167676	0.010247
4131_72_F	finngen_R	outcome	4131_72_F	Simple mc	11	-0.97028	0.384118	0.030077
4131_72_F	finngen_R	outcome	4131_72_F	Weighted	11	-0.22579	0.226456	0.342259
4133_54_(	finngen_R	outcome	4133_54_(	Wald ratic	1	0.017869	0.452659	0.968511
4141_79_(	finngen_R	outcome	4141_79_(	MR Egger	3	1.470869	1.853939	0.573027
4141_79_(	finngen_R	outcome	4141_79_(	Weighted	3	0.08573	0.39056	0.826256
4141_79_(	finngen_R	outcome	4141_79_(	Inverse va	3	0.218219	0.421404	0.604571
4141_79_(	finngen_R	outcome	4141_79_(	Simple mc	3	0.006833	0.529281	0.990872
4141_79_(	finngen_R	outcome	4141_79_(	Weighted	3	0.017428	0.439376	0.971963

4145_58_N	finngen_R	outcome	4145_58_N	MR Egger	9	-0.32842	0.599409	0.600783
4145_58_N	finngen_R	outcome	4145_58_N	Weighted	9	-0.16724	0.290854	0.565298
4145_58_N	finngen_R	outcome	4145_58_N	Inverse va	9	-0.24289	0.234391	0.300085
4145_58_N	finngen_R	outcome	4145_58_N	Simple mc	9	-0.11495	0.453586	0.806333
4145_58_N	finngen_R	outcome	4145_58_N	Weighted	9	-0.12362	0.339436	0.725144
4151_6_PL	finngen_R	outcome	4151_6_PL	MR Egger	37	-0.1143	0.121301	0.352486
4151_6_PL	finngen_R	outcome	4151_6_PL	Weighted	37	-0.18	0.102381	0.078726
4151_6_PL	finngen_R	outcome	4151_6_PL	Inverse va	37	-0.11642	0.068565	0.08952
4151_6_PL	finngen_R	outcome	4151_6_PL	Simple mc	37	-0.2094	0.176576	0.24343
4151_6_PL	finngen_R	outcome	4151_6_PL	Weighted	37	-0.16496	0.117471	0.168821
4152_58_K	finngen_R	outcome	4152_58_K	MR Egger	48	0.04607	0.097787	0.639776
4152_58_K	finngen_R	outcome	4152_58_K	Weighted	48	-0.007	0.083595	0.933296
4152_58_K	finngen_R	outcome	4152_58_K	Inverse va	48	0.014577	0.060109	0.80839
4152_58_K	finngen_R	outcome	4152_58_K	Simple mc	48	0.066068	0.159214	0.680055
4152_58_K	finngen_R	outcome	4152_58_K	Weighted	48	-0.01282	0.074816	0.864684
4153_11_S	finngen_R	outcome	4153_11_S	MR Egger	41	-0.13447	0.147112	0.366304
4153_11_S	finngen_R	outcome	4153_11_S	Weighted	41	-0.0391	0.121075	0.746766
4153_11_S	finngen_R	outcome	4153_11_S	Inverse va	41	-0.03061	0.080037	0.702139
4153_11_S	finngen_R	outcome	4153_11_S	Simple mc	41	0.1148	0.209548	0.586844
4153_11_S	finngen_R	outcome	4153_11_S	Weighted	41	-0.01753	0.127226	0.891091
4154_57_S	finngen_R	outcome	4154_57_S	MR Egger	25	0.042745	0.136913	0.757696
4154_57_S	finngen_R	outcome	4154_57_S	Weighted	25	0.167167	0.108404	0.123057
4154_57_S	finngen_R	outcome	4154_57_S	Inverse va	25	0.11427	0.081991	0.163413
4154_57_S	finngen_R	outcome	4154_57_S	Simple mc	25	0.050357	0.200612	0.803936
4154_57_S	finngen_R	outcome	4154_57_S	Weighted	25	0.101638	0.106501	0.349427
4155_3_TN	finngen_R	outcome	4155_3_TN	MR Egger	134	0.045005	0.039983	0.262377
4155_3_TN	finngen_R	outcome	4155_3_TN	Weighted	134	0.008201	0.039794	0.836726
4155_3_TN	finngen_R	outcome	4155_3_TN	Inverse va	134	0.038021	0.024531	0.121158
4155_3_TN	finngen_R	outcome	4155_3_TN	Simple mc	134	0.009093	0.075634	0.904489
4155_3_TN	finngen_R	outcome	4155_3_TN	Weighted	134	0.009093	0.035443	0.79792
4157_2_F2	finngen_R	outcome	4157_2_F2	MR Egger	6	0.108291	1.652076	0.950883
4157_2_F2	finngen_R	outcome	4157_2_F2	Weighted	6	-0.71539	0.261706	0.006265
4157_2_F2	finngen_R	outcome	4157_2_F2	Inverse va	6	-0.65983	0.3146	0.035961
4157_2_F2	finngen_R	outcome	4157_2_F2	Simple mc	6	-0.47693	0.413017	0.300385
4157_2_F2	finngen_R	outcome	4157_2_F2	Weighted	6	-0.59982	0.242441	0.056248
4158_54_F	finngen_R	outcome	4158_54_F	MR Egger	25	-0.19788	0.155072	0.214673
4158_54_F	finngen_R	outcome	4158_54_F	Weighted	25	-0.25485	0.100998	0.011624
4158_54_F	finngen_R	outcome	4158_54_F	Inverse va	25	-0.20564	0.071094	0.003822
4158_54_F	finngen_R	outcome	4158_54_F	Simple mc	25	-0.20632	0.142489	0.160571
4158_54_F	finngen_R	outcome	4158_54_F	Weighted	25	-0.25427	0.105894	0.02445
4159_130_	finngen_R	outcome	4159_130_	MR Egger	19	0.020049	0.229951	0.93154
4159_130_	finngen_R	outcome	4159_130_	Weighted	19	-0.07027	0.132394	0.595587
4159_130_	finngen_R	outcome	4159_130_	Inverse va	19	-0.05035	0.101672	0.620465
4159_130_	finngen_R	outcome	4159_130_	Simple mc	19	-0.03948	0.217812	0.858185
4159_130_	finngen_R	outcome	4159_130_	Weighted	19	-0.0528	0.135964	0.702337
4160_49_N	finngen_R	outcome	4160_49_N	MR Egger	4	-0.72904	1.460881	0.667235
4160_49_N	finngen_R	outcome	4160_49_N	Weighted	4	0.469281	0.333487	0.15937
4160_49_N	finngen_R	outcome	4160_49_N	Inverse va	4	0.486083	0.280031	0.082596
4160_49_N	finngen_R	outcome	4160_49_N	Simple mc	4	0.861403	0.463292	0.159939
4160_49_N	finngen_R	outcome	4160_49_N	Weighted	4	0.323966	0.346967	0.419332
4162_54_T	finngen_R	outcome	4162_54_T	MR Egger	9	-0.3822	0.294189	0.235035
4162_54_T	finngen_R	outcome	4162_54_T	Weighted	9	-0.00378	0.212781	0.985842
4162_54_T	finngen_R	outcome	4162_54_T	Inverse va	9	-0.06131	0.151047	0.684794
4162_54_T	finngen_R	outcome	4162_54_T	Simple mc	9	0.171445	0.339082	0.626762
4162_54_T	finngen_R	outcome	4162_54_T	Weighted	9	0.15804	0.246344	0.539114
4187_49_F	finngen_R	outcome	4187_49_F	Wald ratic	1	0.523475	0.370234	0.15739
4192_10_A	finngen_R	outcome	4192_10_A	MR Egger	41	-0.23389	0.081403	0.006543
4192_10_A	finngen_R	outcome	4192_10_A	Weighted	41	-0.19174	0.070763	0.006737

4192_10_/	finngen_R	outcome	4192_10_/	Inverse va	41	-0.0649	0.051769	0.209936
4192_10_/	finngen_R	outcome	4192_10_/	Simple mc	41	0.056365	0.119875	0.64077
4192_10_/	finngen_R	outcome	4192_10_/	Weighted	41	-0.20366	0.065843	0.003604
4220_39_F	finngen_R	outcome	4220_39_F	Inverse va	2	0.443169	0.416399	0.287198
4232_19_I	finngen_R	outcome	4232_19_I	MR Egger	11	0.306621	0.793247	0.708077
4232_19_I	finngen_R	outcome	4232_19_I	Weighted	11	-0.28287	0.270438	0.29558
4232_19_I	finngen_R	outcome	4232_19_I	Inverse va	11	-0.27382	0.207693	0.187372
4232_19_I	finngen_R	outcome	4232_19_I	Simple mc	11	-0.39547	0.452607	0.402741
4232_19_I	finngen_R	outcome	4232_19_I	Weighted	11	-0.31956	0.385197	0.426119
4234_8_IL	finngen_R	outcome	4234_8_IL	MR Egger	90	-0.08282	0.05271	0.119732
4234_8_IL	finngen_R	outcome	4234_8_IL	Weighted	90	-0.06671	0.044408	0.133037
4234_8_IL	finngen_R	outcome	4234_8_IL	Inverse va	90	-0.0367	0.029461	0.212836
4234_8_IL	finngen_R	outcome	4234_8_IL	Simple mc	90	-0.11598	0.073075	0.116025
4234_8_IL	finngen_R	outcome	4234_8_IL	Weighted	90	-0.06534	0.040721	0.11213
4278_14_F	finngen_R	outcome	4278_14_F	MR Egger	4	1.376144	0.754968	0.209912
4278_14_F	finngen_R	outcome	4278_14_F	Weighted	4	0.247992	0.24484	0.31112
4278_14_F	finngen_R	outcome	4278_14_F	Inverse va	4	0.234522	0.21887	0.283941
4278_14_F	finngen_R	outcome	4278_14_F	Simple mc	4	-0.26214	0.489593	0.629505
4278_14_F	finngen_R	outcome	4278_14_F	Weighted	4	0.534367	0.287602	0.160146
4294_16_S	finngen_R	outcome	4294_16_S	MR Egger	3	-0.52167	0.422157	0.433123
4294_16_S	finngen_R	outcome	4294_16_S	Weighted	3	-0.0096	0.281743	0.972824
4294_16_S	finngen_R	outcome	4294_16_S	Inverse va	3	0.110011	0.370916	0.766778
4294_16_S	finngen_R	outcome	4294_16_S	Simple mc	3	0.124468	0.560335	0.844831
4294_16_S	finngen_R	outcome	4294_16_S	Weighted	3	-0.12876	0.283352	0.694085
4324_33_C	finngen_R	outcome	4324_33_C	MR Egger	86	0.024852	0.08551	0.772053
4324_33_C	finngen_R	outcome	4324_33_C	Weighted	86	-0.02499	0.066351	0.706478
4324_33_C	finngen_R	outcome	4324_33_C	Inverse va	86	0.033097	0.045693	0.468862
4324_33_C	finngen_R	outcome	4324_33_C	Simple mc	86	0.018593	0.113695	0.870483
4324_33_C	finngen_R	outcome	4324_33_C	Weighted	86	-0.00984	0.069847	0.888317
4328_2_B(	finngen_R	outcome	4328_2_B(	MR Egger	22	-0.09095	0.159141	0.574008
4328_2_B(	finngen_R	outcome	4328_2_B(	Weighted	22	0.00224	0.124686	0.985668
4328_2_B(	finngen_R	outcome	4328_2_B(	Inverse va	22	0.095697	0.091356	0.294863
4328_2_B(	finngen_R	outcome	4328_2_B(	Simple mc	22	-0.05754	0.236257	0.80995
4328_2_B(	finngen_R	outcome	4328_2_B(	Weighted	22	-0.01264	0.126195	0.921192
4337_49_C	finngen_R	outcome	4337_49_C	MR Egger	11	0.38895	0.265414	0.176842
4337_49_C	finngen_R	outcome	4337_49_C	Weighted	11	0.378389	0.168072	0.024364
4337_49_C	finngen_R	outcome	4337_49_C	Inverse va	11	0.330711	0.119965	0.005838
4337_49_C	finngen_R	outcome	4337_49_C	Simple mc	11	0.335469	0.257656	0.222105
4337_49_C	finngen_R	outcome	4337_49_C	Weighted	11	0.335469	0.190791	0.109202
4342_10_I	finngen_R	outcome	4342_10_I	MR Egger	136	-0.02195	0.0288	0.447373
4342_10_I	finngen_R	outcome	4342_10_I	Weighted	136	-0.03151	0.029897	0.291873
4342_10_I	finngen_R	outcome	4342_10_I	Inverse va	136	-0.03188	0.018181	0.079505
4342_10_I	finngen_R	outcome	4342_10_I	Simple mc	136	-0.04015	0.058178	0.491355
4342_10_I	finngen_R	outcome	4342_10_I	Weighted	136	-0.04015	0.025662	0.120071
4355_13_D	finngen_R	outcome	4355_13_D	MR Egger	22	0.241114	0.171286	0.174583
4355_13_D	finngen_R	outcome	4355_13_D	Weighted	22	0.092197	0.122727	0.452511
4355_13_D	finngen_R	outcome	4355_13_D	Inverse va	22	-0.00457	0.086014	0.957661
4355_13_D	finngen_R	outcome	4355_13_D	Simple mc	22	0.118922	0.242505	0.628943
4355_13_D	finngen_R	outcome	4355_13_D	Weighted	22	0.118922	0.126557	0.358069
4374_45_C	finngen_R	outcome	4374_45_C	MR Egger	64	0.053148	0.073052	0.469634
4374_45_C	finngen_R	outcome	4374_45_C	Weighted	64	-0.10263	0.067435	0.128039
4374_45_C	finngen_R	outcome	4374_45_C	Inverse va	64	-0.05371	0.042738	0.208821
4374_45_C	finngen_R	outcome	4374_45_C	Simple mc	64	-0.15266	0.132459	0.253476
4374_45_C	finngen_R	outcome	4374_45_C	Weighted	64	-0.07914	0.069639	0.260078
4407_10_M	finngen_R	outcome	4407_10_M	MR Egger	52	-0.06962	0.055607	0.216386
4407_10_M	finngen_R	outcome	4407_10_M	Weighted	52	-0.01763	0.032848	0.591452
4407_10_M	finngen_R	outcome	4407_10_M	Inverse va	52	-0.01737	0.022137	0.43272
4407_10_M	finngen_R	outcome	4407_10_M	Simple mc	52	-0.04836	0.048954	0.327928

4407_10_N	finngen_R	outcome	4407_10_N	Weighted	52	-0.02702	0.037569	0.4753
4413_3_SL	finngen_R	outcome	4413_3_SL	MR Egger	6	-0.55947	1.448617	0.719006
4413_3_SL	finngen_R	outcome	4413_3_SL	Weighted	6	0.193891	0.334309	0.561932
4413_3_SL	finngen_R	outcome	4413_3_SL	Inverse va	6	-0.04964	0.333288	0.881603
4413_3_SL	finngen_R	outcome	4413_3_SL	Simple mc	6	0.261674	0.533672	0.64466
4413_3_SL	finngen_R	outcome	4413_3_SL	Weighted	6	0.347254	0.607569	0.592362
4435_66_E	finngen_R	outcome	4435_66_E	MR Egger	136	-0.07252	0.0331	0.030194
4435_66_E	finngen_R	outcome	4435_66_E	Weighted	136	-0.03744	0.035473	0.291243
4435_66_E	finngen_R	outcome	4435_66_E	Inverse va	136	-0.05754	0.023333	0.013668
4435_66_E	finngen_R	outcome	4435_66_E	Simple mc	136	-0.07393	0.076306	0.334339
4435_66_E	finngen_R	outcome	4435_66_E	Weighted	136	-0.05372	0.030878	0.084179
4440_15_F	finngen_R	outcome	4440_15_F	MR Egger	108	0.029505	0.086875	0.73481
4440_15_F	finngen_R	outcome	4440_15_F	Weighted	108	-0.10763	0.062553	0.085331
4440_15_F	finngen_R	outcome	4440_15_F	Inverse va	108	-0.11858	0.040351	0.003295
4440_15_F	finngen_R	outcome	4440_15_F	Simple mc	108	-0.36477	0.130465	0.006137
4440_15_F	finngen_R	outcome	4440_15_F	Weighted	108	-0.10935	0.080161	0.175387
4455_89_N	finngen_R	outcome	4455_89_N	MR Egger	26	-0.17689	0.230529	0.450382
4455_89_N	finngen_R	outcome	4455_89_N	Weighted	26	0.046805	0.14008	0.738282
4455_89_N	finngen_R	outcome	4455_89_N	Inverse va	26	0.020086	0.111277	0.856759
4455_89_N	finngen_R	outcome	4455_89_N	Simple mc	26	0.028336	0.25001	0.910667
4455_89_N	finngen_R	outcome	4455_89_N	Weighted	26	0.015781	0.136757	0.909052
4459_68_F	finngen_R	outcome	4459_68_F	MR Egger	166	0.049956	0.044524	0.263499
4459_68_F	finngen_R	outcome	4459_68_F	Weighted	166	0.020633	0.044094	0.639834
4459_68_F	finngen_R	outcome	4459_68_F	Inverse va	166	-0.00335	0.027847	0.904179
4459_68_F	finngen_R	outcome	4459_68_F	Simple mc	166	-0.13924	0.090517	0.125884
4459_68_F	finngen_R	outcome	4459_68_F	Weighted	166	0.002931	0.04342	0.946259
4464_10_S	finngen_R	outcome	4464_10_S	MR Egger	3	-0.4343	1.451548	0.81492
4464_10_S	finngen_R	outcome	4464_10_S	Weighted	3	0.820056	0.576145	0.154635
4464_10_S	finngen_R	outcome	4464_10_S	Inverse va	3	0.954041	0.445972	0.032416
4464_10_S	finngen_R	outcome	4464_10_S	Simple mc	3	0.682939	0.652744	0.405252
4464_10_S	finngen_R	outcome	4464_10_S	Weighted	3	0.69772	0.658217	0.400233
4467_49_S	finngen_R	outcome	4467_49_S	MR Egger	82	-0.07046	0.061593	0.256039
4467_49_S	finngen_R	outcome	4467_49_S	Weighted	82	-0.11019	0.053266	0.038578
4467_49_S	finngen_R	outcome	4467_49_S	Inverse va	82	-0.05435	0.033498	0.104731
4467_49_S	finngen_R	outcome	4467_49_S	Simple mc	82	-0.12272	0.097331	0.210993
4467_49_S	finngen_R	outcome	4467_49_S	Weighted	82	-0.12272	0.053578	0.024595
4471_50_T	finngen_R	outcome	4471_50_T	MR Egger	35	0.113637	0.120309	0.351755
4471_50_T	finngen_R	outcome	4471_50_T	Weighted	35	0.037096	0.098457	0.706345
4471_50_T	finngen_R	outcome	4471_50_T	Inverse va	35	-0.0168	0.064755	0.795315
4471_50_T	finngen_R	outcome	4471_50_T	Simple mc	35	0.094317	0.169945	0.582539
4471_50_T	finngen_R	outcome	4471_50_T	Weighted	35	0.073453	0.090003	0.420114
4479_14_S	finngen_R	outcome	4479_14_S	MR Egger	64	0.007383	0.068607	0.914644
4479_14_S	finngen_R	outcome	4479_14_S	Weighted	64	0.010413	0.052435	0.84259
4479_14_S	finngen_R	outcome	4479_14_S	Inverse va	64	0.020304	0.035423	0.566511
4479_14_S	finngen_R	outcome	4479_14_S	Simple mc	64	-0.0279	0.081583	0.733536
4479_14_S	finngen_R	outcome	4479_14_S	Weighted	64	0.011451	0.05011	0.819991
4480_59_C	finngen_R	outcome	4480_59_C	Wald ratic	1	-0.61512	0.435393	0.157715
4498_62_N	finngen_R	outcome	4498_62_N	MR Egger	40	0.11081	0.1278	0.391352
4498_62_N	finngen_R	outcome	4498_62_N	Weighted	40	0.059237	0.096492	0.539277
4498_62_N	finngen_R	outcome	4498_62_N	Inverse va	40	0.030987	0.065039	0.633764
4498_62_N	finngen_R	outcome	4498_62_N	Simple mc	40	-0.08272	0.165597	0.620213
4498_62_N	finngen_R	outcome	4498_62_N	Weighted	40	0.070921	0.099314	0.479413
4499_21_F	finngen_R	outcome	4499_21_F	MR Egger	3	-1.715	1.155541	0.377462
4499_21_F	finngen_R	outcome	4499_21_F	Weighted	3	-0.37936	0.295251	0.19884
4499_21_F	finngen_R	outcome	4499_21_F	Inverse va	3	-0.3179	0.352413	0.367016
4499_21_F	finngen_R	outcome	4499_21_F	Simple mc	3	-0.13253	0.657685	0.85894
4499_21_F	finngen_R	outcome	4499_21_F	Weighted	3	-0.41018	0.275272	0.274671
4500_50_C	finngen_R	outcome	4500_50_C	MR Egger	20	0.428006	0.374433	0.267982

4500_50_(	finngen_R	outcome	4500_50_(	Weighted	20	0.088868	0.200822	0.658113
4500_50_(	finngen_R	outcome	4500_50_(	Inverse va	20	0.271688	0.17585	0.122346
4500_50_(	finngen_R	outcome	4500_50_(	Simple mc	20	0.302739	0.352495	0.401131
4500_50_(	finngen_R	outcome	4500_50_(	Weighted	20	0.052767	0.229311	0.820465
4534_10_F	finngen_R	outcome	4534_10_F	MR Egger	19	0.140929	0.209028	0.509241
4534_10_F	finngen_R	outcome	4534_10_F	Weighted	19	-0.00512	0.143685	0.971579
4534_10_F	finngen_R	outcome	4534_10_F	Inverse va	19	-0.07358	0.104295	0.480518
4534_10_F	finngen_R	outcome	4534_10_F	Simple mc	19	0.048641	0.237116	0.839767
4534_10_F	finngen_R	outcome	4534_10_F	Weighted	19	0.017823	0.146822	0.904728
4535_50_E	finngen_R	outcome	4535_50_E	MR Egger	169	-0.02372	0.032034	0.459964
4535_50_E	finngen_R	outcome	4535_50_E	Weighted	169	-0.01435	0.030125	0.633863
4535_50_E	finngen_R	outcome	4535_50_E	Inverse va	169	-0.0215	0.020255	0.288385
4535_50_E	finngen_R	outcome	4535_50_E	Simple mc	169	-0.00735	0.05935	0.901586
4535_50_E	finngen_R	outcome	4535_50_E	Weighted	169	-0.03491	0.028395	0.220683
4559_64_k	finngen_R	outcome	4559_64_k	MR Egger	44	0.156551	0.13127	0.239725
4559_64_k	finngen_R	outcome	4559_64_k	Weighted	44	0.050444	0.089463	0.572853
4559_64_k	finngen_R	outcome	4559_64_k	Inverse va	44	-0.02833	0.075817	0.708696
4559_64_k	finngen_R	outcome	4559_64_k	Simple mc	44	0.170946	0.17695	0.339411
4559_64_k	finngen_R	outcome	4559_64_k	Weighted	44	0.088168	0.088665	0.325591
4564_2_PL	finngen_R	outcome	4564_2_PL	MR Egger	119	0.032942	0.036387	0.367159
4564_2_PL	finngen_R	outcome	4564_2_PL	Weighted	119	0.083143	0.040253	0.038876
4564_2_PL	finngen_R	outcome	4564_2_PL	Inverse va	119	0.081535	0.02583	0.001596
4564_2_PL	finngen_R	outcome	4564_2_PL	Simple mc	119	0.110504	0.087787	0.210595
4564_2_PL	finngen_R	outcome	4564_2_PL	Weighted	119	0.038914	0.03431	0.259018
4721_54_T	finngen_R	outcome	4721_54_T	MR Egger	7	0.315262	0.532785	0.579775
4721_54_T	finngen_R	outcome	4721_54_T	Weighted	7	-0.03805	0.269515	0.887732
4721_54_T	finngen_R	outcome	4721_54_T	Inverse va	7	-0.06684	0.225266	0.766679
4721_54_T	finngen_R	outcome	4721_54_T	Simple mc	7	-0.08404	0.364745	0.825428
4721_54_T	finngen_R	outcome	4721_54_T	Weighted	7	0.063698	0.342064	0.858412
4763_31_A	finngen_R	outcome	4763_31_A	MR Egger	6	0.071356	0.219588	0.761501
4763_31_A	finngen_R	outcome	4763_31_A	Weighted	6	-0.00703	0.171452	0.967305
4763_31_A	finngen_R	outcome	4763_31_A	Inverse va	6	-0.03473	0.14941	0.816201
4763_31_A	finngen_R	outcome	4763_31_A	Simple mc	6	-0.01354	0.25653	0.959946
4763_31_A	finngen_R	outcome	4763_31_A	Weighted	6	-0.00875	0.18314	0.963738
4771_10_S	finngen_R	outcome	4771_10_S	MR Egger	10	-0.30215	0.203053	0.175057
4771_10_S	finngen_R	outcome	4771_10_S	Weighted	10	-0.19203	0.129613	0.138447
4771_10_S	finngen_R	outcome	4771_10_S	Inverse va	10	-0.14093	0.110633	0.202713
4771_10_S	finngen_R	outcome	4771_10_S	Simple mc	10	-0.16719	0.200822	0.426637
4771_10_S	finngen_R	outcome	4771_10_S	Weighted	10	-0.16719	0.122954	0.206977
4775_34_(	finngen_R	outcome	4775_34_(	MR Egger	17	0.018674	0.190722	0.923296
4775_34_(	finngen_R	outcome	4775_34_(	Weighted	17	-0.05955	0.142891	0.676837
4775_34_(	finngen_R	outcome	4775_34_(	Inverse va	17	0.063643	0.112036	0.569994
4775_34_(	finngen_R	outcome	4775_34_(	Simple mc	17	0.181171	0.244945	0.470233
4775_34_(	finngen_R	outcome	4775_34_(	Weighted	17	-0.06314	0.159392	0.697256
4801_13_L	finngen_R	outcome	4801_13_L	MR Egger	17	0.213246	0.19358	0.288002
4801_13_L	finngen_R	outcome	4801_13_L	Weighted	17	0.192833	0.108955	0.076756
4801_13_L	finngen_R	outcome	4801_13_L	Inverse va	17	0.092982	0.092605	0.315343
4801_13_L	finngen_R	outcome	4801_13_L	Simple mc	17	0.266424	0.198331	0.197909
4801_13_L	finngen_R	outcome	4801_13_L	Weighted	17	0.183898	0.09971	0.083733
4811_33_I	finngen_R	outcome	4811_33_I	MR Egger	8	0.500457	0.756412	0.532786
4811_33_I	finngen_R	outcome	4811_33_I	Weighted	8	-0.10671	0.255338	0.676002
4811_33_I	finngen_R	outcome	4811_33_I	Inverse va	8	0.028438	0.190404	0.881274
4811_33_I	finngen_R	outcome	4811_33_I	Simple mc	8	-0.2055	0.390917	0.615338
4811_33_I	finngen_R	outcome	4811_33_I	Weighted	8	-0.26504	0.352621	0.476774
4831_4_SE	finngen_R	outcome	4831_4_SE	MR Egger	58	0.066807	0.104896	0.526795
4831_4_SE	finngen_R	outcome	4831_4_SE	Weighted	58	0.056386	0.083692	0.500483
4831_4_SE	finngen_R	outcome	4831_4_SE	Inverse va	58	0.090885	0.054277	0.094036
4831_4_SE	finngen_R	outcome	4831_4_SE	Simple mc	58	0.051854	0.152258	0.734681

4831_4_SE	finngen_R	outcome	4831_4_SE	Weighted	58	0.074396	0.077104	0.338681
4834_61_E	finngen_R	outcome	4834_61_E	MR Egger	26	-0.0399	0.205004	0.847312
4834_61_E	finngen_R	outcome	4834_61_E	Weighted	26	-0.0152	0.14267	0.915153
4834_61_E	finngen_R	outcome	4834_61_E	Inverse va	26	0.005776	0.099414	0.953671
4834_61_E	finngen_R	outcome	4834_61_E	Simple mc	26	0.100576	0.266215	0.708767
4834_61_E	finngen_R	outcome	4834_61_E	Weighted	26	-0.06163	0.225606	0.786954
4866_59_N	finngen_R	outcome	4866_59_N	Wald ratic	1	0.476878	0.588786	0.417978
4867_15_\	finngen_R	outcome	4867_15_\	MR Egger	74	-0.27048	0.121179	0.028721
4867_15_\	finngen_R	outcome	4867_15_\	Weighted	74	-0.25989	0.084699	0.002152
4867_15_\	finngen_R	outcome	4867_15_\	Inverse va	74	-0.17221	0.065774	0.008839
4867_15_\	finngen_R	outcome	4867_15_\	Simple mc	74	-0.22053	0.172674	0.205596
4867_15_\	finngen_R	outcome	4867_15_\	Weighted	74	-0.31737	0.094304	0.001221
4874_3_AI	finngen_R	outcome	4874_3_AI	MR Egger	107	-0.03147	0.058325	0.590644
4874_3_AI	finngen_R	outcome	4874_3_AI	Weighted	107	-0.14081	0.054531	0.009814
4874_3_AI	finngen_R	outcome	4874_3_AI	Inverse va	107	-0.08538	0.035969	0.017612
4874_3_AI	finngen_R	outcome	4874_3_AI	Simple mc	107	-0.15752	0.104277	0.133867
4874_3_AI	finngen_R	outcome	4874_3_AI	Weighted	107	-0.14717	0.054925	0.008551
4878_3_F1	finngen_R	outcome	4878_3_F1	MR Egger	12	0.404633	0.284218	0.184986
4878_3_F1	finngen_R	outcome	4878_3_F1	Weighted	12	0.122884	0.187819	0.51294
4878_3_F1	finngen_R	outcome	4878_3_F1	Inverse va	12	0.083988	0.14993	0.575357
4878_3_F1	finngen_R	outcome	4878_3_F1	Simple mc	12	0.081753	0.291404	0.784263
4878_3_F1	finngen_R	outcome	4878_3_F1	Weighted	12	0.143852	0.210589	0.508681
4886_3_C(	finngen_R	outcome	4886_3_C(	MR Egger	72	-0.06201	0.05916	0.298174
4886_3_C(	finngen_R	outcome	4886_3_C(	Weighted	72	-0.09797	0.057887	0.090571
4886_3_C(	finngen_R	outcome	4886_3_C(	Inverse va	72	-0.0987	0.040202	0.01408
4886_3_C(	finngen_R	outcome	4886_3_C(	Simple mc	72	-0.04463	0.109081	0.683665
4886_3_C(	finngen_R	outcome	4886_3_C(	Weighted	72	-0.09478	0.053351	0.07993
4900_8_C3	finngen_R	outcome	4900_8_C3	Wald ratic	1	0.735176	0.539897	0.173294
4906_35_F	finngen_R	outcome	4906_35_F	MR Egger	36	-0.04384	0.106068	0.681943
4906_35_F	finngen_R	outcome	4906_35_F	Weighted	36	0.003562	0.101197	0.971921
4906_35_F	finngen_R	outcome	4906_35_F	Inverse va	36	0.013529	0.06642	0.838598
4906_35_F	finngen_R	outcome	4906_35_F	Simple mc	36	0.242625	0.197883	0.228344
4906_35_F	finngen_R	outcome	4906_35_F	Weighted	36	0.003488	0.085079	0.967535
4908_6_EN	finngen_R	outcome	4908_6_EN	MR Egger	17	0.070339	0.191699	0.718801
4908_6_EN	finngen_R	outcome	4908_6_EN	Weighted	17	0.126522	0.126925	0.31885
4908_6_EN	finngen_R	outcome	4908_6_EN	Inverse va	17	0.193838	0.105699	0.066675
4908_6_EN	finngen_R	outcome	4908_6_EN	Simple mc	17	-0.26844	0.266813	0.329337
4908_6_EN	finngen_R	outcome	4908_6_EN	Weighted	17	0.146988	0.131095	0.278737
4911_49_(	finngen_R	outcome	4911_49_(	MR Egger	10	-0.66472	0.380236	0.118562
4911_49_(	finngen_R	outcome	4911_49_(	Weighted	10	-0.37384	0.189107	0.048056
4911_49_(	finngen_R	outcome	4911_49_(	Inverse va	10	-0.41632	0.145641	0.004256
4911_49_(	finngen_R	outcome	4911_49_(	Simple mc	10	-0.34412	0.252819	0.206572
4911_49_(	finngen_R	outcome	4911_49_(	Weighted	10	-0.34929	0.235606	0.172355
4913_78_(	finngen_R	outcome	4913_78_(	MR Egger	79	-0.04251	0.06133	0.490274
4913_78_(	finngen_R	outcome	4913_78_(	Weighted	79	-0.07948	0.063431	0.21021
4913_78_(	finngen_R	outcome	4913_78_(	Inverse va	79	0.019509	0.03938	0.620317
4913_78_(	finngen_R	outcome	4913_78_(	Simple mc	79	-0.07613	0.137853	0.582377
4913_78_(	finngen_R	outcome	4913_78_(	Weighted	79	-0.13369	0.068068	0.053081
4920_10_L	finngen_R	outcome	4920_10_L	MR Egger	94	0.026166	0.074073	0.724718
4920_10_L	finngen_R	outcome	4920_10_L	Weighted	94	-0.00034	0.066185	0.995957
4920_10_L	finngen_R	outcome	4920_10_L	Inverse va	94	0.061643	0.04025	0.125642
4920_10_L	finngen_R	outcome	4920_10_L	Simple mc	94	-0.02109	0.136259	0.877318
4920_10_L	finngen_R	outcome	4920_10_L	Weighted	94	0.018002	0.059688	0.763631
4922_13_(	finngen_R	outcome	4922_13_(	Wald ratic	1	-0.40967	0.748803	0.584313
4924_32_N	finngen_R	outcome	4924_32_N	MR Egger	120	0.134173	0.058827	0.024354
4924_32_N	finngen_R	outcome	4924_32_N	Weighted	120	0.044887	0.048898	0.35863
4924_32_N	finngen_R	outcome	4924_32_N	Inverse va	120	0.019272	0.031494	0.54059
4924_32_N	finngen_R	outcome	4924_32_N	Simple mc	120	-0.06951	0.106991	0.517123

4924_32_N	finngen_R	outcome	4924_32_N	Weighted	120	0.030412	0.065166	0.641583
4925_54_N	finngen_R	outcome	4925_54_N	Wald ratic	1	-0.34303	0.59061	0.561368
4929_55_S	finngen_R	outcome	4929_55_S	MR Egger	17	0.052982	0.313603	0.868097
4929_55_S	finngen_R	outcome	4929_55_S	Weighted	17	0.047256	0.14842	0.750189
4929_55_S	finngen_R	outcome	4929_55_S	Inverse va	17	0.018492	0.106616	0.862305
4929_55_S	finngen_R	outcome	4929_55_S	Simple mc	17	0.053046	0.227183	0.818339
4929_55_S	finngen_R	outcome	4929_55_S	Weighted	17	0.050044	0.171212	0.77382
4930_21_S	finngen_R	outcome	4930_21_S	MR Egger	6	-0.091	0.614005	0.889356
4930_21_S	finngen_R	outcome	4930_21_S	Weighted	6	0.075343	0.334323	0.8217
4930_21_S	finngen_R	outcome	4930_21_S	Inverse va	6	0.267942	0.268541	0.31839
4930_21_S	finngen_R	outcome	4930_21_S	Simple mc	6	0.946279	0.54052	0.140399
4930_21_S	finngen_R	outcome	4930_21_S	Weighted	6	-0.11519	0.404632	0.787312
4960_72_A	finngen_R	outcome	4960_72_A	MR Egger	4	-0.13783	0.345573	0.728557
4960_72_A	finngen_R	outcome	4960_72_A	Weighted	4	-0.29332	0.232363	0.206828
4960_72_A	finngen_R	outcome	4960_72_A	Inverse va	4	-0.31142	0.207252	0.132944
4960_72_A	finngen_R	outcome	4960_72_A	Simple mc	4	-0.25914	0.332403	0.49248
4960_72_A	finngen_R	outcome	4960_72_A	Weighted	4	-0.28133	0.251443	0.344718
4961_17_A	finngen_R	outcome	4961_17_A	MR Egger	80	0.062743	0.06665	0.349418
4961_17_A	finngen_R	outcome	4961_17_A	Weighted	80	7.71E-05	0.063591	0.999033
4961_17_A	finngen_R	outcome	4961_17_A	Inverse va	80	0.0422	0.038656	0.274975
4961_17_A	finngen_R	outcome	4961_17_A	Simple mc	80	0.020316	0.125448	0.871762
4961_17_A	finngen_R	outcome	4961_17_A	Weighted	80	0.01251	0.057981	0.829731
4962_52_C	finngen_R	outcome	4962_52_C	MR Egger	34	-0.1212	0.115988	0.303894
4962_52_C	finngen_R	outcome	4962_52_C	Weighted	34	-0.07261	0.098892	0.462806
4962_52_C	finngen_R	outcome	4962_52_C	Inverse va	34	-0.02335	0.079122	0.767935
4962_52_C	finngen_R	outcome	4962_52_C	Simple mc	34	0.082662	0.244107	0.737034
4962_52_C	finngen_R	outcome	4962_52_C	Weighted	34	-0.08304	0.105119	0.435213
4964_67_E	finngen_R	outcome	4964_67_E	MR Egger	138	-0.00254	0.025458	0.92053
4964_67_E	finngen_R	outcome	4964_67_E	Weighted	138	-0.00535	0.023816	0.82234
4964_67_E	finngen_R	outcome	4964_67_E	Inverse va	138	-0.0015	0.015701	0.923824
4964_67_E	finngen_R	outcome	4964_67_E	Simple mc	138	0.006434	0.046636	0.890471
4964_67_E	finngen_R	outcome	4964_67_E	Weighted	138	-0.00334	0.022065	0.879881
4969_2_CA	finngen_R	outcome	4969_2_CA	MR Egger	9	0.106021	0.221886	0.647339
4969_2_CA	finngen_R	outcome	4969_2_CA	Weighted	9	0.17805	0.170064	0.29512
4969_2_CA	finngen_R	outcome	4969_2_CA	Inverse va	9	0.124528	0.12772	0.329556
4969_2_CA	finngen_R	outcome	4969_2_CA	Simple mc	9	0.2679	0.256826	0.327388
4969_2_CA	finngen_R	outcome	4969_2_CA	Weighted	9	0.164091	0.236395	0.507247
4971_1_CI	finngen_R	outcome	4971_1_CI	MR Egger	28	-0.06496	0.106367	0.546719
4971_1_CI	finngen_R	outcome	4971_1_CI	Weighted	28	-0.01	0.100962	0.921117
4971_1_CI	finngen_R	outcome	4971_1_CI	Inverse va	28	-0.05461	0.073094	0.455014
4971_1_CI	finngen_R	outcome	4971_1_CI	Simple mc	28	0.008939	0.150234	0.95299
4971_1_CI	finngen_R	outcome	4971_1_CI	Weighted	28	-0.00949	0.099248	0.924512
4979_34_D	finngen_R	outcome	4979_34_D	MR Egger	70	-0.13232	0.070228	0.063826
4979_34_D	finngen_R	outcome	4979_34_D	Weighted	70	-0.1471	0.069051	0.033143
4979_34_D	finngen_R	outcome	4979_34_D	Inverse va	70	-0.07558	0.043385	0.081484
4979_34_D	finngen_R	outcome	4979_34_D	Simple mc	70	-0.01306	0.116819	0.911276
4979_34_D	finngen_R	outcome	4979_34_D	Weighted	70	-0.13075	0.067723	0.05764
4982_54_F	finngen_R	outcome	4982_54_F	MR Egger	24	-0.20601	0.145543	0.170941
4982_54_F	finngen_R	outcome	4982_54_F	Weighted	24	-0.0863	0.118303	0.465702
4982_54_F	finngen_R	outcome	4982_54_F	Inverse va	24	-0.09168	0.079909	0.251238
4982_54_F	finngen_R	outcome	4982_54_F	Simple mc	24	0.105492	0.21457	0.627632
4982_54_F	finngen_R	outcome	4982_54_F	Weighted	24	-0.17799	0.125616	0.169914
4989_7_FG	finngen_R	outcome	4989_7_FG	MR Egger	5	-0.30062	0.397178	0.504129
4989_7_FG	finngen_R	outcome	4989_7_FG	Weighted	5	-0.39182	0.223055	0.078988
4989_7_FG	finngen_R	outcome	4989_7_FG	Inverse va	5	-0.34719	0.201808	0.08536
4989_7_FG	finngen_R	outcome	4989_7_FG	Simple mc	5	-0.40847	0.324457	0.276513
4989_7_FG	finngen_R	outcome	4989_7_FG	Weighted	5	-0.39301	0.23595	0.171111
4990_87_C	finngen_R	outcome	4990_87_C	MR Egger	17	-0.37363	0.196417	0.076521

4990_87_(	finngen_R	outcome	4990_87_(	Weighted	17	-0.12068	0.170518	0.479122
4990_87_(	finngen_R	outcome	4990_87_(	Inverse va	17	-0.08476	0.111073	0.445401
4990_87_(	finngen_R	outcome	4990_87_(	Simple mc	17	0.070714	0.302149	0.817924
4990_87_(	finngen_R	outcome	4990_87_(	Weighted	17	-0.27099	0.173512	0.137897
4991_12_(	finngen_R	outcome	4991_12_(	MR Egger	121	-0.05388	0.054507	0.324921
4991_12_(	finngen_R	outcome	4991_12_(	Weighted	121	-0.01473	0.057247	0.796956
4991_12_(	finngen_R	outcome	4991_12_(	Inverse va	121	-0.00744	0.033369	0.823527
4991_12_(	finngen_R	outcome	4991_12_(	Simple mc	121	-0.04163	0.090562	0.646574
4991_12_(	finngen_R	outcome	4991_12_(	Weighted	121	-0.02039	0.042166	0.629496
4992_49_(	finngen_R	outcome	4992_49_(	MR Egger	13	0.040658	0.31784	0.90052
4992_49_(	finngen_R	outcome	4992_49_(	Weighted	13	-0.22534	0.15391	0.143167
4992_49_(	finngen_R	outcome	4992_49_(	Inverse va	13	-0.27302	0.140414	0.05185
4992_49_(	finngen_R	outcome	4992_49_(	Simple mc	13	-0.45502	0.357087	0.226697
4992_49_(	finngen_R	outcome	4992_49_(	Weighted	13	-0.27887	0.160435	0.107738
4996_66_I	finngen_R	outcome	4996_66_I	MR Egger	141	-0.06114	0.044277	0.169515
4996_66_I	finngen_R	outcome	4996_66_I	Weighted	141	-0.07926	0.042926	0.064831
4996_66_I	finngen_R	outcome	4996_66_I	Inverse va	141	-0.0678	0.026951	0.011881
4996_66_I	finngen_R	outcome	4996_66_I	Simple mc	141	-0.11038	0.078348	0.161109
4996_66_I	finngen_R	outcome	4996_66_I	Weighted	141	-0.07222	0.041623	0.084932
5000_52_L	finngen_R	outcome	5000_52_L	MR Egger	18	-0.03129	0.225988	0.891617
5000_52_L	finngen_R	outcome	5000_52_L	Weighted	18	-0.0951	0.151646	0.530588
5000_52_L	finngen_R	outcome	5000_52_L	Inverse va	18	-0.11651	0.107374	0.277876
5000_52_L	finngen_R	outcome	5000_52_L	Simple mc	18	-0.17746	0.265262	0.512482
5000_52_L	finngen_R	outcome	5000_52_L	Weighted	18	0.004416	0.199961	0.982636
5005_4_M	finngen_R	outcome	5005_4_M	MR Egger	24	-0.01829	0.331417	0.956491
5005_4_M	finngen_R	outcome	5005_4_M	Weighted	24	0.060495	0.142675	0.671564
5005_4_M	finngen_R	outcome	5005_4_M	Inverse va	24	0.059103	0.104871	0.573039
5005_4_M	finngen_R	outcome	5005_4_M	Simple mc	24	0.054872	0.206454	0.792773
5005_4_M	finngen_R	outcome	5005_4_M	Weighted	24	0.046479	0.164602	0.780182
5006_71_N	finngen_R	outcome	5006_71_N	Inverse va	2	-0.60284	0.349488	0.084543
5015_15_F	finngen_R	outcome	5015_15_F	MR Egger	17	0.398335	0.415492	0.352905
5015_15_F	finngen_R	outcome	5015_15_F	Weighted	17	0.361039	0.209904	0.085429
5015_15_F	finngen_R	outcome	5015_15_F	Inverse va	17	0.277837	0.181955	0.126772
5015_15_F	finngen_R	outcome	5015_15_F	Simple mc	17	0.372588	0.2647	0.178385
5015_15_F	finngen_R	outcome	5015_15_F	Weighted	17	0.329534	0.234069	0.178307
5029_3_FA	finngen_R	outcome	5029_3_FA	MR Egger	10	0.176572	0.190882	0.382004
5029_3_FA	finngen_R	outcome	5029_3_FA	Weighted	10	0.145688	0.170968	0.394136
5029_3_FA	finngen_R	outcome	5029_3_FA	Inverse va	10	0.049128	0.138218	0.72226
5029_3_FA	finngen_R	outcome	5029_3_FA	Simple mc	10	-0.19244	0.320807	0.563398
5029_3_FA	finngen_R	outcome	5029_3_FA	Weighted	10	0.181156	0.172125	0.320021
5036_50_T	finngen_R	outcome	5036_50_T	MR Egger	79	0.014228	0.059337	0.811132
5036_50_T	finngen_R	outcome	5036_50_T	Weighted	79	-0.03227	0.054991	0.557374
5036_50_T	finngen_R	outcome	5036_50_T	Inverse va	79	0.004248	0.035139	0.903779
5036_50_T	finngen_R	outcome	5036_50_T	Simple mc	79	-0.06805	0.105314	0.520068
5036_50_T	finngen_R	outcome	5036_50_T	Weighted	79	-0.01919	0.047676	0.688388
5060_62_(	finngen_R	outcome	5060_62_(	MR Egger	23	0.001735	0.175917	0.992224
5060_62_(	finngen_R	outcome	5060_62_(	Weighted	23	-0.03994	0.135155	0.76759
5060_62_(	finngen_R	outcome	5060_62_(	Inverse va	23	-0.09757	0.090448	0.280689
5060_62_(	finngen_R	outcome	5060_62_(	Simple mc	23	0.115062	0.250866	0.65098
5060_62_(	finngen_R	outcome	5060_62_(	Weighted	23	-0.07775	0.137637	0.577887
5063_12_(	finngen_R	outcome	5063_12_(	Wald ratic	1	-0.37056	1.084692	0.73263
5066_134_	finngen_R	outcome	5066_134_	MR Egger	16	-0.05275	0.194957	0.790662
5066_134_	finngen_R	outcome	5066_134_	Weighted	16	-0.19266	0.157789	0.222095
5066_134_	finngen_R	outcome	5066_134_	Inverse va	16	-0.2145	0.135595	0.113669
5066_134_	finngen_R	outcome	5066_134_	Simple mc	16	-0.66644	0.275024	0.0285
5066_134_	finngen_R	outcome	5066_134_	Weighted	16	-0.15688	0.138791	0.276084
5069_9_CI	finngen_R	outcome	5069_9_CI	MR Egger	40	-0.05163	0.141016	0.716282
5069_9_CI	finngen_R	outcome	5069_9_CI	Weighted	40	-0.14697	0.101922	0.149314

5069_9_CI	finngen_R	outcome	5069_9_CI	Inverse va	40	-0.20653	0.071162	0.003705
5069_9_CI	finngen_R	outcome	5069_9_CI	Simple mc	40	-0.20843	0.172194	0.233403
5069_9_CI	finngen_R	outcome	5069_9_CI	Weighted	40	-0.15245	0.134141	0.26269
5076_53_E	finngen_R	outcome	5076_53_E	Inverse va	2	0.398502	0.676201	0.555644
5078_82_E	finngen_R	outcome	5078_82_E	MR Egger	26	-0.50078	0.216319	0.02948
5078_82_E	finngen_R	outcome	5078_82_E	Weighted	26	-0.35117	0.143725	0.014553
5078_82_E	finngen_R	outcome	5078_82_E	Inverse va	26	-0.32124	0.096181	0.000838
5078_82_E	finngen_R	outcome	5078_82_E	Simple mc	26	-0.48537	0.231326	0.046157
5078_82_E	finngen_R	outcome	5078_82_E	Weighted	26	-0.38129	0.13979	0.011498
5085_18_II	finngen_R	outcome	5085_18_II	Wald ratic	1	0.200412	0.661949	0.762073
5087_5_IL	finngen_R	outcome	5087_5_IL	MR Egger	15	0.120592	0.22075	0.594123
5087_5_IL	finngen_R	outcome	5087_5_IL	Weighted	15	-0.05301	0.17192	0.757801
5087_5_IL	finngen_R	outcome	5087_5_IL	Inverse va	15	0.068537	0.126269	0.587281
5087_5_IL	finngen_R	outcome	5087_5_IL	Simple mc	15	-0.02089	0.234624	0.930318
5087_5_IL	finngen_R	outcome	5087_5_IL	Weighted	15	-0.05038	0.201011	0.805725
5089_11_II	finngen_R	outcome	5089_11_II	Wald ratic	1	0.560923	0.642423	0.382589
5090_49_L	finngen_R	outcome	5090_49_L	MR Egger	154	-0.02958	0.033876	0.383941
5090_49_L	finngen_R	outcome	5090_49_L	Weighted	154	-0.0454	0.038845	0.242532
5090_49_L	finngen_R	outcome	5090_49_L	Inverse va	154	-0.01857	0.02405	0.439988
5090_49_L	finngen_R	outcome	5090_49_L	Simple mc	154	0.025477	0.094129	0.787016
5090_49_L	finngen_R	outcome	5090_49_L	Weighted	154	-0.0194	0.034791	0.577882
5091_28_L	finngen_R	outcome	5091_28_L	MR Egger	110	0.004157	0.035025	0.905734
5091_28_L	finngen_R	outcome	5091_28_L	Weighted	110	0.017607	0.035517	0.620091
5091_28_L	finngen_R	outcome	5091_28_L	Inverse va	110	-0.01569	0.026294	0.550596
5091_28_L	finngen_R	outcome	5091_28_L	Simple mc	110	0.058236	0.111203	0.601556
5091_28_L	finngen_R	outcome	5091_28_L	Weighted	110	0.001353	0.031442	0.965744
5092_51_J	finngen_R	outcome	5092_51_J	Inverse va	2	0.280094	0.846567	0.740751
5095_21_K	finngen_R	outcome	5095_21_K	MR Egger	23	-0.17961	0.16534	0.289649
5095_21_K	finngen_R	outcome	5095_21_K	Weighted	23	-0.14638	0.101777	0.150372
5095_21_K	finngen_R	outcome	5095_21_K	Inverse va	23	-0.17113	0.074579	0.021754
5095_21_K	finngen_R	outcome	5095_21_K	Simple mc	23	-0.2575	0.139726	0.078859
5095_21_K	finngen_R	outcome	5095_21_K	Weighted	23	-0.17048	0.099683	0.101293
5103_30_C	finngen_R	outcome	5103_30_C	MR Egger	4	2.22402	1.184326	0.201187
5103_30_C	finngen_R	outcome	5103_30_C	Weighted	4	0.317357	0.278303	0.254149
5103_30_C	finngen_R	outcome	5103_30_C	Inverse va	4	0.356924	0.244979	0.145128
5103_30_C	finngen_R	outcome	5103_30_C	Simple mc	4	-0.04892	0.445297	0.919451
5103_30_C	finngen_R	outcome	5103_30_C	Weighted	4	0.45019	0.298638	0.228797
5105_2_RT	finngen_R	outcome	5105_2_RT	MR Egger	54	0.120238	0.106285	0.263127
5105_2_RT	finngen_R	outcome	5105_2_RT	Weighted	54	0.082368	0.07301	0.25925
5105_2_RT	finngen_R	outcome	5105_2_RT	Inverse va	54	0.035078	0.052348	0.502799
5105_2_RT	finngen_R	outcome	5105_2_RT	Simple mc	54	0.080437	0.136332	0.557695
5105_2_RT	finngen_R	outcome	5105_2_RT	Weighted	54	0.080437	0.07732	0.302921
5107_7_N(	finngen_R	outcome	5107_7_N(	MR Egger	9	0.168508	0.417786	0.698737
5107_7_N(	finngen_R	outcome	5107_7_N(	Weighted	9	0.032986	0.221593	0.881664
5107_7_N(	finngen_R	outcome	5107_7_N(	Inverse va	9	0.169227	0.175766	0.335649
5107_7_N(	finngen_R	outcome	5107_7_N(	Simple mc	9	-0.03445	0.30853	0.913835
5107_7_N(	finngen_R	outcome	5107_7_N(	Weighted	9	0.009125	0.262666	0.973137
5108_72_N	finngen_R	outcome	5108_72_N	MR Egger	3	-0.71711	0.88537	0.5666
5108_72_N	finngen_R	outcome	5108_72_N	Weighted	3	-0.60397	0.38987	0.121341
5108_72_N	finngen_R	outcome	5108_72_N	Inverse va	3	-0.58263	0.345739	0.091954
5108_72_N	finngen_R	outcome	5108_72_N	Simple mc	3	-0.82242	0.45889	0.214974
5108_72_N	finngen_R	outcome	5108_72_N	Weighted	3	-0.78462	0.458027	0.228835
5111_15_N	finngen_R	outcome	5111_15_N	MR Egger	24	-0.09155	0.207176	0.662893
5111_15_N	finngen_R	outcome	5111_15_N	Weighted	24	0.054447	0.128577	0.671961
5111_15_N	finngen_R	outcome	5111_15_N	Inverse va	24	0.119371	0.093416	0.201306
5111_15_N	finngen_R	outcome	5111_15_N	Simple mc	24	0.100771	0.206714	0.630525
5111_15_N	finngen_R	outcome	5111_15_N	Weighted	24	0.032434	0.150712	0.831507
5112_73_C	finngen_R	outcome	5112_73_C	MR Egger	3	-0.12564	0.878671	0.909586

5112_73_(	finngen_R	outcome	5112_73_(	Weighted	3	-0.31661	0.333037	0.341769
5112_73_(	finngen_R	outcome	5112_73_(	Inverse va	3	-0.34386	0.291453	0.238082
5112_73_(	finngen_R	outcome	5112_73_(	Simple mc	3	-0.32319	0.405596	0.50911
5112_73_(	finngen_R	outcome	5112_73_(	Weighted	3	-0.29512	0.335033	0.471303
5121_3_SE	finngen_R	outcome	5121_3_SE	MR Egger	4	-0.7725	0.983434	0.514433
5121_3_SE	finngen_R	outcome	5121_3_SE	Weighted	4	0.212817	0.333058	0.522837
5121_3_SE	finngen_R	outcome	5121_3_SE	Inverse va	4	0.119421	0.279297	0.668959
5121_3_SE	finngen_R	outcome	5121_3_SE	Simple mc	4	0.342153	0.439715	0.493227
5121_3_SE	finngen_R	outcome	5121_3_SE	Weighted	4	0.197508	0.376644	0.636287
5128_53_S	finngen_R	outcome	5128_53_S	MR Egger	3	0.290359	0.559693	0.695339
5128_53_S	finngen_R	outcome	5128_53_S	Weighted	3	0.126156	0.191711	0.510506
5128_53_S	finngen_R	outcome	5128_53_S	Inverse va	3	0.092595	0.196653	0.637742
5128_53_S	finngen_R	outcome	5128_53_S	Simple mc	3	-0.00185	0.391849	0.996662
5128_53_S	finngen_R	outcome	5128_53_S	Weighted	3	0.139732	0.192848	0.544016
5131_15_T	finngen_R	outcome	5131_15_T	MR Egger	6	1.181718	0.719113	0.175666
5131_15_T	finngen_R	outcome	5131_15_T	Weighted	6	0.55921	0.297627	0.060259
5131_15_T	finngen_R	outcome	5131_15_T	Inverse va	6	0.276312	0.235703	0.241082
5131_15_T	finngen_R	outcome	5131_15_T	Simple mc	6	0.558676	0.482791	0.299473
5131_15_T	finngen_R	outcome	5131_15_T	Weighted	6	0.558676	0.371514	0.192964
5196_7_NI	finngen_R	outcome	5196_7_NI	Wald ratic	1	-0.39848	0.749445	0.594932
5202_4_PF	finngen_R	outcome	5202_4_PF	MR Egger	20	0.053968	0.165167	0.747627
5202_4_PF	finngen_R	outcome	5202_4_PF	Weighted	20	0.135888	0.112615	0.227563
5202_4_PF	finngen_R	outcome	5202_4_PF	Inverse va	20	0.026253	0.097339	0.787387
5202_4_PF	finngen_R	outcome	5202_4_PF	Simple mc	20	0.245679	0.232825	0.304564
5202_4_PF	finngen_R	outcome	5202_4_PF	Weighted	20	0.068942	0.104248	0.516346
5227_60_F	finngen_R	outcome	5227_60_F	MR Egger	13	0.056675	0.420551	0.895233
5227_60_F	finngen_R	outcome	5227_60_F	Weighted	13	0.305095	0.208207	0.142827
5227_60_F	finngen_R	outcome	5227_60_F	Inverse va	13	0.261056	0.154285	0.09064
5227_60_F	finngen_R	outcome	5227_60_F	Simple mc	13	0.330412	0.297887	0.289085
5227_60_F	finngen_R	outcome	5227_60_F	Weighted	13	0.330412	0.258056	0.224605
5231_79_F	finngen_R	outcome	5231_79_F	MR Egger	25	0.082148	0.098887	0.414668
5231_79_F	finngen_R	outcome	5231_79_F	Weighted	25	0.078491	0.09076	0.387141
5231_79_F	finngen_R	outcome	5231_79_F	Inverse va	25	-0.0173	0.067988	0.799087
5231_79_F	finngen_R	outcome	5231_79_F	Simple mc	25	-0.35381	0.200791	0.090786
5231_79_F	finngen_R	outcome	5231_79_F	Weighted	25	0.0602	0.089305	0.506697
5238_26_F	finngen_R	outcome	5238_26_F	MR Egger	29	0.578578	0.239266	0.02262
5238_26_F	finngen_R	outcome	5238_26_F	Weighted	29	0.301897	0.149891	0.043997
5238_26_F	finngen_R	outcome	5238_26_F	Inverse va	29	0.197556	0.100115	0.048462
5238_26_F	finngen_R	outcome	5238_26_F	Simple mc	29	0.149676	0.302956	0.625123
5238_26_F	finngen_R	outcome	5238_26_F	Weighted	29	0.301028	0.200856	0.145137
5256_86_F	finngen_R	outcome	5256_86_F	MR Egger	29	-0.04401	0.228323	0.848598
5256_86_F	finngen_R	outcome	5256_86_F	Weighted	29	-0.2857	0.142138	0.044429
5256_86_F	finngen_R	outcome	5256_86_F	Inverse va	29	-0.19963	0.097053	0.03969
5256_86_F	finngen_R	outcome	5256_86_F	Simple mc	29	-0.23548	0.250338	0.354926
5256_86_F	finngen_R	outcome	5256_86_F	Weighted	29	-0.26238	0.144218	0.079574
5301_7_C(	finngen_R	outcome	5301_7_C(	MR Egger	4	0.190092	1.830902	0.926782
5301_7_C(	finngen_R	outcome	5301_7_C(	Weighted	4	0.14798	0.350295	0.672702
5301_7_C(	finngen_R	outcome	5301_7_C(	Inverse va	4	-0.00632	0.284342	0.982255
5301_7_C(	finngen_R	outcome	5301_7_C(	Simple mc	4	0.129875	0.456829	0.794678
5301_7_C(	finngen_R	outcome	5301_7_C(	Weighted	4	0.234345	0.373186	0.574582
5316_54_F	finngen_R	outcome	5316_54_F	MR Egger	5	-1.37533	0.971647	0.251884
5316_54_F	finngen_R	outcome	5316_54_F	Weighted	5	-0.715	0.282948	0.011506
5316_54_F	finngen_R	outcome	5316_54_F	Inverse va	5	-0.66323	0.242972	0.00634
5316_54_F	finngen_R	outcome	5316_54_F	Simple mc	5	-0.63123	0.425846	0.212398
5316_54_F	finngen_R	outcome	5316_54_F	Weighted	5	-0.8163	0.326186	0.066584
5337_64_(	finngen_R	outcome	5337_64_(	Inverse va	2	-0.45207	0.483344	0.349631
5339_49_S	finngen_R	outcome	5339_49_S	Wald ratic	1	-1.03673	0.73503	0.158403
5350_14_(	finngen_R	outcome	5350_14_(	Inverse va	2	0.578631	0.47241	0.220632

5353_89_I	finngen_R	outcome	5353_89_I	MR Egger	18	-0.34367	0.234027	0.161357
5353_89_I	finngen_R	outcome	5353_89_I	Weighted	18	-0.12983	0.142061	0.360769
5353_89_I	finngen_R	outcome	5353_89_I	Inverse va	18	-0.08115	0.104531	0.437559
5353_89_I	finngen_R	outcome	5353_89_I	Simple mc	18	-0.20932	0.211339	0.33584
5353_89_I	finngen_R	outcome	5353_89_I	Weighted	18	-0.13535	0.124066	0.29051
5355_69_T	finngen_R	outcome	5355_69_T	MR Egger	8	-0.54353	0.394397	0.217354
5355_69_T	finngen_R	outcome	5355_69_T	Weighted	8	-0.0802	0.196385	0.682981
5355_69_T	finngen_R	outcome	5355_69_T	Inverse va	8	-0.08948	0.157124	0.569024
5355_69_T	finngen_R	outcome	5355_69_T	Simple mc	8	0.069447	0.28195	0.812509
5355_69_T	finngen_R	outcome	5355_69_T	Weighted	8	-0.11228	0.206224	0.603026
5358_3_OI	finngen_R	outcome	5358_3_OI	Inverse va	2	0.351727	1.223619	0.773769
5363_51_S	finngen_R	outcome	5363_51_S	MR Egger	142	-0.04123	0.043183	0.341301
5363_51_S	finngen_R	outcome	5363_51_S	Weighted	142	-0.01561	0.037071	0.673767
5363_51_S	finngen_R	outcome	5363_51_S	Inverse va	142	-0.01294	0.024147	0.59211
5363_51_S	finngen_R	outcome	5363_51_S	Simple mc	142	0.004559	0.069293	0.947633
5363_51_S	finngen_R	outcome	5363_51_S	Weighted	142	-0.03434	0.035957	0.341133
5392_73_F	finngen_R	outcome	5392_73_F	MR Egger	23	-0.15462	0.168197	0.368402
5392_73_F	finngen_R	outcome	5392_73_F	Weighted	23	0.081125	0.126384	0.520943
5392_73_F	finngen_R	outcome	5392_73_F	Inverse va	23	0.1697	0.096978	0.08014
5392_73_F	finngen_R	outcome	5392_73_F	Simple mc	23	0.272466	0.213493	0.215183
5392_73_F	finngen_R	outcome	5392_73_F	Weighted	23	0.08869	0.130534	0.503945
5400_52_L	finngen_R	outcome	5400_52_L	MR Egger	140	0.042846	0.03705	0.249502
5400_52_L	finngen_R	outcome	5400_52_L	Weighted	140	0.051233	0.036177	0.156721
5400_52_L	finngen_R	outcome	5400_52_L	Inverse va	140	0.032823	0.023496	0.162425
5400_52_L	finngen_R	outcome	5400_52_L	Simple mc	140	-0.00749	0.064823	0.908237
5400_52_L	finngen_R	outcome	5400_52_L	Weighted	140	0.04024	0.029908	0.180672
5404_53_T	finngen_R	outcome	5404_53_T	MR Egger	8	1.327151	0.770873	0.13592
5404_53_T	finngen_R	outcome	5404_53_T	Weighted	8	-0.18622	0.283321	0.511
5404_53_T	finngen_R	outcome	5404_53_T	Inverse va	8	-0.17247	0.272019	0.526046
5404_53_T	finngen_R	outcome	5404_53_T	Simple mc	8	-0.39669	0.431096	0.388085
5404_53_T	finngen_R	outcome	5404_53_T	Weighted	8	-0.13613	0.318918	0.682313
5430_66_S	finngen_R	outcome	5430_66_S	MR Egger	232	-0.01717	0.032928	0.602558
5430_66_S	finngen_R	outcome	5430_66_S	Weighted	232	0.006694	0.032214	0.83538
5430_66_S	finngen_R	outcome	5430_66_S	Inverse va	232	-0.00378	0.019528	0.846609
5430_66_S	finngen_R	outcome	5430_66_S	Simple mc	232	-0.02024	0.056414	0.720087
5430_66_S	finngen_R	outcome	5430_66_S	Weighted	232	-0.00093	0.030973	0.975976
5443_62_N	finngen_R	outcome	5443_62_N	Wald ratic	1	-0.28876	0.457328	0.527777
5451_1_Al	finngen_R	outcome	5451_1_Al	MR Egger	13	-0.29679	0.426334	0.500778
5451_1_Al	finngen_R	outcome	5451_1_Al	Weighted	13	0.390366	0.207248	0.059623
5451_1_Al	finngen_R	outcome	5451_1_Al	Inverse va	13	0.313975	0.171455	0.067065
5451_1_Al	finngen_R	outcome	5451_1_Al	Simple mc	13	0.533446	0.302519	0.103263
5451_1_Al	finngen_R	outcome	5451_1_Al	Weighted	13	0.469086	0.278362	0.117763
5452_71_A	finngen_R	outcome	5452_71_A	Inverse va	2	-0.05307	0.290504	0.855039
5456_59_C	finngen_R	outcome	5456_59_C	MR Egger	60	0.009518	0.098442	0.923308
5456_59_C	finngen_R	outcome	5456_59_C	Weighted	60	-0.11326	0.081845	0.166408
5456_59_C	finngen_R	outcome	5456_59_C	Inverse va	60	#######	0.052016	0.998603
5456_59_C	finngen_R	outcome	5456_59_C	Simple mc	60	-0.06336	0.17116	0.712581
5456_59_C	finngen_R	outcome	5456_59_C	Weighted	60	-0.03943	0.084726	0.643408
5459_33_C	finngen_R	outcome	5459_33_C	MR Egger	90	0.01348	0.069005	0.845576
5459_33_C	finngen_R	outcome	5459_33_C	Weighted	90	-0.02225	0.057951	0.700999
5459_33_C	finngen_R	outcome	5459_33_C	Inverse va	90	-0.01679	0.040263	0.676599
5459_33_C	finngen_R	outcome	5459_33_C	Simple mc	90	-0.02039	0.109614	0.852821
5459_33_C	finngen_R	outcome	5459_33_C	Weighted	90	-0.02039	0.052625	0.699273
5462_62_F	finngen_R	outcome	5462_62_F	MR Egger	21	0.200792	0.109313	0.081928
5462_62_F	finngen_R	outcome	5462_62_F	Weighted	21	0.114423	0.095047	0.228646
5462_62_F	finngen_R	outcome	5462_62_F	Inverse va	21	0.050934	0.092069	0.580119
5462_62_F	finngen_R	outcome	5462_62_F	Simple mc	21	0.182893	0.164909	0.280564
5462_62_F	finngen_R	outcome	5462_62_F	Weighted	21	0.127023	0.084446	0.148162

5475_10_F	finngen_R	outcome	5475_10_F	Wald ratic	1	-0.6513	0.576156	0.258297
5478_50_F	finngen_R	outcome	5478_50_F	MR Egger	3	-0.24629	0.329159	0.591055
5478_50_F	finngen_R	outcome	5478_50_F	Weighted	3	-0.3722	0.197809	0.05989
5478_50_F	finngen_R	outcome	5478_50_F	Inverse va	3	-0.38174	0.185615	0.039724
5478_50_F	finngen_R	outcome	5478_50_F	Simple mc	3	-0.40613	0.260745	0.259639
5478_50_F	finngen_R	outcome	5478_50_F	Weighted	3	-0.37994	0.248737	0.266213
5480_49_(	finngen_R	outcome	5480_49_(	MR Egger	9	0.563395	0.269347	0.074781
5480_49_(	finngen_R	outcome	5480_49_(	Weighted	9	0.392348	0.164968	0.017391
5480_49_(	finngen_R	outcome	5480_49_(	Inverse va	9	0.406505	0.140382	0.003783
5480_49_(	finngen_R	outcome	5480_49_(	Simple mc	9	0.506832	0.30781	0.138262
5480_49_(	finngen_R	outcome	5480_49_(	Weighted	9	0.391511	0.16728	0.047381
5487_7_SL	finngen_R	outcome	5487_7_SL	MR Egger	99	0.013055	0.045257	0.773601
5487_7_SL	finngen_R	outcome	5487_7_SL	Weighted	99	-0.00036	0.043219	0.99341
5487_7_SL	finngen_R	outcome	5487_7_SL	Inverse va	99	0.003987	0.027792	0.885936
5487_7_SL	finngen_R	outcome	5487_7_SL	Simple mc	99	-0.01637	0.076817	0.83165
5487_7_SL	finngen_R	outcome	5487_7_SL	Weighted	99	-0.00579	0.036064	0.872673
5491_12_S	finngen_R	outcome	5491_12_S	MR Egger	13	0.606812	0.27118	0.046892
5491_12_S	finngen_R	outcome	5491_12_S	Weighted	13	0.417289	0.157138	0.007918
5491_12_S	finngen_R	outcome	5491_12_S	Inverse va	13	0.3824	0.115403	0.000921
5491_12_S	finngen_R	outcome	5491_12_S	Simple mc	13	0.359869	0.243708	0.165526
5491_12_S	finngen_R	outcome	5491_12_S	Weighted	13	0.424571	0.166628	0.025559
5508_62_(	finngen_R	outcome	5508_62_(	MR Egger	39	0.196514	0.172962	0.263192
5508_62_(	finngen_R	outcome	5508_62_(	Weighted	39	0.112336	0.114986	0.32859
5508_62_(	finngen_R	outcome	5508_62_(	Inverse va	39	0.081986	0.082193	0.318535
5508_62_(	finngen_R	outcome	5508_62_(	Simple mc	39	0.273257	0.189524	0.157547
5508_62_(	finngen_R	outcome	5508_62_(	Weighted	39	0.172166	0.137599	0.218504
5509_7_E(	finngen_R	outcome	5509_7_E(	MR Egger	10	0.080935	0.379884	0.836616
5509_7_E(	finngen_R	outcome	5509_7_E(	Weighted	10	-0.02431	0.229247	0.915542
5509_7_E(	finngen_R	outcome	5509_7_E(	Inverse va	10	0.034622	0.176003	0.844053
5509_7_E(	finngen_R	outcome	5509_7_E(	Simple mc	10	0.072008	0.331076	0.832673
5509_7_E(	finngen_R	outcome	5509_7_E(	Weighted	10	-0.01476	0.242989	0.952894
5532_53_F	finngen_R	outcome	5532_53_F	MR Egger	5	0.92418	1.680596	0.620673
5532_53_F	finngen_R	outcome	5532_53_F	Weighted	5	-0.1443	0.322558	0.654611
5532_53_F	finngen_R	outcome	5532_53_F	Inverse va	5	-0.35732	0.344895	0.300186
5532_53_F	finngen_R	outcome	5532_53_F	Simple mc	5	-0.18939	0.405173	0.664528
5532_53_F	finngen_R	outcome	5532_53_F	Weighted	5	-0.14533	0.346034	0.696071
5542_22_N	finngen_R	outcome	5542_22_N	MR Egger	34	0.003573	0.13066	0.978355
5542_22_N	finngen_R	outcome	5542_22_N	Weighted	34	0.044806	0.098128	0.647953
5542_22_N	finngen_R	outcome	5542_22_N	Inverse va	34	-0.00262	0.064863	0.967799
5542_22_N	finngen_R	outcome	5542_22_N	Simple mc	34	-0.12579	0.168396	0.460361
5542_22_N	finngen_R	outcome	5542_22_N	Weighted	34	0.0441	0.093542	0.640421
5581_28_F	finngen_R	outcome	5581_28_F	MR Egger	115	0.007621	0.05272	0.885324
5581_28_F	finngen_R	outcome	5581_28_F	Weighted	115	0.060945	0.05422	0.260996
5581_28_F	finngen_R	outcome	5581_28_F	Inverse va	115	0.061581	0.035397	0.081909
5581_28_F	finngen_R	outcome	5581_28_F	Simple mc	115	0.020658	0.115901	0.858854
5581_28_F	finngen_R	outcome	5581_28_F	Weighted	115	0.05269	0.054753	0.337923
5584_21_T	finngen_R	outcome	5584_21_T	MR Egger	80	0.127691	0.054448	0.021561
5584_21_T	finngen_R	outcome	5584_21_T	Weighted	80	0.084042	0.050869	0.098506
5584_21_T	finngen_R	outcome	5584_21_T	Inverse va	80	0.078792	0.030765	0.010434
5584_21_T	finngen_R	outcome	5584_21_T	Simple mc	80	0.027288	0.086263	0.752584
5584_21_T	finngen_R	outcome	5584_21_T	Weighted	80	0.082214	0.048338	0.092915
5586_66_N	finngen_R	outcome	5586_66_N	MR Egger	6	-0.32846	0.987745	0.756183
5586_66_N	finngen_R	outcome	5586_66_N	Weighted	6	0.226761	0.308018	0.461612
5586_66_N	finngen_R	outcome	5586_66_N	Inverse va	6	0.487165	0.262456	0.063428
5586_66_N	finngen_R	outcome	5586_66_N	Simple mc	6	0.505096	0.509406	0.36695
5586_66_N	finngen_R	outcome	5586_66_N	Weighted	6	0.231875	0.341007	0.526724
5598_3_GI	finngen_R	outcome	5598_3_GI	Wald ratic	1	1.778827	0.899232	0.04791
5601_2_P(	finngen_R	outcome	5601_2_P(	MR Egger	40	-0.02268	0.117468	0.847945

5601_2_P(	finngen_R	outcome	5601_2_P(	Weighted	40	-0.07818	0.101952	0.443163
5601_2_P(	finngen_R	outcome	5601_2_P(	Inverse va	40	-0.01728	0.064524	0.788839
5601_2_P(	finngen_R	outcome	5601_2_P(	Simple mc	40	-0.17596	0.175051	0.320988
5601_2_P(	finngen_R	outcome	5601_2_P(	Weighted	40	-0.10162	0.099669	0.31421
5604_30_I	finngen_R	outcome	5604_30_I	MR Egger	4	-0.41275	1.402206	0.796227
5604_30_I	finngen_R	outcome	5604_30_I	Weighted	4	-0.15564	0.413032	0.7063
5604_30_I	finngen_R	outcome	5604_30_I	Inverse va	4	-0.20672	0.365133	0.571287
5604_30_I	finngen_R	outcome	5604_30_I	Simple mc	4	-0.81202	0.612044	0.276543
5604_30_I	finngen_R	outcome	5604_30_I	Weighted	4	0.128418	0.571713	0.836705
5605_77_N	finngen_R	outcome	5605_77_N	MR Egger	5	-0.07123	0.282458	0.817198
5605_77_N	finngen_R	outcome	5605_77_N	Weighted	5	-0.10491	0.205099	0.608991
5605_77_N	finngen_R	outcome	5605_77_N	Inverse va	5	-0.05812	0.181416	0.748704
5605_77_N	finngen_R	outcome	5605_77_N	Simple mc	5	-0.07371	0.343263	0.840483
5605_77_N	finngen_R	outcome	5605_77_N	Weighted	5	-0.10457	0.237119	0.682003
5609_92_F	finngen_R	outcome	5609_92_F	Wald ratic	1	-0.87492	0.704757	0.214441
5618_50_F	finngen_R	outcome	5618_50_F	MR Egger	16	-0.0266	0.163412	0.87304
5618_50_F	finngen_R	outcome	5618_50_F	Weighted	16	0.013909	0.141237	0.921551
5618_50_F	finngen_R	outcome	5618_50_F	Inverse va	16	0.081276	0.109537	0.45809
5618_50_F	finngen_R	outcome	5618_50_F	Simple mc	16	-0.22999	0.267529	0.403502
5618_50_F	finngen_R	outcome	5618_50_F	Weighted	16	0.01186	0.151423	0.938607
5620_13_F	finngen_R	outcome	5620_13_F	MR Egger	82	-0.00326	0.046524	0.944323
5620_13_F	finngen_R	outcome	5620_13_F	Weighted	82	-0.00535	0.04089	0.895906
5620_13_F	finngen_R	outcome	5620_13_F	Inverse va	82	-0.00125	0.02786	0.964154
5620_13_F	finngen_R	outcome	5620_13_F	Simple mc	82	0.077562	0.058149	0.185994
5620_13_F	finngen_R	outcome	5620_13_F	Weighted	82	-0.00742	0.036965	0.84134
5621_64_T	finngen_R	outcome	5621_64_T	MR Egger	35	0.059059	0.078287	0.455964
5621_64_T	finngen_R	outcome	5621_64_T	Weighted	35	-0.04838	0.075665	0.522549
5621_64_T	finngen_R	outcome	5621_64_T	Inverse va	35	0.01953	0.055318	0.724057
5621_64_T	finngen_R	outcome	5621_64_T	Simple mc	35	-0.17745	0.161125	0.278499
5621_64_T	finngen_R	outcome	5621_64_T	Weighted	35	-0.02975	0.074338	0.691509
5628_21_S	finngen_R	outcome	5628_21_S	MR Egger	22	0.049955	0.187718	0.792874
5628_21_S	finngen_R	outcome	5628_21_S	Weighted	22	-0.06964	0.13085	0.594597
5628_21_S	finngen_R	outcome	5628_21_S	Inverse va	22	-0.01515	0.09647	0.875209
5628_21_S	finngen_R	outcome	5628_21_S	Simple mc	22	-0.08333	0.204364	0.687579
5628_21_S	finngen_R	outcome	5628_21_S	Weighted	22	-0.05771	0.133715	0.670454
5630_48_(	finngen_R	outcome	5630_48_(	MR Egger	94	-0.14966	0.055514	0.008348
5630_48_(	finngen_R	outcome	5630_48_(	Weighted	94	-0.12585	0.062269	0.043277
5630_48_(	finngen_R	outcome	5630_48_(	Inverse va	94	-0.09697	0.035837	0.00681
5630_48_(	finngen_R	outcome	5630_48_(	Simple mc	94	-0.17935	0.114848	0.12177
5630_48_(	finngen_R	outcome	5630_48_(	Weighted	94	-0.13406	0.045838	0.004333
5631_83_N	finngen_R	outcome	5631_83_N	MR Egger	15	0.198802	0.370145	0.600276
5631_83_N	finngen_R	outcome	5631_83_N	Weighted	15	-0.18135	0.219728	0.409184
5631_83_N	finngen_R	outcome	5631_83_N	Inverse va	15	-0.05294	0.162515	0.744603
5631_83_N	finngen_R	outcome	5631_83_N	Simple mc	15	-0.1357	0.323976	0.681679
5631_83_N	finngen_R	outcome	5631_83_N	Weighted	15	-0.19378	0.286751	0.5102
5632_6_CI	finngen_R	outcome	5632_6_CI	MR Egger	53	-0.1792	0.110925	0.112371
5632_6_CI	finngen_R	outcome	5632_6_CI	Weighted	53	-0.10631	0.084652	0.209162
5632_6_CI	finngen_R	outcome	5632_6_CI	Inverse va	53	-0.08858	0.059853	0.138899
5632_6_CI	finngen_R	outcome	5632_6_CI	Simple mc	53	0.237453	0.194681	0.228082
5632_6_CI	finngen_R	outcome	5632_6_CI	Weighted	53	-0.06955	0.081451	0.397119
5636_10_N	finngen_R	outcome	5636_10_N	MR Egger	12	0.342796	0.163305	0.062169
5636_10_N	finngen_R	outcome	5636_10_N	Weighted	12	0.235911	0.149681	0.115005
5636_10_N	finngen_R	outcome	5636_10_N	Inverse va	12	0.092789	0.136554	0.496817
5636_10_N	finngen_R	outcome	5636_10_N	Simple mc	12	0.15264	0.268624	0.581293
5636_10_N	finngen_R	outcome	5636_10_N	Weighted	12	0.223452	0.13763	0.132753
5637_81_N	finngen_R	outcome	5637_81_N	MR Egger	70	-0.06028	0.051615	0.246903
5637_81_N	finngen_R	outcome	5637_81_N	Weighted	70	-0.05472	0.046726	0.241556
5637_81_N	finngen_R	outcome	5637_81_N	Inverse va	70	-0.03774	0.034635	0.275857

5637_81_N	finngen_R	outcome	5637_81_N	Simple mc	70	-0.04402	0.082378	0.594772
5637_81_N	finngen_R	outcome	5637_81_N	Weighted	70	-0.04402	0.039986	0.274729
5644_60_F	finngen_R	outcome	5644_60_F	MR Egger	40	-0.19461	0.112539	0.091886
5644_60_F	finngen_R	outcome	5644_60_F	Weighted	40	-0.10184	0.090052	0.258097
5644_60_F	finngen_R	outcome	5644_60_F	Inverse va	40	-0.08812	0.062324	0.157387
5644_60_F	finngen_R	outcome	5644_60_F	Simple mc	40	0.050086	0.169398	0.769052
5644_60_F	finngen_R	outcome	5644_60_F	Weighted	40	-0.11394	0.091103	0.218499
5646_20_F	finngen_R	outcome	5646_20_F	MR Egger	150	-0.16205	0.041718	0.000154
5646_20_F	finngen_R	outcome	5646_20_F	Weighted	150	-0.1252	0.040594	0.002042
5646_20_F	finngen_R	outcome	5646_20_F	Inverse va	150	-0.11669	0.02594	6.84E-06
5646_20_F	finngen_R	outcome	5646_20_F	Simple mc	150	-0.13774	0.073136	0.061611
5646_20_F	finngen_R	outcome	5646_20_F	Weighted	150	-0.12316	0.038774	0.001813
5648_28_C	finngen_R	outcome	5648_28_C	MR Egger	106	-0.00714	0.040137	0.859136
5648_28_C	finngen_R	outcome	5648_28_C	Weighted	106	-0.00537	0.040417	0.89427
5648_28_C	finngen_R	outcome	5648_28_C	Inverse va	106	0.031948	0.025409	0.208626
5648_28_C	finngen_R	outcome	5648_28_C	Simple mc	106	0.081774	0.075721	0.282645
5648_28_C	finngen_R	outcome	5648_28_C	Weighted	106	-0.00743	0.036537	0.839353
5656_53_E	finngen_R	outcome	5656_53_E	Inverse va	2	0.123221	0.469429	0.792943
5657_28_S	finngen_R	outcome	5657_28_S	MR Egger	29	0.124863	0.161747	0.446843
5657_28_S	finngen_R	outcome	5657_28_S	Weighted	29	0.000431	0.121552	0.997169
5657_28_S	finngen_R	outcome	5657_28_S	Inverse va	29	-0.02091	0.079827	0.793384
5657_28_S	finngen_R	outcome	5657_28_S	Simple mc	29	0.095798	0.210802	0.653012
5657_28_S	finngen_R	outcome	5657_28_S	Weighted	29	0.06044	0.118044	0.612655
5658_64_F	finngen_R	outcome	5658_64_F	MR Egger	37	0.139841	0.10448	0.18938
5658_64_F	finngen_R	outcome	5658_64_F	Weighted	37	-0.00704	0.089939	0.937637
5658_64_F	finngen_R	outcome	5658_64_F	Inverse va	37	-0.0218	0.057849	0.706231
5658_64_F	finngen_R	outcome	5658_64_F	Simple mc	37	-0.04821	0.160465	0.765577
5658_64_F	finngen_R	outcome	5658_64_F	Weighted	37	0.001167	0.08443	0.98905
5660_51_S	finngen_R	outcome	5660_51_S	MR Egger	91	0.016643	0.058792	0.77777
5660_51_S	finngen_R	outcome	5660_51_S	Weighted	91	0.017511	0.055548	0.752575
5660_51_S	finngen_R	outcome	5660_51_S	Inverse va	91	0.000561	0.037964	0.988206
5660_51_S	finngen_R	outcome	5660_51_S	Simple mc	91	0.013758	0.097745	0.888377
5660_51_S	finngen_R	outcome	5660_51_S	Weighted	91	0.022442	0.06046	0.711368
5663_18_F	finngen_R	outcome	5663_18_F	MR Egger	21	-0.11818	0.130935	0.378034
5663_18_F	finngen_R	outcome	5663_18_F	Weighted	21	-0.12443	0.108202	0.250153
5663_18_F	finngen_R	outcome	5663_18_F	Inverse va	21	-0.07573	0.076265	0.320706
5663_18_F	finngen_R	outcome	5663_18_F	Simple mc	21	0.063568	0.218917	0.774517
5663_18_F	finngen_R	outcome	5663_18_F	Weighted	21	-0.16383	0.105659	0.1367
5671_1_C1	finngen_R	outcome	5671_1_C1	MR Egger	53	0.057409	0.083408	0.494384
5671_1_C1	finngen_R	outcome	5671_1_C1	Weighted	53	0.108484	0.075995	0.153428
5671_1_C1	finngen_R	outcome	5671_1_C1	Inverse va	53	0.080152	0.052714	0.128379
5671_1_C1	finngen_R	outcome	5671_1_C1	Simple mc	53	0.15039	0.152256	0.327852
5671_1_C1	finngen_R	outcome	5671_1_C1	Weighted	53	0.126056	0.0821	0.13075
5676_54_A	finngen_R	outcome	5676_54_A	MR Egger	20	-0.20539	0.143943	0.170729
5676_54_A	finngen_R	outcome	5676_54_A	Weighted	20	-0.19676	0.116931	0.092437
5676_54_A	finngen_R	outcome	5676_54_A	Inverse va	20	-0.17943	0.082072	0.028799
5676_54_A	finngen_R	outcome	5676_54_A	Simple mc	20	-0.13428	0.186043	0.479211
5676_54_A	finngen_R	outcome	5676_54_A	Weighted	20	-0.16062	0.138434	0.260314
5680_54_C	finngen_R	outcome	5680_54_C	MR Egger	35	0.052928	0.139086	0.705983
5680_54_C	finngen_R	outcome	5680_54_C	Weighted	35	-0.13211	0.092887	0.154947
5680_54_C	finngen_R	outcome	5680_54_C	Inverse va	35	-0.12046	0.085676	0.15974
5680_54_C	finngen_R	outcome	5680_54_C	Simple mc	35	0.041734	0.241417	0.863777
5680_54_C	finngen_R	outcome	5680_54_C	Weighted	35	-0.06838	0.096951	0.48545
5682_13_\	finngen_R	outcome	5682_13_\	MR Egger	3	1.481349	1.357318	0.472201
5682_13_\	finngen_R	outcome	5682_13_\	Weighted	3	0.071776	0.378744	0.849693
5682_13_\	finngen_R	outcome	5682_13_\	Inverse va	3	0.20448	0.353652	0.563132
5682_13_\	finngen_R	outcome	5682_13_\	Simple mc	3	0.069536	0.493861	0.900928
5682_13_\	finngen_R	outcome	5682_13_\	Weighted	3	0.05154	0.434675	0.916451

5687_5_PI	finngen_R	outcome	5687_5_PI	MR Egger	3	-0.07147	1.609804	0.971753
5687_5_PI	finngen_R	outcome	5687_5_PI	Weighted	3	-0.41018	0.33818	0.225171
5687_5_PI	finngen_R	outcome	5687_5_PI	Inverse va	3	-0.44274	0.340949	0.1941
5687_5_PI	finngen_R	outcome	5687_5_PI	Simple mc	3	-0.23872	0.5134	0.687657
5687_5_PI	finngen_R	outcome	5687_5_PI	Weighted	3	-0.39748	0.346669	0.370232
5688_65_(	finngen_R	outcome	5688_65_(	MR Egger	35	0.071448	0.333565	0.831714
5688_65_(	finngen_R	outcome	5688_65_(	Weighted	35	0.091433	0.125198	0.465204
5688_65_(	finngen_R	outcome	5688_65_(	Inverse va	35	0.134046	0.107456	0.212232
5688_65_(	finngen_R	outcome	5688_65_(	Simple mc	35	0.23581	0.195139	0.235223
5688_65_(	finngen_R	outcome	5688_65_(	Weighted	35	0.193998	0.146586	0.19452
5691_2_CI	finngen_R	outcome	5691_2_CI	MR Egger	43	0.190264	0.115255	0.10642
5691_2_CI	finngen_R	outcome	5691_2_CI	Weighted	43	0.018321	0.096949	0.850111
5691_2_CI	finngen_R	outcome	5691_2_CI	Inverse va	43	0.071555	0.063592	0.2605
5691_2_CI	finngen_R	outcome	5691_2_CI	Simple mc	43	-0.05828	0.165605	0.726649
5691_2_CI	finngen_R	outcome	5691_2_CI	Weighted	43	-0.00639	0.101324	0.949991
5701_81_(	finngen_R	outcome	5701_81_(	MR Egger	18	-0.03369	0.28301	0.906722
5701_81_(	finngen_R	outcome	5701_81_(	Weighted	18	0.114463	0.178475	0.521301
5701_81_(	finngen_R	outcome	5701_81_(	Inverse va	18	0.143031	0.121786	0.240219
5701_81_(	finngen_R	outcome	5701_81_(	Simple mc	18	0.146399	0.307385	0.639945
5701_81_(	finngen_R	outcome	5701_81_(	Weighted	18	0.146399	0.187942	0.446713
5704_74_(	finngen_R	outcome	5704_74_(	MR Egger	3	-0.77473	3.781463	0.871352
5704_74_(	finngen_R	outcome	5704_74_(	Weighted	3	-0.24016	0.538515	0.655619
5704_74_(	finngen_R	outcome	5704_74_(	Inverse va	3	-0.34676	0.580652	0.550383
5704_74_(	finngen_R	outcome	5704_74_(	Simple mc	3	0.010017	0.801888	0.991167
5704_74_(	finngen_R	outcome	5704_74_(	Weighted	3	0.17079	0.939859	0.872554
5708_1_LE	finngen_R	outcome	5708_1_LE	MR Egger	37	0.136841	0.131383	0.304767
5708_1_LE	finngen_R	outcome	5708_1_LE	Weighted	37	0.12876	0.117354	0.272555
5708_1_LE	finngen_R	outcome	5708_1_LE	Inverse va	37	0.108522	0.075698	0.151679
5708_1_LE	finngen_R	outcome	5708_1_LE	Simple mc	37	0.030475	0.20451	0.882372
5708_1_LE	finngen_R	outcome	5708_1_LE	Weighted	37	0.123524	0.112181	0.278157
5713_9_IFI	finngen_R	outcome	5713_9_IFI	Wald ratic	1	0.006829	0.710765	0.992335
5722_78_F	finngen_R	outcome	5722_78_F	MR Egger	14	-0.35618	0.289802	0.242613
5722_78_F	finngen_R	outcome	5722_78_F	Weighted	14	-0.0395	0.178614	0.824966
5722_78_F	finngen_R	outcome	5722_78_F	Inverse va	14	0.208995	0.13749	0.128493
5722_78_F	finngen_R	outcome	5722_78_F	Simple mc	14	0.062259	0.349361	0.861307
5722_78_F	finngen_R	outcome	5722_78_F	Weighted	14	0.003427	0.213279	0.987423
5728_60_F	finngen_R	outcome	5728_60_F	MR Egger	55	0.037109	0.102647	0.719144
5728_60_F	finngen_R	outcome	5728_60_F	Weighted	55	-0.04612	0.088195	0.601034
5728_60_F	finngen_R	outcome	5728_60_F	Inverse va	55	-0.05926	0.054142	0.273706
5728_60_F	finngen_R	outcome	5728_60_F	Simple mc	55	-0.32634	0.15344	0.038019
5728_60_F	finngen_R	outcome	5728_60_F	Weighted	55	-0.05025	0.078535	0.524989
5731_1_SF	finngen_R	outcome	5731_1_SF	MR Egger	64	-0.07225	0.046159	0.122616
5731_1_SF	finngen_R	outcome	5731_1_SF	Weighted	64	-0.05169	0.047737	0.278924
5731_1_SF	finngen_R	outcome	5731_1_SF	Inverse va	64	-0.05406	0.034153	0.113472
5731_1_SF	finngen_R	outcome	5731_1_SF	Simple mc	64	-0.10262	0.102953	0.322685
5731_1_SF	finngen_R	outcome	5731_1_SF	Weighted	64	-0.06508	0.040598	0.113916
5734_13_F	finngen_R	outcome	5734_13_F	Wald ratic	1	-1.17577	0.649934	0.070441
5737_61_S	finngen_R	outcome	5737_61_S	MR Egger	65	0.019126	0.045844	0.677958
5737_61_S	finngen_R	outcome	5737_61_S	Weighted	65	0.055777	0.045137	0.216557
5737_61_S	finngen_R	outcome	5737_61_S	Inverse va	65	0.042177	0.029943	0.158965
5737_61_S	finngen_R	outcome	5737_61_S	Simple mc	65	0.046992	0.073088	0.522553
5737_61_S	finngen_R	outcome	5737_61_S	Weighted	65	0.046992	0.03808	0.221705
5742_14_A	finngen_R	outcome	5742_14_A	MR Egger	82	0.001099	0.049841	0.982455
5742_14_A	finngen_R	outcome	5742_14_A	Weighted	82	-0.04973	0.043004	0.247567
5742_14_A	finngen_R	outcome	5742_14_A	Inverse va	82	-0.08458	0.025991	0.001137
5742_14_A	finngen_R	outcome	5742_14_A	Simple mc	82	-0.03821	0.073172	0.602998
5742_14_A	finngen_R	outcome	5742_14_A	Weighted	82	-0.06103	0.043686	0.166243
5749_53_(	finngen_R	outcome	5749_53_(	MR Egger	79	0.029237	0.104398	0.780185

5749_53_(	finngen_R	outcome	5749_53_(	Weighted	79	-0.00711	0.081647	0.930619
5749_53_(	finngen_R	outcome	5749_53_(	Inverse va	79	-0.01454	0.055404	0.792997
5749_53_(	finngen_R	outcome	5749_53_(	Simple mc	79	-0.14488	0.146226	0.324847
5749_53_(	finngen_R	outcome	5749_53_(	Weighted	79	0.016669	0.081277	0.838034
5763_67_[	finngen_R	outcome	5763_67_[	MR Egger	22	-0.05339	0.312919	0.866227
5763_67_[	finngen_R	outcome	5763_67_[	Weighted	22	-0.16533	0.206424	0.423171
5763_67_[	finngen_R	outcome	5763_67_[	Inverse va	22	-0.04791	0.154956	0.757164
5763_67_[	finngen_R	outcome	5763_67_[	Simple mc	22	-0.18968	0.370857	0.614356
5763_67_[	finngen_R	outcome	5763_67_[	Weighted	22	-0.15099	0.320975	0.642921
5803_24_(	finngen_R	outcome	5803_24_(	MR Egger	3	-0.25984	4.061469	0.959326
5803_24_(	finngen_R	outcome	5803_24_(	Weighted	3	0.526365	0.284653	0.064437
5803_24_(	finngen_R	outcome	5803_24_(	Inverse va	3	0.4687	0.245191	0.055931
5803_24_(	finngen_R	outcome	5803_24_(	Simple mc	3	0.578368	0.333993	0.22547
5803_24_(	finngen_R	outcome	5803_24_(	Weighted	3	0.569648	0.353391	0.248292
5810_25_T	finngen_R	outcome	5810_25_T	MR Egger	88	-0.04218	0.030609	0.171731
5810_25_T	finngen_R	outcome	5810_25_T	Weighted	88	-0.00418	0.029888	0.888894
5810_25_T	finngen_R	outcome	5810_25_T	Inverse va	88	0.020378	0.017958	0.256456
5810_25_T	finngen_R	outcome	5810_25_T	Simple mc	88	0.093551	0.065924	0.159448
5810_25_T	finngen_R	outcome	5810_25_T	Weighted	88	-0.00362	0.025332	0.886746
5825_49_I	finngen_R	outcome	5825_49_I	Inverse va	2	0.629596	0.525708	0.231067
5834_18_I	finngen_R	outcome	5834_18_I	Wald ratic	1	0.263199	0.636202	0.679091
5837_49_L	finngen_R	outcome	5837_49_L	MR Egger	18	-0.01371	0.291447	0.963053
5837_49_L	finngen_R	outcome	5837_49_L	Weighted	18	-0.27789	0.159544	0.08155
5837_49_L	finngen_R	outcome	5837_49_L	Inverse va	18	-0.21131	0.145371	0.146059
5837_49_L	finngen_R	outcome	5837_49_L	Simple mc	18	-0.40064	0.282999	0.174922
5837_49_L	finngen_R	outcome	5837_49_L	Weighted	18	-0.34325	0.163543	0.051077
5852_6_S1	finngen_R	outcome	5852_6_S1	MR Egger	11	-0.10654	0.266269	0.69839
5852_6_S1	finngen_R	outcome	5852_6_S1	Weighted	11	-0.27977	0.158923	0.078334
5852_6_S1	finngen_R	outcome	5852_6_S1	Inverse va	11	-0.27446	0.135012	0.042065
5852_6_S1	finngen_R	outcome	5852_6_S1	Simple mc	11	-0.42228	0.287369	0.172447
5852_6_S1	finngen_R	outcome	5852_6_S1	Weighted	11	-0.29408	0.166414	0.107651
5939_42_T	finngen_R	outcome	5939_42_T	MR Egger	30	0.16379	0.117682	0.174942
5939_42_T	finngen_R	outcome	5939_42_T	Weighted	30	0.064773	0.097741	0.507522
5939_42_T	finngen_R	outcome	5939_42_T	Inverse va	30	-0.01334	0.066541	0.841059
5939_42_T	finngen_R	outcome	5939_42_T	Simple mc	30	-0.01506	0.16926	0.929707
5939_42_T	finngen_R	outcome	5939_42_T	Weighted	30	0.083015	0.087333	0.349682
5963_9_DI	finngen_R	outcome	5963_9_DI	MR Egger	4	-1.46758	1.102149	0.314488
5963_9_DI	finngen_R	outcome	5963_9_DI	Weighted	4	0.00991	0.389379	0.979696
5963_9_DI	finngen_R	outcome	5963_9_DI	Inverse va	4	0.295855	0.415591	0.476534
5963_9_DI	finngen_R	outcome	5963_9_DI	Simple mc	4	-0.12473	0.507315	0.821652
5963_9_DI	finngen_R	outcome	5963_9_DI	Weighted	4	-0.10372	0.444083	0.830361
5980_55_E	finngen_R	outcome	5980_55_E	Inverse va	2	0.514082	0.265707	0.053019
5988_49_T	finngen_R	outcome	5988_49_T	Wald ratic	1	-0.95735	0.646528	0.13867
6039_24_(	finngen_R	outcome	6039_24_(	MR Egger	83	0.064019	0.068814	0.35497
6039_24_(	finngen_R	outcome	6039_24_(	Weighted	83	0.011625	0.067426	0.863113
6039_24_(	finngen_R	outcome	6039_24_(	Inverse va	83	-0.04597	0.039864	0.248812
6039_24_(	finngen_R	outcome	6039_24_(	Simple mc	83	0.011285	0.113107	0.92077
6039_24_(	finngen_R	outcome	6039_24_(	Weighted	83	0.022142	0.063542	0.728388
6049_64_F	finngen_R	outcome	6049_64_F	MR Egger	3	-0.11089	0.535415	0.869993
6049_64_F	finngen_R	outcome	6049_64_F	Weighted	3	0.092646	0.262417	0.724051
6049_64_F	finngen_R	outcome	6049_64_F	Inverse va	3	0.114559	0.25199	0.649384
6049_64_F	finngen_R	outcome	6049_64_F	Simple mc	3	0.20224	0.388638	0.654671
6049_64_F	finngen_R	outcome	6049_64_F	Weighted	3	0.045393	0.312296	0.897758
6060_2_PI	finngen_R	outcome	6060_2_PI	MR Egger	46	0.059199	0.058204	0.314667
6060_2_PI	finngen_R	outcome	6060_2_PI	Weighted	46	0.113907	0.068725	0.097431
6060_2_PI	finngen_R	outcome	6060_2_PI	Inverse va	46	0.048132	0.041937	0.251079
6060_2_PI	finngen_R	outcome	6060_2_PI	Simple mc	46	0.126988	0.127836	0.325845
6060_2_PI	finngen_R	outcome	6060_2_PI	Weighted	46	0.070127	0.057535	0.229243

6077_63_(	finngen_R	outcome	6077_63_(	MR Egger	78	0.117448	0.072266	0.108253
6077_63_(	finngen_R	outcome	6077_63_(	Weighted	78	0.05081	0.060107	0.397932
6077_63_(	finngen_R	outcome	6077_63_(	Inverse va	78	0.089619	0.039284	0.022531
6077_63_(	finngen_R	outcome	6077_63_(	Simple mc	78	0.041438	0.115528	0.720814
6077_63_(	finngen_R	outcome	6077_63_(	Weighted	78	0.033228	0.065657	0.614245
6081_52_F	finngen_R	outcome	6081_52_F	MR Egger	74	0.07368	0.068346	0.284608
6081_52_F	finngen_R	outcome	6081_52_F	Weighted	74	0.079085	0.07213	0.272898
6081_52_F	finngen_R	outcome	6081_52_F	Inverse va	74	0.103914	0.043206	0.016168
6081_52_F	finngen_R	outcome	6081_52_F	Simple mc	74	0.068531	0.129404	0.598006
6081_52_F	finngen_R	outcome	6081_52_F	Weighted	74	0.068531	0.070527	0.334414
6086_15_(	finngen_R	outcome	6086_15_(	MR Egger	26	0.341849	0.129475	0.014334
6086_15_(	finngen_R	outcome	6086_15_(	Weighted	26	0.225305	0.10832	0.037526
6086_15_(	finngen_R	outcome	6086_15_(	Inverse va	26	0.234723	0.072287	0.001166
6086_15_(	finngen_R	outcome	6086_15_(	Simple mc	26	0.321581	0.164212	0.061438
6086_15_(	finngen_R	outcome	6086_15_(	Weighted	26	0.224428	0.107678	0.047511
6151_18_N	finngen_R	outcome	6151_18_N	Wald ratic	1	-1.19419	0.781712	0.126596
6207_10_F	finngen_R	outcome	6207_10_F	MR Egger	43	0.015252	0.105036	0.885256
6207_10_F	finngen_R	outcome	6207_10_F	Weighted	43	0.003906	0.084372	0.963074
6207_10_F	finngen_R	outcome	6207_10_F	Inverse va	43	0.074399	0.054202	0.169871
6207_10_F	finngen_R	outcome	6207_10_F	Simple mc	43	-0.03785	0.147285	0.798455
6207_10_F	finngen_R	outcome	6207_10_F	Weighted	43	-0.00382	0.07953	0.961935
6223_5_Gl	finngen_R	outcome	6223_5_Gl	MR Egger	7	0.343163	0.398684	0.428716
6223_5_Gl	finngen_R	outcome	6223_5_Gl	Weighted	7	0.257076	0.233644	0.271207
6223_5_Gl	finngen_R	outcome	6223_5_Gl	Inverse va	7	0.30553	0.19443	0.116087
6223_5_Gl	finngen_R	outcome	6223_5_Gl	Simple mc	7	0.381693	0.346607	0.313002
6223_5_Gl	finngen_R	outcome	6223_5_Gl	Weighted	7	0.162609	0.299298	0.606503
6227_1_KL	finngen_R	outcome	6227_1_KL	MR Egger	120	0.056963	0.054138	0.294866
6227_1_KL	finngen_R	outcome	6227_1_KL	Weighted	120	0.068878	0.05598	0.218543
6227_1_KL	finngen_R	outcome	6227_1_KL	Inverse va	120	0.039681	0.0342	0.245945
6227_1_KL	finngen_R	outcome	6227_1_KL	Simple mc	120	-0.16414	0.105455	0.122238
6227_1_KL	finngen_R	outcome	6227_1_KL	Weighted	120	0.048804	0.047712	0.308445
6234_74_\	finngen_R	outcome	6234_74_\	MR Egger	76	0.155615	0.080685	0.057607
6234_74_\	finngen_R	outcome	6234_74_\	Weighted	76	0.049942	0.065264	0.444133
6234_74_\	finngen_R	outcome	6234_74_\	Inverse va	76	0.014112	0.042349	0.738967
6234_74_\	finngen_R	outcome	6234_74_\	Simple mc	76	0.113543	0.127873	0.377415
6234_74_\	finngen_R	outcome	6234_74_\	Weighted	76	0.066911	0.068849	0.334251
6245_4_P\	finngen_R	outcome	6245_4_P\	Inverse va	2	-0.77382	0.58725	0.187602
6247_9_SII	finngen_R	outcome	6247_9_SII	MR Egger	255	-0.06455	0.024973	0.010305
6247_9_SII	finngen_R	outcome	6247_9_SII	Weighted	255	-0.05451	0.029083	0.060867
6247_9_SII	finngen_R	outcome	6247_9_SII	Inverse va	255	-0.04621	0.016344	0.004698
6247_9_SII	finngen_R	outcome	6247_9_SII	Simple mc	255	-0.10611	0.054783	0.053859
6247_9_SII	finngen_R	outcome	6247_9_SII	Weighted	255	-0.06594	0.026402	0.013135
6252_62_S	finngen_R	outcome	6252_62_S	MR Egger	9	0.383546	0.290189	0.227819
6252_62_S	finngen_R	outcome	6252_62_S	Weighted	9	-0.03719	0.192047	0.84643
6252_62_S	finngen_R	outcome	6252_62_S	Inverse va	9	0.064649	0.151557	0.669696
6252_62_S	finngen_R	outcome	6252_62_S	Simple mc	9	-0.08514	0.288022	0.775072
6252_62_S	finngen_R	outcome	6252_62_S	Weighted	9	-0.02114	0.235515	0.930668
6255_74_(	finngen_R	outcome	6255_74_(	MR Egger	70	-0.01193	0.08159	0.884216
6255_74_(	finngen_R	outcome	6255_74_(	Weighted	70	0.045283	0.081988	0.580737
6255_74_(	finngen_R	outcome	6255_74_(	Inverse va	70	0.002735	0.049057	0.955533
6255_74_(	finngen_R	outcome	6255_74_(	Simple mc	70	0.048604	0.146673	0.741362
6255_74_(	finngen_R	outcome	6255_74_(	Weighted	70	0.033369	0.074323	0.654864
6342_10_N	finngen_R	outcome	6342_10_N	MR Egger	11	-0.33734	0.400697	0.421666
6342_10_N	finngen_R	outcome	6342_10_N	Weighted	11	-0.12445	0.182369	0.494994
6342_10_N	finngen_R	outcome	6342_10_N	Inverse va	11	-0.06535	0.146286	0.655094
6342_10_N	finngen_R	outcome	6342_10_N	Simple mc	11	0.305504	0.338389	0.387852
6342_10_N	finngen_R	outcome	6342_10_N	Weighted	11	-0.10721	0.188631	0.582333
6364_7_TA	finngen_R	outcome	6364_7_TA	MR Egger	137	-0.0371	0.02966	0.213181

6364_7_TA	finngen_R	outcome	6364_7_TA	Weighted	137	-0.02524	0.033664	0.453474
6364_7_TA	finngen_R	outcome	6364_7_TA	Inverse va	137	0.005371	0.019404	0.781957
6364_7_TA	finngen_R	outcome	6364_7_TA	Simple mc	137	-0.06802	0.053451	0.205335
6364_7_TA	finngen_R	outcome	6364_7_TA	Weighted	137	-0.04663	0.029016	0.110373
6367_66_F	finngen_R	outcome	6367_66_F	MR Egger	10	0.007309	0.287304	0.980327
6367_66_F	finngen_R	outcome	6367_66_F	Weighted	10	-0.25472	0.201374	0.20591
6367_66_F	finngen_R	outcome	6367_66_F	Inverse va	10	-0.33744	0.180328	0.061309
6367_66_F	finngen_R	outcome	6367_66_F	Simple mc	10	-0.39412	0.296223	0.216079
6367_66_F	finngen_R	outcome	6367_66_F	Weighted	10	-0.26087	0.196682	0.217394
6373_54_S	finngen_R	outcome	6373_54_S	MR Egger	73	-0.03896	0.071761	0.588919
6373_54_S	finngen_R	outcome	6373_54_S	Weighted	73	0.058588	0.060123	0.329826
6373_54_S	finngen_R	outcome	6373_54_S	Inverse va	73	0.021088	0.038418	0.583063
6373_54_S	finngen_R	outcome	6373_54_S	Simple mc	73	-0.00871	0.104812	0.933975
6373_54_S	finngen_R	outcome	6373_54_S	Weighted	73	0.034365	0.052817	0.517342
6382_17_M	finngen_R	outcome	6382_17_M	MR Egger	57	0.000298	0.090181	0.997375
6382_17_M	finngen_R	outcome	6382_17_M	Weighted	57	0.020592	0.0725	0.776391
6382_17_M	finngen_R	outcome	6382_17_M	Inverse va	57	-0.06791	0.047824	0.155607
6382_17_M	finngen_R	outcome	6382_17_M	Simple mc	57	-0.07912	0.13464	0.559152
6382_17_M	finngen_R	outcome	6382_17_M	Weighted	57	0.006827	0.068464	0.920925
6383_90_T	finngen_R	outcome	6383_90_T	Wald ratic	1	0.651643	0.623399	0.295881
6385_63_V	finngen_R	outcome	6385_63_V	MR Egger	38	-0.02027	0.082617	0.807565
6385_63_V	finngen_R	outcome	6385_63_V	Weighted	38	0.010525	0.078925	0.893913
6385_63_V	finngen_R	outcome	6385_63_V	Inverse va	38	-0.03011	0.062606	0.630512
6385_63_V	finngen_R	outcome	6385_63_V	Simple mc	38	0.243221	0.189838	0.208094
6385_63_V	finngen_R	outcome	6385_63_V	Weighted	38	0.007197	0.075486	0.924559
6392_7_W	finngen_R	outcome	6392_7_W	MR Egger	67	0.11206	0.09834	0.258669
6392_7_W	finngen_R	outcome	6392_7_W	Weighted	67	-0.02149	0.075512	0.775941
6392_7_W	finngen_R	outcome	6392_7_W	Inverse va	67	0.082722	0.049657	0.095743
6392_7_W	finngen_R	outcome	6392_7_W	Simple mc	67	-0.00294	0.139788	0.983278
6392_7_W	finngen_R	outcome	6392_7_W	Weighted	67	-0.03623	0.091954	0.694876
6393_63_H	finngen_R	outcome	6393_63_H	MR Egger	119	-0.00968	0.055084	0.860846
6393_63_H	finngen_R	outcome	6393_63_H	Weighted	119	-0.02817	0.050111	0.573961
6393_63_H	finngen_R	outcome	6393_63_H	Inverse va	119	0.030411	0.030945	0.325732
6393_63_H	finngen_R	outcome	6393_63_H	Simple mc	119	-0.05118	0.090581	0.573164
6393_63_H	finngen_R	outcome	6393_63_H	Weighted	119	-0.02387	0.055627	0.668614
6404_20_C	finngen_R	outcome	6404_20_C	MR Egger	23	-0.04018	0.206333	0.847475
6404_20_C	finngen_R	outcome	6404_20_C	Weighted	23	0.127822	0.130959	0.32904
6404_20_C	finngen_R	outcome	6404_20_C	Inverse va	23	-0.00238	0.10364	0.981673
6404_20_C	finngen_R	outcome	6404_20_C	Simple mc	23	0.226816	0.230368	0.335534
6404_20_C	finngen_R	outcome	6404_20_C	Weighted	23	0.114532	0.143358	0.432876
6408_2_IN	finngen_R	outcome	6408_2_IN	MR Egger	7	0.092514	0.59318	0.882163
6408_2_IN	finngen_R	outcome	6408_2_IN	Weighted	7	0.563857	0.265586	0.033748
6408_2_IN	finngen_R	outcome	6408_2_IN	Inverse va	7	0.660841	0.207502	0.001449
6408_2_IN	finngen_R	outcome	6408_2_IN	Simple mc	7	0.439556	0.439099	0.355454
6408_2_IN	finngen_R	outcome	6408_2_IN	Weighted	7	0.504367	0.321866	0.168155
6415_90_C	finngen_R	outcome	6415_90_C	MR Egger	45	-0.14253	0.089489	0.118544
6415_90_C	finngen_R	outcome	6415_90_C	Weighted	45	-0.02173	0.071467	0.761115
6415_90_C	finngen_R	outcome	6415_90_C	Inverse va	45	-0.05753	0.060177	0.339096
6415_90_C	finngen_R	outcome	6415_90_C	Simple mc	45	-0.10635	0.176853	0.550694
6415_90_C	finngen_R	outcome	6415_90_C	Weighted	45	-0.02536	0.061222	0.680724
6416_8_GI	finngen_R	outcome	6416_8_GI	MR Egger	41	-0.06394	0.099923	0.525988
6416_8_GI	finngen_R	outcome	6416_8_GI	Weighted	41	-0.20379	0.089376	0.022598
6416_8_GI	finngen_R	outcome	6416_8_GI	Inverse va	41	-0.03779	0.063901	0.554222
6416_8_GI	finngen_R	outcome	6416_8_GI	Simple mc	41	0.172183	0.205829	0.407825
6416_8_GI	finngen_R	outcome	6416_8_GI	Weighted	41	-0.16932	0.08655	0.057434
6425_87_M	finngen_R	outcome	6425_87_M	MR Egger	9	0.758225	0.343522	0.063056
6425_87_M	finngen_R	outcome	6425_87_M	Weighted	9	0.545013	0.246777	0.027208
6425_87_M	finngen_R	outcome	6425_87_M	Inverse va	9	0.518519	0.191513	0.006779

6425_87_N	finngen_R	outcome	6425_87_N	Simple mc	9	0.543333	0.37789	0.18843
6425_87_N	finngen_R	outcome	6425_87_N	Weighted	9	0.699919	0.323688	0.062562
6431_68_F	finngen_R	outcome	6431_68_F	MR Egger	77	0.033684	0.059121	0.570548
6431_68_F	finngen_R	outcome	6431_68_F	Weighted	77	-0.05898	0.058617	0.314345
6431_68_F	finngen_R	outcome	6431_68_F	Inverse va	77	-0.02025	0.037294	0.587205
6431_68_F	finngen_R	outcome	6431_68_F	Simple mc	77	0.02005	0.107328	0.852307
6431_68_F	finngen_R	outcome	6431_68_F	Weighted	77	-0.02607	0.047736	0.586506
6433_57_F	finngen_R	outcome	6433_57_F	MR Egger	12	0.150957	0.27831	0.599418
6433_57_F	finngen_R	outcome	6433_57_F	Weighted	12	-0.07083	0.171186	0.679053
6433_57_F	finngen_R	outcome	6433_57_F	Inverse va	12	-0.07429	0.132282	0.574378
6433_57_F	finngen_R	outcome	6433_57_F	Simple mc	12	-0.03519	0.230475	0.881401
6433_57_F	finngen_R	outcome	6433_57_F	Weighted	12	-0.05893	0.17732	0.745887
6434_18_A	finngen_R	outcome	6434_18_A	Inverse va	2	0.176229	0.583145	0.762496
6448_36_S	finngen_R	outcome	6448_36_S	Inverse va	2	-0.32521	0.380282	0.39245
6451_64_A	finngen_R	outcome	6451_64_A	MR Egger	62	0.159846	0.118975	0.184161
6451_64_A	finngen_R	outcome	6451_64_A	Weighted	62	0.111838	0.076864	0.145667
6451_64_A	finngen_R	outcome	6451_64_A	Inverse va	62	0.114885	0.051908	0.02688
6451_64_A	finngen_R	outcome	6451_64_A	Simple mc	62	0.1413	0.16264	0.388366
6451_64_A	finngen_R	outcome	6451_64_A	Weighted	62	0.077443	0.127742	0.546599
6461_54_A	finngen_R	outcome	6461_54_A	MR Egger	12	0.081431	0.269678	0.768873
6461_54_A	finngen_R	outcome	6461_54_A	Weighted	12	0.189463	0.187097	0.31123
6461_54_A	finngen_R	outcome	6461_54_A	Inverse va	12	0.171093	0.140725	0.22406
6461_54_A	finngen_R	outcome	6461_54_A	Simple mc	12	0.285683	0.266356	0.306436
6461_54_A	finngen_R	outcome	6461_54_A	Weighted	12	0.255466	0.21107	0.251515
6462_12_T	finngen_R	outcome	6462_12_T	MR Egger	51	-0.06583	0.098365	0.506513
6462_12_T	finngen_R	outcome	6462_12_T	Weighted	51	0.034975	0.076281	0.646585
6462_12_T	finngen_R	outcome	6462_12_T	Inverse va	51	-0.02475	0.056387	0.660672
6462_12_T	finngen_R	outcome	6462_12_T	Simple mc	51	-0.08137	0.148108	0.585177
6462_12_T	finngen_R	outcome	6462_12_T	Weighted	51	-0.00215	0.081799	0.979184
6467_65_F	finngen_R	outcome	6467_65_F	MR Egger	15	0.196331	0.195933	0.334614
6467_65_F	finngen_R	outcome	6467_65_F	Weighted	15	0.028899	0.155306	0.852384
6467_65_F	finngen_R	outcome	6467_65_F	Inverse va	15	0.045155	0.11173	0.686105
6467_65_F	finngen_R	outcome	6467_65_F	Simple mc	15	-0.09266	0.252999	0.719653
6467_65_F	finngen_R	outcome	6467_65_F	Weighted	15	0.07096	0.146211	0.63495
6470_19_F	finngen_R	outcome	6470_19_F	MR Egger	13	0.038522	0.204747	0.854188
6470_19_F	finngen_R	outcome	6470_19_F	Weighted	13	-0.07624	0.153684	0.619839
6470_19_F	finngen_R	outcome	6470_19_F	Inverse va	13	-0.06836	0.1213	0.573067
6470_19_F	finngen_R	outcome	6470_19_F	Simple mc	13	-0.14344	0.289758	0.629508
6470_19_F	finngen_R	outcome	6470_19_F	Weighted	13	-0.037	0.178458	0.839209
6471_53_C	finngen_R	outcome	6471_53_C	MR Egger	84	-0.00211	0.036713	0.954302
6471_53_C	finngen_R	outcome	6471_53_C	Weighted	84	-0.0442	0.03328	0.184184
6471_53_C	finngen_R	outcome	6471_53_C	Inverse va	84	-0.02226	0.023369	0.340747
6471_53_C	finngen_R	outcome	6471_53_C	Simple mc	84	-0.0222	0.051012	0.664603
6471_53_C	finngen_R	outcome	6471_53_C	Weighted	84	-0.05309	0.035851	0.142401
6472_40_F	finngen_R	outcome	6472_40_F	MR Egger	17	-0.01036	0.154948	0.947579
6472_40_F	finngen_R	outcome	6472_40_F	Weighted	17	0.062424	0.135077	0.643984
6472_40_F	finngen_R	outcome	6472_40_F	Inverse va	17	0.142012	0.102216	0.164731
6472_40_F	finngen_R	outcome	6472_40_F	Simple mc	17	0.161587	0.223374	0.479882
6472_40_F	finngen_R	outcome	6472_40_F	Weighted	17	0.08917	0.136443	0.522695
6485_59_I	finngen_R	outcome	6485_59_I	MR Egger	51	-0.12445	0.071474	0.087932
6485_59_I	finngen_R	outcome	6485_59_I	Weighted	51	-0.03987	0.073801	0.58906
6485_59_I	finngen_R	outcome	6485_59_I	Inverse va	51	0.027236	0.051221	0.594912
6485_59_I	finngen_R	outcome	6485_59_I	Simple mc	51	0.100042	0.152797	0.515639
6485_59_I	finngen_R	outcome	6485_59_I	Weighted	51	-0.0609	0.066647	0.365196
6493_9_CI	finngen_R	outcome	6493_9_CI	Inverse va	2	-0.01912	0.403905	0.962244
6496_60_D	finngen_R	outcome	6496_60_D	MR Egger	74	-0.01249	0.071157	0.861125
6496_60_D	finngen_R	outcome	6496_60_D	Weighted	74	0.026804	0.059062	0.649948
6496_60_D	finngen_R	outcome	6496_60_D	Inverse va	74	0.047697	0.037285	0.200813

6496_60_[	finngen_R	outcome	6496_60_[	Simple mc	74	0.015812	0.099898	0.874676
6496_60_[	finngen_R	outcome	6496_60_[	Weighted	74	0.015812	0.05165	0.760378
6520_87_I	finngen_R	outcome	6520_87_I	MR Egger	26	-0.36757	0.290826	0.21841
6520_87_I	finngen_R	outcome	6520_87_I	Weighted	26	-0.06252	0.13471	0.642569
6520_87_I	finngen_R	outcome	6520_87_I	Inverse va	26	-0.18351	0.113299	0.105297
6520_87_I	finngen_R	outcome	6520_87_I	Simple mc	26	-0.42079	0.237903	0.089138
6520_87_I	finngen_R	outcome	6520_87_I	Weighted	26	-0.16313	0.142079	0.261776
6521_35_I	finngen_R	outcome	6521_35_I	MR Egger	6	-0.34425	1.253208	0.797155
6521_35_I	finngen_R	outcome	6521_35_I	Weighted	6	0.169009	0.398887	0.671784
6521_35_I	finngen_R	outcome	6521_35_I	Inverse va	6	0.22405	0.312076	0.472797
6521_35_I	finngen_R	outcome	6521_35_I	Simple mc	6	-0.02717	0.589162	0.965
6521_35_I	finngen_R	outcome	6521_35_I	Weighted	6	0.105872	0.588123	0.864207
6544_33_I	finngen_R	outcome	6544_33_I	MR Egger	86	0.060068	0.061951	0.335022
6544_33_I	finngen_R	outcome	6544_33_I	Weighted	86	0.06439	0.061632	0.296141
6544_33_I	finngen_R	outcome	6544_33_I	Inverse va	86	0.047205	0.039681	0.234208
6544_33_I	finngen_R	outcome	6544_33_I	Simple mc	86	0.217563	0.124361	0.083824
6544_33_I	finngen_R	outcome	6544_33_I	Weighted	86	0.068147	0.064422	0.293138
6550_4_IC	finngen_R	outcome	6550_4_IC	MR Egger	4	-0.75047	0.893602	0.4894
6550_4_IC	finngen_R	outcome	6550_4_IC	Weighted	4	-0.11918	0.269595	0.658448
6550_4_IC	finngen_R	outcome	6550_4_IC	Inverse va	4	-0.12787	0.239636	0.593625
6550_4_IC	finngen_R	outcome	6550_4_IC	Simple mc	4	0.071907	0.469611	0.888022
6550_4_IC	finngen_R	outcome	6550_4_IC	Weighted	4	-0.37493	0.307138	0.309382
6551_94_S	finngen_R	outcome	6551_94_S	MR Egger	106	-0.05745	0.059404	0.335703
6551_94_S	finngen_R	outcome	6551_94_S	Weighted	106	-0.07768	0.055982	0.165259
6551_94_S	finngen_R	outcome	6551_94_S	Inverse va	106	-0.10401	0.036649	0.00454
6551_94_S	finngen_R	outcome	6551_94_S	Simple mc	106	-0.10917	0.111727	0.330774
6551_94_S	finngen_R	outcome	6551_94_S	Weighted	106	-0.06344	0.055364	0.254466
6556_5_EI	finngen_R	outcome	6556_5_EI	MR Egger	124	0.02862	0.037239	0.443639
6556_5_EI	finngen_R	outcome	6556_5_EI	Weighted	124	0.003801	0.03836	0.921076
6556_5_EI	finngen_R	outcome	6556_5_EI	Inverse va	124	-0.00768	0.025457	0.762818
6556_5_EI	finngen_R	outcome	6556_5_EI	Simple mc	124	-0.06342	0.076366	0.407865
6556_5_EI	finngen_R	outcome	6556_5_EI	Weighted	124	-0.00909	0.034417	0.792123
6558_5_C(	finngen_R	outcome	6558_5_C(	Inverse va	2	0.822073	0.636428	0.196461
6576_1_AI	finngen_R	outcome	6576_1_AI	MR Egger	133	0.001558	0.039352	0.968477
6576_1_AI	finngen_R	outcome	6576_1_AI	Weighted	133	-0.01433	0.039955	0.719871
6576_1_AI	finngen_R	outcome	6576_1_AI	Inverse va	133	-0.02419	0.022984	0.292546
6576_1_AI	finngen_R	outcome	6576_1_AI	Simple mc	133	-0.01311	0.072365	0.856494
6576_1_AI	finngen_R	outcome	6576_1_AI	Weighted	133	-0.02962	0.033828	0.382837
6580_29_F	finngen_R	outcome	6580_29_F	MR Egger	24	-0.30759	0.240173	0.213636
6580_29_F	finngen_R	outcome	6580_29_F	Weighted	24	0.016158	0.162066	0.920582
6580_29_F	finngen_R	outcome	6580_29_F	Inverse va	24	-0.1004	0.116103	0.387176
6580_29_F	finngen_R	outcome	6580_29_F	Simple mc	24	-0.12225	0.329364	0.713914
6580_29_F	finngen_R	outcome	6580_29_F	Weighted	24	0.109692	0.271003	0.689391
6605_17_I(	finngen_R	outcome	6605_17_I(	MR Egger	20	0.168187	0.183148	0.370597
6605_17_I(	finngen_R	outcome	6605_17_I(	Weighted	20	-0.07175	0.129706	0.580155
6605_17_I(	finngen_R	outcome	6605_17_I(	Inverse va	20	-0.03479	0.094024	0.711394
6605_17_I(	finngen_R	outcome	6605_17_I(	Simple mc	20	0.140204	0.198954	0.489543
6605_17_I(	finngen_R	outcome	6605_17_I(	Weighted	20	-0.07985	0.164047	0.632013
6609_22_(	finngen_R	outcome	6609_22_(	Inverse va	2	0.092999	0.358623	0.795386
6617_12_F	finngen_R	outcome	6617_12_F	MR Egger	3	0.423701	0.626991	0.621671
6617_12_F	finngen_R	outcome	6617_12_F	Weighted	3	-0.13633	0.293836	0.642683
6617_12_F	finngen_R	outcome	6617_12_F	Inverse va	3	-0.15102	0.276836	0.585384
6617_12_F	finngen_R	outcome	6617_12_F	Simple mc	3	0.028486	0.445599	0.954843
6617_12_F	finngen_R	outcome	6617_12_F	Weighted	3	-0.10288	0.31383	0.774187
6620_82_L	finngen_R	outcome	6620_82_L	MR Egger	3	-2.2055	2.663519	0.559711
6620_82_L	finngen_R	outcome	6620_82_L	Weighted	3	-0.19049	0.316382	0.547112
6620_82_L	finngen_R	outcome	6620_82_L	Inverse va	3	-0.2824	0.293	0.335132
6620_82_L	finngen_R	outcome	6620_82_L	Simple mc	3	-0.18906	0.388866	0.674899

6620_82_L	finngen_R	outcome	6620_82_L	Weighted	3	-0.19067	0.371059	0.658491
6626_81_(	finngen_R	outcome	6626_81_(	MR Egger	8	0.088604	0.443491	0.848246
6626_81_(	finngen_R	outcome	6626_81_(	Weighted	8	-0.42892	0.190002	0.023981
6626_81_(	finngen_R	outcome	6626_81_(	Inverse va	8	-0.28862	0.138073	0.036588
6626_81_(	finngen_R	outcome	6626_81_(	Simple mc	8	-0.43696	0.299539	0.187993
6626_81_(	finngen_R	outcome	6626_81_(	Weighted	8	-0.50765	0.245078	0.07706
6627_25_F	finngen_R	outcome	6627_25_F	MR Egger	7	-1.33368	0.498915	0.044181
6627_25_F	finngen_R	outcome	6627_25_F	Weighted	7	-0.36217	0.207045	0.080248
6627_25_F	finngen_R	outcome	6627_25_F	Inverse va	7	-0.3264	0.191743	0.088707
6627_25_F	finngen_R	outcome	6627_25_F	Simple mc	7	-0.24125	0.331379	0.494028
6627_25_F	finngen_R	outcome	6627_25_F	Weighted	7	-0.3564	0.209663	0.140061
6629_3_DI	finngen_R	outcome	6629_3_DI	MR Egger	57	-0.01649	0.102714	0.873036
6629_3_DI	finngen_R	outcome	6629_3_DI	Weighted	57	0.055951	0.0814	0.491858
6629_3_DI	finngen_R	outcome	6629_3_DI	Inverse va	57	0.091488	0.051521	0.075774
6629_3_DI	finngen_R	outcome	6629_3_DI	Simple mc	57	0.226392	0.14924	0.134899
6629_3_DI	finngen_R	outcome	6629_3_DI	Weighted	57	0.051601	0.080464	0.523948
6649_51_N	finngen_R	outcome	6649_51_N	MR Egger	41	-0.09313	0.097248	0.344128
6649_51_N	finngen_R	outcome	6649_51_N	Weighted	41	-0.0738	0.083409	0.376298
6649_51_N	finngen_R	outcome	6649_51_N	Inverse va	41	-0.05012	0.056393	0.374136
6649_51_N	finngen_R	outcome	6649_51_N	Simple mc	41	-0.13138	0.137278	0.344309
6649_51_N	finngen_R	outcome	6649_51_N	Weighted	41	-0.09752	0.074829	0.199958
6706_18_A	finngen_R	outcome	6706_18_A	Inverse va	2	0.762711	0.581447	0.189606
6715_63_A	finngen_R	outcome	6715_63_A	MR Egger	19	0.038049	0.201291	0.852313
6715_63_A	finngen_R	outcome	6715_63_A	Weighted	19	0.133413	0.141171	0.344637
6715_63_A	finngen_R	outcome	6715_63_A	Inverse va	19	0.140187	0.1031	0.173919
6715_63_A	finngen_R	outcome	6715_63_A	Simple mc	19	0.020208	0.209696	0.924292
6715_63_A	finngen_R	outcome	6715_63_A	Weighted	19	0.105996	0.162589	0.522685
6984_6_IG	finngen_R	outcome	6984_6_IG	MR Egger	4	-0.14916	0.409993	0.750854
6984_6_IG	finngen_R	outcome	6984_6_IG	Weighted	4	-0.22892	0.215283	0.287632
6984_6_IG	finngen_R	outcome	6984_6_IG	Inverse va	4	-0.24911	0.204836	0.223921
6984_6_IG	finngen_R	outcome	6984_6_IG	Simple mc	4	-0.41262	0.339364	0.310983
6984_6_IG	finngen_R	outcome	6984_6_IG	Weighted	4	-0.19057	0.23591	0.478317
7009_8_CI	finngen_R	outcome	7009_8_CI	MR Egger	5	-1.04324	0.499741	0.128075
7009_8_CI	finngen_R	outcome	7009_8_CI	Weighted	5	-0.33479	0.31028	0.280594
7009_8_CI	finngen_R	outcome	7009_8_CI	Inverse va	5	-0.28462	0.269536	0.290991
7009_8_CI	finngen_R	outcome	7009_8_CI	Simple mc	5	0.183399	0.478459	0.72099
7009_8_CI	finngen_R	outcome	7009_8_CI	Weighted	5	-0.4791	0.47339	0.368756
7015_8_LII	finngen_R	outcome	7015_8_LII	MR Egger	205	0.013727	0.028039	0.624972
7015_8_LII	finngen_R	outcome	7015_8_LII	Weighted	205	0.019042	0.030382	0.530814
7015_8_LII	finngen_R	outcome	7015_8_LII	Inverse va	205	0.003399	0.017791	0.848489
7015_8_LII	finngen_R	outcome	7015_8_LII	Simple mc	205	-0.02802	0.055825	0.616269
7015_8_LII	finngen_R	outcome	7015_8_LII	Weighted	205	0.029665	0.026116	0.257335
7019_13_S	finngen_R	outcome	7019_13_S	Wald ratic	1	0.127514	0.517206	0.805261
7049_2_AI	finngen_R	outcome	7049_2_AI	MR Egger	110	0.084821	0.050891	0.098467
7049_2_AI	finngen_R	outcome	7049_2_AI	Weighted	110	0.006378	0.05453	0.906891
7049_2_AI	finngen_R	outcome	7049_2_AI	Inverse va	110	0.024434	0.032425	0.451121
7049_2_AI	finngen_R	outcome	7049_2_AI	Simple mc	110	-0.11328	0.103094	0.274283
7049_2_AI	finngen_R	outcome	7049_2_AI	Weighted	110	0.045282	0.058401	0.439803
7050_5_NI	finngen_R	outcome	7050_5_NI	MR Egger	9	0.521099	0.619732	0.428219
7050_5_NI	finngen_R	outcome	7050_5_NI	Weighted	9	0.043178	0.247128	0.861301
7050_5_NI	finngen_R	outcome	7050_5_NI	Inverse va	9	0.040722	0.19158	0.831672
7050_5_NI	finngen_R	outcome	7050_5_NI	Simple mc	9	0.295957	0.400916	0.481483
7050_5_NI	finngen_R	outcome	7050_5_NI	Weighted	9	0.06698	0.265961	0.807514
7059_14_L	finngen_R	outcome	7059_14_L	MR Egger	239	-0.03937	0.030611	0.199674
7059_14_L	finngen_R	outcome	7059_14_L	Weighted	239	-0.03643	0.033271	0.273561
7059_14_L	finngen_R	outcome	7059_14_L	Inverse va	239	-0.01565	0.018925	0.408182
7059_14_L	finngen_R	outcome	7059_14_L	Simple mc	239	-0.06259	0.069285	0.367217
7059_14_L	finngen_R	outcome	7059_14_L	Weighted	239	-0.03836	0.036736	0.297463

7083_74_I	finngen_R	outcome	7083_74_I	MR Egger	27	-0.28129	0.151441	0.075068
7083_74_I	finngen_R	outcome	7083_74_I	Weighted	27	-0.27464	0.107027	0.010285
7083_74_I	finngen_R	outcome	7083_74_I	Inverse va	27	-0.27514	0.074956	0.000242
7083_74_I	finngen_R	outcome	7083_74_I	Simple mc	27	-0.50049	0.191438	0.014675
7083_74_I	finngen_R	outcome	7083_74_I	Weighted	27	-0.30875	0.112603	0.010905
7110_2_DI	finngen_R	outcome	7110_2_DI	MR Egger	4	2.10555	1.745012	0.350938
7110_2_DI	finngen_R	outcome	7110_2_DI	Weighted	4	-0.12591	0.457729	0.783261
7110_2_DI	finngen_R	outcome	7110_2_DI	Inverse va	4	0.074689	0.391766	0.848802
7110_2_DI	finngen_R	outcome	7110_2_DI	Simple mc	4	-0.15011	0.552546	0.803503
7110_2_DI	finngen_R	outcome	7110_2_DI	Weighted	4	-0.15011	0.481306	0.775555
7128_9_V\	finngen_R	outcome	7128_9_V\	MR Egger	31	-0.13738	0.15123	0.37113
7128_9_V\	finngen_R	outcome	7128_9_V\	Weighted	31	-0.0896	0.124955	0.473357
7128_9_V\	finngen_R	outcome	7128_9_V\	Inverse va	31	-0.14812	0.08381	0.077175
7128_9_V\	finngen_R	outcome	7128_9_V\	Simple mc	31	-0.05369	0.22143	0.810061
7128_9_V\	finngen_R	outcome	7128_9_V\	Weighted	31	-0.04056	0.170102	0.81317
7140_1_CE	finngen_R	outcome	7140_1_CE	MR Egger	6	-0.48555	0.600322	0.463983
7140_1_CE	finngen_R	outcome	7140_1_CE	Weighted	6	-0.00999	0.383509	0.979214
7140_1_CE	finngen_R	outcome	7140_1_CE	Inverse va	6	-0.04828	0.300622	0.872419
7140_1_CE	finngen_R	outcome	7140_1_CE	Simple mc	6	0.159425	0.526985	0.774435
7140_1_CE	finngen_R	outcome	7140_1_CE	Weighted	6	0.197781	0.468714	0.690579
7145_1_ITI	finngen_R	outcome	7145_1_ITI	MR Egger	85	-0.02306	0.055815	0.680615
7145_1_ITI	finngen_R	outcome	7145_1_ITI	Weighted	85	0.022452	0.049866	0.652536
7145_1_ITI	finngen_R	outcome	7145_1_ITI	Inverse va	85	0.014183	0.033684	0.673724
7145_1_ITI	finngen_R	outcome	7145_1_ITI	Simple mc	85	-0.01456	0.08514	0.864594
7145_1_ITI	finngen_R	outcome	7145_1_ITI	Weighted	85	0.008333	0.050247	0.868681
7161_25_H	finngen_R	outcome	7161_25_H	MR Egger	97	-0.01655	0.055289	0.765334
7161_25_H	finngen_R	outcome	7161_25_H	Weighted	97	-0.04631	0.050956	0.363451
7161_25_H	finngen_R	outcome	7161_25_H	Inverse va	97	-0.03521	0.033342	0.290995
7161_25_H	finngen_R	outcome	7161_25_H	Simple mc	97	-0.03616	0.086905	0.678244
7161_25_H	finngen_R	outcome	7161_25_H	Weighted	97	-0.05055	0.043366	0.246611
7185_29_C	finngen_R	outcome	7185_29_C	MR Egger	4	0.830149	1.229675	0.569203
7185_29_C	finngen_R	outcome	7185_29_C	Weighted	4	-0.16443	0.323734	0.611516
7185_29_C	finngen_R	outcome	7185_29_C	Inverse va	4	-0.18779	0.277837	0.499114
7185_29_C	finngen_R	outcome	7185_29_C	Simple mc	4	-0.22464	0.442955	0.646993
7185_29_C	finngen_R	outcome	7185_29_C	Weighted	4	-0.15813	0.341672	0.675012
7206_20_F	finngen_R	outcome	7206_20_F	MR Egger	3	-0.93501	0.797495	0.449576
7206_20_F	finngen_R	outcome	7206_20_F	Weighted	3	-0.5246	0.297458	0.077799
7206_20_F	finngen_R	outcome	7206_20_F	Inverse va	3	-0.52273	0.282567	0.064326
7206_20_F	finngen_R	outcome	7206_20_F	Simple mc	3	-0.35914	0.395487	0.459678
7206_20_F	finngen_R	outcome	7206_20_F	Weighted	3	-0.54307	0.374861	0.284418
7211_2_RI	finngen_R	outcome	7211_2_RI	MR Egger	25	-0.17099	0.164862	0.310444
7211_2_RI	finngen_R	outcome	7211_2_RI	Weighted	25	0.085321	0.14066	0.544132
7211_2_RI	finngen_R	outcome	7211_2_RI	Inverse va	25	0.131743	0.08968	0.14182
7211_2_RI	finngen_R	outcome	7211_2_RI	Simple mc	25	-0.05139	0.259844	0.844891
7211_2_RI	finngen_R	outcome	7211_2_RI	Weighted	25	-0.00337	0.152065	0.982504
7218_87_A	finngen_R	outcome	7218_87_A	MR Egger	38	-0.01903	0.159779	0.905859
7218_87_A	finngen_R	outcome	7218_87_A	Weighted	38	-0.03562	0.09668	0.71254
7218_87_A	finngen_R	outcome	7218_87_A	Inverse va	38	0.009322	0.067655	0.890405
7218_87_A	finngen_R	outcome	7218_87_A	Simple mc	38	0.058986	0.194109	0.76292
7218_87_A	finngen_R	outcome	7218_87_A	Weighted	38	0.025285	0.113975	0.825653
7227_75_C	finngen_R	outcome	7227_75_C	MR Egger	22	0.058304	0.116526	0.62229
7227_75_C	finngen_R	outcome	7227_75_C	Weighted	22	0.077363	0.100692	0.442301
7227_75_C	finngen_R	outcome	7227_75_C	Inverse va	22	0.08591	0.072546	0.236323
7227_75_C	finngen_R	outcome	7227_75_C	Simple mc	22	0.157883	0.160665	0.336949
7227_75_C	finngen_R	outcome	7227_75_C	Weighted	22	0.092168	0.093774	0.336858
7266_4_SE	finngen_R	outcome	7266_4_SE	MR Egger	24	0.205257	0.208882	0.336466
7266_4_SE	finngen_R	outcome	7266_4_SE	Weighted	24	-0.09383	0.124926	0.45262
7266_4_SE	finngen_R	outcome	7266_4_SE	Inverse va	24	-0.12286	0.089578	0.170214

7266_4_SE	finngen_R	outcome	7266_4_SE	Simple mc	24	-0.02338	0.194172	0.90519
7266_4_SE	finngen_R	outcome	7266_4_SE	Weighted	24	-0.0072	0.156895	0.963793
7655_11_N	finngen_R	outcome	7655_11_N	MR Egger	20	0.105295	0.172737	0.549765
7655_11_N	finngen_R	outcome	7655_11_N	Weighted	20	0.060185	0.121351	0.61992
7655_11_N	finngen_R	outcome	7655_11_N	Inverse va	20	0.070483	0.088294	0.424712
7655_11_N	finngen_R	outcome	7655_11_N	Simple mc	20	-0.18214	0.205038	0.38547
7655_11_N	finngen_R	outcome	7655_11_N	Weighted	20	0.017359	0.130375	0.895476
7779_86_C	finngen_R	outcome	7779_86_C	MR Egger	47	0.088769	0.157023	0.57466
7779_86_C	finngen_R	outcome	7779_86_C	Weighted	47	-0.01909	0.098227	0.845907
7779_86_C	finngen_R	outcome	7779_86_C	Inverse va	47	-0.11598	0.066719	0.082144
7779_86_C	finngen_R	outcome	7779_86_C	Simple mc	47	0.079037	0.162536	0.629081
7779_86_C	finngen_R	outcome	7779_86_C	Weighted	47	0.031187	0.115161	0.78775
7784_1_KN	finngen_R	outcome	7784_1_KN	MR Egger	28	-0.03968	0.118364	0.740145
7784_1_KN	finngen_R	outcome	7784_1_KN	Weighted	28	-0.03704	0.114981	0.747333
7784_1_KN	finngen_R	outcome	7784_1_KN	Inverse va	28	-0.01843	0.076245	0.808991
7784_1_KN	finngen_R	outcome	7784_1_KN	Simple mc	28	0.017137	0.185914	0.927237
7784_1_KN	finngen_R	outcome	7784_1_KN	Weighted	28	-0.02512	0.103852	0.810689
7787_25_L	finngen_R	outcome	7787_25_L	MR Egger	30	0.038953	0.115877	0.73926
7787_25_L	finngen_R	outcome	7787_25_L	Weighted	30	0.001879	0.091768	0.983664
7787_25_L	finngen_R	outcome	7787_25_L	Inverse va	30	-0.01665	0.067329	0.80466
7787_25_L	finngen_R	outcome	7787_25_L	Simple mc	30	0.174982	0.169109	0.309347
7787_25_L	finngen_R	outcome	7787_25_L	Weighted	30	0.04462	0.088602	0.61835
7810_20_C	finngen_R	outcome	7810_20_C	MR Egger	7	-0.38819	0.247487	0.177545
7810_20_C	finngen_R	outcome	7810_20_C	Weighted	7	0.038811	0.152263	0.798802
7810_20_C	finngen_R	outcome	7810_20_C	Inverse va	7	0.299834	0.17917	0.094237
7810_20_C	finngen_R	outcome	7810_20_C	Simple mc	7	0.801106	0.339611	0.056369
7810_20_C	finngen_R	outcome	7810_20_C	Weighted	7	0.073943	0.158557	0.657417
7841_84_E	finngen_R	outcome	7841_84_E	MR Egger	26	0.08978	0.170164	0.602616
7841_84_E	finngen_R	outcome	7841_84_E	Weighted	26	0.332884	0.138202	0.01601
7841_84_E	finngen_R	outcome	7841_84_E	Inverse va	26	0.210599	0.093749	0.024678
7841_84_E	finngen_R	outcome	7841_84_E	Simple mc	26	0.323701	0.239637	0.18886
7841_84_E	finngen_R	outcome	7841_84_E	Weighted	26	0.291895	0.149609	0.062344
7849_3_QI	finngen_R	outcome	7849_3_QI	MR Egger	32	-0.10621	0.140128	0.454408
7849_3_QI	finngen_R	outcome	7849_3_QI	Weighted	32	-0.0888	0.103041	0.388806
7849_3_QI	finngen_R	outcome	7849_3_QI	Inverse va	32	-0.0434	0.07147	0.543677
7849_3_QI	finngen_R	outcome	7849_3_QI	Simple mc	32	-0.10046	0.159669	0.533854
7849_3_QI	finngen_R	outcome	7849_3_QI	Weighted	32	-0.10965	0.103806	0.298994
7861_9_RC	finngen_R	outcome	7861_9_RC	MR Egger	20	-0.07907	0.437191	0.858491
7861_9_RC	finngen_R	outcome	7861_9_RC	Weighted	20	-0.35933	0.16655	0.030969
7861_9_RC	finngen_R	outcome	7861_9_RC	Inverse va	20	-0.2585	0.145121	0.074868
7861_9_RC	finngen_R	outcome	7861_9_RC	Simple mc	20	0.267131	0.368586	0.477439
7861_9_RC	finngen_R	outcome	7861_9_RC	Weighted	20	-0.25321	0.212603	0.248325
7875_86_F	finngen_R	outcome	7875_86_F	MR Egger	11	0.379915	0.430401	0.40036
7875_86_F	finngen_R	outcome	7875_86_F	Weighted	11	0.557578	0.233442	0.016917
7875_86_F	finngen_R	outcome	7875_86_F	Inverse va	11	0.20962	0.19113	0.272755
7875_86_F	finngen_R	outcome	7875_86_F	Simple mc	11	0.458102	0.448863	0.331508
7875_86_F	finngen_R	outcome	7875_86_F	Weighted	11	0.561157	0.275621	0.069111
7891_45_L	finngen_R	outcome	7891_45_L	MR Egger	23	-0.31045	0.175868	0.092066
7891_45_L	finngen_R	outcome	7891_45_L	Weighted	23	-0.18651	0.123688	0.131583
7891_45_L	finngen_R	outcome	7891_45_L	Inverse va	23	0.015408	0.088241	0.861381
7891_45_L	finngen_R	outcome	7891_45_L	Simple mc	23	0.170304	0.271015	0.536215
7891_45_L	finngen_R	outcome	7891_45_L	Weighted	23	-0.19113	0.117272	0.117374
7905_30_H	finngen_R	outcome	7905_30_H	MR Egger	77	-0.00087	0.050905	0.986457
7905_30_H	finngen_R	outcome	7905_30_H	Weighted	77	0.007426	0.041752	0.858835
7905_30_H	finngen_R	outcome	7905_30_H	Inverse va	77	-0.00571	0.027437	0.835166
7905_30_H	finngen_R	outcome	7905_30_H	Simple mc	77	-0.03813	0.070011	0.587583
7905_30_H	finngen_R	outcome	7905_30_H	Weighted	77	0.021954	0.045058	0.627492
7916_10_S	finngen_R	outcome	7916_10_S	MR Egger	55	-0.059	0.043431	0.180076

7916_10_S	finngen_R	outcome	7916_10_S	Weighted	55	-0.07182	0.044596	0.107316
7916_10_S	finngen_R	outcome	7916_10_S	Inverse va	55	-0.04107	0.0331	0.214733
7916_10_S	finngen_R	outcome	7916_10_S	Simple mc	55	-0.04447	0.087882	0.614903
7916_10_S	finngen_R	outcome	7916_10_S	Weighted	55	-0.07723	0.039807	0.057605
7918_114_	finngen_R	outcome	7918_114_	MR Egger	70	0.004621	0.071599	0.948727
7918_114_	finngen_R	outcome	7918_114_	Weighted	70	0.026882	0.054121	0.619391
7918_114_	finngen_R	outcome	7918_114_	Inverse va	70	0.033708	0.035874	0.347416
7918_114_	finngen_R	outcome	7918_114_	Simple mc	70	0.075266	0.096554	0.438336
7918_114_	finngen_R	outcome	7918_114_	Weighted	70	0.035397	0.074115	0.634449
7921_65_F	finngen_R	outcome	7921_65_F	MR Egger	27	0.076362	0.235725	0.748672
7921_65_F	finngen_R	outcome	7921_65_F	Weighted	27	-0.0495	0.141097	0.725711
7921_65_F	finngen_R	outcome	7921_65_F	Inverse va	27	0.111125	0.093338	0.233823
7921_65_F	finngen_R	outcome	7921_65_F	Simple mc	27	0.373588	0.286255	0.203295
7921_65_F	finngen_R	outcome	7921_65_F	Weighted	27	-0.06109	0.192576	0.753598
7926_13_S	finngen_R	outcome	7926_13_S	MR Egger	43	0.138895	0.159011	0.387484
7926_13_S	finngen_R	outcome	7926_13_S	Weighted	43	0.191225	0.109778	0.081522
7926_13_S	finngen_R	outcome	7926_13_S	Inverse va	43	0.211322	0.073173	0.003877
7926_13_S	finngen_R	outcome	7926_13_S	Simple mc	43	0.261772	0.207412	0.21388
7926_13_S	finngen_R	outcome	7926_13_S	Weighted	43	0.194193	0.10911	0.082347
7955_195_	finngen_R	outcome	7955_195_	MR Egger	65	-0.00567	0.07325	0.938588
7955_195_	finngen_R	outcome	7955_195_	Weighted	65	-0.00165	0.064818	0.979675
7955_195_	finngen_R	outcome	7955_195_	Inverse va	65	-0.01346	0.040766	0.741321
7955_195_	finngen_R	outcome	7955_195_	Simple mc	65	-0.03597	0.11558	0.756659
7955_195_	finngen_R	outcome	7955_195_	Weighted	65	0.004144	0.053289	0.938256
7957_2_SC	finngen_R	outcome	7957_2_SC	MR Egger	55	-0.04562	0.097572	0.642015
7957_2_SC	finngen_R	outcome	7957_2_SC	Weighted	55	-0.00595	0.087231	0.945663
7957_2_SC	finngen_R	outcome	7957_2_SC	Inverse va	55	0.022896	0.055372	0.679241
7957_2_SC	finngen_R	outcome	7957_2_SC	Simple mc	55	0.085606	0.15765	0.589356
7957_2_SC	finngen_R	outcome	7957_2_SC	Weighted	55	0.007392	0.091455	0.935882
7968_15_C	finngen_R	outcome	7968_15_C	MR Egger	41	0.209584	0.114456	0.074729
7968_15_C	finngen_R	outcome	7968_15_C	Weighted	41	0.149735	0.091015	0.099935
7968_15_C	finngen_R	outcome	7968_15_C	Inverse va	41	0.124325	0.06496	0.055638
7968_15_C	finngen_R	outcome	7968_15_C	Simple mc	41	0.237369	0.149415	0.120012
7968_15_C	finngen_R	outcome	7968_15_C	Weighted	41	0.153098	0.082811	0.071891
7999_23_E	finngen_R	outcome	7999_23_E	MR Egger	9	-0.11691	0.316011	0.722348
7999_23_E	finngen_R	outcome	7999_23_E	Weighted	9	0.011526	0.224518	0.959058
7999_23_E	finngen_R	outcome	7999_23_E	Inverse va	9	0.018147	0.171234	0.915602
7999_23_E	finngen_R	outcome	7999_23_E	Simple mc	9	0.212727	0.327959	0.534744
7999_23_E	finngen_R	outcome	7999_23_E	Weighted	9	-0.02136	0.243156	0.932159
8007_19_C	finngen_R	outcome	8007_19_C	MR Egger	87	-0.04649	0.056059	0.409299
8007_19_C	finngen_R	outcome	8007_19_C	Weighted	87	-0.13251	0.052117	0.011004
8007_19_C	finngen_R	outcome	8007_19_C	Inverse va	87	-0.16702	0.032993	4.14E-07
8007_19_C	finngen_R	outcome	8007_19_C	Simple mc	87	-0.18888	0.085201	0.029272
8007_19_C	finngen_R	outcome	8007_19_C	Weighted	87	-0.14757	0.044153	0.001232
8028_22_S	finngen_R	outcome	8028_22_S	MR Egger	6	-0.23554	0.409794	0.596227
8028_22_S	finngen_R	outcome	8028_22_S	Weighted	6	-0.15592	0.206759	0.450773
8028_22_S	finngen_R	outcome	8028_22_S	Inverse va	6	-0.24803	0.161052	0.123541
8028_22_S	finngen_R	outcome	8028_22_S	Simple mc	6	-0.75396	0.330016	0.071125
8028_22_S	finngen_R	outcome	8028_22_S	Weighted	6	-0.07823	0.233565	0.751268
8043_153_	finngen_R	outcome	8043_153_	MR Egger	14	0.198559	0.239089	0.422483
8043_153_	finngen_R	outcome	8043_153_	Weighted	14	0.203218	0.168868	0.228815
8043_153_	finngen_R	outcome	8043_153_	Inverse va	14	0.089649	0.124883	0.472842
8043_153_	finngen_R	outcome	8043_153_	Simple mc	14	-0.18853	0.261154	0.483136
8043_153_	finngen_R	outcome	8043_153_	Weighted	14	0.149108	0.167637	0.389915
8065_245_	finngen_R	outcome	8065_245_	MR Egger	5	-0.19635	0.555435	0.747093
8065_245_	finngen_R	outcome	8065_245_	Weighted	5	-0.14097	0.239854	0.556703
8065_245_	finngen_R	outcome	8065_245_	Inverse va	5	-0.17409	0.208739	0.404272
8065_245_	finngen_R	outcome	8065_245_	Simple mc	5	-0.14522	0.335193	0.687181

8065_245_	finngen_R	outcome	8065_245_	Weighted	5	-0.13834	0.27563	0.642111
8080_24_N	finngen_R	outcome	8080_24_N	Wald ratic	1	-0.43959	0.555131	0.428439
8097_77_L	finngen_R	outcome	8097_77_L	MR Egger	133	0.066662	0.047942	0.166741
8097_77_L	finngen_R	outcome	8097_77_L	Weighted	133	0.100178	0.044564	0.024579
8097_77_L	finngen_R	outcome	8097_77_L	Inverse va	133	0.093609	0.027698	0.000726
8097_77_L	finngen_R	outcome	8097_77_L	Simple mc	133	-0.00945	0.081047	0.907384
8097_77_L	finngen_R	outcome	8097_77_L	Weighted	133	0.07665	0.044756	0.089132
8099_42_S	finngen_R	outcome	8099_42_S	MR Egger	31	-0.23609	0.124778	0.068508
8099_42_S	finngen_R	outcome	8099_42_S	Weighted	31	-0.17259	0.09866	0.080239
8099_42_S	finngen_R	outcome	8099_42_S	Inverse va	31	-0.15187	0.067161	0.023741
8099_42_S	finngen_R	outcome	8099_42_S	Simple mc	31	-0.19667	0.163132	0.237391
8099_42_S	finngen_R	outcome	8099_42_S	Weighted	31	-0.1655	0.095775	0.094276
8100_15_A	finngen_R	outcome	8100_15_A	MR Egger	5	-0.42433	0.482733	0.444112
8100_15_A	finngen_R	outcome	8100_15_A	Weighted	5	-0.13345	0.28065	0.634435
8100_15_A	finngen_R	outcome	8100_15_A	Inverse va	5	-0.19594	0.238256	0.41086
8100_15_A	finngen_R	outcome	8100_15_A	Simple mc	5	-0.20982	0.436496	0.655852
8100_15_A	finngen_R	outcome	8100_15_A	Weighted	5	-0.13759	0.287747	0.657497
8221_19_N	finngen_R	outcome	8221_19_N	Wald ratic	1	-1.38618	0.764295	0.069729
8225_86_E	finngen_R	outcome	8225_86_E	MR Egger	16	-0.17963	0.347285	0.61306
8225_86_E	finngen_R	outcome	8225_86_E	Weighted	16	0.197254	0.194362	0.310163
8225_86_E	finngen_R	outcome	8225_86_E	Inverse va	16	0.106739	0.138835	0.441999
8225_86_E	finngen_R	outcome	8225_86_E	Simple mc	16	0.223116	0.322738	0.499923
8225_86_E	finngen_R	outcome	8225_86_E	Weighted	16	0.291571	0.267769	0.293389
8231_122_	finngen_R	outcome	8231_122_	Wald ratic	1	-1.01618	0.69143	0.141648
8233_2_ITI	finngen_R	outcome	8233_2_ITI	MR Egger	94	0.10472	0.088208	0.238208
8233_2_ITI	finngen_R	outcome	8233_2_ITI	Weighted	94	0.026518	0.065534	0.685741
8233_2_ITI	finngen_R	outcome	8233_2_ITI	Inverse va	94	0.039834	0.043747	0.362535
8233_2_ITI	finngen_R	outcome	8233_2_ITI	Simple mc	94	-0.0629	0.13271	0.63663
8233_2_ITI	finngen_R	outcome	8233_2_ITI	Weighted	94	0.051731	0.075256	0.493541
8235_48_C	finngen_R	outcome	8235_48_C	Wald ratic	1	0.684974	0.404525	0.090402
8243_55_S	finngen_R	outcome	8243_55_S	MR Egger	4	1.099564	0.733958	0.272821
8243_55_S	finngen_R	outcome	8243_55_S	Weighted	4	0.265436	0.29146	0.362448
8243_55_S	finngen_R	outcome	8243_55_S	Inverse va	4	0.095951	0.267073	0.719394
8243_55_S	finngen_R	outcome	8243_55_S	Simple mc	4	0.183162	0.521751	0.748755
8243_55_S	finngen_R	outcome	8243_55_S	Weighted	4	0.288897	0.307776	0.417152
8250_2_PT	finngen_R	outcome	8250_2_PT	MR Egger	8	0.017132	0.312738	0.958093
8250_2_PT	finngen_R	outcome	8250_2_PT	Weighted	8	-0.0594	0.226839	0.793418
8250_2_PT	finngen_R	outcome	8250_2_PT	Inverse va	8	-0.13876	0.180926	0.443101
8250_2_PT	finngen_R	outcome	8250_2_PT	Simple mc	8	-0.07681	0.31936	0.816814
8250_2_PT	finngen_R	outcome	8250_2_PT	Weighted	8	-0.04086	0.279445	0.887864
8252_2_NO	finngen_R	outcome	8252_2_NO	MR Egger	10	0.210597	0.351174	0.565305
8252_2_NO	finngen_R	outcome	8252_2_NO	Weighted	10	-0.11145	0.220083	0.612576
8252_2_NO	finngen_R	outcome	8252_2_NO	Inverse va	10	-0.04867	0.16854	0.772766
8252_2_NO	finngen_R	outcome	8252_2_NO	Simple mc	10	-0.02508	0.282542	0.931212
8252_2_NO	finngen_R	outcome	8252_2_NO	Weighted	10	-0.06001	0.225518	0.796147
8288_27_A	finngen_R	outcome	8288_27_A	MR Egger	18	0.11984	0.106503	0.27709
8288_27_A	finngen_R	outcome	8288_27_A	Weighted	18	0.095221	0.100066	0.341312
8288_27_A	finngen_R	outcome	8288_27_A	Inverse va	18	0.112429	0.080633	0.163219
8288_27_A	finngen_R	outcome	8288_27_A	Simple mc	18	0.115234	0.196673	0.565631
8288_27_A	finngen_R	outcome	8288_27_A	Weighted	18	0.088922	0.105911	0.412793
8289_8_GI	finngen_R	outcome	8289_8_GI	MR Egger	26	-0.22868	0.167016	0.183617
8289_8_GI	finngen_R	outcome	8289_8_GI	Weighted	26	-0.03662	0.127552	0.774055
8289_8_GI	finngen_R	outcome	8289_8_GI	Inverse va	26	-0.05758	0.089377	0.519431
8289_8_GI	finngen_R	outcome	8289_8_GI	Simple mc	26	-0.11259	0.207281	0.591806
8289_8_GI	finngen_R	outcome	8289_8_GI	Weighted	26	-0.06235	0.121433	0.612172
8299_66_L	finngen_R	outcome	8299_66_L	MR Egger	58	0.130025	0.089066	0.149913
8299_66_L	finngen_R	outcome	8299_66_L	Weighted	58	0.069148	0.072361	0.339275
8299_66_L	finngen_R	outcome	8299_66_L	Inverse va	58	0.123793	0.050125	0.013524

8299_66_L	finngen_R	outcome	8299_66_L	Simple mc	58	0.08388	0.134659	0.535829
8299_66_L	finngen_R	outcome	8299_66_L	Weighted	58	0.117085	0.076369	0.130772
8304_50_T	finngen_R	outcome	8304_50_T	MR Egger	27	-0.14483	0.276706	0.605296
8304_50_T	finngen_R	outcome	8304_50_T	Weighted	27	-0.19391	0.155362	0.211983
8304_50_T	finngen_R	outcome	8304_50_T	Inverse va	27	-0.15236	0.10473	0.145727
8304_50_T	finngen_R	outcome	8304_50_T	Simple mc	27	-0.13541	0.312497	0.668349
8304_50_T	finngen_R	outcome	8304_50_T	Weighted	27	-0.21172	0.197917	0.294569
8309_12_H	finngen_R	outcome	8309_12_H	MR Egger	4	0.904919	0.405263	0.155186
8309_12_H	finngen_R	outcome	8309_12_H	Weighted	4	0.726469	0.283685	0.010442
8309_12_H	finngen_R	outcome	8309_12_H	Inverse va	4	0.701168	0.259373	0.006865
8309_12_H	finngen_R	outcome	8309_12_H	Simple mc	4	0.603814	0.4402	0.263746
8309_12_H	finngen_R	outcome	8309_12_H	Weighted	4	0.737014	0.28835	0.083507
8323_163_	finngen_R	outcome	8323_163_	Inverse va	2	0.475822	0.407809	0.243301
8325_37_A	finngen_R	outcome	8325_37_A	MR Egger	3	-0.23569	1.090806	0.864529
8325_37_A	finngen_R	outcome	8325_37_A	Weighted	3	-0.04419	0.355268	0.90101
8325_37_A	finngen_R	outcome	8325_37_A	Inverse va	3	-0.036	0.31012	0.907591
8325_37_A	finngen_R	outcome	8325_37_A	Simple mc	3	0.011535	0.397652	0.979493
8325_37_A	finngen_R	outcome	8325_37_A	Weighted	3	-0.05827	0.422012	0.902825
8327_26_D	finngen_R	outcome	8327_26_D	MR Egger	7	-0.32291	0.556279	0.586763
8327_26_D	finngen_R	outcome	8327_26_D	Weighted	7	0.084722	0.18819	0.652568
8327_26_D	finngen_R	outcome	8327_26_D	Inverse va	7	0.030239	0.148557	0.838706
8327_26_D	finngen_R	outcome	8327_26_D	Simple mc	7	0.060133	0.215954	0.790012
8327_26_D	finngen_R	outcome	8327_26_D	Weighted	7	0.097535	0.176341	0.600175
8345_27_C	finngen_R	outcome	8345_27_C	MR Egger	51	0.105382	0.077793	0.181744
8345_27_C	finngen_R	outcome	8345_27_C	Weighted	51	0.127828	0.066606	0.054962
8345_27_C	finngen_R	outcome	8345_27_C	Inverse va	51	0.030247	0.049525	0.541377
8345_27_C	finngen_R	outcome	8345_27_C	Simple mc	51	0.156987	0.128219	0.226551
8345_27_C	finngen_R	outcome	8345_27_C	Weighted	51	0.129983	0.060606	0.036859
8346_9_DI	finngen_R	outcome	8346_9_DI	MR Egger	63	0.023503	0.068471	0.732588
8346_9_DI	finngen_R	outcome	8346_9_DI	Weighted	63	0.047191	0.068451	0.490564
8346_9_DI	finngen_R	outcome	8346_9_DI	Inverse va	63	0.059942	0.043573	0.168928
8346_9_DI	finngen_R	outcome	8346_9_DI	Simple mc	63	0.144714	0.120227	0.23329
8346_9_DI	finngen_R	outcome	8346_9_DI	Weighted	63	0.054203	0.060113	0.370712
8356_88_C	finngen_R	outcome	8356_88_C	MR Egger	41	-0.1008	0.115837	0.389529
8356_88_C	finngen_R	outcome	8356_88_C	Weighted	41	-0.05944	0.095673	0.534414
8356_88_C	finngen_R	outcome	8356_88_C	Inverse va	41	-0.06718	0.064035	0.294155
8356_88_C	finngen_R	outcome	8356_88_C	Simple mc	41	-0.12432	0.186882	0.50972
8356_88_C	finngen_R	outcome	8356_88_C	Weighted	41	-0.08602	0.087152	0.329558
8360_169_	finngen_R	outcome	8360_169_	MR Egger	23	0.162579	0.141669	0.264043
8360_169_	finngen_R	outcome	8360_169_	Weighted	23	0.154043	0.100361	0.12481
8360_169_	finngen_R	outcome	8360_169_	Inverse va	23	0.094913	0.075054	0.206016
8360_169_	finngen_R	outcome	8360_169_	Simple mc	23	0.099927	0.153007	0.520466
8360_169_	finngen_R	outcome	8360_169_	Weighted	23	0.148713	0.096091	0.135979
8368_102_	finngen_R	outcome	8368_102_	MR Egger	8	0.288004	0.206162	0.211902
8368_102_	finngen_R	outcome	8368_102_	Weighted	8	0.237307	0.155295	0.126487
8368_102_	finngen_R	outcome	8368_102_	Inverse va	8	0.150194	0.121542	0.216557
8368_102_	finngen_R	outcome	8368_102_	Simple mc	8	0.340787	0.26045	0.232041
8368_102_	finngen_R	outcome	8368_102_	Weighted	8	0.210243	0.213068	0.356639
8376_25_L	finngen_R	outcome	8376_25_L	MR Egger	25	-0.24844	0.124093	0.057207
8376_25_L	finngen_R	outcome	8376_25_L	Weighted	25	-0.13996	0.104951	0.18235
8376_25_L	finngen_R	outcome	8376_25_L	Inverse va	25	-0.16611	0.079132	0.035808
8376_25_L	finngen_R	outcome	8376_25_L	Simple mc	25	-0.31095	0.215703	0.162353
8376_25_L	finngen_R	outcome	8376_25_L	Weighted	25	-0.19532	0.108865	0.085394
8394_56_F	finngen_R	outcome	8394_56_F	MR Egger	4	0.122107	0.45008	0.811596
8394_56_F	finngen_R	outcome	8394_56_F	Weighted	4	-0.40566	0.215517	0.059801
8394_56_F	finngen_R	outcome	8394_56_F	Inverse va	4	-0.41147	0.198433	0.038118
8394_56_F	finngen_R	outcome	8394_56_F	Simple mc	4	-0.74592	0.438935	0.187809
8394_56_F	finngen_R	outcome	8394_56_F	Weighted	4	-0.23387	0.237195	0.396847

8397_147_	finngen_R	outcome	8397_147_	MR Egger	53	-0.07732	0.099048	0.438616
8397_147_	finngen_R	outcome	8397_147_	Weighted	53	-0.05357	0.086817	0.537203
8397_147_	finngen_R	outcome	8397_147_	Inverse va	53	-0.10082	0.055384	0.068705
8397_147_	finngen_R	outcome	8397_147_	Simple mc	53	-0.0584	0.145895	0.690562
8397_147_	finngen_R	outcome	8397_147_	Weighted	53	-0.04228	0.077524	0.58784
8402_22_(	finngen_R	outcome	8402_22_(	MR Egger	37	-0.08964	0.080222	0.271453
8402_22_(	finngen_R	outcome	8402_22_(	Weighted	37	-0.09848	0.078364	0.20888
8402_22_(	finngen_R	outcome	8402_22_(	Inverse va	37	-0.10924	0.055018	0.047087
8402_22_(	finngen_R	outcome	8402_22_(	Simple mc	37	-0.121	0.142555	0.401591
8402_22_(	finngen_R	outcome	8402_22_(	Weighted	37	-0.08946	0.066711	0.188333
8403_18_F	finngen_R	outcome	8403_18_F	MR Egger	8	-0.70459	0.652506	0.321704
8403_18_F	finngen_R	outcome	8403_18_F	Weighted	8	-0.55057	0.296024	0.062904
8403_18_F	finngen_R	outcome	8403_18_F	Inverse va	8	-0.48132	0.221571	0.029833
8403_18_F	finngen_R	outcome	8403_18_F	Simple mc	8	-0.66037	0.444764	0.181184
8403_18_F	finngen_R	outcome	8403_18_F	Weighted	8	-0.61959	0.336151	0.10784
8427_118_	finngen_R	outcome	8427_118_	MR Egger	23	0.28688	0.206333	0.178977
8427_118_	finngen_R	outcome	8427_118_	Weighted	23	0.230963	0.125764	0.066288
8427_118_	finngen_R	outcome	8427_118_	Inverse va	23	0.374251	0.102491	0.000261
8427_118_	finngen_R	outcome	8427_118_	Simple mc	23	0.556874	0.202543	0.011702
8427_118_	finngen_R	outcome	8427_118_	Weighted	23	0.241628	0.125114	0.066434
8428_102_	finngen_R	outcome	8428_102_	MR Egger	86	0.002344	0.079732	0.976618
8428_102_	finngen_R	outcome	8428_102_	Weighted	86	-0.00274	0.068363	0.967974
8428_102_	finngen_R	outcome	8428_102_	Inverse va	86	0.054183	0.04578	0.236593
8428_102_	finngen_R	outcome	8428_102_	Simple mc	86	0.058672	0.125319	0.640852
8428_102_	finngen_R	outcome	8428_102_	Weighted	86	0.02727	0.062024	0.661288
8458_111_	finngen_R	outcome	8458_111_	Wald ratic	1	0.243393	0.755165	0.747222
8464_31_F	finngen_R	outcome	8464_31_F	MR Egger	6	-0.20774	0.624692	0.756177
8464_31_F	finngen_R	outcome	8464_31_F	Weighted	6	0.499214	0.275002	0.069476
8464_31_F	finngen_R	outcome	8464_31_F	Inverse va	6	0.372127	0.234233	0.112127
8464_31_F	finngen_R	outcome	8464_31_F	Simple mc	6	0.621128	0.377325	0.160654
8464_31_F	finngen_R	outcome	8464_31_F	Weighted	6	0.536748	0.323588	0.158065
8465_52_(	finngen_R	outcome	8465_52_(	MR Egger	151	0.032986	0.044899	0.463702
8465_52_(	finngen_R	outcome	8465_52_(	Weighted	151	0.018241	0.041156	0.657605
8465_52_(	finngen_R	outcome	8465_52_(	Inverse va	151	0.036279	0.027447	0.186246
8465_52_(	finngen_R	outcome	8465_52_(	Simple mc	151	-0.02301	0.082663	0.781127
8465_52_(	finngen_R	outcome	8465_52_(	Weighted	151	0.022863	0.040734	0.575439
8468_19_k	finngen_R	outcome	8468_19_k	Inverse va	2	0.75716	0.536463	0.158128
8469_41_I	finngen_R	outcome	8469_41_I	Inverse va	2	-0.06955	0.602842	0.908153
8476_11_(	finngen_R	outcome	8476_11_(	MR Egger	20	0.139518	0.155197	0.380536
8476_11_(	finngen_R	outcome	8476_11_(	Weighted	20	0.016875	0.131017	0.897513
8476_11_(	finngen_R	outcome	8476_11_(	Inverse va	20	0.035911	0.096435	0.709607
8476_11_(	finngen_R	outcome	8476_11_(	Simple mc	20	-0.05264	0.255365	0.838872
8476_11_(	finngen_R	outcome	8476_11_(	Weighted	20	0.017754	0.13752	0.898633
8479_4_M	finngen_R	outcome	8479_4_M	MR Egger	85	-0.01446	0.08999	0.87272
8479_4_M	finngen_R	outcome	8479_4_M	Weighted	85	0.007542	0.069899	0.914071
8479_4_M	finngen_R	outcome	8479_4_M	Inverse va	85	-0.00419	0.045783	0.927074
8479_4_M	finngen_R	outcome	8479_4_M	Simple mc	85	0.058004	0.124608	0.642785
8479_4_M	finngen_R	outcome	8479_4_M	Weighted	85	0.017327	0.072334	0.811269
8480_29_E	finngen_R	outcome	8480_29_E	MR Egger	10	-0.3184	0.350868	0.390675
8480_29_E	finngen_R	outcome	8480_29_E	Weighted	10	-0.18904	0.204367	0.354969
8480_29_E	finngen_R	outcome	8480_29_E	Inverse va	10	-0.05469	0.155158	0.72446
8480_29_E	finngen_R	outcome	8480_29_E	Simple mc	10	-0.12086	0.328223	0.721228
8480_29_E	finngen_R	outcome	8480_29_E	Weighted	10	-0.16359	0.21209	0.460283
8606_39_(	finngen_R	outcome	8606_39_(	MR Egger	43	-0.05864	0.085295	0.495655
8606_39_(	finngen_R	outcome	8606_39_(	Weighted	43	0.083524	0.088393	0.344702
8606_39_(	finngen_R	outcome	8606_39_(	Inverse va	43	0.032246	0.056978	0.571435
8606_39_(	finngen_R	outcome	8606_39_(	Simple mc	43	0.34618	0.206039	0.10035
8606_39_(	finngen_R	outcome	8606_39_(	Weighted	43	0.063795	0.081988	0.440874

8660_5_OI	finngen_R	outcome	8660_5_OI	MR Egger	3	0.171816	1.564805	0.930378
8660_5_OI	finngen_R	outcome	8660_5_OI	Weighted	3	0.141044	0.3477	0.685
8660_5_OI	finngen_R	outcome	8660_5_OI	Inverse va	3	0.276975	0.306659	0.366419
8660_5_OI	finngen_R	outcome	8660_5_OI	Simple mc	3	0.05613	0.479362	0.917485
8660_5_OI	finngen_R	outcome	8660_5_OI	Weighted	3	0.06194	0.415982	0.89529
8697_38_(	finngen_R	outcome	8697_38_(	MR Egger	53	0.090381	0.083553	0.284464
8697_38_(	finngen_R	outcome	8697_38_(	Weighted	53	0.073111	0.074303	0.325135
8697_38_(	finngen_R	outcome	8697_38_(	Inverse va	53	0.078483	0.055199	0.15508
8697_38_(	finngen_R	outcome	8697_38_(	Simple mc	53	0.144577	0.158712	0.366531
8697_38_(	finngen_R	outcome	8697_38_(	Weighted	53	0.088079	0.068512	0.20428
8766_29_L	finngen_R	outcome	8766_29_L	MR Egger	47	-0.02914	0.089918	0.747354
8766_29_L	finngen_R	outcome	8766_29_L	Weighted	47	-0.00295	0.071928	0.967249
8766_29_L	finngen_R	outcome	8766_29_L	Inverse va	47	-0.0123	0.052326	0.814196
8766_29_L	finngen_R	outcome	8766_29_L	Simple mc	47	0.131323	0.15262	0.394001
8766_29_L	finngen_R	outcome	8766_29_L	Weighted	47	0.026325	0.065428	0.689288
8773_172_	finngen_R	outcome	8773_172_	MR Egger	36	0.226949	0.115991	0.058647
8773_172_	finngen_R	outcome	8773_172_	Weighted	36	0.088149	0.09922	0.374314
8773_172_	finngen_R	outcome	8773_172_	Inverse va	36	0.06329	0.067059	0.345274
8773_172_	finngen_R	outcome	8773_172_	Simple mc	36	0.10186	0.145999	0.489987
8773_172_	finngen_R	outcome	8773_172_	Weighted	36	0.074918	0.102687	0.470504
8778_3_N(	finngen_R	outcome	8778_3_N(	MR Egger	17	-0.00215	0.146521	0.988501
8778_3_N(	finngen_R	outcome	8778_3_N(	Weighted	17	-0.04049	0.113571	0.721438
8778_3_N(	finngen_R	outcome	8778_3_N(	Inverse va	17	-0.02152	0.110174	0.845127
8778_3_N(	finngen_R	outcome	8778_3_N(	Simple mc	17	0.047198	0.211921	0.826576
8778_3_N(	finngen_R	outcome	8778_3_N(	Weighted	17	-0.04488	0.107381	0.681527
8794_13_[	finngen_R	outcome	8794_13_[	MR Egger	87	-0.00732	0.07671	0.92424
8794_13_[	finngen_R	outcome	8794_13_[	Weighted	87	-0.06355	0.064413	0.323813
8794_13_[	finngen_R	outcome	8794_13_[	Inverse va	87	-0.06219	0.038679	0.107846
8794_13_[	finngen_R	outcome	8794_13_[	Simple mc	87	-0.00746	0.115914	0.948853
8794_13_[	finngen_R	outcome	8794_13_[	Weighted	87	-0.0747	0.067352	0.270459
8795_48_T	finngen_R	outcome	8795_48_T	MR Egger	3	4.839327	7.163457	0.621763
8795_48_T	finngen_R	outcome	8795_48_T	Weighted	3	-0.15111	0.475225	0.7505
8795_48_T	finngen_R	outcome	8795_48_T	Inverse va	3	-0.21578	0.651856	0.740626
8795_48_T	finngen_R	outcome	8795_48_T	Simple mc	3	-0.00446	0.68885	0.995425
8795_48_T	finngen_R	outcome	8795_48_T	Weighted	3	-0.11819	0.593932	0.860663
8840_61_(	finngen_R	outcome	8840_61_(	MR Egger	11	-0.20644	0.282233	0.48312
8840_61_(	finngen_R	outcome	8840_61_(	Weighted	11	-0.06504	0.182993	0.722275
8840_61_(	finngen_R	outcome	8840_61_(	Inverse va	11	-0.07187	0.1454	0.621098
8840_61_(	finngen_R	outcome	8840_61_(	Simple mc	11	-0.1069	0.271834	0.702376
8840_61_(	finngen_R	outcome	8840_61_(	Weighted	11	-0.17024	0.221837	0.460573
8841_65_(	finngen_R	outcome	8841_65_(	MR Egger	5	0.105152	0.944337	0.91837
8841_65_(	finngen_R	outcome	8841_65_(	Weighted	5	0.270311	0.355769	0.447378
8841_65_(	finngen_R	outcome	8841_65_(	Inverse va	5	0.110168	0.294857	0.70868
8841_65_(	finngen_R	outcome	8841_65_(	Simple mc	5	0.425703	0.526878	0.464415
8841_65_(	finngen_R	outcome	8841_65_(	Weighted	5	0.297845	0.417476	0.514988
8859_51_(	finngen_R	outcome	8859_51_(	MR Egger	3	0.392807	0.458621	0.549112
8859_51_(	finngen_R	outcome	8859_51_(	Weighted	3	-0.13463	0.233211	0.563737
8859_51_(	finngen_R	outcome	8859_51_(	Inverse va	3	-0.22798	0.271703	0.40143
8859_51_(	finngen_R	outcome	8859_51_(	Simple mc	3	-0.27151	0.433561	0.595107
8859_51_(	finngen_R	outcome	8859_51_(	Weighted	3	-0.06462	0.251504	0.821255
8885_6_C/	finngen_R	outcome	8885_6_C/	MR Egger	62	-0.0944	0.174263	0.590041
8885_6_C/	finngen_R	outcome	8885_6_C/	Weighted	62	0.039237	0.112382	0.726982
8885_6_C/	finngen_R	outcome	8885_6_C/	Inverse va	62	0.079963	0.075441	0.289173
8885_6_C/	finngen_R	outcome	8885_6_C/	Simple mc	62	0.053516	0.235782	0.821205
8885_6_C/	finngen_R	outcome	8885_6_C/	Weighted	62	0.053516	0.200018	0.789945
8893_29_F	finngen_R	outcome	8893_29_F	Wald ratic	1	1.054227	0.694251	0.128886
8925_25_F	finngen_R	outcome	8925_25_F	MR Egger	4	0.568173	0.383912	0.277018
8925_25_F	finngen_R	outcome	8925_25_F	Weighted	4	0.478531	0.217203	0.027584

8925_25_F	finngen_R	outcome	8925_25_F	Inverse va	4	0.399806	0.211314	0.058491
8925_25_F	finngen_R	outcome	8925_25_F	Simple mc	4	0.607734	0.315728	0.14992
8925_25_F	finngen_R	outcome	8925_25_F	Weighted	4	0.424551	0.233465	0.166573
8932_1_EN	finngen_R	outcome	8932_1_EN	Inverse va	2	0.578676	0.314952	0.066159
8953_47_H	finngen_R	outcome	8953_47_H	Wald ratic	1	-0.89084	0.482778	0.065001
8958_51_C	finngen_R	outcome	8958_51_C	MR Egger	41	-0.07536	0.151859	0.622485
8958_51_C	finngen_R	outcome	8958_51_C	Weighted	41	-0.01326	0.096631	0.890815
8958_51_C	finngen_R	outcome	8958_51_C	Inverse va	41	0.039552	0.066054	0.54932
8958_51_C	finngen_R	outcome	8958_51_C	Simple mc	41	-0.13401	0.151511	0.381707
8958_51_C	finngen_R	outcome	8958_51_C	Weighted	41	-0.03847	0.103001	0.710749
8969_49_C	finngen_R	outcome	8969_49_C	MR Egger	19	-0.43947	0.266807	0.117886
8969_49_C	finngen_R	outcome	8969_49_C	Weighted	19	-0.43182	0.161205	0.007391
8969_49_C	finngen_R	outcome	8969_49_C	Inverse va	19	-0.2738	0.115623	0.017883
8969_49_C	finngen_R	outcome	8969_49_C	Simple mc	19	-0.54185	0.263833	0.05482
8969_49_C	finngen_R	outcome	8969_49_C	Weighted	19	-0.49943	0.173418	0.009968
8970_9_RI	finngen_R	outcome	8970_9_RI	Wald ratic	1	0.069125	0.530731	0.896373
8973_23_F	finngen_R	outcome	8973_23_F	MR Egger	145	-0.03072	0.039867	0.442248
8973_23_F	finngen_R	outcome	8973_23_F	Weighted	145	-0.06564	0.037993	0.084025
8973_23_F	finngen_R	outcome	8973_23_F	Inverse va	145	-0.07143	0.024963	0.004216
8973_23_F	finngen_R	outcome	8973_23_F	Simple mc	145	-0.12651	0.070241	0.073795
8973_23_F	finngen_R	outcome	8973_23_F	Weighted	145	-0.06741	0.037829	0.076863
8974_172_	finngen_R	outcome	8974_172_	MR Egger	21	-0.22753	0.210561	0.293399
8974_172_	finngen_R	outcome	8974_172_	Weighted	21	-0.0935	0.128474	0.466742
8974_172_	finngen_R	outcome	8974_172_	Inverse va	21	-0.00769	0.092467	0.933728
8974_172_	finngen_R	outcome	8974_172_	Simple mc	21	-0.04478	0.223523	0.84323
8974_172_	finngen_R	outcome	8974_172_	Weighted	21	-0.12166	0.155129	0.442069
8982_65_T	finngen_R	outcome	8982_65_T	MR Egger	6	0.534072	0.485084	0.332701
8982_65_T	finngen_R	outcome	8982_65_T	Weighted	6	-0.10847	0.338259	0.748457
8982_65_T	finngen_R	outcome	8982_65_T	Inverse va	6	0.092307	0.257399	0.719884
8982_65_T	finngen_R	outcome	8982_65_T	Simple mc	6	-0.04612	0.417339	0.916296
8982_65_T	finngen_R	outcome	8982_65_T	Weighted	6	-0.114	0.368186	0.769341
8989_40_S	finngen_R	outcome	8989_40_S	MR Egger	18	-0.22754	0.333261	0.504511
8989_40_S	finngen_R	outcome	8989_40_S	Weighted	18	-0.29252	0.151669	0.053774
8989_40_S	finngen_R	outcome	8989_40_S	Inverse va	18	-0.1553	0.113174	0.169984
8989_40_S	finngen_R	outcome	8989_40_S	Simple mc	18	-0.32738	0.21732	0.150307
8989_40_S	finngen_R	outcome	8989_40_S	Weighted	18	-0.3455	0.174414	0.064019
9002_36_S	finngen_R	outcome	9002_36_S	MR Egger	41	-0.22966	0.140571	0.110353
9002_36_S	finngen_R	outcome	9002_36_S	Weighted	41	-0.15959	0.106177	0.132817
9002_36_S	finngen_R	outcome	9002_36_S	Inverse va	41	-0.17438	0.068692	0.011131
9002_36_S	finngen_R	outcome	9002_36_S	Simple mc	41	-0.07957	0.194418	0.684506
9002_36_S	finngen_R	outcome	9002_36_S	Weighted	41	-0.16672	0.107675	0.129412
9017_58_L	finngen_R	outcome	9017_58_L	MR Egger	56	-0.01555	0.03795	0.683571
9017_58_L	finngen_R	outcome	9017_58_L	Weighted	56	0.020302	0.032354	0.530346
9017_58_L	finngen_R	outcome	9017_58_L	Inverse va	56	0.027216	0.021188	0.198963
9017_58_L	finngen_R	outcome	9017_58_L	Simple mc	56	0.052455	0.064156	0.417108
9017_58_L	finngen_R	outcome	9017_58_L	Weighted	56	0.01337	0.044264	0.763762
9026_40_E	finngen_R	outcome	9026_40_E	Wald ratic	1	-0.3731	0.628586	0.552808
9076_25_F	finngen_R	outcome	9076_25_F	MR Egger	91	-0.04101	0.066103	0.536583
9076_25_F	finngen_R	outcome	9076_25_F	Weighted	91	-0.05341	0.051498	0.299634
9076_25_F	finngen_R	outcome	9076_25_F	Inverse va	91	-0.02542	0.031846	0.424679
9076_25_F	finngen_R	outcome	9076_25_F	Simple mc	91	-0.07526	0.097452	0.441975
9076_25_F	finngen_R	outcome	9076_25_F	Weighted	91	-0.06259	0.057181	0.276613
9092_33_A	finngen_R	outcome	9092_33_A	MR Egger	5	0.820789	1.072362	0.499737
9092_33_A	finngen_R	outcome	9092_33_A	Weighted	5	-0.08983	0.368899	0.807615
9092_33_A	finngen_R	outcome	9092_33_A	Inverse va	5	-0.41399	0.298544	0.165537
9092_33_A	finngen_R	outcome	9092_33_A	Simple mc	5	-0.22141	0.579362	0.721773
9092_33_A	finngen_R	outcome	9092_33_A	Weighted	5	-0.07666	0.450775	0.873216
9094_5_Cl	finngen_R	outcome	9094_5_Cl	MR Egger	8	-0.27529	0.735354	0.721006

9094_5_Cl	finngen_R	outcome	9094_5_Cl	Weighted	8	-0.23139	0.29527	0.43325
9094_5_Cl	finngen_R	outcome	9094_5_Cl	Inverse va	8	-0.07203	0.221194	0.744685
9094_5_Cl	finngen_R	outcome	9094_5_Cl	Simple mc	8	-0.34322	0.468361	0.487478
9094_5_Cl	finngen_R	outcome	9094_5_Cl	Weighted	8	-0.36893	0.411686	0.39994
9172_69_N	finngen_R	outcome	9172_69_N	MR Egger	17	-0.35553	0.210038	0.111177
9172_69_N	finngen_R	outcome	9172_69_N	Weighted	17	-0.2001	0.151642	0.186976
9172_69_N	finngen_R	outcome	9172_69_N	Inverse va	17	-0.17643	0.147636	0.232074
9172_69_N	finngen_R	outcome	9172_69_N	Simple mc	17	-0.4342	0.256403	0.109751
9172_69_N	finngen_R	outcome	9172_69_N	Weighted	17	-0.2444	0.159847	0.145799
9175_48_D	finngen_R	outcome	9175_48_D	MR Egger	7	-0.13137	0.26002	0.634885
9175_48_D	finngen_R	outcome	9175_48_D	Weighted	7	-0.11668	0.189505	0.538079
9175_48_D	finngen_R	outcome	9175_48_D	Inverse va	7	-0.04891	0.166008	0.768294
9175_48_D	finngen_R	outcome	9175_48_D	Simple mc	7	-0.18386	0.274093	0.527324
9175_48_D	finngen_R	outcome	9175_48_D	Weighted	7	-0.12777	0.207292	0.560283
9177_6_FA	finngen_R	outcome	9177_6_FA	MR Egger	63	0.03382	0.075569	0.656068
9177_6_FA	finngen_R	outcome	9177_6_FA	Weighted	63	-0.00045	0.080164	0.995515
9177_6_FA	finngen_R	outcome	9177_6_FA	Inverse va	63	0.059233	0.051281	0.24806
9177_6_FA	finngen_R	outcome	9177_6_FA	Simple mc	63	-0.05166	0.169971	0.762197
9177_6_FA	finngen_R	outcome	9177_6_FA	Weighted	63	-0.00326	0.077684	0.966619
9183_7_IFI	finngen_R	outcome	9183_7_IFI	MR Egger	89	-0.02082	0.060147	0.73012
9183_7_IFI	finngen_R	outcome	9183_7_IFI	Weighted	89	-0.06349	0.05406	0.240233
9183_7_IFI	finngen_R	outcome	9183_7_IFI	Inverse va	89	-0.06378	0.036447	0.080129
9183_7_IFI	finngen_R	outcome	9183_7_IFI	Simple mc	89	-0.09297	0.099155	0.351025
9183_7_IFI	finngen_R	outcome	9183_7_IFI	Weighted	89	-0.03321	0.054104	0.540967
9185_15_T	finngen_R	outcome	9185_15_T	MR Egger	26	-0.15708	0.136219	0.26022
9185_15_T	finngen_R	outcome	9185_15_T	Weighted	26	-0.16969	0.11267	0.13204
9185_15_T	finngen_R	outcome	9185_15_T	Inverse va	26	-0.07424	0.073443	0.312058
9185_15_T	finngen_R	outcome	9185_15_T	Simple mc	26	-0.22179	0.221969	0.327284
9185_15_T	finngen_R	outcome	9185_15_T	Weighted	26	-0.17148	0.1164	0.153173
9188_119_	finngen_R	outcome	9188_119_	Inverse va	2	-0.13721	0.562779	0.807376
9191_8_TF	finngen_R	outcome	9191_8_TF	MR Egger	4	1.298048	1.118687	0.365699
9191_8_TF	finngen_R	outcome	9191_8_TF	Weighted	4	0.436282	0.36723	0.234819
9191_8_TF	finngen_R	outcome	9191_8_TF	Inverse va	4	0.247723	0.292922	0.397722
9191_8_TF	finngen_R	outcome	9191_8_TF	Simple mc	4	0.420866	0.483328	0.447959
9191_8_TF	finngen_R	outcome	9191_8_TF	Weighted	4	0.468212	0.433557	0.359252
9197_4_LC	finngen_R	outcome	9197_4_LC	MR Egger	5	-0.45524	0.773998	0.597767
9197_4_LC	finngen_R	outcome	9197_4_LC	Weighted	5	-0.33408	0.251761	0.184522
9197_4_LC	finngen_R	outcome	9197_4_LC	Inverse va	5	-0.59353	0.239687	0.013276
9197_4_LC	finngen_R	outcome	9197_4_LC	Simple mc	5	-1.36148	0.580326	0.078854
9197_4_LC	finngen_R	outcome	9197_4_LC	Weighted	5	-0.30186	0.274184	0.332718
9204_33_F	finngen_R	outcome	9204_33_F	MR Egger	4	-0.63709	1.001227	0.589682
9204_33_F	finngen_R	outcome	9204_33_F	Weighted	4	0.302948	0.306343	0.322704
9204_33_F	finngen_R	outcome	9204_33_F	Inverse va	4	0.383326	0.259146	0.139089
9204_33_F	finngen_R	outcome	9204_33_F	Simple mc	4	0.208732	0.453183	0.676427
9204_33_F	finngen_R	outcome	9204_33_F	Weighted	4	0.247206	0.437715	0.611708
9211_19_S	finngen_R	outcome	9211_19_S	MR Egger	26	-0.04505	0.141309	0.752657
9211_19_S	finngen_R	outcome	9211_19_S	Weighted	26	-0.00568	0.106156	0.957363
9211_19_S	finngen_R	outcome	9211_19_S	Inverse va	26	0.146606	0.075115	0.050969
9211_19_S	finngen_R	outcome	9211_19_S	Simple mc	26	0.057505	0.188884	0.763307
9211_19_S	finngen_R	outcome	9211_19_S	Weighted	26	0.03453	0.107942	0.751703
9212_22_C	finngen_R	outcome	9212_22_C	MR Egger	17	0.570338	0.449692	0.224028
9212_22_C	finngen_R	outcome	9212_22_C	Weighted	17	0.188618	0.197693	0.340036
9212_22_C	finngen_R	outcome	9212_22_C	Inverse va	17	0.109463	0.142128	0.441198
9212_22_C	finngen_R	outcome	9212_22_C	Simple mc	17	0.314233	0.324335	0.347037
9212_22_C	finngen_R	outcome	9212_22_C	Weighted	17	0.256458	0.266425	0.350076
9223_11_N	finngen_R	outcome	9223_11_N	Inverse va	2	0.524865	0.444104	0.237264
9233_71_T	finngen_R	outcome	9233_71_T	Inverse va	2	-0.49862	0.500408	0.319047
9234_8_TV	finngen_R	outcome	9234_8_TV	MR Egger	8	0.701745	0.718438	0.366407

9234_8_TV	finngen_R	outcome	9234_8_TV	Weighted	8	0.056446	0.244644	0.817528
9234_8_TV	finngen_R	outcome	9234_8_TV	Inverse va	8	0.086899	0.195857	0.65727
9234_8_TV	finngen_R	outcome	9234_8_TV	Simple mc	8	-0.07045	0.361218	0.850902
9234_8_TV	finngen_R	outcome	9234_8_TV	Weighted	8	-0.04532	0.329364	0.894439
9241_40_S	finngen_R	outcome	9241_40_S	MR Egger	7	0.45934	0.428763	0.332992
9241_40_S	finngen_R	outcome	9241_40_S	Weighted	7	0.143534	0.219709	0.513568
9241_40_S	finngen_R	outcome	9241_40_S	Inverse va	7	0.078036	0.189726	0.680846
9241_40_S	finngen_R	outcome	9241_40_S	Simple mc	7	0.09393	0.379916	0.812965
9241_40_S	finngen_R	outcome	9241_40_S	Weighted	7	0.162661	0.241452	0.525602
9244_27_F	finngen_R	outcome	9244_27_F	MR Egger	11	0.098823	0.21673	0.65921
9244_27_F	finngen_R	outcome	9244_27_F	Weighted	11	0.025716	0.140249	0.854514
9244_27_F	finngen_R	outcome	9244_27_F	Inverse va	11	-0.02194	0.115444	0.849273
9244_27_F	finngen_R	outcome	9244_27_F	Simple mc	11	-0.03567	0.228734	0.879175
9244_27_F	finngen_R	outcome	9244_27_F	Weighted	11	0.024487	0.143154	0.867592
9263_57_S	finngen_R	outcome	9263_57_S	Wald ratic	1	-0.17917	0.35415	0.612913
9266_1_TF	finngen_R	outcome	9266_1_TF	MR Egger	61	-0.23177	0.088611	0.011291
9266_1_TF	finngen_R	outcome	9266_1_TF	Weighted	61	-0.1553	0.074486	0.037075
9266_1_TF	finngen_R	outcome	9266_1_TF	Inverse va	61	-0.15087	0.04996	0.00253
9266_1_TF	finngen_R	outcome	9266_1_TF	Simple mc	61	-0.14412	0.133677	0.285303
9266_1_TF	finngen_R	outcome	9266_1_TF	Weighted	61	-0.16794	0.077199	0.033549
9267_2_CF	finngen_R	outcome	9267_2_CF	MR Egger	178	-0.0344	0.02736	0.21036
9267_2_CF	finngen_R	outcome	9267_2_CF	Weighted	178	-0.04029	0.031671	0.203277
9267_2_CF	finngen_R	outcome	9267_2_CF	Inverse va	178	-0.00081	0.017606	0.963284
9267_2_CF	finngen_R	outcome	9267_2_CF	Simple mc	178	0.125137	0.05832	0.033258
9267_2_CF	finngen_R	outcome	9267_2_CF	Weighted	178	-0.02422	0.027294	0.376111
9275_2_C1	finngen_R	outcome	9275_2_C1	MR Egger	192	-0.00243	0.028622	0.932463
9275_2_C1	finngen_R	outcome	9275_2_C1	Weighted	192	-0.02186	0.028511	0.44315
9275_2_C1	finngen_R	outcome	9275_2_C1	Inverse va	192	-0.0066	0.018177	0.7165
9275_2_C1	finngen_R	outcome	9275_2_C1	Simple mc	192	0.045977	0.056069	0.413232
9275_2_C1	finngen_R	outcome	9275_2_C1	Weighted	192	-0.01196	0.027031	0.658576
9278_9_EF	finngen_R	outcome	9278_9_EF	MR Egger	7	0.668089	1.410856	0.655786
9278_9_EF	finngen_R	outcome	9278_9_EF	Weighted	7	-0.38335	0.32123	0.232725
9278_9_EF	finngen_R	outcome	9278_9_EF	Inverse va	7	-0.60218	0.277205	0.02983
9278_9_EF	finngen_R	outcome	9278_9_EF	Simple mc	7	-0.36493	0.493088	0.487178
9278_9_EF	finngen_R	outcome	9278_9_EF	Weighted	7	-0.32486	0.405453	0.453557
9282_12_(	finngen_R	outcome	9282_12_(	MR Egger	73	-0.05541	0.051734	0.287781
9282_12_(	finngen_R	outcome	9282_12_(	Weighted	73	-0.05983	0.043898	0.172934
9282_12_(	finngen_R	outcome	9282_12_(	Inverse va	73	-0.09972	0.028561	0.00048
9282_12_(	finngen_R	outcome	9282_12_(	Simple mc	73	-0.05198	0.089618	0.56375
9282_12_(	finngen_R	outcome	9282_12_(	Weighted	73	-0.02938	0.044696	0.513084
9288_7_FK	finngen_R	outcome	9288_7_FK	MR Egger	30	-0.01078	0.122077	0.930272
9288_7_FK	finngen_R	outcome	9288_7_FK	Weighted	30	0.048483	0.110978	0.662203
9288_7_FK	finngen_R	outcome	9288_7_FK	Inverse va	30	0.104908	0.07362	0.154159
9288_7_FK	finngen_R	outcome	9288_7_FK	Simple mc	30	0.177246	0.158027	0.271224
9288_7_FK	finngen_R	outcome	9288_7_FK	Weighted	30	0.048671	0.102661	0.638984
9294_45_N	finngen_R	outcome	9294_45_N	MR Egger	18	0.321618	0.289223	0.282565
9294_45_N	finngen_R	outcome	9294_45_N	Weighted	18	0.122983	0.187342	0.511527
9294_45_N	finngen_R	outcome	9294_45_N	Inverse va	18	0.027174	0.130557	0.835121
9294_45_N	finngen_R	outcome	9294_45_N	Simple mc	18	0.05039	0.265565	0.851755
9294_45_N	finngen_R	outcome	9294_45_N	Weighted	18	0.130308	0.202917	0.529328
9312_8_AZ	finngen_R	outcome	9312_8_AZ	MR Egger	16	0.19828	0.381988	0.611817
9312_8_AZ	finngen_R	outcome	9312_8_AZ	Weighted	16	0.000803	0.184827	0.996535
9312_8_AZ	finngen_R	outcome	9312_8_AZ	Inverse va	16	-0.05102	0.141197	0.717869
9312_8_AZ	finngen_R	outcome	9312_8_AZ	Simple mc	16	0.027355	0.279566	0.923347
9312_8_AZ	finngen_R	outcome	9312_8_AZ	Weighted	16	0.006077	0.231647	0.979416
9313_27_(	finngen_R	outcome	9313_27_(	MR Egger	56	-0.04898	0.147225	0.740667
9313_27_(	finngen_R	outcome	9313_27_(	Weighted	56	-0.02056	0.096236	0.830842
9313_27_(	finngen_R	outcome	9313_27_(	Inverse va	56	-0.05081	0.07098	0.474067

9313_27_(	finngen_R	outcome	9313_27_(	Simple mc	56	0.102875	0.207009	0.621199
9313_27_(	finngen_R	outcome	9313_27_(	Weighted	56	-0.0215	0.162782	0.895403
9321_400_	finngen_R	outcome	9321_400_	MR Egger	11	-0.14423	0.533128	0.792857
9321_400_	finngen_R	outcome	9321_400_	Weighted	11	0.071457	0.224967	0.750764
9321_400_	finngen_R	outcome	9321_400_	Inverse va	11	0.040235	0.163711	0.805861
9321_400_	finngen_R	outcome	9321_400_	Simple mc	11	0.03719	0.317338	0.909027
9321_400_	finngen_R	outcome	9321_400_	Weighted	11	0.067727	0.238535	0.782257
9326_33_I	finngen_R	outcome	9326_33_I	MR Egger	80	0.045191	0.053569	0.401478
9326_33_I	finngen_R	outcome	9326_33_I	Weighted	80	0.00345	0.051054	0.946131
9326_33_I	finngen_R	outcome	9326_33_I	Inverse va	80	0.072065	0.039012	0.064708
9326_33_I	finngen_R	outcome	9326_33_I	Simple mc	80	0.142207	0.111527	0.206015
9326_33_I	finngen_R	outcome	9326_33_I	Weighted	80	0.037518	0.044952	0.406442
9337_43_T	finngen_R	outcome	9337_43_T	MR Egger	20	0.012082	0.21409	0.955619
9337_43_T	finngen_R	outcome	9337_43_T	Weighted	20	0.05162	0.126582	0.68342
9337_43_T	finngen_R	outcome	9337_43_T	Inverse va	20	0.083468	0.09627	0.385932
9337_43_T	finngen_R	outcome	9337_43_T	Simple mc	20	-0.02277	0.190212	0.905969
9337_43_T	finngen_R	outcome	9337_43_T	Weighted	20	0.028231	0.13821	0.84032
9339_204_	finngen_R	outcome	9339_204_	Wald ratic	1	-0.35327	0.786703	0.653394
9341_1_PI	finngen_R	outcome	9341_1_PI	MR Egger	46	-0.11295	0.123059	0.363707
9341_1_PI	finngen_R	outcome	9341_1_PI	Weighted	46	-0.04196	0.096274	0.662923
9341_1_PI	finngen_R	outcome	9341_1_PI	Inverse va	46	0.032232	0.060688	0.595342
9341_1_PI	finngen_R	outcome	9341_1_PI	Simple mc	46	-0.05733	0.16522	0.730221
9341_1_PI	finngen_R	outcome	9341_1_PI	Weighted	46	-0.07369	0.093003	0.432319
9343_16_II	finngen_R	outcome	9343_16_II	Inverse va	2	-0.30021	0.328277	0.360446
9348_1_C1	finngen_R	outcome	9348_1_C1	MR Egger	12	0.082753	0.233289	0.730163
9348_1_C1	finngen_R	outcome	9348_1_C1	Weighted	12	0.075956	0.199238	0.703033
9348_1_C1	finngen_R	outcome	9348_1_C1	Inverse va	12	0.042706	0.147983	0.772899
9348_1_C1	finngen_R	outcome	9348_1_C1	Simple mc	12	-0.22881	0.293906	0.45268
9348_1_C1	finngen_R	outcome	9348_1_C1	Weighted	12	0.036288	0.268968	0.895116
9350_3_FS	finngen_R	outcome	9350_3_FS	MR Egger	4	-0.71791	1.782855	0.726152
9350_3_FS	finngen_R	outcome	9350_3_FS	Weighted	4	0.278526	0.395068	0.480806
9350_3_FS	finngen_R	outcome	9350_3_FS	Inverse va	4	0.312725	0.332899	0.347525
9350_3_FS	finngen_R	outcome	9350_3_FS	Simple mc	4	0.305029	0.529702	0.605074
9350_3_FS	finngen_R	outcome	9350_3_FS	Weighted	4	0.229065	0.450026	0.645824
9357_4_CI	finngen_R	outcome	9357_4_CI	MR Egger	9	-0.03847	0.313789	0.905872
9357_4_CI	finngen_R	outcome	9357_4_CI	Weighted	9	0.055832	0.18527	0.763144
9357_4_CI	finngen_R	outcome	9357_4_CI	Inverse va	9	0.041848	0.13807	0.761821
9357_4_CI	finngen_R	outcome	9357_4_CI	Simple mc	9	0.219831	0.313351	0.502855
9357_4_CI	finngen_R	outcome	9357_4_CI	Weighted	9	0.286475	0.250026	0.285002
9360_33_E	finngen_R	outcome	9360_33_E	MR Egger	5	-0.21087	0.810693	0.81161
9360_33_E	finngen_R	outcome	9360_33_E	Weighted	5	0.211953	0.382962	0.57995
9360_33_E	finngen_R	outcome	9360_33_E	Inverse va	5	0.390595	0.371852	0.293532
9360_33_E	finngen_R	outcome	9360_33_E	Simple mc	5	-0.21995	0.616938	0.739461
9360_33_E	finngen_R	outcome	9360_33_E	Weighted	5	-0.07783	0.474811	0.877752
9369_174_	finngen_R	outcome	9369_174_	MR Egger	18	0.109519	0.375557	0.774327
9369_174_	finngen_R	outcome	9369_174_	Weighted	18	-0.00455	0.170513	0.978733
9369_174_	finngen_R	outcome	9369_174_	Inverse va	18	0.025316	0.130875	0.84662
9369_174_	finngen_R	outcome	9369_174_	Simple mc	18	-0.09239	0.281019	0.746357
9369_174_	finngen_R	outcome	9369_174_	Weighted	18	0.016267	0.17176	0.925655
9370_69_(	finngen_R	outcome	9370_69_(	MR Egger	44	0.06523	0.082206	0.431951
9370_69_(	finngen_R	outcome	9370_69_(	Weighted	44	0.117621	0.073358	0.108848
9370_69_(	finngen_R	outcome	9370_69_(	Inverse va	44	0.19274	0.051325	0.000173
9370_69_(	finngen_R	outcome	9370_69_(	Simple mc	44	0.187324	0.123239	0.135829
9370_69_(	finngen_R	outcome	9370_69_(	Weighted	44	0.119435	0.069997	0.095171
9380_2_PL	finngen_R	outcome	9380_2_PL	MR Egger	4	-1.24844	0.577317	0.163081
9380_2_PL	finngen_R	outcome	9380_2_PL	Weighted	4	-0.49405	0.327804	0.131775
9380_2_PL	finngen_R	outcome	9380_2_PL	Inverse va	4	-0.2285	0.306856	0.456489
9380_2_PL	finngen_R	outcome	9380_2_PL	Simple mc	4	-0.51927	0.448574	0.330824

9380_2_PL	finngen_R	outcome	9380_2_PL	Weighted	4	-0.50354	0.401235	0.298352
9383_24_(	finngen_R	outcome	9383_24_(	Wald ratic	1	-0.36109	0.641012	0.573222
9384_17_(	finngen_R	outcome	9384_17_(	MR Egger	5	-0.42118	0.378267	0.346701
9384_17_(	finngen_R	outcome	9384_17_(	Weighted	5	-0.23791	0.226959	0.29452
9384_17_(	finngen_R	outcome	9384_17_(	Inverse va	5	-0.091	0.205648	0.658131
9384_17_(	finngen_R	outcome	9384_17_(	Simple mc	5	-0.27464	0.28186	0.385046
9384_17_(	finngen_R	outcome	9384_17_(	Weighted	5	-0.28517	0.261082	0.336096
9385_4_G/	finngen_R	outcome	9385_4_G/	MR Egger	91	-0.00328	0.081293	0.967919
9385_4_G/	finngen_R	outcome	9385_4_G/	Weighted	91	-0.04854	0.068568	0.479013
9385_4_G/	finngen_R	outcome	9385_4_G/	Inverse va	91	#######	0.044659	0.998769
9385_4_G/	finngen_R	outcome	9385_4_G/	Simple mc	91	-0.07947	0.150462	0.598663
9385_4_G/	finngen_R	outcome	9385_4_G/	Weighted	91	-0.13529	0.111967	0.230082
9394_19_(	finngen_R	outcome	9394_19_(	MR Egger	54	-0.12314	0.063249	0.056947
9394_19_(	finngen_R	outcome	9394_19_(	Weighted	54	-0.07755	0.063413	0.22134
9394_19_(	finngen_R	outcome	9394_19_(	Inverse va	54	-0.12239	0.040872	0.002748
9394_19_(	finngen_R	outcome	9394_19_(	Simple mc	54	-0.09691	0.095869	0.316702
9394_19_(	finngen_R	outcome	9394_19_(	Weighted	54	-0.08784	0.052762	0.101862
9398_30_(	finngen_R	outcome	9398_30_(	Wald ratic	1	0.579375	0.561594	0.302231
9409_11_T	finngen_R	outcome	9409_11_T	MR Egger	142	0.024956	0.030554	0.415435
9409_11_T	finngen_R	outcome	9409_11_T	Weighted	142	0.009961	0.037204	0.788902
9409_11_T	finngen_R	outcome	9409_11_T	Inverse va	142	0.012538	0.02171	0.563577
9409_11_T	finngen_R	outcome	9409_11_T	Simple mc	142	-0.00796	0.074786	0.915349
9409_11_T	finngen_R	outcome	9409_11_T	Weighted	142	0.01772	0.031505	0.574688
9416_77_(	finngen_R	outcome	9416_77_(	MR Egger	10	-0.27879	0.564604	0.634737
9416_77_(	finngen_R	outcome	9416_77_(	Weighted	10	-0.31216	0.233696	0.181625
9416_77_(	finngen_R	outcome	9416_77_(	Inverse va	10	-0.26601	0.179165	0.137625
9416_77_(	finngen_R	outcome	9416_77_(	Simple mc	10	-0.47613	0.390292	0.253496
9416_77_(	finngen_R	outcome	9416_77_(	Weighted	10	-0.41933	0.35273	0.264931
9449_150_	finngen_R	outcome	9449_150_	MR Egger	28	0.137491	0.120653	0.264861
9449_150_	finngen_R	outcome	9449_150_	Weighted	28	0.153079	0.109576	0.16241
9449_150_	finngen_R	outcome	9449_150_	Inverse va	28	0.21098	0.079189	0.007716
9449_150_	finngen_R	outcome	9449_150_	Simple mc	28	0.417716	0.220536	0.068972
9449_150_	finngen_R	outcome	9449_150_	Weighted	28	0.177613	0.097656	0.080057
9459_7_FA	finngen_R	outcome	9459_7_FA	MR Egger	23	-0.03607	0.177816	0.841219
9459_7_FA	finngen_R	outcome	9459_7_FA	Weighted	23	0.064424	0.125368	0.607338
9459_7_FA	finngen_R	outcome	9459_7_FA	Inverse va	23	0.122332	0.096371	0.204301
9459_7_FA	finngen_R	outcome	9459_7_FA	Simple mc	23	0.176632	0.223124	0.437029
9459_7_FA	finngen_R	outcome	9459_7_FA	Weighted	23	0.081359	0.131848	0.543524
9525_1_PT	finngen_R	outcome	9525_1_PT	MR Egger	4	-0.81999	1.713573	0.679482
9525_1_PT	finngen_R	outcome	9525_1_PT	Weighted	4	-0.63617	0.314054	0.042799
9525_1_PT	finngen_R	outcome	9525_1_PT	Inverse va	4	-0.63176	0.280034	0.02407
9525_1_PT	finngen_R	outcome	9525_1_PT	Simple mc	4	-0.65702	0.393561	0.193626
9525_1_PT	finngen_R	outcome	9525_1_PT	Weighted	4	-0.64271	0.380237	0.189552
9545_156_	finngen_R	outcome	9545_156_	MR Egger	8	-0.25159	0.68366	0.725505
9545_156_	finngen_R	outcome	9545_156_	Weighted	8	-0.21779	0.218389	0.318639
9545_156_	finngen_R	outcome	9545_156_	Inverse va	8	-0.21807	0.266059	0.412433
9545_156_	finngen_R	outcome	9545_156_	Simple mc	8	-0.4271	0.424424	0.347784
9545_156_	finngen_R	outcome	9545_156_	Weighted	8	-0.25872	0.2156	0.269175
9580_5_LA	finngen_R	outcome	9580_5_LA	MR Egger	71	0.07076	0.064328	0.275161
9580_5_LA	finngen_R	outcome	9580_5_LA	Weighted	71	-0.00633	0.060713	0.91692
9580_5_LA	finngen_R	outcome	9580_5_LA	Inverse va	71	-0.04377	0.041248	0.288624
9580_5_LA	finngen_R	outcome	9580_5_LA	Simple mc	71	-0.02774	0.092999	0.76635
9580_5_LA	finngen_R	outcome	9580_5_LA	Weighted	71	-0.01761	0.050528	0.728511
9713_67_F	finngen_R	outcome	9713_67_F	MR Egger	39	0.107462	0.092633	0.253444
9713_67_F	finngen_R	outcome	9713_67_F	Weighted	39	0.135404	0.090265	0.133595
9713_67_F	finngen_R	outcome	9713_67_F	Inverse va	39	0.121912	0.064419	0.058428
9713_67_F	finngen_R	outcome	9713_67_F	Simple mc	39	0.099924	0.21005	0.637001
9713_67_F	finngen_R	outcome	9713_67_F	Weighted	39	0.147228	0.086316	0.096231

9747_48_N	finngen_R	outcome	9747_48_N	Inverse va	2	-0.06541	0.455894	0.885907
9748_31_C	finngen_R	outcome	9748_31_C	MR Egger	115	0.007753	0.049916	0.87684
9748_31_C	finngen_R	outcome	9748_31_C	Weighted	115	-0.01296	0.044319	0.769887
9748_31_C	finngen_R	outcome	9748_31_C	Inverse va	115	0.015384	0.029976	0.607811
9748_31_C	finngen_R	outcome	9748_31_C	Simple mc	115	0.084452	0.086268	0.329682
9748_31_C	finngen_R	outcome	9748_31_C	Weighted	115	0.005963	0.046917	0.899092
9754_33_N	finngen_R	outcome	9754_33_N	MR Egger	164	0.0754	0.035408	0.034725
9754_33_N	finngen_R	outcome	9754_33_N	Weighted	164	0.050308	0.039787	0.206079
9754_33_N	finngen_R	outcome	9754_33_N	Inverse va	164	0.024697	0.022631	0.275129
9754_33_N	finngen_R	outcome	9754_33_N	Simple mc	164	0.068914	0.074099	0.353734
9754_33_N	finngen_R	outcome	9754_33_N	Weighted	164	0.077229	0.033678	0.023114
9759_13_II	finngen_R	outcome	9759_13_II	Wald ratic	1	0.908494	0.640795	0.156261
9772_153_	finngen_R	outcome	9772_153_	MR Egger	7	-0.92581	0.616897	0.193716
9772_153_	finngen_R	outcome	9772_153_	Weighted	7	-0.05173	0.24182	0.830613
9772_153_	finngen_R	outcome	9772_153_	Inverse va	7	-0.09952	0.197245	0.613891
9772_153_	finngen_R	outcome	9772_153_	Simple mc	7	0.19752	0.377728	0.619773
9772_153_	finngen_R	outcome	9772_153_	Weighted	7	-0.08424	0.258	0.755129
9790_28_E	finngen_R	outcome	9790_28_E	MR Egger	6	0.045506	0.326353	0.895843
9790_28_E	finngen_R	outcome	9790_28_E	Weighted	6	0.061668	0.191966	0.748025
9790_28_E	finngen_R	outcome	9790_28_E	Inverse va	6	-0.0808	0.16087	0.615466
9790_28_E	finngen_R	outcome	9790_28_E	Simple mc	6	0.043613	0.368984	0.910512
9790_28_E	finngen_R	outcome	9790_28_E	Weighted	6	0.089146	0.201334	0.676428
9796_4_CE	finngen_R	outcome	9796_4_CE	MR Egger	62	0.009612	0.079618	0.90431
9796_4_CE	finngen_R	outcome	9796_4_CE	Weighted	62	0.027249	0.070019	0.697157
9796_4_CE	finngen_R	outcome	9796_4_CE	Inverse va	62	-0.04014	0.054927	0.464931
9796_4_CE	finngen_R	outcome	9796_4_CE	Simple mc	62	-0.00981	0.130973	0.940515
9796_4_CE	finngen_R	outcome	9796_4_CE	Weighted	62	0.015233	0.060726	0.802776
9829_91_S	finngen_R	outcome	9829_91_S	MR Egger	5	0.465605	0.403357	0.331973
9829_91_S	finngen_R	outcome	9829_91_S	Weighted	5	0.043423	0.188431	0.817746
9829_91_S	finngen_R	outcome	9829_91_S	Inverse va	5	-0.03067	0.169009	0.855983
9829_91_S	finngen_R	outcome	9829_91_S	Simple mc	5	-0.36422	0.317169	0.314829
9829_91_S	finngen_R	outcome	9829_91_S	Weighted	5	0.122301	0.210085	0.591699
9837_60_N	finngen_R	outcome	9837_60_N	MR Egger	65	-0.0395	0.07282	0.589416
9837_60_N	finngen_R	outcome	9837_60_N	Weighted	65	-0.07385	0.061882	0.232691
9837_60_N	finngen_R	outcome	9837_60_N	Inverse va	65	-0.02448	0.042705	0.56641
9837_60_N	finngen_R	outcome	9837_60_N	Simple mc	65	-0.05341	0.108662	0.62477
9837_60_N	finngen_R	outcome	9837_60_N	Weighted	65	-0.09257	0.068561	0.181692
9848_22_C	finngen_R	outcome	9848_22_C	Wald ratic	1	-0.94505	0.413187	0.022182
9869_28_N	finngen_R	outcome	9869_28_N	Wald ratic	1	0.210164	0.54365	0.699067
9883_29_C	finngen_R	outcome	9883_29_C	MR Egger	4	-0.07385	0.57084	0.9089
9883_29_C	finngen_R	outcome	9883_29_C	Weighted	4	0.123719	0.299233	0.679274
9883_29_C	finngen_R	outcome	9883_29_C	Inverse va	4	0.175249	0.249689	0.48276
9883_29_C	finngen_R	outcome	9883_29_C	Simple mc	4	-0.01562	0.424492	0.972961
9883_29_C	finngen_R	outcome	9883_29_C	Weighted	4	-0.00013	0.331929	0.999702
9884_8_PF	finngen_R	outcome	9884_8_PF	MR Egger	63	0.033751	0.077563	0.664989
9884_8_PF	finngen_R	outcome	9884_8_PF	Weighted	63	0.074623	0.07867	0.342844
9884_8_PF	finngen_R	outcome	9884_8_PF	Inverse va	63	0.009441	0.053005	0.858635
9884_8_PF	finngen_R	outcome	9884_8_PF	Simple mc	63	0.148015	0.181705	0.418427
9884_8_PF	finngen_R	outcome	9884_8_PF	Weighted	63	0.060181	0.078122	0.444021
9901_28_E	finngen_R	outcome	9901_28_E	MR Egger	35	0.117459	0.172618	0.500963
9901_28_E	finngen_R	outcome	9901_28_E	Weighted	35	0.017173	0.129785	0.894735
9901_28_E	finngen_R	outcome	9901_28_E	Inverse va	35	0.0712	0.098145	0.468172
9901_28_E	finngen_R	outcome	9901_28_E	Simple mc	35	-0.17634	0.219403	0.427128
9901_28_E	finngen_R	outcome	9901_28_E	Weighted	35	0.001167	0.168639	0.994517
9906_21_S	finngen_R	outcome	9906_21_S	MR Egger	65	-0.05716	0.063369	0.370491
9906_21_S	finngen_R	outcome	9906_21_S	Weighted	65	-0.06844	0.064144	0.285985
9906_21_S	finngen_R	outcome	9906_21_S	Inverse va	65	-0.0496	0.042022	0.237826
9906_21_S	finngen_R	outcome	9906_21_S	Simple mc	65	0.017606	0.112536	0.876175

9906_21_S	finngen_R	outcome	9906_21_S	Weighted	65	-0.04199	0.061573	0.49773
9916_146_	finngen_R	outcome	9916_146_	MR Egger	6	0.514918	0.640217	0.466313
9916_146_	finngen_R	outcome	9916_146_	Weighted	6	-0.39401	0.334174	0.238374
9916_146_	finngen_R	outcome	9916_146_	Inverse va	6	-0.35542	0.263311	0.177078
9916_146_	finngen_R	outcome	9916_146_	Simple mc	6	-0.39713	0.504892	0.467165
9916_146_	finngen_R	outcome	9916_146_	Weighted	6	-0.40092	0.425914	0.389766
9950_229_	finngen_R	outcome	9950_229_	MR Egger	6	0.681556	0.725366	0.40062
9950_229_	finngen_R	outcome	9950_229_	Weighted	6	-0.29192	0.22886	0.202114
9950_229_	finngen_R	outcome	9950_229_	Inverse va	6	-0.04909	0.2744	0.858009
9950_229_	finngen_R	outcome	9950_229_	Simple mc	6	-0.31561	0.345832	0.403307
9950_229_	finngen_R	outcome	9950_229_	Weighted	6	-0.38082	0.238172	0.170729
9986_14_N	finngen_R	outcome	9986_14_N	MR Egger	38	-0.01906	0.082291	0.818134
9986_14_N	finngen_R	outcome	9986_14_N	Weighted	38	0.0348	0.075698	0.645715
9986_14_N	finngen_R	outcome	9986_14_N	Inverse va	38	-0.01279	0.052337	0.806917
9986_14_N	finngen_R	outcome	9986_14_N	Simple mc	38	0.099758	0.133673	0.460214
9986_14_N	finngen_R	outcome	9986_14_N	Weighted	38	0.014747	0.064127	0.81938
9995_6_DI	finngen_R	outcome	9995_6_DI	Wald ratic	1	-0.53022	0.595131	0.372965
